# The IPEA dilemma in CASPT2†
†Electronic supplementary information (ESI) available: Original data (Table S1) and additional comments for the literature survey; note on symmetry (Table S2), geometries (Table S3), data (Tables S4–S6) and comments (Section S2) for calculations on di-/triatomic molecules; results (Tables S7–S25) and comments (Section S3) for calculations on the organic molecular data set. See DOI: 10.1039/c6sc03759c


**DOI:** 10.1039/c6sc03759c

**Published:** 2016-09-26

**Authors:** J. Patrick Zobel, Juan J. Nogueira, Leticia González

**Affiliations:** a Institute of Theoretical Chemistry , Faculty of Chemistry , University of Vienna , Währinger Straße 17 , 1090 Vienna , Austria . Email: nogueira.perez.juanjose@univie.ac.at ; Email: leticia.gonzalez@univie.ac.at

## Abstract

Multi-configurational second order perturbation theory (CASPT2) has become a very popular method for describing excited-state properties since its development in 1990. To account for systematic errors found in the calculation of dissociation energies, an empirical correction applied to the zeroth-order Hamiltonian, called the IPEA shift, was introduced in 2004. The errors were attributed to an unbalanced description of open-shell *versus* closed-shell electronic states and is believed to also lead to an underestimation of excitation energies. Here we show that the use of the IPEA shift is not justified and the IPEA should not be used to calculate excited states, at least for organic chromophores. This conclusion is the result of three extensive analyses. Firstly, we survey the literature for excitation energies of organic molecules that have been calculated with the unmodified CASPT2 method. We find that the excitation energies of 356 reference values are negligibly underestimated by 0.02 eV. This value is an order of magnitude smaller than the expected error based on the calculation of dissociation energies. Secondly, we perform benchmark full configuration interaction calculations on 137 states of 13 di- and triatomic molecules and compare the results with CASPT2. Also in this case, the excited states are underestimated by only 0.05 eV. Finally, we perform CASPT2 calculations with different IPEA shift values on 309 excited states of 28 organic small and medium-sized organic chromophores. We demonstrate that the size of the IPEA correction scales with the amount of dynamical correlation energy (and thus with the size of the system), and gets immoderate already for the molecules considered here, leading to an overestimation of the excitation energies. It is also found that the IPEA correction strongly depends on the size of the basis set. The dependency on both the size of the system and of the basis set, contradicts the idea of a universal IPEA shift which is able to compensate for systematic CASPT2 errors in the calculation of excited states.

## Abstract 2

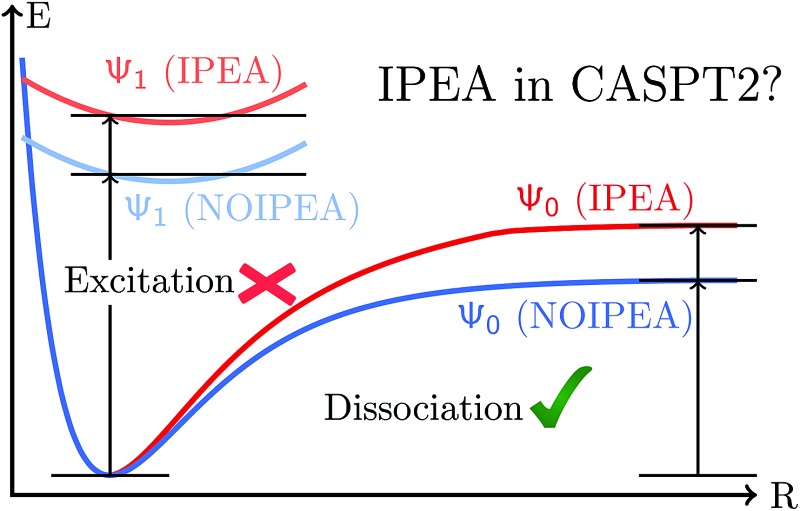
We show that the use of the IPEA correction in CASPT2 for excited state calculations of organic chromophores is not justified.

## 1 Introduction

The toolbox of modern computational chemistry contains a great variety of methods. While semi-empirical methods are fitted to give good performance for individual tasks, *ab initio* methods are driven to be parameter-free and provide similar performance regardless of the molecule and process under study. In practice, though, the nature of the problem dictates the choice of a particular *ab initio* method. The ground state properties of a molecule at its equilibrium geometry are usually well described by single-configurational methods. However, as soon as we leave this safe harbor, for example, when dealing with electronically excited states or dissociation, many configurations are likely to be needed.

Ideally, multi-configurational problems would be solved by a full configuration interaction (FCI) calculation. However, due to its factorial scaling with the number of electrons, FCI calculations are restricted to systems with few electrons and modest basis sets. As a compromise between accuracy and cost, one can select only the most important configurations, *e.g.*, within the complete-active-space self-consistent field (CASSCF) method,^1^ where FCI is performed only in a subspace of active orbitals. CASSCF provides a qualitatively good description for many multi-configurational problems,^2^ but CASSCF energies are typically not accurate. For this reason, CASSCF is almost routinely accompanied by a subsequent CI expansion – resulting in the so-called multi-reference configuration interaction (MRCI)^3^ – or considered as a zeroth-order reference function in a perturbation expansion. The second-order expansion developed by Roos and co-workers, widely known as CASPT2,^4,5^ is a prominent example of the latter. CASPT2 uses a combination of projection operators and an effective one-electron operator in its zeroth-order Hamiltonian. Different choices of projection operators^6–10^ have given rise to similar approaches and there are also CASSCF-based perturbation theory methods including two-electron terms in the zeroth-order Hamiltonian,^11^ *e.g.*, the second-order *n*-electron valence state perturbation theory (NEVPT2) method.^12,13^

Since the initial implementation of CASPT2 (ref. 4 and 5) in the MOLCAS program package,^14^ three important additions have been introduced. One is the multi-state CASPT2 (MS-CASPT2) variant^15^ to remedy problems encountered at avoided crossings or when there are other nonphysical mixings at the CASSCF level, *e.g.*, between excited valence and Rydberg states. The second addition includes different shift techniques,^16–18^ introduced to remove coupling with the so-called “intruder states”. Intruder states are states in the first-order wave function expansion with energies close to that of the reference state. Their presence leads to small denominators in the second-order energy expression and eventually to nonphysical artifacts in the potential energy surfaces or even divergence of the perturbation expansion at certain points. The shifts used to suppress their coupling to the reference state are added to the zeroth-order Hamiltonian and affect the computation of the first-order wave function and second-order energy contributions. For the energy, their effect is approximately removed after the intruder states are handled. The third addition is the so-called IPEA shift,^19^ introduced to correct systematic errors observed in systems with open-shell electronic states. An optimal IPEA shift value was determined through fitting against experimental data. This shift is added to the zeroth-order Hamiltonian in the computation of the first-order wave function and second-order energy contributions but it affects only the properties of open-shell states. Its usage is recommended in the standard CASPT2 procedure and it has been employed by default in the MOLCAS package since version 6.4 (released in 2006).

In this contribution, we critically examine the actual performance of CASPT2 in describing both open- and closed-shell states and systematically investigate the effect of the IPEA shift on excitation energies. The observation that many excited states of organic molecules calculated by our group are better described without the IPEA shift, see *e.g.* ref. 20 and 21, and that a large number of recent CASPT2 studies do not use the IPEA shift, see *e.g.* ref. 22–45, encouraged us to perform a comprehensive literature survey on past excited-state CASPT2 calculations of organic molecules carried out before the IPEA shift was introduced and to evaluate systematic errors. Further, we tested the performance of CASPT2 against FCI reference data for di- and triatomic molecules, as well as against experimental values and other theoretical methods for medium-sized organic molecules using the Thiel test set.^46^ The results show that, on average, CASPT2 slightly underestimates excitation energies of di- and triatomic molecules and the IPEA shift corrects for this error only partially. With increasing molecular size, however, the effect of the IPEA shift becomes excessive and predicted excitation energies are too high. In general, therefore, already for small- and medium-sized organic molecules the use of the IPEA shift in the calculation of excitation energies is not justified.

## 2 Theory

### 2.1 CASPT2 in a nutshell

For the discussion below, it is convenient to briefly revise a few aspects of the CASPT2 theory; further details can be found elsewhere.^47–49^ In the CASPT2 formulation of Andersson, Roos and co-workers,^4,5^ the zeroth-order Hamiltonian is given by1
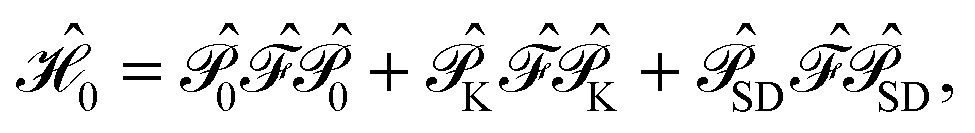
where 
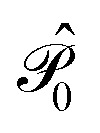
 is the operator that projects on the CASSCF reference function |*Ψ* ^(0)^, 
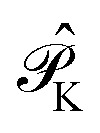
 projects on the orthogonal complements to |*Ψ* ^(0)^ generated by excitations in the active space, and 
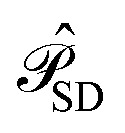
 projects on the first-order interaction space {*Φ*} generated by all single and double excitations not projected on by 
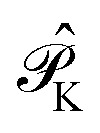
. Higher-order excitations do not contribute to the second-order energy. 
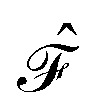
 is an effective one-electron operator, called the generalized Fock operator, which is a sum of matrix elements *f*_*pq*_ and spin-averaged excitation operators *Ê*_*pq*_. It reads2
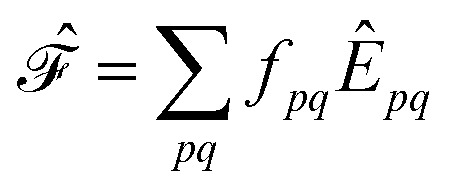

3
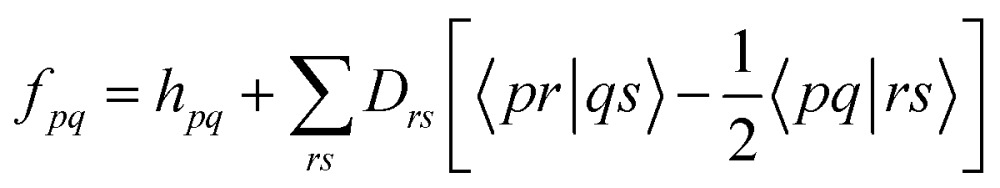

4
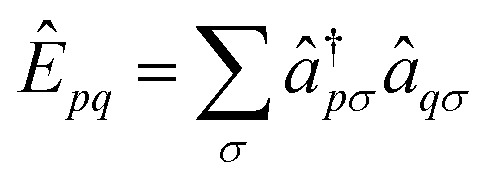
where *D* is the one-particle density matrix. The generalized Fock matrix *f* consists of 3 × 3 blocks corresponding to the three orbital subspaces if we order the index *p* by inactive (*D*_*pp*_ = 2), active (0 ≤ *D*_*pp*_ ≤ 2), and secondary (*D*_*pp*_ = 0) orbitals. The coupling between the inactive and secondary blocks is zero according to the generalized Brillouin theorem. The inactive–inactive, active–active, and secondary–secondary blocks may be diagonalized; in general, however, *f* is non-diagonal. The non-zero coupling blocks were neglected in the first (diagonal) CASPT2 formulation,^4^ but accounted for in all subsequent formulations.^5^ The first-order wave function and second-order energy contributions are given by5
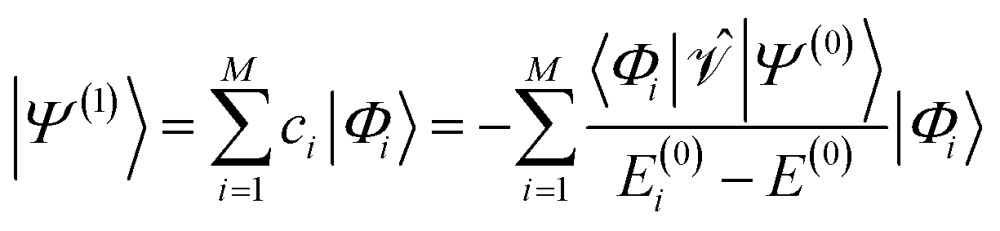

6
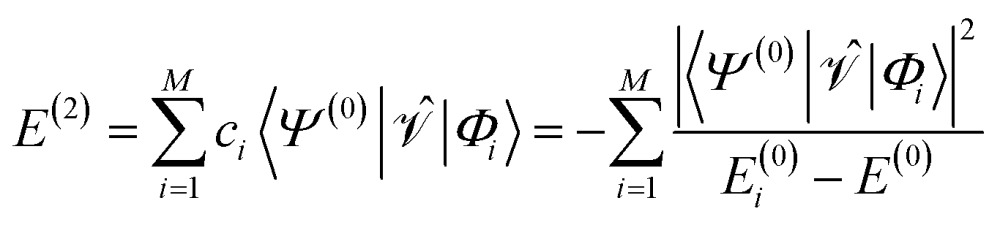
where the perturbation is given by 
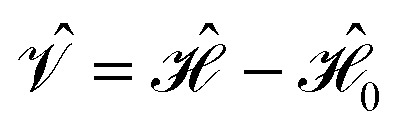
 and the first-order interaction space components satisfy 
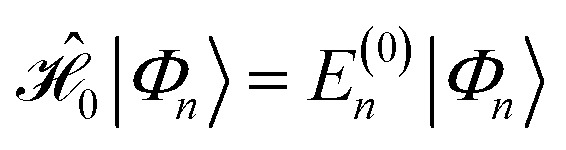
. For low-lying reference states |*Ψ* ^(0)^, typically *E*(0)*i* – *E* ^(0)^ ≥ 0, and, thus, *E* ^(2)^ < 0.

### 2.2 The IPEA shift

The first report on systematic errors in CASPT2 was given by Andersson and Roos^50^ for equilibrium geometries and atomization energies of a set of 32 small molecules calculated with extended atomic natural orbital (ANO) basis sets. Equilibrium geometries agreed well with experimental values but the atomization energies were underestimated. It was observed that the error scaled by 3–6 kcal mol^–1^ (0.13–0.26 eV) times the difference between the number of paired electrons within the molecule and within the atoms. For example, the total error in the atomization energy of CO_2_ amounted to 13.0 kcal mol^–1^ and the difference number of electron pairs between the CO_2_ molecule and its atomic fragments C, O, and O is 3. This error was later^51^ ascribed to an energetic favoring of wave functions dominated by open-shell configurations over those dominated by closed-shell configurations. In order to alleviate the unbalanced description of open- and closed-shell configurations, three modifications to the zeroth-order Hamiltonian were suggested^51^ that added correction terms to the generalized Fock operator 
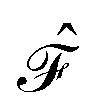
. However, as these modifications provided only minor improvements, they were hardly employed later on.^52,53^

In 2004, an explanation for the underestimation of open-shell energies was suggested upon inspection of the diagonal elements of the generalized Fock matrix *f*.^19^ Analogous to Koopmans' theorem – proposed for the single-configuration case – the diagonal elements of *f* for inactive and secondary orbitals can be associated with negative ionization potentials –(IP)_*p*_ and electron affinities –(EA)_*p*_, respectively, assuming that the couplings between the inactive/active and active/secondary blocks in the nondiagonal generalized Fock matrix are neglected.^19^ For active orbitals, the diagonal elements of *f* may be written as weighted averages of –(IP)_*p*_ and –(EA)_*p*_, so that7
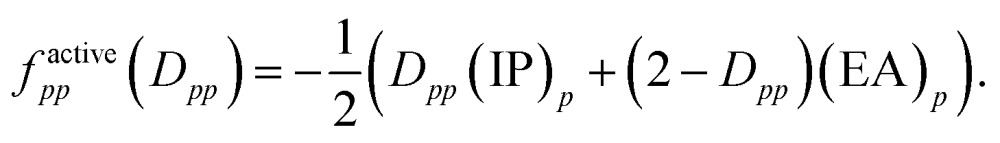



Accordingly, for doubly occupied active orbitals,

8 *f*active*pp*(*D*_*pp*_ = 2) = –(IP)_*p*_

and for empty active orbitals,

9 *f*active*pp*(*D*_*pp*_ = 0) = –(EA)_*p*_.

For singly occupied orbitals, *f*active*pp* is10
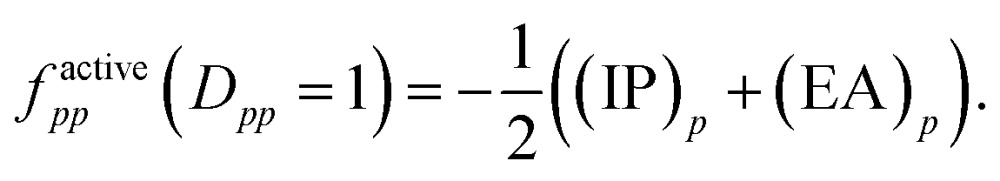



Then, it was asserted that “this feature of the [generalized] Fock operator will lead to denominators in the expression for the second-order energy that are too small in the case of excitation into or out from a partially occupied orbital”.^19^ Thus, it was assumed that the systematic lowering of the energies of the open-shell states was due to these denominators being too small. As a remedy, a modification in the zeroth-order Hamiltonian was suggested in order to yield diagonal elements *f*active*pp* that resemble negative ionization potentials and electron affinities also for singly occupied orbitals. This was realized by adding a shift *σ*(EA)*p* to *f*active*pp* when exciting into an active orbital, so that the shifted matrix element reads11
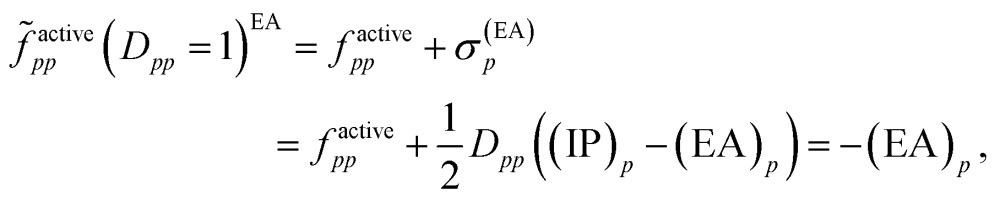
and adding a shift *σ*(IP)*p* to *f*active*pp* when exciting out of an active orbital, so that12
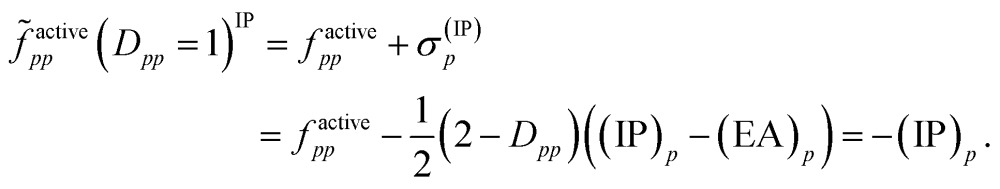



Since both shifts, *σ*(EA)*p* and *σ*(IP)*p*, depend only on the difference (IP)_*p*_ – (EA)_*p*_ and it is not clear how to determine the individual (IP)_*p*_ and (EA)_*p*_ values, this difference was replaced by an average shift parameter *ε*, so that13
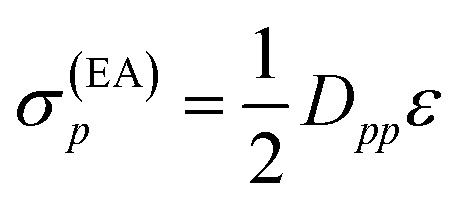

14
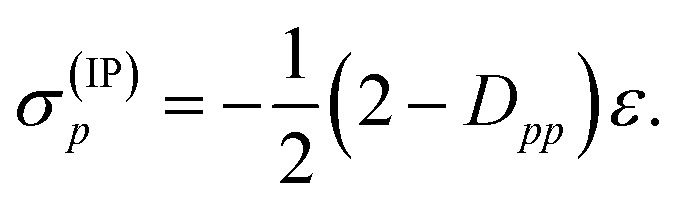



For *ε* > 0, the shift will always lead to larger denominators in the second-order energy expression for open-shell states resulting in larger total energies. This becomes apparent when one splits up the sum of eqn (6) into terms belonging to closed-shell configurations (index *i*) and terms belonging to open-shell configurations (index *j*),15
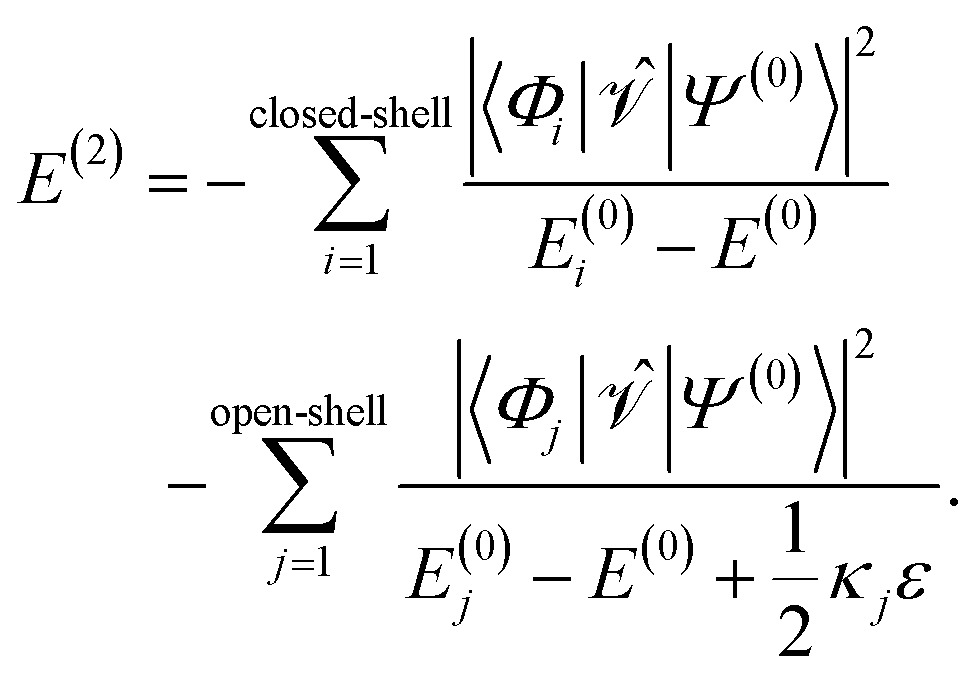



For the elements of the first partial sum, *D*_*pp*_ = 0 for *σ*(EA)*p* and *D*_*pp*_ = 2 for *σ*(IP)*p*, meaning that no shift *ε* is added. But for the elements in the second sum, a shift is added. The prefactor *κ*_*j*_ can take the values 0, 1, and 2, depending on the excitation class of *Φ*_*j*_ (see ref. 54 but note the different sign convention in the denominator of *E* ^(2)^). For example, in the case where two electrons are promoted from inactive orbitals *φ*_a_ and *φ*_b_ to active orbitals *φ*_*p*_ and *φ*_*q*_, *κ*_*j*_ is 0 if *D*_*pp*_ = *D*_*qq*_ = 0, *κ*_*j*_ = 1 if either *D*_*pp*_ = 1 and *D*_*qq*_ = 0 or *vice versa*, and *κ*_*j*_ = 2 if *D*_*pp*_ = *D*_*qq*_ = 1. Naturally, *D*_*pp*_ and *D*_*qq*_ cannot equal 2, if an electron should be promoted into the orbitals *φ*_*p*_ and *φ*_*q*_. So-defined, the shift does not affect electronic states that are composed mainly of closed-shell configurations, as most ground states of organic molecules at their equilibrium geometry are. In this case, the most important terms in the second-order energy contribution are those in the first sum of eqn (15). However, states with open-shell character, such as excited states or states at the dissociation limit, possess important contributions in the second sum. Therefore, due to the shift, the absolute value of the second-order energy contribution is smaller, thereby increasing the total energies of such states.

To determine an optimal value of the effective IPEA shift parameter *ε*, Roos and co-workers^19^ calculated the dissociation energies of 49 diatomic molecules at the CASPT2 level using values of *ε* ranging from 0 to 0.5 a.u., extended ANO-RCC basis sets^53,55^ and active spaces comprising all valence electrons. Most of the CASPT2 dissociation energies computed without the IPEA shift underestimated the experimental values. Individual errors peaked around 0.5 eV for the triply-bonded dimers N_2_, P_2_, and As_2_ supporting the initial assumption that the error scales with the difference in number of paired open shells by 0.13–0.26 eV (the pnictogen atoms possess three unpaired electrons in their ground-state electronic configurations).^50^ The root-mean-square (RMS) deviation of all dissociation energies calculated without a shift amounted to 0.22 eV. In contrast, a shift of *ε* = 0.25 a.u. led to the minimal RMS value of 0.09 eV.

The influence of the shift was further tested for equilibrium geometries (*r*_e_) and vibrational frequencies (*ω*_e_ and *ω*_e_ *χ*_e_) for some of the diatomic molecules in their ground and excited states. The tests suggested optimal parameters of *ε* = 0.1 a.u. (*ω*_e_ *χ*_e_) and *ε* ≥ 0.5 a.u. (*r*_e_ and *ω*_e_), but the RMS deviations compared to the experimental data were already small without using the IPEA shift. Furthermore, adiabatic excitation energies for four excited states of N_2_ and vertical excitation energies for four excited states of benzene were computed. For N_2_, the best agreement with experimental results was achieved for *ε* ≥ 0.4 a.u., while for benzene, the optimal shift was *ε* = 0.1 a.u. for the largest active space considered. In addition, the ionization potentials of the 3d transition metals were computed using a shift of *ε* = 0.25 a.u. and good agreement with the experimental results was observed.

From all of these results, it was concluded that a shift of *ε* = 0.25 a.u. represented the optimal value for CASPT2 calculations to be able to correct the systematic error inherent to open-shell states. This value, coincidentally, resembles the average atomic value of the quantity (IP –EA) when going through the periodic table, which was seen as a good omen to give some physical motivation to the size of the IPEA shift.^47^

## 3 Deviations of CASPT2 excitation energies: a literature survey

**Fig. 1:**
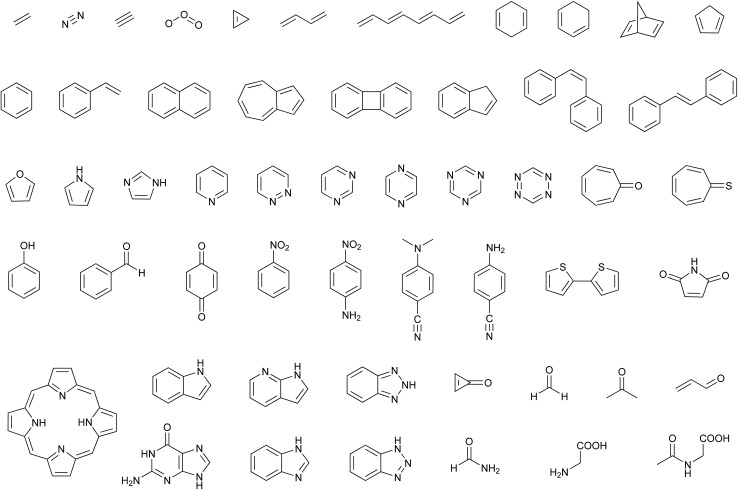
Molecules considered in the literature survey.

The introduction of the IPEA shift in CASPT2 was motivated by a systematic underestimation in dissociation energies ascribed to originate from a general underestimation of energies of open-shell states.^19,50^ This underestimation of open-shell state energies was believed to be present also when calculating excitation energies. Since we could not find any study demonstrating a systematic underestimation of excitation energies themselves, we performed a survey to collect vertical excitation energies computed with CASPT2 up to 2004 – when the IPEA shift was introduced. The energies of 356 excited states of 53 organic molecules^15,17,51,52,56–91^ for which experimental data is available (see Fig. 1) have been collected. How publications were selected is explained in Section S1 of the ESI.† Table 1 lists the mean signed error of the excitation energies (MSEE) and mean unsigned error of the excitation energies (MUEE) between the computed and experimental data. The corresponding calculated and experimental excitation energies for each state are collected in Table S1 of the ESI.†

**Table 1:** Mean signed error (MSEE) and mean unsigned error (MUEE) in eV for the CASPT2 vertical excitation energies *V*calc*i* compared to experimental excitation energies *V*exp*i* of the 53 organic molecules shown in Fig. 1

States^*a*^	Environment	Method^*b*^	*N*_States_	MSEE^*c*^	MUEE^*d*^
Any	Any	Any	356	–0.02	0.16
Any	Gas phase	Any	295	–0.03	0.15
Any	Gas phase	Standard	163	–0.04	0.14
Valence	Any	Any	247	–0.02	0.17
Valence	Gas phase	Any	196	–0.03	0.17
Valence	Gas phase	Standard	93	–0.07	0.17
Rydberg	Any	Any	109	–0.01	0.10
Rydberg	Gas phase	Any	99	–0.03	0.09
Rydberg	Gas phase	Standard	70	–0.03	0.08

^*a*^Valence and Rydberg states.

^*b*^Standard refers to the original nondiagonal CASPT2 implementation.^5^

^*c*^Mean signed error of excitation energies in eV, computed as 
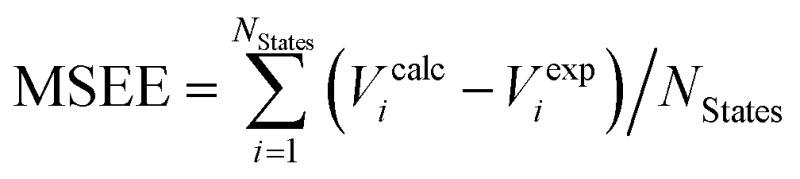
.

^*d*^Mean unsigned error of excitation energies in eV, computed as 
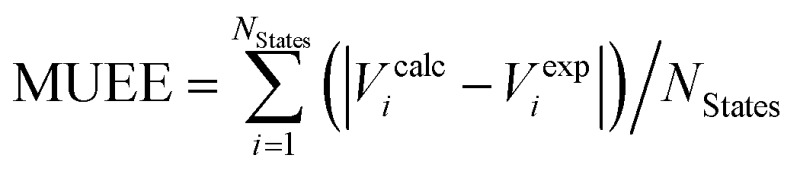
.

Conspicuously, CASPT2 seems to underestimate the vertical excitation energies of the organic molecules very slightly. Since the ground state of organic molecules is typically a closed-shell state and most of the low-lying excited states are described by a single excitation, *i.e.*, one electron pair is unpaired, the excitation energies are expected to be underestimated by 0.13–0.26 eV. In contrast, the MSEE for all 356 states amounts only to –0.02 eV, an order of magnitude smaller.

Most of the calculations surveyed were performed in the gas phase. However, the experimental reference data of some excited states was only available in the condensed phase. If we exclude such states, *i.e.*, we restrict ourselves to cases where both experimental and theoretical studies were conducted in the gas phase (295 states), still a MSEE of only –0.03 eV is obtained.

Next, we may exclude all data obtained by non-standard CASPT2 calculations. Non-standard refers to the usage of MS-CASPT2,^15^ level-shift (LS) corrected CASPT2,^16^ and diagonal CASPT2.^4^ The former two are meant to be used for troublesome systems and the latter is a lower-level approximation. Even in this case, restricting to data solely obtained by standard non-diagonal CASPT2 (163 states), the MSEE is only –0.04 eV.

One could also separate the valence from Rydberg states, since the latter are usually more difficult to describe.^92^ In this case, the valence excited states are underestimated by 0.02 eV or 0.03 eV, for any environment or for the gas phase, respectively, both computed with any variant of CASPT2. The deviation for the gas phase valence states computed solely with the standard CASPT2 (93 states) is larger, –0.07 eV, but still 2–4 times smaller than expected from the errors reported for dissociation energies. For Rydberg states, the underestimation of the excitation energies amounts only to –0.03 eV for the gas phase results using the standard CASPT2 method (70 states).

Taking into account all states, it is gratifying to see that the MUEE value is below 0.2 eV, which is considered the error of CASPT2 in predicting excitation energies. Most importantly, none of the MSEE values reported shows the general underestimation of the CASPT2 energies for open-shell states scaling with 0.13–0.26 eV times the number of open shells (NOS), as predicted in ref. 50 for dissociation energies. These results imply that the error present in the dissociation energies found by Roos *et al.* ^50^ has a different source, and is fortuitously cancelled out when computing excitation energies.

## 4 Full CI benchmark against CASPT2 excitation energies for di- and triatomic molecules

In the last section, we evaluated the performance of CASPT2 in predicting vertical excitation energies through comparisons with experimental reference data. Despite such comparisons being common practice, neglecting effects such as vibronic couplings or intermolecular interactions present in the experiment could translate into unpredictable errors in the computed value. Therefore, in order to allow for a comparison of the very same well-defined property, in this section we compare calculated CASPT2 electronic states to a FCI benchmark, which can be considered exact, disregarding the finite size of the basis set. To this end, we selected a number of small molecules for which we calculated the lowest-lying electronic states with FCI and CASPT2: the first-row hydrides HLi, HBe, HB, HC, HN, HO, and HF and the homodiatomics Li_2_, B_2_, C_2_, and N_2_, as well as the triatomic molecules H_2_O and CH_2_. In our comparison between the CASPT2 and FCI results, we analyze not only the excitation energies but also the total energies in order to get a better understanding of the IPEA correction. Unlike the underlying CASSCF calculation, CASPT2 is a non-variational method, *i.e.*, the CASPT2 total energies for each state are not bound from below to the exact energies obtained by FCI. However, as we will see, the errors of the CASPT2 total energies compared to FCI are very small, indicating that CASPT2 is able to almost quantitatively reproduce the FCI results for our benchmark set.

### 4.1 Computational details

The equilibrium distances of the diatomic molecules were taken from the NIST database.^93^ The geometries of the triatomic molecules were taken from previous FCI studies.^94,95^ All geometries are listed in Table S3 in the ESI.† Given the computational cost, the FCI calculations were restricted to the 6-31G and 6-311G basis sets.^96,97^ The frozen-core approximation was employed for the homodiatomics as well as for H_2_O and CH_2_; so, strictly speaking, only the first-row hydrides were treated with FCI. As starting orbitals we used the results from restricted and unrestricted Hartree–Fock calculations for molecules with closed-shell and open-shell ground states, respectively. The molecular symmetry was restricted to *D*_2h_ or *C*_2ν_ for the homodiatomics and first-row hydrides, respectively, while full *C*_2ν_ symmetry for H_2_O and CH_2_ was employed. All FCI calculations were performed using the MOLPRO version 2012.1.^98^

CASPT2 and preceding CASSCF calculations were done using the same geometries and basis sets. We used the combination of state-averaged CASSCF and MS-CASPT2 for the cases where several electronic states of the same symmetry were calculated, with equal weights for all the states considered. Initially, restricted and unrestricted Hartree–Fock calculations were performed to obtain the same set of starting orbitals as in the FCI calculations. The active spaces in the CASSCF calculations always comprised all valence orbitals and electrons, that is, the 1s shell of hydrogen as well as the 2s and 2p shells of all first-row atoms. We used the frozen-core approximation for the same molecules as in the FCI calculations. All CASPT2 calculations were performed for two cases: (i) using the standard unshifted zeroth-order Hamiltonian of Andersson *et al.* ^5^ and (ii) using the IPEA-shifted zeroth-order Hamiltonian^19^ with the recommended shift value of *ε* = 0.25 a.u. Henceforth, we will refer to the results of both CASPT2 variants by NOIPEA and IPEA CASPT2, respectively. These calculations were performed using MOLCAS version 8.0.15-05-24.^99^ Further details are given in Section S2.1 of the ESI.†

### 4.2 Full CI excitation energies *versus* CASSCF and CASPT2

A total of 137 different electronic states of 13 di- and triatomic molecules have been calculated at the CASPT2/CASSCF and FCI levels of theory. Energies are listed in Tables S4 and S5 of the ESI.† For all states the mean unsigned and signed errors of the vertical CASSCF and CASPT2 excitation energies (MUEE and MSEE) as well as of the total energies (MUET and MSET) with respect to the FCI values are reported in Table 2. The assignment of whether a state is open-shell or closed-shell is explained in Section S2.2 of the ESI.†

**Table 2:** Mean unsigned error (MUEE) and mean signed error (MSEE) of the excitation energies in eV as well as mean unsigned error (MUET) and mean signed error (MSET) of the total energies in eV for the CASSCF and CASPT2 results compared to the FCI energies for the ground and excited states of 13 di- and triatomic molecules. The MUET for closed-shell and open-shell states as well as ground and excited states is presented separately. Total and excitation energies are given by *E*_*i*_ and *V*_*i*_, respectively

Basis set	*N*_States_	6-31G			6-311G		
Method		CASSCF	NOIPEA	IPEA	CASSCF	NOIPEA	IPEA
MUET^*a*^	137	1.34	0.14	0.19	1.84	0.20	0.25
MUEE^*b*^	124	0.28	0.08	0.06	0.38	0.11	0.10
MSET^*c*^	137	1.34	0.13	0.18	1.84	0.19	0.25
MSEE^*d*^	124	–0.12	–0.05	–0.03	–0.18	–0.05	–0.03
MUET_all states_ ^*a*^	149^*e*^	1.35	0.14	0.19	1.84	0.19	0.25
MUET_ground state_ ^*a*^	13^*e*^	1.49	0.17	0.19	2.05	0.23	0.27
MUET_excited state_ ^*a*^	136^*e*^	1.33	0.14	0.19	1.82	0.18	0.25
MUET_closed-shell_ ^*a*^	33^*e*^	1.35	0.17	0.21	1.90	0.23	0.28
MUET_open-shell_ ^*a*^	116^*e*^	1.35	0.13	0.18	1.82	0.17	0.24

^*a*^Computed as 
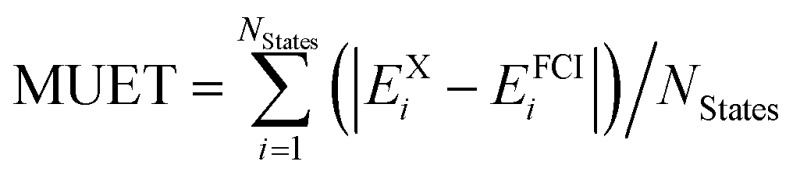
.

^*b*^Computed as 
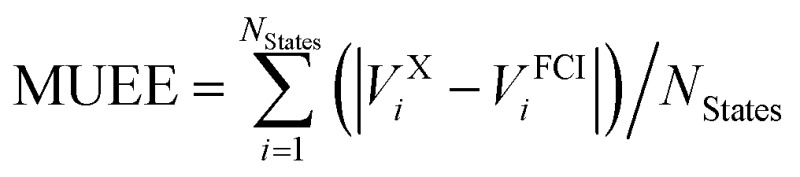
.

^*c*^Computed as 
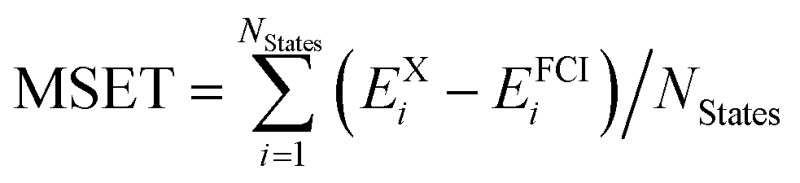
.

^*d*^Computed as 
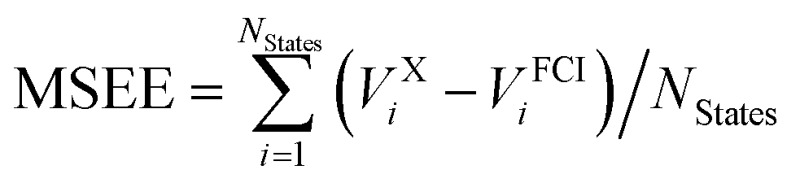
.

^*e*^Taking into account both components of all Δ states (see Sections S2.1 and S2.2 in the ESI for more details).

The first observation is that already the CASSCF excitation energies are in reasonable agreement with the FCI ones. Although the errors in the total energies are large (MUET values of 1.34 and 1.84 eV for the 6-31G and 6-311G basis sets, respectively), the MUEEs are considerably smaller due to error cancellation. For example, the MUET for the ground state using the 6-31G (6-311G) basis set is 1.49 eV (2.05 eV) and it is partially cancelled out by the MUET of 1.33 eV (1.82 eV) for the excited states, thus providing a MUEE of 0.28 eV (0.38 eV). The MSEE for CASSCF is around –0.15 eV for both basis sets, *i.e.*, CASSCF underestimates vertical excitation energies because the MUET for the ground states is larger than that for the excited states.

As expected, the use of CASPT2 without IPEA (labeled as NOIPEA in Table 2) improves the agreement with FCI with respect to CASSCF. The MUET for NOIPEA CASPT2 decreases to 0.14 eV (0.20 eV) for the 6-31G (6-311G) basis set. Similar to CASSCF, NOIPEA CASPT2 also underestimates the excitation energies, but to a lesser extent [compare MSEE –0.05 eV (–0.05 eV) *versus* –0.12 eV (–0.18 eV) in CASSCF]. The errors in the total energies for the ground [MUET 0.17 eV (0.23 eV)] and excited states [MUET 0.14 eV (0.18 eV)] are also more similar to each other resulting in excitation energies closer to the FCI ones. Interestingly, the reason why CASPT2 performs better than CASSCF with respect to FCI for this benchmark set is not trivial. Typically, CASSCF overestimates excitation energies and CASPT2 decreases the error because it lowers the excitation energies. This behavior is due to the following argument: for low-lying electronic states, the second-order correction to the energy included in a CASPT2 treatment is negative (recall Section 2.1), *i.e.*, it lowers the total energy. As the size of the correction is inversely proportional to the energy difference between the CASSCF reference state and the first-order interaction space states (see eqn (6)), one assumes that the correction is larger for higher-lying electronic states since they exhibit a smaller difference in energy to the first-order interaction space states. Thus, the energy of excited states should be more stabilized than the energy of ground states which, in turn, decreases the excitation energy. A closer look into our energies reveals that, contrary to what would have been expected, CASSCF underestimates the excitation energies and CASPT2 increases them. This means that the second-order energy correction is larger for the ground state than for the excited states suggesting that it is the size of the numerators which describe the coupling of the reference states and the first-order interaction space states over the perturbation operator 
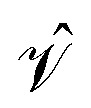
 (eqn (6)), which determines the size of the energy correction.

In general, the vertical excitation energies are underestimated with NOIPEA CASPT2 (MSEE = –0.05 eV). Note that this error is of the same size as the MSEE obtained for the valence excited states of the organic molecules included in our literature survey in the previous section. The inclusion of the IPEA shift decreases this error to MSEE = –0.03 eV. This fact can be understood by analyzing the errors of closed-shell and open-shell states in Table 2. For the 6-31G basis set, the MUET for CASSCF is fortuitously the same for closed- and open-shell states (1.35 eV) but for the larger 6-311G basis set, the MUET of closed-shell states is *ca.* 0.1 eV larger than for open-shell states. A larger error for closed-shell states is also found when using either NOIPEA or IPEA CASPT2, regardless of the basis set. This unbalanced description between closed-shell and open-shell states is precisely the reason for the MUETs in the ground and excited states. Ten out of 13 molecules considered here have closed-shell ground states (only NH, B_2_, and CH_2_ possess an open-shell ground state), while the majority of the excited states possess a larger open-shell character. Thus, a larger error is found for the ground states compared to the excited states when using either NOIPEA or IPEA CASPT2. Compared to NOIPEA, the IPEA variant increases the MUETs for both the ground and excited states, but the increase is larger for the excited states, thus reducing the error in the excitation energies.

**Fig. 2:**
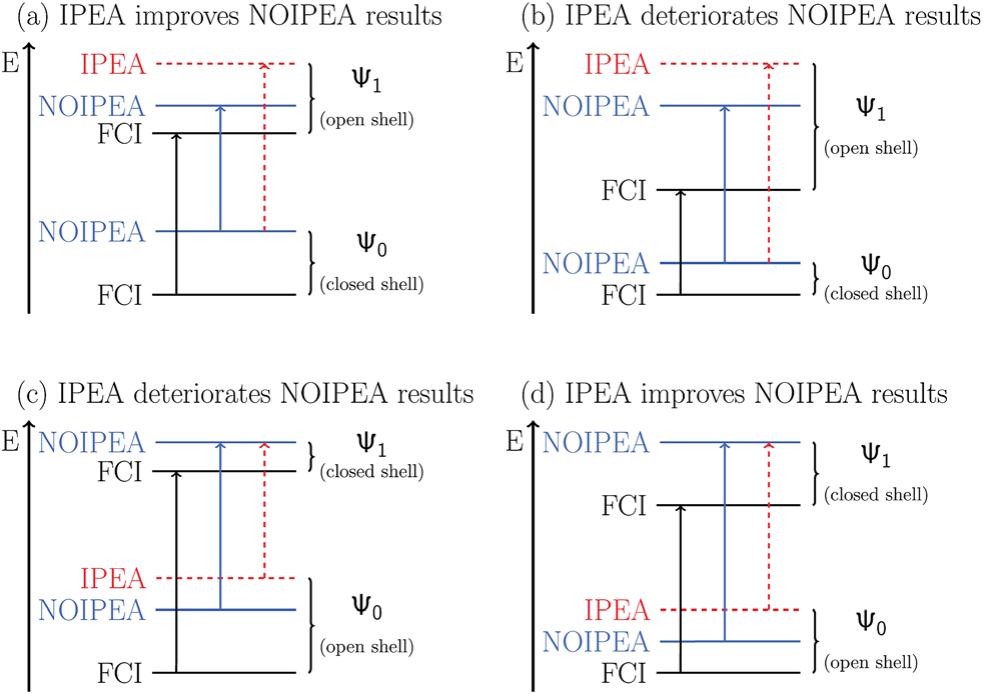
Comparison of CASPT2 results with respect to FCI results using the NOIPEA and IPEA variants. Horizontal lines represent energy levels and vertical arrows represent energy differences.

Gratifyingly, the mean errors in the total energies of CASPT2 compared to FCI are very small. These are typically positive and of the size of 0.01–0.02% of the total energy for the small basis sets. For application purposes, however, chemistry is usually more interested in obtaining accurate relative energies between different states than total energies. As the errors in total energies are usually positive, one can easily predict when the IPEA-modified CASPT2 will give a better agreement than the standard NOIPEA-CASPT2 for vertical excitation energies. If two states have a similar closed-shell or open-shell character, IPEA should not affect the energy difference between both states as IPEA should increase the error in the energy for both states evenhandedly. But if both states differ in the NOS, four different situations, depicted in Fig. 2, are possible. If the ground state is closed-shell and the excited state is open-shell [cases (a) and (b)], IPEA will yield a better relative energy between these two states when the error in the energy is larger for the ground state [case (a)] than for the excited state [case (b)]. In case (a), IPEA will increase the error in the excited state and, if the increase is not too large, both errors will cancel each other when calculating the energy difference between both states. Similarly, when the ground state is open-shell and the excited-state is of a closed-shell type [cases (c) and (d)], a better agreement in the vertical excitation energies will be achieved if the error is larger in the excited state [case (d)]. If, however, the error is smaller for the closed-shell state (regardless if it is the ground state or excited state), the IPEA shift will enhance the error in the open-shell state, thus yielding a larger error in the relative energy [cases (b) and (c)].

Since in the molecules considered here the IPEA shift decreases the MSEE with respect to NOIPEA, one can conclude that cases (a) and (d) apply most often, *i.e.*, the error is smaller in the open-shell state when using the standard NOIPEA CASPT2. In turn, this means that the better agreement in the vertical excitation energies when using IPEA is due to an improved cancellation of errors, as IPEA increases the error in the energy of open-shell states. Case (a) is thereby more common since the majority of molecules considered possess a closed-shell ground state, as is the case with most organic molecules. Indeed, the preceding literature survey showed that NOIPEA underestimates vertical excitation energies of small and medium-sized organic molecules only slightly (as compared to experimental reference data). This suggests that the case most frequently encountered in organic molecules will be case (a), and one would expect that using the IPEA-modified CASPT2 method should give better excitation energies than the standard NOIPEA approach. We will test this assumption in Section 5; however, it is useful to analyze the size of the errors and the IPEA correction beforehand.

### 4.3 Error size *vs.* number of open shells

**Fig. 3:**
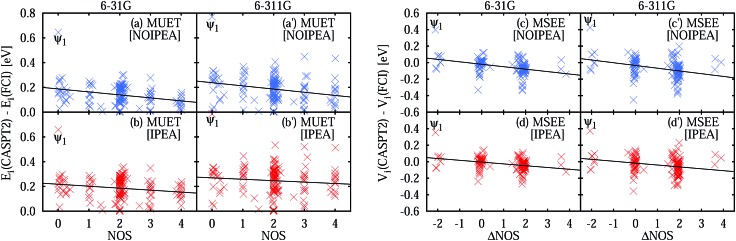
Unsigned errors in eV of the NOIPEA (a and a′) and IPEA (b and b′) CASPT2 total energies *E*_*i*_ compared to the FCI energies as a function of the number of open shells (NOS). Signed errors in eV of the NOIPEA (c and c′) and IPEA (d and d′) CASPT2 vertical excitation energies *V*_*i*_ compared to the FCI excitation energies as a function of the difference number in open shells (ΔNOS). The large error of the state labeled *Ψ*_1_ is discussed separately in Section S2.5 in the ESI.†

The IPEA shift was introduced under the assumption that CASPT2 systematically underestimates the energies of open-shell states thus leading to excitation energies that are too small. This assumption was based on the observation that CASPT2 predicted atomization energies of small molecules which were too low and that the error compared to experimental data scaled with the difference in the NOS between the molecule and its fragments. The latter observation is investigated with the help of Fig. 3, which shows the MUET of all calculated states as a function of the NOS and the MSEE of the excited states as a function of the difference number of open shells ΔNOS = NOS (excited state) – NOS (ground state). A linear fit is performed for each data set to identify trends, yielding the equations listed in Table 3.

**Table 3:** Linear fits *E* = *a* × NOS + *b* and *E* = *a* × ΔNOS + *b* for the MUETs and MSEEs for the 6-31G and 6-311G results, respectively (Fig. 3). Coefficients *a* and *b* given in eV

Basis set	6-31G	6-311G
MUET (NOIPEA)	–0.0241 × NOS + 0.1874	–0.0257 × NOS + 0.2378
MUET (IPEA)	–0.0159 × NOS + 0.2180	–0.0114 × NOS + 0.2696
MSEE (NOIPEA)	–0.0296 × ΔNOS – 0.0200	–0.0332 × ΔNOS – 0.0346
MSEE (IPEA)	–0.0218 × ΔNOS – 0.0048	–0.0229 × ΔNOS – 0.0172

From the linear fit of the MUET of NOIPEA (Fig. 3a and a′) it can be observed that on average the error of the CASPT2 energies with respect to FCI becomes smaller with increasing NOS, although the values are considerably spread around the linear fit line. Using the IPEA-modified CASPT2 variant, the errors in the total energies become larger and this increase is slightly more pronounced for states with more open shells, leading to a more balanced description of all the states. This is reflected in the much smaller slope of the linear fit lines of the MUET as a function of NOS for the IPEA data set (–0.0159 eV for 6-31G and –0.0114 eV for 6-311G) than for the NOIPEA data set (–0.0241 eV for 6-31G and –0.0257 eV for 6-311G), see Fig. 3b and b′ and Table 3.

The improved error cancellation for IPEA is also apparent in the vertical excitation energies (Fig. 3c, c′, d and d′). For both basis sets, the IPEA fit line possesses both a smaller slope (–0.0218 *vs.* –0.0296 eV for 6-31G and –0.0229 *vs.* –0.0332 eV for 6-311G) and a smaller intercept (–0.0048 *vs.* –0.0200 eV for 6-31G and –0.0172 *vs.* –0.0346 eV for 6-311G) at ΔNOS = 0, *i.e.*, the error in the excitation energies appears both smaller and more constant for different excitation types. We note the clustering of the data points around ΔNOS = 0 and 2, as well as the considerable spread around the fitted line for excited states with similar ΔNOS values. There are only few data points with ΔNOS = –2 or +4 because these types of states are sparse in our test set. Excited states with ΔNOS ≈ –2 are closed-shell excited states that belong to molecules with an open-shell ground state – NH, B_2_, and CH_2_ in our test set according to our definition of open-shell. Excited states with ΔNOS ≈ +4 describe double excitations where two electron pairs are unpaired. Both combinations are not often met for the excited states in our test set and are also not common for larger organic molecules. From this analysis, one would be tempted to conclude that, for larger organic molecules, a better error cancellation in the energies of electronic states of different character will apply when using the IPEA variant. However, as we will see in Section 5, the error of open-shell electronic states is increased too much with respect to the error of closed-shell states and thus this mechanism of error cancellation does not apply to organic molecules.

### 4.4 Size of the IPEA correction

**Fig. 4:**
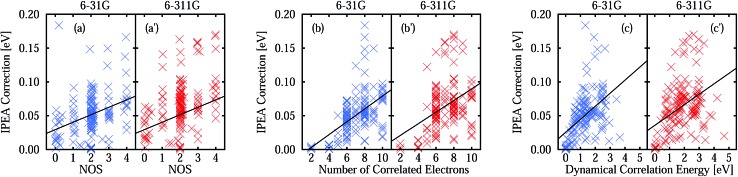
IPEA correction as a function of (a and a′) the number of open shells (NOS), (b and b′) the number of correlated electrons, and (c and c′) the dynamical correlation energy. The IPEA correction to a state *Ψ*_*i*_ is given as the difference *E*IPEA*i* – *E*NOIPEA*i* while the dynamical correlation energy is defined as *E*CASSCF*i* – *E*FCI*i*.

The better error cancellation for CASPT2 vertical excitation energies when using the IPEA shift is due to an increase in the energy of open-shell states. For open-shell states, the error of NOIPEA CASPT2 compared to the FCI total energies is typically smaller than for closed-shell states. As the errors in the total energies are positive throughout the states in our test set, the energy increase of open shells due to the IPEA shift partially cancels the difference in the errors. The error in the NOIPEA total energies compared to the FCI results decreases with an increasing NOS (see Fig. 3a and a′). Likewise, the IPEA correction becomes larger the larger the NOS is in a state. This can be appreciated explicitly in Fig. 4a and a′, where we show the IPEA correction (calculated as the difference between the total energies obtained from IPEA and NOIPEA calculations) to the total energy of a state as a function of its NOS. On average, the IPEA correction is larger with increasing NOS, as evidence by the positive slope of the linear fit.

The size of the IPEA correction is also represented in Fig. 4b, b′, c and c′ as a function of the size of the system, measured by the number of correlated electrons and the dynamical correlation energy, respectively. The number of correlated electrons in the CASPT2 calculations is the number of electrons of the active spaces in the preceding CASSCF calculations (note that the frozen-core approximation is employed). As one can see in Fig. 4b and b′, the size of the IPEA correction becomes larger with the number of correlated electrons. Similarly, the size of the IPEA correction is also larger for states with larger dynamical correlation energies (Fig. 4c and c′), defined as the energy difference between the CASSCF and FCI energies.

In Table 4, we show that the average IPEA corrections to the CASPT2 total and vertical excitation energies. The former amounts to *ca.* 0.05 eV, regardless of the basis set, and the latter is also positive and very small (*ca.* 0.02–0.03 eV). The IPEA correction is slightly larger for states of molecules with a closed-shell ground state since these molecules have a larger number of excited states that differ in the NOS.

**Table 4:** Average IPEA correction ΔIPEA in eV to the CASPT2 total energies (tot) and vertical excitation energies (exc) for the molecules considered in the FCI benchmark

Basis set	6-31G		6-311G	
Molecules	ΔIPEA (tot)	ΔIPEA (exc)	ΔIPEA (tot)	ΔIPEA (exc)
All^*a*^	0.05	0.02	0.06	0.03
Closed-shell^*b*^	0.05	0.03	0.06	0.03
Open-shell^*c*^	0.05	0.01	0.07	0.02

^*a*^Considering the states of all molecules.

^*b*^Only states belonging to a molecule with a closed-shell ground state.

^*c*^Only states belonging to a molecule with an open-shell ground state.

## 5 Benchmark for excitation energies of organic molecules

**Fig. 5:**
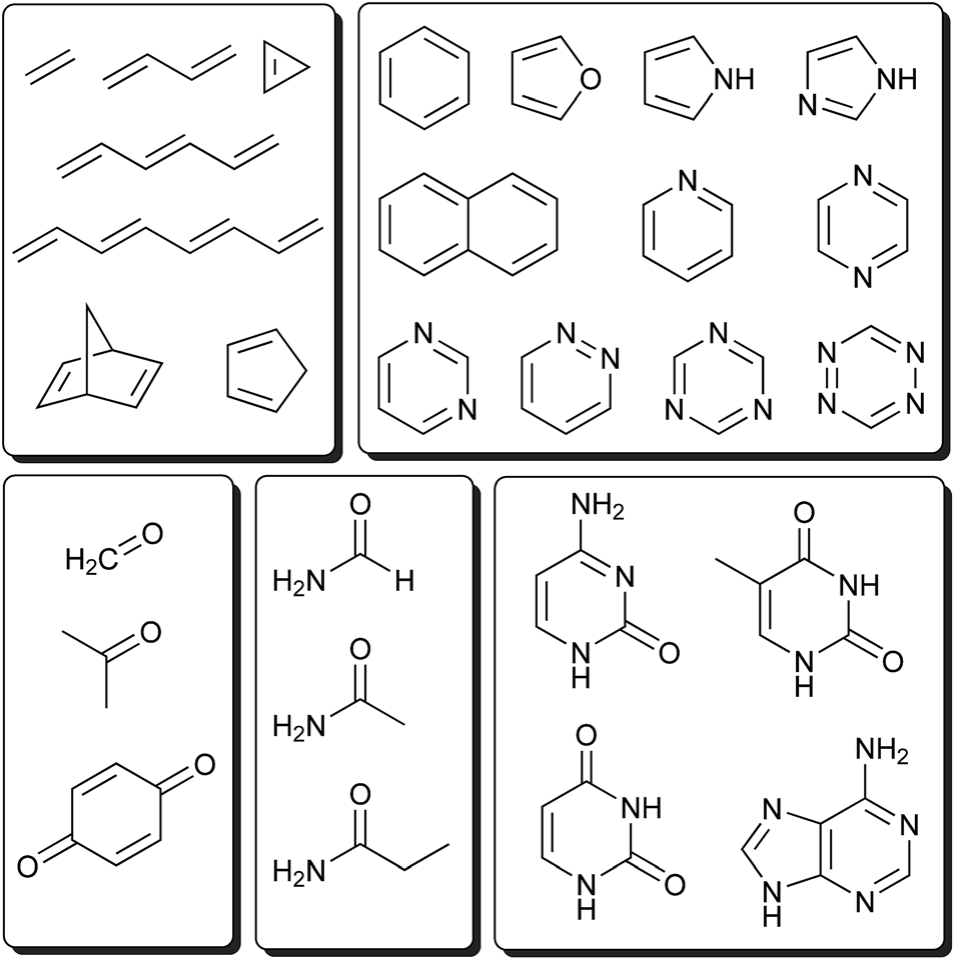
Molecules considered in the Thiel benchmark set.^46^

This section systematically investigates the performance of the IPEA-modified CASPT2 variant on the electronically excited energies of small and medium-sized organic molecules, as contained in the test set of Thiel and coworkers.^46^ This set contains singlet and triplet excited states of 28 important chromophores (see Fig. 5), including unsaturated aliphatic hydrocarbons, aromatic hydrocarbons, aromatic heterocycles, carbonyls, amides, and nucleobases, which were investigated by MS-CASPT2 and several coupled-cluster methods (CC2, CCSD, CC3) with the TZVP basis set. Due to the nature of the TZVP basis set, the study excluded Rydberg states but rather focused on the spectroscopically relevant valence excited states (ππ*, nπ*, and σπ* excited states).

The study of Thiel *et al.* ^46^ was later extended to consider the effect of the larger basis set aug-cc-pVTZ^100,101^ as well as to evaluate the performance of time-dependent density-functional theory (TDDFT) with different functionals (BP86, B3LYP and BHLYP) and the hybrid density-functional theory/multi-reference configuration interaction (DFT/MRCI) approach using the BHLYP functional in the DFT part.^102^ The same test set was also used by Neese and co-workers to calculate excited states with different versions of the second-order *n*-electron valence state perturbation theory (NEVPT2).^103^ NEVPT2 also employs CASSCF wave functions as a reference but, as an extension compared to CASPT2, the zeroth-order Hamiltonian in NEVPT2 also considers two-electron terms. Due to the increased computational demands that come with NEVPT2, for practical implementation a smaller, contracted first-order interaction space is used resulting in the partially-contracted (pc) and strongly-contracted (sc) NEVPT2 schemes.^13^ Since there is no quasi-degenerate NEVPT2 formalism corresponding to the MS-CASPT2 approach, the NEVPT2 calculations of Neese and co-workers are state-specific and were compared to single-state (SS) CASPT2 results. Furthermore, the Thiel set was employed in a recent benchmark by Dreuw and co-workers who performed excited-state calculations using second and third-order algebraic diagrammatic construction (ADC).^104,105^ Of the above available results, we consider here only those that made use of the TZVP basis set.

Our calculations using the IPEA-shifted MS-CASPT2 variant reproduce the excitation energies of nearly all states reported in ref. 46. A complete list of excitation energies is listed in Table S7 of the ESI.† For the sake of consistency, the discussion below is based exclusively on our results.

### 5.1 Computational details

CASPT2 calculations employing both, the NOIPEA and the IPEA-shifted zeroth-order Hamiltonians with the recommended shift value of *ε* = 0.25 a.u. have been performed for the ground and excited states of all the molecules contained in the Thiel set.^46^ The same MP2/6-31G*-optimized geometries, TZVP basis set,^106^ and CASSCF/CASPT2 parameters were used as in the original publication.^46^ These parameters include the number of states considered in each irreducible representation, the use of a level shift for some of the molecules, as well as the employment of the multi-state CASPT2 variant. As in the initial study, for all molecules full point-group symmetry was applied except for benzene and *s*-triazine, where the symmetry was restricted to the *C*_s_ point group. The benchmark study by Thiel and co-workers employed a patch of the MOLCAS6.4 program package.^107^ For this study, we used the newer MOLCAS8.0.15-05-24 version.^99^ The energies of all excited states calculated are presented in Table S10 in the ESI.†

In total, 222 singlet and 87 triplet excited states for the 28 molecules were calculated by Thiel and co-workers. From these, only 170 (174) singlet and 72 (74) triplet excited states were reported in the Supporting Information of ref. 46 and only 149 (153) singlets but all 72 (74) triplet excited states were reported in the main paper (number in parentheses is obtained by double counting the degenerate *E* states of benzene and triazine, for which both components had to be calculated due to the reduced *C*_s_ symmetry). Neese and co-workers report in a footnote^103^ that four singlet states of octatetraene, that were printed in the Supporting Information from ref. 46 but not in the main paper,^46^ were included in the statistical evaluation in the original paper.

We have also calculated 222 singlet and 87 triplet excited states but considered for comparison only the states reported in the ESI in ref. 46, see Table S7 in the ESI.† From the set, we had to exclude a number of states due to intruder state problems in the NOIPEA CASPT2 calculations. To deal with the intruder state problems, we could have employed a larger level shift than the one used in ref. 46, however this would have been at the cost of comparability. Since the number of intruder state problems emerging in NOIPEA CASPT2 was small, the respective states were skipped in our analysis. Further discussion is provided in Section S3.4 in the ESI.†

### 5.2 Size of the IPEA correction

**Fig. 6:**
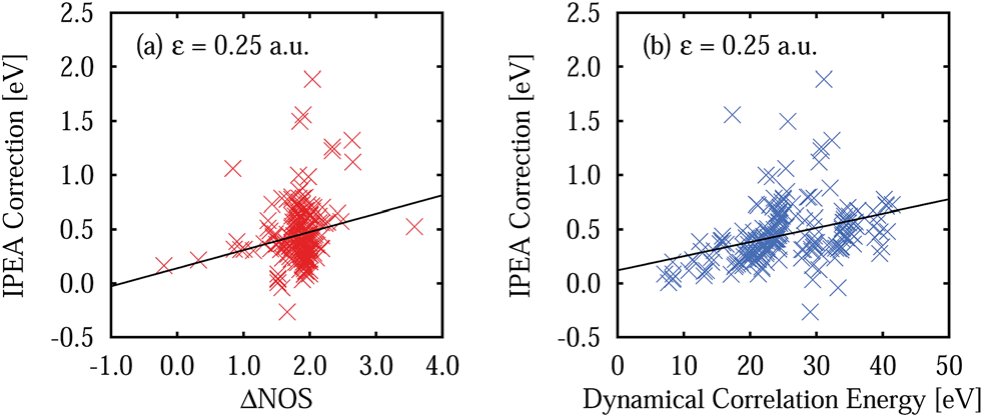
IPEA correction for the vertical excitation energies defined as Δ*V*_IPEA_ = *V*IPEA*i* – *V*NOIPEA*i* for an IPEA shift value of *ε* = 0.25 a.u. (a) IPEA correction as a function of the difference number of open shells ΔNOS. (b) IPEA correction as a function of the dynamical correlation energy ** ^dyn^ = *E*_CASSCF_ – *E*_NOIPEA_. Black lines represent linear fits for both data sets.

A comparison of the calculated excitation energies (see Table S10 in the ESI†) shows that, on average, the use of IPEA with the recommended IPEA shift value (*ε* = 0.25 a.u.) increases the excitation energies by 0.45 eV with respect to the NOIPEA variant. Conspicuously, this increase is much larger than the 0.02 eV found in the di- and triatomic molecules discussed in Section 4. The difference in the vertical excitation energies introduced by the IPEA shift is nearly always positive, except for the 2^1^B_1u_ and 1^3^B_1g_ states of benzoquinone, for which the excitation energies become smaller upon introducing the IPEA parameter. The systematic increase in the excitation energies is due to the consistent increase in the NOS when going from the ground state to the excited state. Fig. 6a shows the IPEA correction as a function of the difference in the NOS between the excited state and its corresponding ground state (ΔNOS). The majority of the excited states are described by a difference of 2 and in this region, the size of the IPEA correction spreads widely with most corrections lying between zero and 1 eV. A small number of states show larger changes in the excitation energy for different ΔNOS. A linear fit indicates that, despite the large spread of the distribution, a dependence of the size of the IPEA correction on ΔNOS can be appreciated.

In Section 4.4, we have observed that the size of the IPEA correction in the di- and triatomic molecules becomes larger with the system size. This was quantified using the dynamical correlation energy *E* ^dyn^ defined as *E*dyn*i* = *E*CASSCF*i* – *E*FCI*i*. Since for Thiel's benchmark set FCI energies are not available we define the dynamical correlation energy **dyn*i* as **dyn*i* = *E*CASSCF*i* – *E*NOIPEA*i*. As an assessment of the quality of ** ^dyn^, we calculated the average ratio 
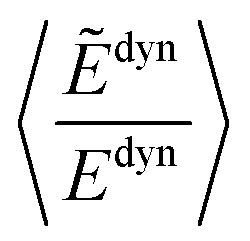
 for the di- and triatomic molecules in Section 4 and found values of 0.88 ± 0.07 (0.88 ± 0.06) for the 6-31G (6-311G) basis set, indicating that *E* ^dyn^ is captured in large part by ** ^dyn^ in standard CASPT2 calculations (see Table S11† for the list of ** ^dyn^).

Fig. 6b displays the IPEA correction for the vertical excitation energies as a function of ** ^dyn^. Clearly, an increase in the dynamical correlation energy increases the size of the IPEA correction, as was found in the di- and triatomic systems. This means that the larger the change in total energy is, which is introduced by CASPT2 with respect to the initial CASSCF calculation, the larger the IPEA correction becomes.

This observation is also in line with a recent study on the ground-state potential energy surface of the chromium dimer which was investigated using CASPT2/RASPT2 employing different active spaces and IPEA shift values.^108^ There, it was observed that with larger active spaces the effect of the IPEA shift on the energies becomes smaller, *i.e.*, when a larger active space is employed in the initial CASSCF action, the energetic changes added by the subsequent CASPT2 treatment are smaller and the effect of the IPEA shift is diminished.

### 5.3 Comparison to experiment

The literature survey of Section 3 evidenced that CASPT2 without IPEA underestimates the experimental excitation energies of organic molecules by less than 0.1 eV. Likewise, the study of Section 4 showed an underestimation of only 0.05 eV compared to FCI excitation energies, which could be decreased to 0.02 eV using the recommended IPEA shift value of *ε* = 0.25 a.u. Since we have just shown that the IPEA correction scales with the size of the system, in large molecules a larger influence of the IPEA shift is to be expected, and we have already seen that the average IPEA correction to the excitation energies becomes as large as 0.45 eV for the states in the Thiel set.

Table 5 shows the mean unsigned and signed errors of the CASPT2 excitation energies (MUEE and MSEE, respectively) of the Thiel benchmark set with respect to experimental reference data. Coincidentally, *ε* = 0 and *ε* = 0.25 a.u. provide the same MUEE of 0.33 eV but the MSEE are different: in the absence of IPEA, CASPT2 underestimates the excitation energies by 0.13 eV but *ε* = 0.25 a.u. overestimates the excitation energies by 0.29 eV. If a different ground state energy is used for reference (MS-CASPT2 ground-state energy for totally symmetric excited states and SS-CASPT2 energy of a separately calculated ground state for the non-totally symmetric excited states, as in ref. 46) slightly smaller MUEEs and MSEEs of 0.29 and 0.24 eV, respectively, are obtained with IPEA *ε* = 0.25 a.u. The latter approach is, however, less consistent as it does not treat all excited states in a similar manner and thus lowers the excitation energy of the non-totally symmetric states artificially (see discussion in Section S3.2 of the ESI†).

**Table 5:** Mean signed error (MSEE) and mean unsigned error (MUEE) for CASPT2 vertical excitation energies *V*calc*i* computed using the TZVP basis set using different IPEA values EPSILON compared to experimental excitation energies *V*exp*i* of the organic molecules from Thiel's benchmark set

*ε* [a.u.]	*N*_States_ ^*a*^	Ground state^*b*^	MUEE^*c*^ [eV]	MSEE^*d*^ [eV]
–0.12	15	MS	0.53	–0.04
0	130	MS	0.33	–0.13
0.08	132	MS	0.27	0.01
0.1337	135	MS	0.25	0.11
0.16	135	MS	0.26	0.15
0.25	137	MS	0.33	0.29
0.25	137	MS/SS	0.29	0.24

^*a*^Different number of states due to intruder state problems (see Section S3.4 in the ESI).

^*b*^Reference energy of ground state for computing excitation energies; MS: only MS-CASPT2 energies used; MS/SS: MS-CASPT2 energy used for totally-symmetric states and SS-CASPT2 energy used for non-totally symmetric states (see Section 5.1).

^*c*^Mean unsigned error of excitation energies computed as 
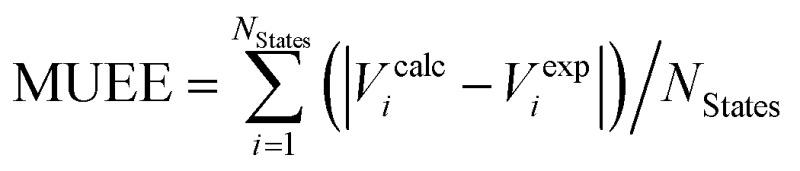
.

^*d*^Mean signed error of excitation energies computed as 
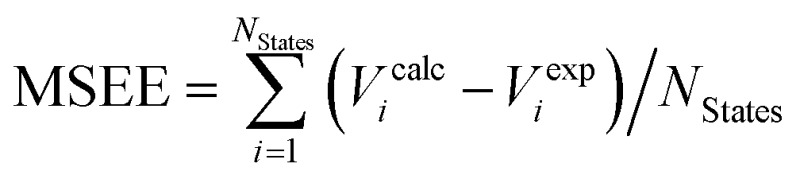
.

**Fig. 7:**
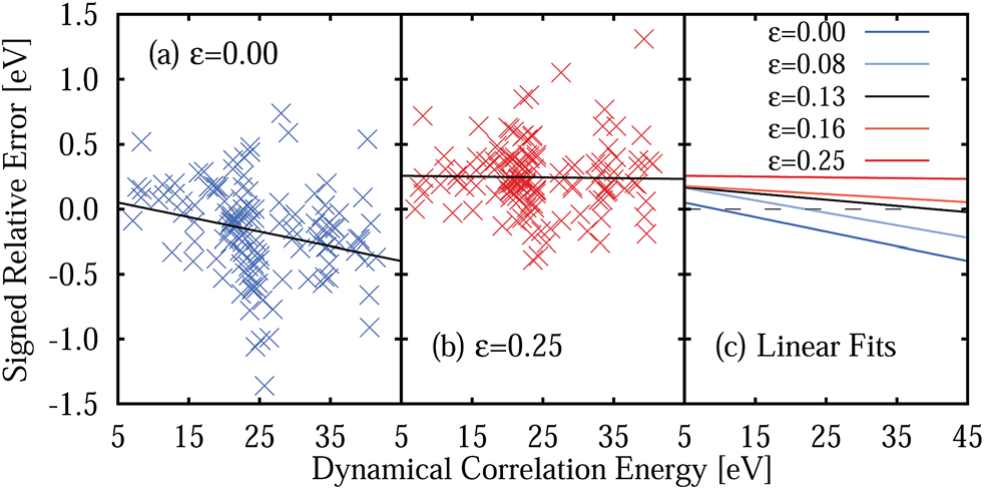
Signed relative error (SRE) of the CASPT2 vertical excitation energies compared to experimental reference data as a function of the dynamical correlation energy ** ^dyn^ for an IPEA shift value of (a) *ε* = 0 and (b) *ε* = 0.25 a.u. (c) Linear fits for the SRE *vs.* ^dyn^ for various IPEA shift values given in a.u. The dashed line represents the constant SRE with a value of 0.

The large MSEE obtained with the *ε* = 0.25 a.u. IPEA shift is due to a consistent overestimation of the energies of the excited states, as 119 of 137 excitation energies present a positive relative error. This is best appreciated in Fig. 7 where the signed relative error (SRE) of all states is plotted as a function of the dynamical correlation energy ** ^dyn^. As can be seen, the average SRE is nearly constant for *ε* = 0.25 a.u. (Fig. 7b) but more equally distributed around SRE = 0 for *ε* = 0 (Fig. 7a), thus leading to a smaller MSE. The linear fits show that the SRE becomes more negative with increasing dynamical correlation energy.

The underestimation of excitation energies for *ε* = 0 and the overestimation for *ε* = 0.25 a.u. may suggest the use of an intermediate IPEA shift value. Accordingly, we have performed calculations for all molecules with example IPEA shift values of *ε* = 0.08, 0.1337, and 0.16 a.u. (see Table 5). It is interesting to note that *ε* = 0.1337 a.u. gives the smallest MUEE and *ε* = 0.08 a.u. gives the smallest MSEE. However, no single IPEA shift value can be favored for the TZVP basis set since the shift leading to the smallest SRE depends on the dynamical correlation of the excited state, as can be seen in Fig. 7c. There, linear fits of the SREs as a function of the dynamical correlation for all IPEA shift values are plotted and, as one can appreciate, the fitted lines cross the SRE = 0 line (dashed line) in a different region of the dynamical correlation energy. For larger IPEA shift values the crossing point is found at a larger value of the dynamical correlation energy.

The dependence of the SRE on the dynamical correlation energy indicates that each type of excited state demands its individual IPEA shift value, what is certainly discouraging for practical applications. A spark of hope appears in the very small slope of the SRE fit for *ε* = 0.25 a.u. The small slope is found as both the average size of the IPEA correction as well as the average SRE of CASPT2, when no IPEA shift is applied, vary in an even manner with the dynamical correlation energy. Thus, the slopes of both functions cancel each other for the largest part and a nearly constant average SRE remains. This behavior would be very promising if it would be a general feature of IPEA-modified CASPT2, as one could just add the remaining error as a correction value and thus completely eliminate the average error of CASPT2 to calculate excitation energies of organic molecules. Alas, such convenient error cancellation is only fortuitous for the TZVP basis set combined with *ε* = 0.25 a.u., as will be demonstrated in the next section.

### 5.4 Basis set effects

#### 5.4.1 Excitation energies

When the IPEA shift technique was introduced,^19^ the ideal shift parameter *ε* was determined by fitting the CASPT2 dissociation energies of diatomic molecules to experimental reference data (recall Section 2.2). Those calculations employed extended ANO-RCC basis sets with minimum contraction. Our conclusions, however, are based on the smaller TZVP basis set. It is therefore important to evaluate how the size of the basis set affects the errors. To this aim, CASPT2 calculations for Thiel's set have been performed with the ANO-RCC basis set^53^ in different sizes (MB, VDZ, VDZP, VTZP, VQZP) and employing different values of the IPEA shift parameter (*ε* = 0, 0.1, 0.2, 0.25, 0.3, 0.4, and 0.5 a.u.). Note the difference between the ANO-RCC-VTZP (from now onwards called VTZP) and the previously used TZVP basis sets. The contraction schemes of the employed basis sets are listed in Table S12 of the ESI.† The obtained excitation energies of all states are reported in Tables S13–S17.†

**Fig. 8:**
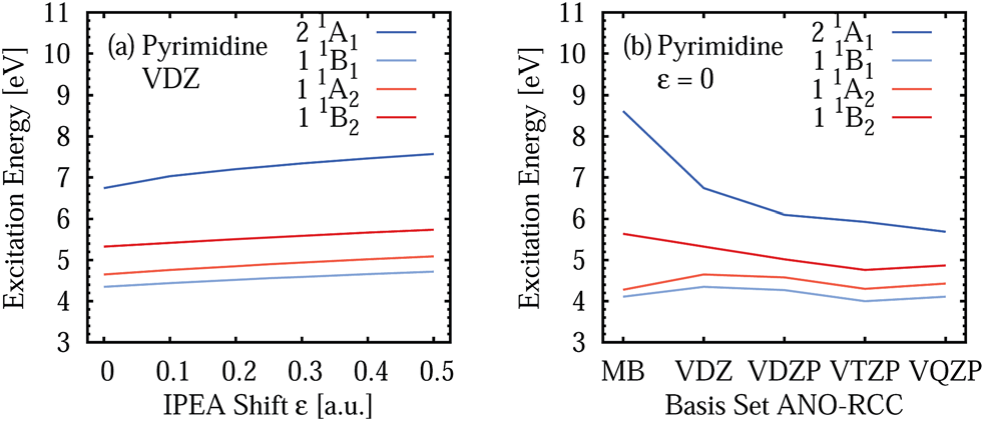
Vertical excitation energies of four excited states of pyrimidine for different IPEA shift values (a) and ANO-RCC basis sets (b).

Two opposite trends can be observed when varying the IPEA shift parameter and the size of the basis set. These are exemplified in the energies of the four excited states of pyrimidine, shown in Fig. 8. The excitation energies increase with increasing value of the IPEA shift but concomitantly decrease when increasing the basis set size. The most drastic changes occur when going from MB to VDZ and VDZP and the changes become smaller when the larger VTZP and VQZP basis sets are reached. This is a general feature, as can be appreciated from Table 6, where we list the average differences Δ*V*_basis_ of all the vertical excitation energies obtained for the different basis sets, for *ε* = 0 (values in the upper triangle) and *ε* = 0.25 a.u. (lower triangle). The differences Δ*V*_basis_ are always positive, indicating that the excitation energies always decrease with larger basis sets. In passing we note that, as expected, the differences are smaller when comparing larger basis sets, indicating convergence of the CASPT2 solution with respect to the size of the basis set; however, since perturbation theory does not satisfy the variational principle, the converged CASPT2 solution does not necessarily need to be closer to the exact solution than that obtained with smaller basis sets. The use of the IPEA shift *ε* = 0.25 a.u. always induces a smaller difference in the excitation energies; as an example, see that when going from MB to VDZ, the excitation energies decrease by 0.69 eV on average for *ε* = 0 *versus* 0.55 eV for *ε* = 0.25 a.u.

**Table 6:** Average differences of the vertical excitation energies Δ*V*_basis_ computed as 
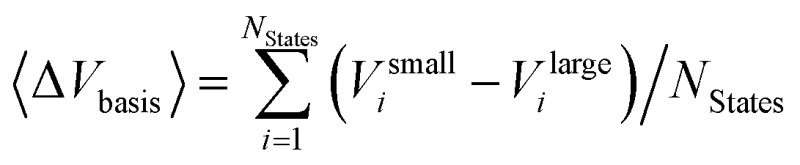
 with different ANO-RCC basis sets in eV. Values above the diagonal (blue) are obtained with *ε* = 0.0 a.u. while entries below the diagonal (red) are obtained with the IPEA shift parameter *ε* = 0.25 a.u.

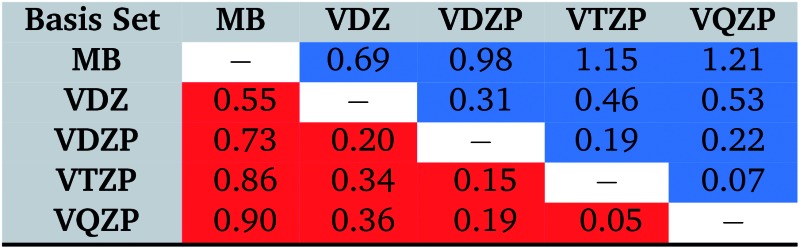

**Fig. 9:**
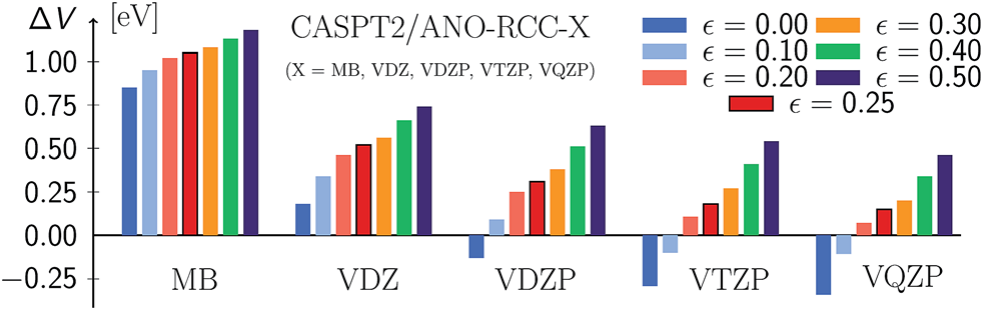
Mean signed errors (MSEE) in eV of CASPT2 vertical excitation energies compared to experimental reference data for different ANO-RCC basis sets and IPEA shift values *ε* (in a.u.).

Fig. 9 (see also Table S23†) displays the MSEE obtained for the different ANO-RCC basis sets for various IPEA shift values *ε* with respect to the experimental results. For the small MB and VDZ basis sets, CASPT2 drastically overestimates the experimental excitation energies regardless of the size of the IPEA shift. This overestimation is systematic in that it is larger for the recommended shift value of *ε* = 0.25 a.u. than for zero and seems to be proportional to the size of the IPEA shift. In contrast, for the larger VDZP, VTZP, and VQZP basis sets, the sign of the MSEE depends on the IPEA shift employed, but neither *ε* = 0 nor the recommended *ε* = 0.25 a.u. yield the smallest MSEE. Instead, better agreement with experimental excitation energies is achieved for intermediate shifts. This analysis clearly demonstrates that the recommended shift value of *ε* = 0.25 a.u. is not appropriate for calculating vertical excitation energies.

**Fig. 10:**
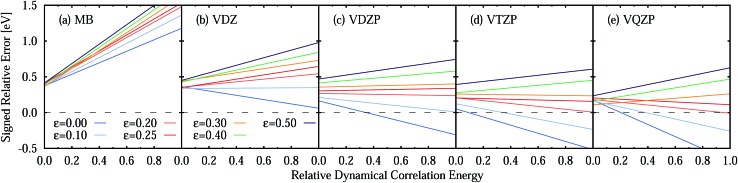
Signed relative error of CASPT2 vertical excitation energies compared to experimental reference data as a function of the basis-set specific relative dynamical correlation **dyndyn of the excited states for different ANO-RCC basis sets and IPEA shift values *ε*. The dashed line represents the constant SRE = 0.

One should remember here that for the TZVP basis set (Section 5.3), the MSEE varies with the dynamical correlation energy ** ^dyn^ of the excited states. This is also the case for any other basis set, as demonstrated in Fig. 10, which shows the SRE as a fitted function of the relative dynamical correlation energy **dynrel for the various basis set/IPEA shift combinations. The relative dynamical correlation energy **dynrel(*i*; *j*) for a state *Ψ*_*i*_ in the basis set {*j*} is given by scaling its total dynamical correlation energy ** ^dyn^(*i*; *j*) with the largest total dynamical correlation energy **dynmax(*i*; *j*) encountered in the set of states {*Ψ*_*p*_} in our benchmark set. This scaling is necessary as the total correlation energy ** ^dyn^(*i*; *j*) of one particular state varies quite considerably with the basis set. For example, for the 2^1^A′ state of adenine computed with *ε* = 0.25 a.u. we find ** ^dyn^(*i*; *j*) of 11.60 (MB), 23.75 (VDZ), 38.21 (VDZP), 47.34 (VTZP), and 50.02 eV (VQZP).

A close look at Fig. 10 shows that for the MB and VDZ basis sets *ε* = 0 yields the smallest error, while for the VDZP basis set, *ε* = 0 gives the smallest SRE only for small values of the relative dynamical correlation energy. For VTZP and VQZP, the smallest SRE for large values of **dynrel is found for *ε* = 0.20 a.u. Fair enough, the SRE for *ε* = 0.25 exhibits only a very small slope in all the excited states of the benchmark set for all the VXZP (X = D, T, Q) basis sets. Nevertheless, it is quite clear that there is no favorite IPEA shift for which errors are consistently small for all basis sets and independent of the dynamical correlation energy.

#### 5.4.2 Dissociation processes

We have shown that the CASPT2 excitation energies of the organic molecules contained in Thiel's benchmark set^46^ decrease with increasing basis set size. The majority of these molecules have a closed-shell electronic ground state and excited states with one pair of open shells. Thus, the decrease in excitation energy indicates a larger stabilization of open-shell electronic states when larger basis sets are used.

In the initial benchmark study by Andersson and Roos,^50^ as well as in the study introducing the IPEA shift technique,^19^ quite large basis sets were employed. Both studies reported unequivocally an underestimation of dissociation energies, which was then related to an underestimation of open-shell electronic states.^19,51^ We have so far shown that this claim is in general not true, at least, for excited states.

Based on the basis set effects shown in Section 5.4.1, one may speculate whether the underestimation of dissociation energies found by Roos and coworkers was due to the large basis sets employed. To investigate this question, we have calculated the ground state potential energy curves of the diatomic molecules considered in Section 4. Calculations were performed with the standard CASPT2 (*ε* = 0) employing different ANO-RCC basis sets and the same active spaces and frozen-core approximation as in Section 4. Total energies along the potential energy curves are reported in Table S6.†

Table 7 collects the well depths *D*_e_ of the ground state potential energy curves calculated as the difference between the energies at the dissociation limit and the energies at the minimum-energy equilibrium geometry, *i.e.*, *D*_e_ = *E*(*R*_inf_) – *E*(*R*_eq_). We report well depths rather than dissociation energies to avoid the calculation of the zero-point energy, since we are only interested in the qualitative behavior of the potential energy curves when varying the basis set size. The values show that the well depths *D*_e_ increase with the basis set size rather than decrease, ruling out that the underestimation of dissociation energies found by Roos and co-workers would be due to the use of basis sets which are too large.

**Table 7:** Well depths *D*_e_ in kcal mol^–1^ of the ground-state potential energy curves of diatomic molecules computed at the CASPT2 level employing different ANO-RCC basis sets for *ε* = 0

Molecule	MB	VDZ	VDZP	VTZP	VQZP
HLi	42.4	42.5	52.7	56.5	57.5
HBe	32.4	33.4	41.7	46.4	47.7
HB	62.6	67.3	78.9	83.3	82.1
HC	51.2	63.9	76.3	80.7	87.5
HN	42.4	58.3	73.0	78.4	81.2
HO	55.3	77.9	97.2	103.4	106.0
HF	77.5	109.5	132.5	139.1	141.7
Li_2_	16.9	17.4	21.4	23.7	23.9
B_2_	51.0	57.2	61.6	65.1	65.7
C_2_	93.4	132.4	134.6	149.9	156.4
N_2_	109.9	165.8	203.8	217.4	222.1

**Fig. 11:**
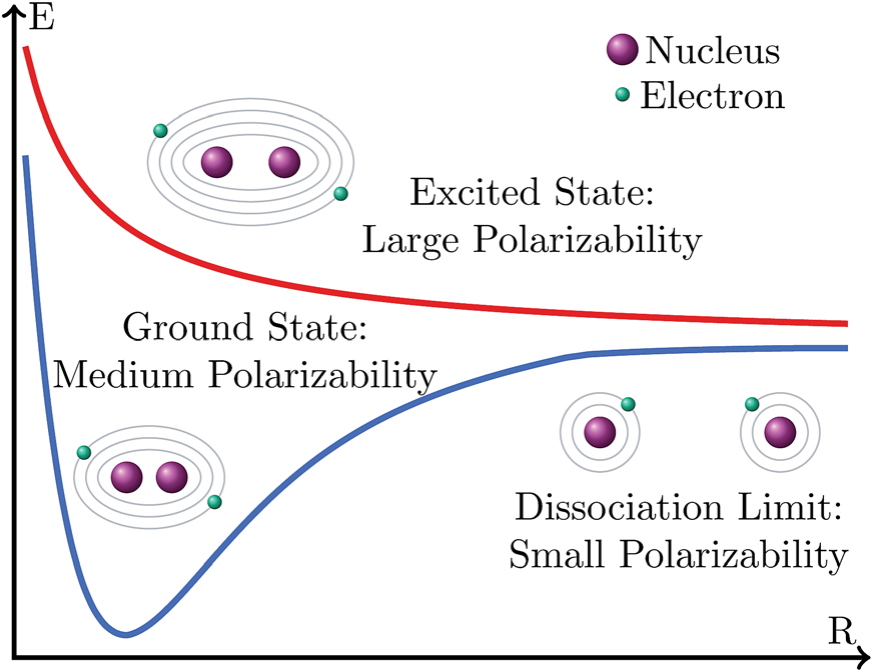
Schematic representation of the polarizability of electronic states at different positions of the potential energy surface. The farther away from the nucleus the electrons are, the larger the polarizability is.

We arrive now at a seemingly peculiar situation when we compare the effect of the basis set size on the variation of excitation and dissociation energies. Both energies are differences between the energy of an electronic state that is, rather described by a closed-shell electronic configuration (the ground state at the equilibrium geometry) and the energy of an electronic state that possesses a rather open-shell character (the excited state at the Franck–Condon geometry or the ground state at the dissociation limit). However, while the excitation energy becomes smaller with the increase in the basis set size, the dissociation energy becomes larger. This behavior could be explained in the following manner, as depicted in Fig. 11. Situations where electrons have large polarizabilities demand large basis sets; in contrast, when the polarizability is small, a small basis is enough to provide a good description. In general, the polarizability is larger when the electrons are more weakly bound to the atomic nucleus. Thus, at the dissociation limit the fragments possess smaller polarizability than the molecule at the equilibrium geometry because the number of inner electrons in the fragments is smaller and thus the valence electrons are more bound to the nuclei. Accordingly, an increase in the basis set size should stabilize the molecule at the equilibrium geometry more than the molecule at the dissociation limit, leading to an increase in the dissociation energy. In contrast, within an electronic excitation, electrons in the excited state have larger polarizability than those in the ground state because they are less bound to the nucleus. Accordingly, the increase in the basis set stabilizes the excited state more than the ground state leading to a decrease in the excitation energy.

### 5.5 Epilogue: comparison to other methods

As we have shown, the use of the IPEA-modified CASPT2 variant to compute excitation energies is an intricate problem. If we compare CASPT2 results with experimental excitation energies, the optimal shift value that minimizes the error depends on the dynamical correlation energy. Moreover, we have seen a dependency on the size of the basis set, a problem which is aggravated with the fact that perturbation theory is non-variational, and thus increasing the basis set size does not necessarily yield better energies. Both options, not using the IPEA shift or using the recommended IPEA value of *ε* = 0.25 a.u., which was deduced from errors in dissociation energies, seems to be rather unsatisfactory. In this situation, one could finally wonder, how adequate CASPT2 is in comparison with other methods. Therefore, in this section, we compare the accuracy of these two opposed strategies to other *ab initio* methods^46,102,103,105^ employed on Thiel's benchmark set.

**Fig. 12:**
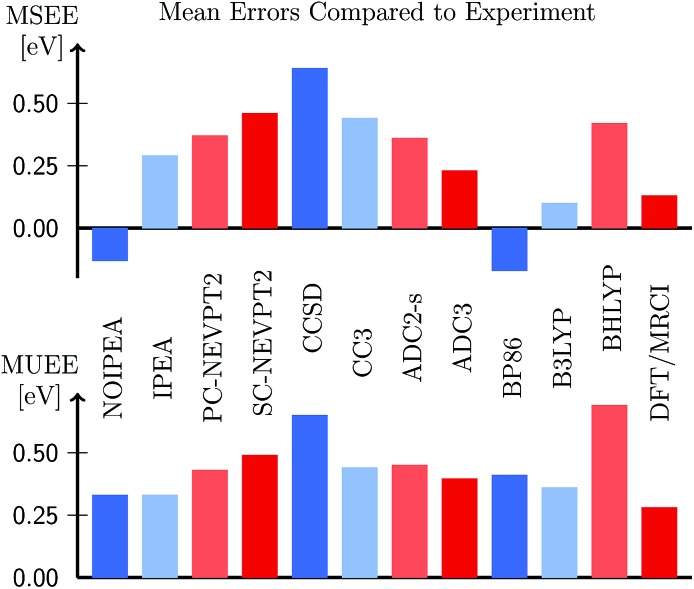
Mean signed error (MSEE) and mean unsigned error (MUEE) in eV in the vertical excitation energies of the organic molecules from Thiel's benchmark set, computed at different levels of theory (this work and ref. 46, 102, 103 and 105) using the TZVP basis set.

The MSEE and MUEE for all levels of theory considered are compiled in Fig. 12 for the TZVP basis set (see also Table S25); the list of all excitation energies can be found in Table S24.† Reassuringly, we can observe that from all the wave function based methods, CASPT2, with (*ε* = 0.25 a.u.) and without the IPEA shift (*ε* = 0), yield the smallest errors. Both NEVPT2 formulations, the two coupled-cluster variants, as well as the two ADC variants overestimate the experimental excitation energies. From these methods, ADC(3) possesses the smallest MSEE, which is of the same size as the MSEE of IPEA CASPT2. As far as TD-DFT is concerned, not surprisingly, the error depends on the functional: the GGA functional BP86 underestimates the excitation energies, while the hybrid B3LYP and BHLYP functionals overestimate them. The MSEE of BP86 and B3LYP are quite small and on the same scale as the MSEE for the standard NOIPEA CASPT2 variant. The MSEE of B3LYP is even smaller in magnitude than for standard CASPT2 amounting only to 0.10 eV, however, at the cost of a slightly larger MUEE indicating wider spread of the errors. An excellent performance is also displayed by the DFT/MRCI approach, with an MSEE of only 0.13 eV and the smallest MUEE of all the methods (0.28 eV).

## 6 Concluding remarks

This paper presents compelling evidence that the IPEA shift technique widely employed in the CASPT2 method is not necessary to calculate electronically excited states of organic chromophores. This technique was introduced to correct an underestimation in the energies of open-shell states that was observed in the calculation of dissociation energies. However, from a large collection of excited states of small and medium-sized organic molecules available in the literature, we found that the actual underestimation in the excited state energies is minimal (0.02 eV), and thus an order of magnitude smaller than it was anticipated^19,50^ based on errors reported for dissociation energies (0.13–0.26 eV times the difference between the number of paired electrons within the molecule and within the atoms).

We then performed full CI benchmark calculations on a series of di- and triatomic molecules to compare the results against CASPT2 with and without the recommended IPEA shift value of 0.25 a.u. Without IPEA an error of –0.05 eV is found, which is again considerably smaller than the expected error. Using the IPEA variant, the underestimation of the CASPT2 vertical excitation energies decreases only to –0.03 eV. Since the error in CASPT2 was supposed to scale with the difference in the number of open shells, the IPEA correction should also scale with the number of open shells. On average, we find that the IPEA correction does increase when the number of open shells increases but for the individual states with a common number of open shells, the size of the IPEA correction exhibits a noticeable spread. This is due to the fact that the IPEA correction also scales with the size of the system, here measured in terms of the amount of dynamical correlation energy.

The observation that the energy correction introduced by the IPEA shift scales with the size of the system led us to the assumption that the changes in vertical excitation energies introduced by the IPEA shift will increase for larger systems, eventually leading to an overestimation of vertical excitation energies. To investigate this possibility, we carried out a systematic calculation of the excited states of small and medium-sized chromophores contained in the benchmark set of Thiel and coworkers.^46^ The inclusion of the IPEA shift increases the excitation energies by 0.45 eV – an increase which specifically depends on the system size (or the amount of dynamical correlation energy). The excitation energies without IPEA are underestimated by 0.13 eV with respect to experimental values, while those including IPEA are overestimated by 0.24 eV, using the TZVP basis set. We find, therefore, that on average not using IPEA gives a better agreement with the experiment.

The errors involved in the CASPT2 excitation energies depend on the amount of dynamical correlation energy. Despite the fact that it is possible to find a correlation between the size of the IPEA correction and the dynamical correlation energy, this relationship is different for different basis sets. This indicates that an ideal IPEA shift that minimizes the average error of CASPT2 depends on both the system size and the basis set – very much against the spirit of an *ab initio* or parameter-free method. The use of basis sets of double-*ζ* quality leads to the smallest errors by setting the IPEA shift parameter to zero. For larger basis sets of triple- or quadruple-*ζ* quality, better agreement with the experiment is found for IPEA shift values which are different from zero, in particular for values of IPEA below 0.25 a.u. From a pragmatic point of view, it seems that just neglecting the IPEA shift and using CASPT2 with double-*ζ* basis sets can be the most convenient approach in standard excited state calculations. The slight underestimation involved is tolerable as it lies (on average) below the commonly accepted accuracy of the CASPT2 method. Although the small size of the error is simply due to error cancellation, the same holds true when larger basis sets and a nonzero IPEA shift are used. The latter approach, however, does not substantially improve the energies but comes with a higher computational price.

In general, therefore, the use of the IPEA shift in the CASPT2 calculation of electronic excited states of organic chromophores is not justified and a universal shift parameter valid for any basis set or system size cannot be optimized. The good news is that without the IPEA correction, CASPT2 still yields one of the best agreements with experimental data within the family of *ab initio* methods, at least for organic chromophores, such as those included in Thiel's molecular benchmark set. Thus, twenty five years after its introduction, CASPT2 still remains an excellent choice for investigating excited states.

Future work could focus on investigating the effect of the IPEA shift parameter on transition metal complexes. A number of studies, mostly concerned with the electronic properties (high-spin low-spin gaps) of six-coordinate/octahedral hexaaza iron(ii) complexes and similar compounds, indicate that in such cases the IPEA shift is important. In contrast to the present work about organic molecules, some of these studies advocate for maintaining the recommended value of *ε* = 0.25 a.u.,^109–112^ or even use larger IPEA shift values.^54,113–115^ One could speculate whether these recommendations are due to intricate electronic structure related to metals, or due to the typically large basis sets employed for transition metal complexes. In fact, for excitation energies of organic molecules we have found that when larger basis sets are used, a larger IPEA shift is required to profit from better error cancellation. Clearly, it will be interesting to investigate to what extent the trends found here can be extrapolated to other type of systems.
