# Supplementary material for: The IPEA dilemma in CASPT2
Source: Chem Sci. 2016 Sep 26;8(2):1482–99. doi: 10.1039/c6sc03759c (PMC5452265; doi:10.1039/c6sc03759c)
Supplement: Supplementary file 1 [file SC-008-C6SC03759C-s001.pdf]

Electronic Supplementary Material (ESI) for Chemical Science.
This journal is © The Royal Society of Chemistry 2016

The IPEA Dilemma in CASPT2

J. Patrick Zobel, Juan J. Nogueira,* and Leticia González*

Institute of Theoretical Chemistry, Faculty of Chemistry, University of Vienna, Währinger Straße 17, A-1090 Vienna, Austria

E-mail: nogueira.perez.juanjose@univie.ac.at; leticia.gonzalez@univie.ac.at

Contents

S1 Excitation Energies from the Literature Survey **S4**

S2 CASPT2/CASSCF vs. FCI Benchmark **S20**

S2.1 Computational Details S20

S2.2 Closed-Shell vs. Open-Shell Characterization S21

S2.3 Ground State Geometries of Di- and Triatomic Molecules S23

S2.4 Excited State Energies of Di- and Triatomic Molecules S24

S2.5 The 2 $^1\Sigma^+$ State of NH S38

S2.6 Potential Energy Curves of Diatomic Molecules S38

S3 Thiel's Benchmark Set **S48**

S3.1 Comments of the Benchmark Calculations of Thiel S48

S3.2 Ground State Energies of all Molecules S56

S3.3 Additional Computational Details S57

S3.4 Intruder State Problems S57

S3.4.1 General Considerations S57

*To whom correspondence should be addressed

S3.4.2 Intruder State Problems in NOIPEA-CASPT2 Calculations S59
S3.4.3 Intruder State Problems in IPEA-CASPT2 Calculations S64
S3.5 CASPT2 Results for Thiel's Benchmark Set S65
S3.6 Basis Set Effects S80
S3.7 Comparison to other Computational Results S119

List of Tables

S1 Excitation energies of organic molecules considered in the literature survey . . S5

S2 Correspondence of irreducible representations. S21

S3 Cartesian coordinates of di- and triatomic molecules. S23

S4 Energies of di- and triatomic molecules for the 6-31G basis set. S26

S5 Energies of di- and triatomic molecules for the 6-311G basis set. S32

S6 Potential energy curves of diatomic molecules. S38

S7 Comparison of CASPT2 excitation energies obtained by Thiel and this work. . S49

S8 CASPT2 ground-state energies for molecules in the Thiel benchmark set. . . . S56

S9 List of states excluded due to intruder state problems. S64

S10 CASPT2/TZVP and experimental excitation energies for Thiel's benchmark set. S65

S11 NOS and $\widetilde{E}^{\mathrm{dyn}}$ for the electronic states in Thiel's benchmark set. S73

S12 Size of contracted basis sets for hydrogen and second-row atoms. S80

S13 CASPT2/MB vertical excitation energies for Thiel's benchmark set. S81

S14 CASPT2/VDZ vertical excitation energies for Thiel's benchmark set. S85

S15 CASPT2/VDZP vertical excitation energies for Thiel's benchmark set. S89

S16 CASPT2/VTZP vertical excitation energies for Thiel's benchmark set. S93

S17 CASPT2/VQZP vertical excitation energies for Thiel's benchmark set. S96

S18 CASPT2/MB dynamical correlation energies for Thiel's benchmark set. S100

S19 CASPT2/VDZ dynamical correlation energies for Thiel's benchmark set. S104

S20 CASPT2/VDZP dynamical correlation energies for Thiel's benchmark set. . . . S108

S21 CASPT2/VTZP dynamical correlation energies for Thiel's benchmark set. . . . S112

S22 CASPT2/VQZP dynamical correlation energies for Thiel's benchmark set. . . . S116

S23 MSEE for different ANO-RCC basis sets and IPEA shift values for Thiel's benchmark set S120

S24 Excitation energies at different levels of theory for Thiel's benchmark set. . . . S121

S25 MSEE and MUEE for different levels of theory for Thiel's benchmark set . . . S131

Electronic Supplementary Information

This document is structured as follows. Section S1 collects all data related to the literature survey conducted in section 3 of the main manuscript and explains the procedure we followed to select the publications. In section S2, we provide additional computational details and data related to the CASSCF/CASPT2 and FCI calculations for the di- and triatomic molecules, described in section 4 of the main manuscript. And finally, section S3 presents the computational details, data, and additional discussion related to the CASPT2 calculations on the Thiel's benchmark set, described in section 5 of the main manuscript.

S1 Excitation Energies from the Literature Survey

The excitation energies considered in our analysis are collected in Table S1. The publications that entered our analysis were selected in the following manner. A simple search for the topic "CASPT2" in the SciFinder database generates over 2000 hits. Out of these 2000 hits, 770 refer to papers published until 2004. From those, we selected only studies concerned with the calculation of vertical excitation energies based on their title, i.e., we discarded adiabatic excitation energies or comparison of full potential energy surfaces. Further, we eliminated all studies where no experimental data was given for comparison. In case where several computational studies were provided, we included only the results obtained using the largest active space and/or basis set. Likewise, when multiple experimental values for the excitation energies were reported in the computational studies, we used the average of all of them as the reference data with one exception: since most computational studies were performed in the gas phase, we neglected experimental reference data obtained in solution if experimental reference data in gas phase was available.

For the organic molecules, we differentiated between valence and Rydberg excited states, excited states calculated in gas phase or any other environment, and excited states calculated with the standard non-diagonal CASPT2 variant[1] or any other CASPT2 variant.

Table S1: Calculated (V^{calc}) and experimental(V^{exp}) excitation energies and energy differences (ΔV) in eV.

**Molecule**	**State**	**V^{calc}**	**V^{exp}**	**ΔV**	**Character**	**Environment**	**Method**	**Ref.**
Pyridazine	$^1A_1$	4.86	4.45	0.41	valence	gas phase	any	2
Pyridazine	$^1B_2$	6.61	6.35	0.26	valence	gas phase	any	2
Pyridazine	$^1B_2$	7.39	7.2	0.19	valence	gas phase	any	2
Pyridazine	$^1A_1$	7.50	7.2	0.30	valence	gas phase	any	2
Pyridazine	$^1B_1$	3.48	3.4	0.08	valence	gas phase	any	2
Pyridazine	$^1A_2$	5.09	5.75	−0.66	valence	gas phase	any	2
Pyridazine	$^1B_1$	5.80	5.75	0.05	valence	gas phase	any	2
s-Triazine	$^1A_2'$	5.33	5.7	−0.37	valence	gas phase	any	2
s-Triazine	$^1A_1'$	6.77	6.86	−0.09	valence	gas phase	any	2
s-Triazine	$^1E'$	8.16	7.76	0.40	valence	gas phase	any	2
s-Triazine	$^1A_2''$	4.00	4.59	−0.59	valence	gas phase	any	2
s-Triazine	$^1E''$	4.24	3.97	0.27	valence	gas phase	any	2
s-Triazine	$^1E''$	7.13	6.15	0.98	valence	gas phase	any	2
Cyclopentadiene	1^1B_2	5.27	5.26	0.01	valence	gas phase	standard	3
Cyclopentadiene	1^1A_2	5.65	5.68	−0.03	Rydberg	gas phase	standard	3
Cyclopentadiene	1^1B_1	6.24	6.25	−0.01	Rydberg	gas phase	standard	3
Cyclopentadiene	2^1B_2	6.25	6.31	−0.06	Rydberg	gas phase	standard	3
Cyclopentadiene	2^1A_2	6.30	6.26	0.04	Rydberg	gas phase	standard	3
Cyclopentadiene	2^1A_1	6.31	6.2	0.11	valence	gas phase	standard	3
Cyclopentadiene	3^1B_2	6.86	6.8	0.06	Rydberg	gas phase	standard	3
Cyclopentadiene	3^1A_1	6.93	7.05	−0.12	Rydberg	gas phase	standard	3
Cyclopentadiene	4^1A_1	7.89	7.95	−0.06	valence	gas phase	standard	3
Cyclopentadiene	3^1B_1	7.95	8.03	−0.08	Rydberg	gas phase	standard	3
Cyclopentadiene	1^3B_2	3.15	3.1	0.05	valence	gas phase	standard	3
Cyclopentadiene	1^3A_1	4.90	4.7	0.20	valence	gas phase	standard	3

Table S1: . . . continued

**Molecule**	**State**	$\mathbf{V}^{calc}$	$\mathbf{V}^{exp}$	$\boldsymbol{\Delta V}$	**Character**	**Environment**	**Method**	**Ref.**
Pyrrole	3^1B_1	6.40	6.43	−0.03	Rydberg	gas phase	standard	3
Pyrrole	3^1B_2	6.53	6.5	0.03	Rydberg	gas phase	standard	3
Pyrrole	3^1A_1	6.54	6.5	0.04	Rydberg	gas phase	standard	3
Pyrrole	4^1B_1	6.62	6.78	−0.16	Rydberg	gas phase	standard	3
Pyrrole	4^1A_1	6.65	6.78	−0.13	Rydberg	gas phase	standard	3
Pyrrole	5^1B_1	7.32	7.26	0.06	Rydberg	gas phase	standard	3
Pyrrole	5^1A_1	7.36	7.43	−0.07	Rydberg	gas phase	standard	3
Pyrrole	6^1B_1	7.39	7.43	−0.04	Rydberg	gas phase	standard	3
Pyrrole	4^1B_2	7.43	7.43	0.00	Rydberg	gas phase	standard	3
Pyrrole	6^1A_1	7.46	7.54	−0.08	valence	gas phase	standard	3
Pyrrole	5^1B_2	7.72	7.4	0.32	Rydberg	gas phase	standard	3
Pyrrole	1^3B_2	4.27	4.21	0.06	valence	gas phase	standard	3
Pyrrole	1^3A_1	5.16	5.1	0.06	valence	gas phase	standard	3
Furan	1^1A_2	5.92	5.94	−0.02	Rydberg	gas phase	standard	3
Furan	1^1B_2	6.04	6.06	−0.02	valence	gas phase	standard	3
Furan	1^1B_1	6.46	6.48	−0.02	Rydberg	gas phase	standard	3
Furan	2^1B_2	6.48	6.48	0.00	Rydberg	gas phase	standard	3
Furan	2^1A_2	6.59	6.61	−0.02	Rydberg	gas phase	standard	3
Furan	3^1A_1	7.31	7.28	0.03	Rydberg	gas phase	standard	3
Furan	3^1B_1	7.21	7.38	−0.17	Rydberg	gas phase	standard	3
Furan	4^1A_1	7.74	7.82	−0.08	valence	gas phase	standard	3
Furan	1^3B_2	3.99	4.02	−0.03	valence	gas phase	standard	3
Furan	1^3A_1	5.15	5.22	−0.07	valence	gas phase	standard	3
Octatetraene	1^1B_u	4.42	4.41	0.01	valence	gas phase	standard	4
Octatetraene	2^1B_u	5.70	5.69	0.01	Rydberg	gas phase	standard	4

Table S1: … continued

**Molecule**	**State**	$\mathbf{V}^{calc}$	$\mathbf{V}^{exp}$	$\mathbf{\Delta}V$	**Character**	**Environment**	**Method**	**Ref.**
Octatetraene	3^1B_u	5.83	5.88	−0.05	valence	gas phase	standard	4
Octatetraene	3^1A_g	6.10	6.04	0.06	Rydberg	gas phase	standard	4
Octatetraene	1^3B_u	2.17	2.1	0.07	valence	gas phase	standard	4
Octatetraene	1^3A_g	3.39	3.55	−0.16	valence	gas phase	standard	4
Naphthalene	1^1B_{3u}	4.03	3.99	0.05	valence	gas phase	standard	5
Naphthalene	1^1B_{2u}	4.56	4.58	−0.01	valence	gas phase	standard	5
Naphthalene	2^1A_g	5.39	5.51	−0.12	valence	gas phase	standard	5
Naphthalene	1^1B_{1g}	5.53	5.25	0.28	valence	gas phase	standard	5
Naphthalene	2^1B_{3u}	5.54	5.69	−0.15	valence	gas phase	standard	5
Naphthalene	1^1A_u	5.54	5.6	−0.06	Rydberg	gas phase	standard	5
Naphthalene	2^1B_{1g}	5.87	5.8	0.07	valence	gas phase	standard	5
Naphthalene	2^1B_{2u}	5.93	6.07	−0.14	valence	gas phase	standard	5
Naphthalene	3^1A_g	6.04	6.03	0.01	valence	gas phase	standard	5
Naphthalene	4^1B_{2u}	7.16	11.35	−4.19	valence	gas phase	standard	5
Pyrazine	1^1B_{2u}	4.75	4.81	−0.06	valence	gas phase	standard	6
Pyrazine	2^1B_{2u}	7.70	7.67	0.03	valence	gas phase	standard	6
Pyrazine	1^1B_{1u}	6.70	6.51	0.19	valence	gas phase	standard	6
Pyrazine	2^1B_{1u}	7.57	7.67	−0.10	valence	gas phase	standard	6
Pyrazine	1^1B_{3u}	3.63	3.83	−0.20	valence	gas phase	standard	6
Norbornadiene	1^1A_2	5.28	5.24	0.04	valence	gas phase	standard	7
Norbornadiene	1^1B_1	5.73	5.85	−0.12	Rydberg	gas phase	standard	7
Norbornadiene	1^1B_2	6.20	5.94	0.26	valence	gas phase	standard	7
Norbornadiene	2^1A_1	6.33	6.37	−0.04	Rydberg	gas phase	standard	7
Norbornadiene	2^1B_2	6.48	6.68	−0.20	valence	gas phase	standard	7
Norbornadiene	3^1B_2	6.50	6.6	−0.10	Rydberg	gas phase	standard	7

Table S1: . . . continued

**Molecule**	**State**	$\mathbf{V}^{calc}$	$\mathbf{V}^{exp}$	$\mathbf{\Delta}V$	**Character**	**Environment**	**Method**	**Ref.**
Norbornadiene	3^1A_1	6.67	6.87	−0.20	Rydberg	gas phase	standard	7
Norbornadiene	4^1A_1	6.94	7.2	−0.26	Rydberg	gas phase	standard	7
Norbornadiene	5^1A_2	7.36	7.5	−0.14	valence	gas phase	standard	7
Norbornadiene	6^1B_1	7.39	7.65	−0.26	Rydberg	gas phase	standard	7
Norbornadiene	7^1A_1	7.74	7.9	−0.16	Rydberg	gas phase	standard	7
Norbornadiene	8^1A_1	7.84	8	−0.16	Rydberg	gas phase	standard	7
Norbornadiene	1^3A_2	3.42	3.44	−0.02	valence	gas phase	standard	7
Norbornadiene	1^3B_2	3.80	3.9	−0.10	valence	gas phase	standard	7
Benzene	1^1B_{2u}	4.84	4.9	−0.06	valence	gas phase	standard	8
Benzene	1^1B_{1u}	6.30	6.2	0.10	valence	gas phase	standard	8
Benzene	1^1E_{1u}	7.03	6.98	0.05	valence	gas phase	standard	8
Benzene	2^1E_{2g}	7.90	7.8	0.10	valence	gas phase	standard	8
Benzene	2^1E_{1u}	7.16	7.41	−0.25	Rydberg	gas phase	standard	8
Benzene	2^1A_{1g}	7.74	7.81	−0.07	Rydberg	gas phase	standard	8
Benzene	1^1E_{2g}	7.77	7.81	−0.04	Rydberg	gas phase	standard	8
Benzene	1^1A_{2g}	7.81	7.81	0.00	Rydberg	gas phase	standard	8
Benzene	1^1E_{1g}	6.38	6.33	0.05	Rydberg	gas phase	standard	8
Benzene	1^1A_{2u}	6.86	6.93	−0.07	Rydberg	gas phase	standard	8
Benzene	1^1E_{2u}	6.91	6.95	−0.04	Rydberg	gas phase	standard	8
Benzene	1^1B_{2g}	7.58	7.46	0.12	Rydberg	gas phase	standard	8
Benzene	1^1B_{1g}	7.58	7.46	0.12	Rydberg	gas phase	standard	8
Benzene	2^1E_{1g}	7.57	7.53	0.04	Rydberg	gas phase	standard	8
Benzene	1^3B_{1u}	3.89	3.94	−0.05	Rydberg	gas phase	standard	8
Benzene	1^3E_{1u}	4.49	4.76	−0.27	Rydberg	gas phase	standard	8
Benzene	1^eB_{2u}	5.49	5.6	−0.11	Rydberg	gas phase	standard	8

Table S1: … continued

Molecule	State	$\mathbf{V}^{calc}$	$\mathbf{V}^{exp}$	$\boldsymbol{\Delta}V$	Character	Environment	Method	Ref.
Benzene	1^3E_{2g}	7.12	7.49	−0.37	Rydberg	gas phase	standard	8
Phenol	$^1B_{2u}$	4.53	4.51	0.02	valence	gas phase	standard	8
Phenol	$^1V_{1z}$	5.80	5.77	0.03	valence	gas phase	standard	8
Phenol	$^1E_{1u}$	6.50	6.66	−0.16	valence	gas phase	standard	8
Phenol	$^1E_{1u}$	6.56	6.66	−0.10	valence	gas phase	standard	8
Ozone	$^1A_2$	1.77	1.6	0.17	valence	gas phase	standard	9
Ozone	$^1B_1$	1.87	2.1	−0.23	valence	gas phase	standard	9
Ozone	$^1B_2$	4.51	4.9	−0.39	valence	gas phase	standard	9
Formaldehyde	1^1A_2	3.91	3.93	−0.02	valence	gas phase	standard	10
Formaldehyde	1^1B_2	7.30	7.09	0.21	Rydberg	gas phase	standard	10
Formaldehyde	2^1B_2	8.09	8.14	−0.05	Rydberg	gas phase	standard	10
Formaldehyde	2^1A_1	8.12	7.97	0.15	Rydberg	gas phase	standard	10
Formaldehyde	2^1A_2	8.32	8.37	−0.05	Rydberg	gas phase	standard	10
Formaldehyde	3^1B_2	9.13	8.88	0.25	Rydberg	gas phase	standard	10
Formaldehyde	3^1A_2	9.31	9.22	0.09	Rydberg	gas phase	standard	10
Formaldehyde	1^3A_2	3.48	3.5	−0.02	valence	gas phase	standard	10
Formaldehyde	1^3A_1	5.99	5.82	0.17	valence	gas phase	standard	10
N_2	$^3\Sigma_u^+$	5.90	6.22	−0.32	valence	gas phase	standard	11
N_2	$^3\Pi_g$	7.07	7.39	−0.32	valence	gas phase	standard	11
Formamide	$1^1A''$	5.61	5.5	0.11	valence	gas phase	any	12
Formamide	$2^1A'$	7.41	7.4	0.01	valence	gas phase	any	12
Formamide	$1^3A''$	5.34	5.3	0.04	valence	gas phase	any	12
Glycine	$1^1A''$	5.65	5.9	−0.25	valence	any	any	13
Glycine	$3^1A'$	6.98	6.95	0.03	Rydberg	any	any	13
Glycine	$11^1A'$	8.10	8.1	0.00	valence	any	any	13

Table S1: . . . continued

Molecule	State	$\mathbf{V}^{calc}$	$\mathbf{V}^{exp}$	$\boldsymbol{\Delta}V$	Character	Environment	Method	Ref.
Glycine	$15^1A'$	8.86	8.5	0.36	Rydberg	any	any	13
Glycine	$16^1A'$	8.99	8	0.99	Rydberg	any	any	13
Glycine	$19^1A'$	10.21	10	0.21	valence	any	any	13
N-acetylglycine	$1^1A''$	5.25	5.6	−0.35	valence	any	any	13
N-acetylglycine	$2^1A''$	5.63	5.9	−0.27	valence	any	any	13
N-acetylglycine	$2^1A'$	6.70	6.5	0.20	Rydberg	any	any	13
N-acetylglycine	$3^1A'$	6.76	6.6	0.16	valence	any	any	13
N-acetylglycine	$7^1A'$	7.14	6.9	0.24	Rydberg	any	any	13
N-acetylglycine	$11^1A'$	8.55	8	0.55	valence	any	any	13
N-acetylglycine	$13^1A'$	9.51	9.1	0.41	valence	any	any	13
Imidazole	$2^1A'$	6.32	6	0.32	valence	gas phase	any	14
Imidazole	$3^1A'$	6.53	6.5	0.03	valence	gas phase	any	14
Indole	$2^1A'$	4.43	4.37	0.06	valence	gas phase	standard	15
Indole	$3^1A'$	4.73	4.77	−0.04	valence	gas phase	standard	15
Indole	$4^1A'$	5.21	5.27	−0.06	Rydberg	gas phase	standard	15
Indole	$5^1A'$	5.65	5.55	0.10	Rydberg	gas phase	standard	15
Indole	$6^1A'$	5.84	6.02	−0.18	valence	gas phase	standard	15
Indole	$12^1A'$	6.35	6.35	0.00	valence	gas phase	standard	15
Biphenylene	1^1B_{1u}	3.31	3.55	−0.24	valence	gas phase	any	16
Biphenylene	2^1A_g	4.49	4.59	−0.10	valence	any	any	16
Biphenylene	2^1B_{1u}	4.85	5.1	−0.25	valence	gas phase	any	16
Biphenylene	3^1A_g	5.30	5.2	0.10	valence	any	any	16
Biphenylene	2^1B_{2u}	5.75	5.7	0.05	valence	any	any	16
Biphenylene	3^1B_{2u}	6.07	6.2	−0.13	valence	any	any	16
trans-Stilbene	1^1B_u	3.77	3.86	−0.08	valence	gas phase	any	17

Table S1: … continued

Molecule	State	$\mathbf{V}^{calc}$	$\mathbf{V}^{exp}$	$\mathbf{\Delta} V$	Character	Environment	Method	Ref.
trans-Stilbene	2^1B_u	4.07	3.86	0.22	valence	gas phase	any	17
trans-Stilbene	2^1A_g	4.13	4.1	0.03	valence	any	any	17
trans-Stilbene	3^1A_g	4.95	5.02	−0.07	valence	any	any	17
trans-Stilbene	4^1A_g	5.30	5.3	0.00	valence	any	any	17
trans-Stilbene	3^1B_u	5.42	5.42	0.00	valence	any	any	17
trans-Stilbene	4^1B_u	5.42	5.42	0.00	valence	any	any	17
trans-Stilbene	6^1B_u	5.95	6.15	−0.20	valence	gas phase	any	17
trans-Stilbene	1^3B_u	2.56	2.17	0.39	valence	any	any	17
p-Aminobenzonitrile	$\pi\pi^*$	4.69	4.7	−0.01	valence	gas phase	any	18
p-Dimethylaminobenzonitrile	$\pi\pi^*$	4.38	4.4	−0.02	valence	gas phase	any	18
Nitroaniline	$\pi\pi^*$	3.73	3.84	−0.11	valence	gas phase	any	18
Pyrimidine	1^1B_1	4.26	4.2	0.06	valence	gas phase	standard	18
Pyrimidine	1^1A_2	4.59	4.62	−0.03	valence	gas phase	standard	18
Pyrimidine	1^1B_2	5.01	4.99	0.02	valence	gas phase	standard	18
Pyrimidine	2^1A_1	6.85	6.7	0.15	valence	gas phase	standard	18
Pyrimidine	2^1B_2	7.53	7.57	−0.04	valence	gas phase	standard	18
Pyrimidine	3^1A_1	7.56	7.57	−0.01	valence	gas phase	standard	18
Butadiene	1^1B_u	6.32	5.92	0.40	valence	gas phase	standard	18
Guanine	$2^1A'$	4.73	4.45	0.28	valence	gas phase	any	19
Guanine	$3^1A'$	5.11	4.95	0.16	valence	gas phase	any	19
Guanine	$4^1A'$	5.98	5.75	0.23	valence	gas phase	any	19
Guanine	$5^1A'$	6.49	6.2	0.29	valence	gas phase	any	19
Guanine	$6^1A'$	6.59	6.65	−0.06	valence	gas phase	any	19
Guanine	$7^1A'$	6.72	6.65	0.07	valence	gas phase	any	19
Guanine	$8^1A'$	6.74	6.65	0.09	valence	gas phase	any	19

Table S1: ... continued

Molecule	State	$\mathbf{V}^{calc}$	$\mathbf{V}^{exp}$	$\mathbf{\Delta}V$	Character	Environment	Method	Ref.
Acetylene	$1^1\Sigma_u^-$	6.96	7.1	−0.14	valence	gas phase	any	20
Acetylene	$1^1\Delta_u$	7.30	7.2	0.10	valence	gas phase	any	20
Acetylene	$1^1\Delta_u$	7.30	7.2	0.10	valence	gas phase	any	20
Acetylene	$1^1\Pi_u$	8.30	8.16	0.14	Rydberg	gas phase	any	20
Acetylene	$1^1\Pi_g$	8.60	9.01	−0.41	Rydberg	gas phase	any	20
Acetylene	$1^1\Delta_g$	8.96	9.01	−0.05	Rydberg	gas phase	any	20
Acetylene	$2^1\Sigma_g^+$	9.06	9.21	−0.15	Rydberg	gas phase	any	20
Acetylene	$2^1\Pi_u$	9.55	9.24	0.31	Rydberg	gas phase	any	20
Acetylene	$1^1\Sigma_u^+$	9.50	9.27	0.23	Rydberg	gas phase	any	20
Acetylene	$2^1\Delta_u$	9.73	10	−0.27	Rydberg	gas phase	any	20
Acetylene	$1^1\Phi_u$	9.93	9.91	0.02	Rydberg	gas phase	any	20
Acetylene	$3^1\Pi_u$	9.93	9.98	−0.05	Rydberg	gas phase	any	20
Acetylene	$1^3\Sigma_u^+$	5.26	5.2	0.06	valence	gas phase	any	20
Acetylene	$1^3\Delta_u$	6.20	6	0.20	valence	gas phase	any	20
Acetylene	$1^3\Sigma_u^-$	6.90	7.1	−0.2	valence	gas phase	any	20
Acetylene	$1^3\Pi_u$	8.19	8.03	0.16	Rydberg	gas phase	any	20
Acetylene	$1^3\Pi_g$	8.43	8.81	−0.38	Rydberg	gas phase	any	20
Acetylene	$1^3\Sigma_g^+$	8.80	9.03	−0.23	Rydberg	gas phase	any	20
Acetylene	$1^3\Delta_g$	8.88	8.96	−0.08	Rydberg	gas phase	any	20
Acetylene	$2^3\Pi_u$	9.42	9.18	0.25	Rydberg	gas phase	any	20
Porphin	1^1B_{3u}	1.63	1.98	−0.35	valence	gas phase	any	21
Porphin	1^1B_{2u}	2.11	2.42	−0.31	valence	gas phase	any	21
Porphin	2^1B_{2u}	3.08	3.33	−0.25	valence	gas phase	any	21
Porphin	2^1B_{3u}	3.12	3.33	−0.21	valence	gas phase	any	21
Porphin	3^1B_{2u}	3.42	3.65	−0.23	valence	gas phase	any	21

Table S1: . . . continued

Molecule	State	$\mathbf{V}^{\mathrm{calc}}$	$\mathbf{V}^{\mathrm{exp}}$	$\boldsymbol{\Delta}V$	Character	Environment	Method	Ref.
Porphin	3^1B_{3u}	3.53	3.65	−0.12	valence	gas phase	any	21
Porphin	4^1B_{2u}	3.96	4.25	−0.29	valence	gas phase	any	21
Porphin	3^1B_{3z}	4.04	4.25	−0.21	valence	gas phase	any	21
Porphin	1^3B_{2u}	1.52	1.58	−0.06	valence	any	any	21
Ethene	1^1B_{1u}	7.98	8	−0.02	valence	gas phase	any	22
Ethene	2^1B_{1u}	9.40	9.33	0.07	valence	gas phase	any	22
cis-Stilbene	2^1B	4.61	4.48	0.13	valence	any	any	23
cis-Stilbene	3^1B	5.46	5.54	−0.08	valence	any	any	23
cis-Stilbene	7^1B	6.00	6.14	−0.14	valence	any	any	23
s-Tetrazine	1^1B_{3u}	1.96	2.25	−0.29	valence	gas phase	any	24
s-Tetrazine	1^1A_u	3.06	3.4	−0.34	valence	gas phase	any	24
s-Tetrazine	2^1B_{2g}	5.48	5.5	−0.02	valence	gas phase	any	24
s-Tetrazine	2^1B_{1g}	5.99	5.9	0.09	valence	gas phase	any	24
s-Tetrazine	2^1B_{3u}	6.37	6.47	−0.10	valence	gas phase	any	24
s-Tetrazine	1^1B_{2u}	4.89	4.99	−0.09	valence	gas phase	any	24
s-Tetrazine	3^1B_{1u}	7.13	7.1	0.03	valence	gas phase	any	24
s-Tetrazine	5^1B_{1u}	7.54	7.6	−0.06	valence	gas phase	any	24
s-Tetrazine	4^1B_{2u}	7.94	8.3	−0.36	valence	gas phase	any	24
Styrene	$2^1A'$	4.34	4.33	0.01	valence	gas phase	any	25
Styrene	$3^1A'$	4.97	5.06	−0.09	valence	gas phase	any	25
Styrene	$5^1A'$	6.19	6.3	−0.11	valence	gas phase	any	25
Styrene	$1^3A'$	3.03	2.97	0.06	valence	gas phase	any	25
Styrene	$2^3A'$	4.09	3.98	0.11	valence	gas phase	any	25
Cyclopropene	1^1B_1	6.36	6.45	−0.09	valence	gas phase	standard	26
Cyclopropene	1^1B_2	7.54	7.1	0.45	valence	gas phase	standard	26

Table S1: ... continued

Molecule	State	$\mathbf{V}^{calc}$	$\mathbf{V}^{exp}$	$\mathbf{\Delta} V$	Character	Environment	Method	Ref.
Cyclopropene	3^1A_1	8.20	8.2	0.00	Rydberg	gas phase	standard	26
Cyclopropene	5^1B_2	8.97	9	−0.03	Rydberg	gas phase	standard	26
Cyclopropene	1^3B_2	4.18	4.16	0.02	valence	gas phase	standard	26
Cyclopropene	1^3B_1	6.05	6.1	−0.05	valence	gas phase	standard	26
Cyclopropene	2^3B_1	6.81	6.7	0.11	Rydberg	gas phase	standard	26
Indene	^1L_b	4.46	4.4	0.06	valence	any	any	27
Indene	^1L_a	5.02	5	0.02	valence	any	any	27
Indene	^1B_b	6.14	5.8	0.34	valence	any	any	27
Indene	^1B_a	6.36	5.9	0.46	valence	any	any	27
1,4-Cyclohexadiene	1^1B_{1g}	5.74	5.8	−0.06	valence	gas phase	standard	28
1,4-Cyclohexadiene	1^1B_{1u}	5.90	6.1	−0.2	Rydberg	gas phase	standard	28
1,4-Cyclohexadiene	1^1B_{3g}	6.53	6.42	0.11	Rydberg	gas phase	standard	28
1,4-Cyclohexadiene	2^1B_{3g}	6.67	6.65	0.02	Rydberg	gas phase	standard	28
1,4-Cyclohexadiene	1^1B_{3u}	7.16	7.05	0.11	valence	gas phase	standard	28
1,4-Cyclohexadiene	4^1B_{1u}	7.22	7.3	−0.08	Rydberg	gas phase	standard	28
1,4-Cyclohexadiene	3^1B_{3u}	7.49	7.75	−0.26	valence	gas phase	standard	28
1,4-Cyclohexadiene	1^3B_{3u}	4.29	4.08	0.21	valence	gas phase	standard	28
1,3-Cyclohexadiene	1^1B	4.72	4.94	−0.22	valence	gas phase	any	28
1,3-Cyclohexadiene	2^1A	5.49	5.39	0.10	Rydberg	gas phase	any	28
1,3-Cyclohexadiene	2^1B	5.98	6.03	−0.05	Rydberg	gas phase	any	28
1,3-Cyclohexadiene	3^1A	6.12	6.05	0.07	Rydberg	gas phase	any	28
1,3-Cyclohexadiene	4^1A	6.18	6.3	−0.12	valence	any	any	28
1,3-Cyclohexadiene	5^1A	6.64	6.68	−0.04	Rydberg	gas phase	any	28
1,3-Cyclohexadiene	6^1A	6.75	6.87	−0.12	Rydberg	gas phase	any	28
1,3-Cyclohexadiene	6^1B	7.72	7.7	0.02	Rydberg	gas phase	any	28

Table S1: … continued

**Molecule**	**State**	**V**^{calc}	**V**^{exp}	$\boldsymbol{\Delta V}$	**Character**	**Environment**	**Method**	**Ref.**
1,3-Cyclohexadiene	8^1A	7.77	7.75	0.02	valence	gas phase	any	28
1,3-Cyclohexadiene	7^1B	9.27	9	0.27	valence	gas phase	any	28
1,3-Cyclohexadiene	1^3B	2.91	2.94	−0.03	valence	gas phase	any	28
1,3-Cyclohexadiene	1^3A	4.79	4.5	0.29	valence	gas phase	any	28
Pyridine	$^1B_1$	4.93	4.74	0.19	valence	gas phase	standard	29
Pyridine	$^1A_2$	5.25	5.43	−0.18	valence	gas phase	standard	29
Pyridine	$^1B_2$	4.88	4.99	−0.11	valence	gas phase	standard	29
Pyridine	$^3A_1$	4.06	4.1	−0.04	valence	gas phase	standard	29
Pyridine	$^3A_1$	4.77	4.84	−0.07	valence	gas phase	standard	29
Pyridine	$^3A_2$	5.22	5.43	−0.21	valence	gas phase	standard	29
Pyridine	$^3B_2$	4.52	4.84	−0.32	valence	gas phase	standard	29
Nitrobenzene	1^1A_2	3.57	3.65	−0.08	valence	gas phase	standard	30
Nitrobenzene	1^1B_1	4.14	3.65	0.49	valence	gas phase	standard	30
Nitrobenzene	1^1B_2	4.40	4.38	0.02	valence	gas phase	standard	30
Nitrobenzene	2^1A_1	4.99	5.17	−0.18	valence	gas phase	standard	30
Nitrobenzene	3^1A_1	6.20	6.14	0.06	valence	gas phase	standard	30
Nitrobenzene	3^1B_2	6.34	6.42	−0.08	valence	gas phase	standard	30
Nitrobenzene	6^1A_1	6.95	7.56	−0.61	valence	gas phase	standard	30
Nitrobenzene	3^1A_2	6.96	7.56	−0.60	valence	gas phase	standard	30
Nitrobenzene	7^1B_2	7.46	7.56	−0.10	valence	gas phase	standard	30
Nitrobenzene	7^1A_1	7.52	7.56	−0.04	valence	gas phase	standard	30
Nitrobenzene	8^1B_2	7.74	7.56	0.18	valence	gas phase	standard	30
Indene	$1^3A'$	3.17	3.1	0.07	valence	any	any	31
Benzimidazole	$2^1A'$	4.56	4.5	0.06	valence	any	any	31
Benzimidazole	$3^1A'$	4.99	5	−0.01	valence	any	any	31

Table S1: . . . continued

**Molecule**	**State**	$\mathbf{V}^{\text{calc}}$	$\mathbf{V}^{\text{exp}}$	$\mathbf{\Delta}V$	**Character**	**Environment**	**Method**	**Ref.**
Benzimidazole	$4^1A'$	6.29	6.1	0.19	valence	any	any	31
7-azaindole	$2^1A'$	4.22	4.29	−0.07	valence	gas phase	any	31
7-azaindole	$3^3A'$	4.49	4.49	0.00	valence	any	any	31
7-azaindole	$4^1A'$	5.77	5.76	0.01	valence	any	any	31
7-azaindole	$5^1A'$	5.93	5.99	−0.06	valence	any	any	31
Benzaldehyde	$1^1A''$	3.71	3.34	0.37	valence	gas phase	any	32
Benzaldehyde	$2^1A'$	4.33	4.51	−0.18	Rydberg	gas phase	any	32
Benzaldehyde	$3^1A'$	4.89	5.25	−0.36	Rydberg	gas phase	any	32
Benzaldehyde	$5^1A'$	6.23	6.36	−0.13	Rydberg	gas phase	any	32
Benzaldehyde	$1^3A''$	3.40	3.12	0.28	valence	gas phase	any	32
Benzaldehyde	$1^3A'$	3.49	3.3	0.19	valence	gas phase	any	32
Pyrrole	1^1A_2	5.22	5.22	0.00	valence	gas phase	any	33
Pyrrole	1^1B_2	5.87	5.86	0.01	valence	gas phase	any	33
Pyrrole	1^1B_1	5.86	5.88	−0.02	valence	gas phase	any	33
Pyrrole	2^1B_2	5.70	5.98	−0.28	valence	gas phase	any	33
Cyclopropenon	2^1A_1	4.25	4.13	0.12	valence	gas phase	any	34
Cyclopropenon	3^1A_1	5.59	5.5	0.09	valence	gas phase	any	34
Cyclopropenon	2^1A_2	5.96	6.1	−0.14	valence	gas phase	any	34
Cyclopropenon	3^1B_1	7.80	8	−0.20	valence	gas phase	any	34
Cycloheptatrienone	$n\pi^*$	3.94	4.13	−0.19	valence	any	any	34
Cycloheptatrienone	$\pi\pi^*$	4.22	4.13	0.09	valence	any	any	34
Cycloheptatrienone	$\pi\pi^*$	5.67	5.56	0.11	valence	gas phase	any	34
Cycloheptatrienone	$\pi\pi^*$	7.05	6.9	0.15	valence	gas phase	any	34
Cycloheptatrienone	$\pi\pi^*$	7.16	6.9	0.26	valence	gas phase	any	34
Cycloheptatrienone	$\pi\pi^*$	7.36	7.2	0.16	valence	gas phase	any	34

Table S1: ... continued

**Molecule**	**State**	$\mathbf{V}^{calc}$	$\mathbf{V}^{exp}$	$\boldsymbol{\Delta V}$	**Character**	**Environment**	**Method**	**Ref.**
Cycloheptatrienthione	$n\pi^*$	2.05	1.82	0.23	valence	gas phase	any	34
Cycloheptatrienthione	$n\pi^*$	2.10	2.03	0.07	valence	gas phase	any	34
Cycloheptatrienthione	$\pi\pi^*$	2.31	2.03	0.28	valence	any	any	34
Cycloheptatrienthione	$\pi\pi^*$	3.21	3.34	−0.13	valence	any	any	34
Cycloheptatrienthione	$\pi\pi^*$	4.74	4.9	−0.16	valence	any	any	34
Cycloheptatrienthione	$\pi\pi^*$	4.80	4.9	−0.10	valence	any	any	34
Cycloheptatrienthione	$\pi\pi^*$	5.19	5.53	−0.34	valence	any	any	34
Cycloheptatrienthione	$\pi\pi^*$	5.22	5.53	−0.31	valence	any	any	34
Cycloheptatrienthione	$\pi\pi^*$	5.87	5.53	0.34	valence	any	any	34
p-Benzoquinone	1^1B_{1g}	2.39	2.49	−0.10	valence	gas phase	any	35
p-Benzoquinone	1^1A_u	2.43	2.48	−0.05	valence	gas phase	any	35
p-Benzoquinone	1^1B_{3g}	4.01	4.07	−0.06	valence	gas phase	any	35
p-Benzoquinone	1^1B_{1u}	5.09	5.12	−0.03	valence	gas phase	any	35
p-Benzoquinone	1^1B_{2u}	6.83	7.01	−0.18	Rydberg	any	any	35
p-Benzoquinone	4^1B_{1u}	7.18	7.1	0.08	valence	gas phase	any	35
p-Benzoquinone	3^1B_{2u}	7.53	7.5	0.03	Rydberg	any	any	35
p-Benzoquinone	3^1B_{3u}	7.76	7.8	−0.04	Rydberg	any	any	35
p-Benzoquinone	5^1B_{1u}	7.81	7.8	0.01	Rydberg	any	any	35
p-Benzoquinone	5^1B_{2u}	8.02	7.8	0.22	Rydberg	any	any	35
p-Benzoquinone	1^3B_{1g}	2.16	2.3	−0.14	valence	gas phase	any	35
p-Benzoquinone	1^3A_u	2.22	2.33	−0.11	valence	gas phase	any	35
p-Benzoquinone	1^3B_{1u}	2.57	2.84	−0.27	valence	any	any	35
p-Benzoquinone	1^3B_{3g}	3.09	3	0.09	valence	any	any	35
p-Benzoquinone	2^3B_{1u}	4.35	4.37	−0.02	valence	any	any	35
Maleimide	1^1A_2	3.29	3.33	−0.04	valence	any	any	36

Table S1: ... continued

Molecule	State	V^{calc}	V^{exp}	ΔV	Character	Environment	Method	Ref.
Maleimide	1^1B_2	4.44	4.46	−0.02	valence	any	any	36
Maleimide	2^1B_2	5.59	5.72	−0.13	valence	any	any	36
1H-Benzotriazole	$2^1A'$	4.48	4.5	−0.02	valence	gas phase	any	37
1H-Benzotriazole	$3^1A'$	5.05	5.2	−0.15	valence	gas phase	any	37
1H-Benzotriazole	$4^1A'$	6.27	6.2	0.07	valence	any	any	37
1H-Benzotriazole	$5^1A'$	6.28	6.2	0.08	valence	any	any	37
2H-Benzotriazole	2^1A_1	4.54	4.5	0.04	valence	gas phase	any	37
2H-Benzotriazole	1^1B_2	4.60	4.5	0.10	valence	gas phase	any	37
Acrolein	$1^1A''$	3.63	3.73	−0.1	valence	gas phase	any	37
Acrolein	$2^1A'$	6.10	6.41	−0.31	valence	gas phase	any	38
Acrolein	$^1A'$	6.97	7.08	−0.11	Rydberg	gas phase	any	38
Acrolein	$^1A'$	7.49	7.38	0.11	Rydberg	gas phase	any	38
Acrolein	$^1A'$	7.55	7.51	0.04	Rydberg	gas phase	any	38
Acrolein	$^1A'$	8.29	8.37	−0.08	Rydberg	gas phase	any	38
Acrolein	$^1A''$	7.76	7.75	0.01	Rydberg	gas phase	any	38
Acrolein	$^1A'$	8.45	8.49	−0.04	Rydberg	gas phase	any	39
Azulene	1^1B_2	1.66	1.78	−0.12	valence	gas phase	any	39
Azulene	2^1A_1	3.67	3.56	0.11	valence	gas phase	any	39
Azulene	2^1B_2	4.07	4.23	−0.16	valence	gas phase	any	39
Azulene	3^1A_1	4.63	4.4	0.23	valence	gas phase	standard	39
2,2-Bithiophene	1^1B_u	4.22	4.13	0.09	valence	gas phase	standard	40
2,2-Bithiophene	2^1B_u	4.36	4.13	0.23	valence	gas phase	standard	40
2,2-Bithiophene	1^3B_u	2.07	2.26	−0.19	valence	gas phase	standard	40
Acetone	$^1A_2$	4.49	4.38	0.11	valence	gas phase	standard	41
Acetone	$^1B_2$	6.57	6.35	0.22	Rydberg	gas phase	standard	41

Table S1: . . . continued

Molecule	State	$\mathbf{V}^{calc}$	$\mathbf{V}^{exp}$	$\mathbf{\Delta}V$	Character	Environment	Method	Ref.
Acetone	$^1B_2$	7.54	7.45	0.09	Rydberg	gas phase	standard	41
Acetone	$^1A_1$	7.73	7.41	0.32	Rydberg	gas phase	standard	41
Acetone	$^1A_2$	7.75	7.36	0.39	Rydberg	gas phase	standard	41
Acetone	$^1A_1$	7.86	7.8	0.06	Rydberg	gas phase	standard	41
Acetone	$^1B_2$	8.14	8.09	0.05	Rydberg	gas phase	standard	41
Acetone	$^1B_1$	8.33	8.17	0.16	Rydberg	gas phase	standard	41

S2 CASPT2/CASSCF vs. FCI Benchmark

S2.1 Computational Details

The general computational details for our FCI/CASPT2 benchmark calculations are given in section 4.1 in the main paper. Here, we only discuss a few additional issues. All states comprised in our benchmark set are given in Tables S4 and S5 for the 6-31G and 6-311G basis sets, respectively.

As may be seen by inspecting these tables, the number of low-lying electronic states considered varies from molecule to molecule. Each number was inspired by results obtained in previous FCI calculations[42–49] and the goal to cover the most probable low-lying excitations that could be expected on the basis of simple orbital considerations regarding only the valence orbitals in the active spaces.

Initially, we pondered to use the already published FCI results for our reference data. However, most of these studies use different basis sets often augmented with diffuse function to also describe Rydberg states. Since our aim was only to investigate valence excited states using a consistent treatment for all the molecules, all the FCI calculations were repeated using the 6-31G and 6-311G basis sets.

A comment is important here regarding the effect of lowering the symmetry in the calculations of the linear diatomic molecules. Symmetry lowering was unavoidable since both MOLPRO and MOLCAS only support the usage of Abelian point groups with the highest symmetric point group being D_{2h}. In Abelian point groups, there exist only one-dimensional irreducible representations. Thus, states that are degenerate by symmetry in the linear point groups, e.g., Π or Δ states, transform according to different irreducible representations. The correspondence of the irreducible representations is shown in Table S2.

A symmetry lowering does not affect the degeneracies in the FCI calculations, but it can have an effect on the degeneracies in the CASSCF/CASPT2 calculations. When computing Σ and Δ states, one of the components of the Δ state and the Σ state can fall into the same irreducible representation. Since we used state-averaged CASSCF, the CASSCF wave functions of both components of the Δ state then will differ leading to splitting of the energies of both components. The average splitting of the CASSCF energies is 0.07 eV and it is reduced again in the multi-state CASPT2 treatment to 0.05 eV. Note that the Π states are not affected by

Table S2: Correspondence of irreducible representations of the linear point groups $D_{\infty h}$ and $C_{\infty v}$ with the Abelian point groups D_{2h} and C_{2v} used in the calculation, respectively.

$\boldsymbol{D_{\infty h}}$	$\boldsymbol{D_{2h}}$	$\boldsymbol{C_{\infty v}}$	$\boldsymbol{C_{2v}}$
Σ_g^+	A_g	Σ^+	A_1
Σ_g^+	B_{1g}	Σ^-	A_2
Π_g	$B_{2g} \oplus B_{3g}$	Π	$B_1 \oplus B_2$
Δ_g	$A_g \oplus B_{1g}$	Δ	$A_1 \oplus A_2$
Σ_u^+	B_{1u}		
Σ_u^+	A_u		
Π_u	$B_{2u} \oplus B_{3u}$		
Δ_u	$B_{1u} \oplus A_u$		

symmetry lowering since no single states fall into one of the irreducible representations of the individual components. To avoid double counting when computing the mean errors, only one component of each Π state is considered and we simply take the mean value of the energies of both Δ components since it does not have a significant effect on the size of the errors.

S2.2 Closed-Shell vs. Open-Shell Characterization

In this section, we explain how we determined whether a states is "closed-shell" or "open-shell". Let us first consider only the molecules with an even number of electrons. For them, each electronic state is described in either a FCI or a CASSCF/CASPT2 calculations by linear combination of both closed-shell and open-shell determinants or configurational state functions (which are spin-adapted linear combinations of single determinants) thus being neither purely closed-shell nor purely open-shell. Still, the majority of the states are dominated by a single or a small number of configurations giving them approximately closed-shell or open-shell character. To introduce this characterization for each state, we considered the leading configurations of the wave functions obtained by a MS-CASPT2 treatment and calculated the mean number of open shells (NOS) by counting the number of open shells of each configuration and weighting it by the corresponding coefficient of the configuration in the wave function. To illustrate this with one example, consider a wave function represented by a single configuration. If all the orbitals are doubly occupied, NOS = 0. When one electron is promoted from an occupied to an unoccupied orbital, NOS = 2 as two orbitals are singly occupied. Promoting a second electron can already give rise to three different cases: NOS = 0, NOS = 2, and NOS = 4. In the averaging we did not consider the complete wave function, but considered only the

configurations with a coefficient larger than a threshold of 0.05, which is set as default print level in a MOLCAS CASPT2 calculation.

Then, we finally characterize every state with NOS < 0.5 as closed-shell and every state with NOS > 0.5 as open-shell. We chose this smaller value of NOS $= 0.5$ rather than NOS $= 1$ for discrimination as our main interest still lies in the difference between the performance of NOIPEA and IPEA CASPT2. When a state possesses an already substantial amount of open-shell character, one should expect a sizable impact of the IPEA shift. We applied the same procedure also to all states of the molecules with an odd number of electrons. Here, necessarily, NOS > 1, as we have at least one partially filled shell. However, as the effect of the IPEA shift should scale with the difference in the number of open shells, we simply shift our criterion by 1, i.e., NOS < 1.5 and NOS > 1.5 for closed-shell and open-shell states of molecules with an odd number of electrons. The NOS values of all states are reported in Tables S4 and S5.

Using the NOS criterion to characterize a state as closed-shell or open-shell bring us to the question whether or not to include both components of each Δ state instead of their average results when calculating the errors. Due to the symmetry lowering, both components fall into different irreducible representations in the lower-symmetry point group and their wave functions differ in the number of open shells. Consider for example the $1\ ^1\Delta$ state belonging to the $(1\sigma^+)^2(2\sigma^+)^2(1\pi 1\pi)^2$ electronic configuration of the HB molecule. In the lowered C_{2v} symmetry the $^1\Delta$ state splits up into a $^1A_1$ and a $^1A_2$ state. The σ^+ orbitals become a_1 orbitals while the 1π orbitals split up into the $1b_1$ and $1b_2$ orbitals. The wave function of the $^1A_1$ component is given by the linear combination of the configuration where both electrons are in the same orbitals, i.e.,

$$\left|\Psi(^1A_1)\right\rangle = c_{A_1}\Bigg(\left|(1a_1)^2(2a_1)^2(1b_1)^2(1b_2)^0\right| - \left|(1a_1)^2(2a_1)^2(1b_1)^0(1b_2)^2\right| \Bigg) + \dots \tag{S1}$$

while in the leading configuration of the wave function of the $^1A_2$ component the electrons in the π orbitals are in the symmetry-distinct b_1 and b_2 orbitals:

$$\left|\Psi(^1A_2)\right\rangle = c_{A_2}\left|(1a_1)^2(2a_1)^2(1b_1)^1(1b_2)^1\right| + \dots \tag{S2}$$

In the original $C_{\infty v}$ point group, both components should be characterized as open-shell, since the $(1\pi 1\pi)$ double shell is not fully occupied. In the lowered C_{2v} symmetry, NOS$(^1A_2) \approx 2$

while NOS($^1A_1$) $\approx$ 0, making the latter state "closed-shell" by our definition. Thus, both components are affected differently when employing the IPEA-shifted CASPT2 and to capture this difference, we decided to include both components of each Δ state when calculating the errors in Table 2.

S2.3 Ground State Geometries of Di- and Triatomic Molecules

The Cartesian coordinates of the ground-state geometries of the di- and triatomic molecules used in the CASSCF/CASPT2 vs. FCI benchmark are given in Table S3. The geometries of the diatomic molecules were taken from the NIST database[50] while for the triatomic molecules we used geometries employed in previous FCI studies.[43,45]

Table S3: Cartesian coordinates in Å of of the di- and triatomic molecules used in the FCI and CASSCF/CASPT2 calculations. Ground-state geometries adopted from the NIST database and previous FCI studies.[43,45,50]

**Atom**	**x**	**y**	**z**
HLi			
H	0.000000	0.00000	0.79785
Li	0.000000	0.00000	−0.79785
HBe			
H	0.67130	0.00000	0.00000
Be	−0.67130	0.00000	0.00000
HB			
H	0.00000	0.00000	0.61620
B	0.00000	0.00000	−0.61620
HC			
H	0.00000	0.00000	−0.55995
C	0.00000	0.00000	0.55995
HN			
H	0.00000	0.00000	−0.51810
N	0.00000	0.00000	0.51810
HO			
H	0.00000	0.00000	−0.48483
O	0.00000	0.00000	0.48483

Table S3: ... continued

Atom	x	y	z
HF			
H	0.00000	0.00000	0.45840
F	0.00000	0.00000	−0.45840
Li_2			
Li	0.00000	0.00000	1.33645
Li	0.00000	0.00000	−1.33645
B_2			
B	0.00000	0.00000	0.79500
B	0.00000	0.00000	−0.79500
C_2			
C	0.00000	0.00000	0.62125
C	0.00000	0.00000	−0.62125
N_2			
N	0.00000	0.000000	0.54884
N	0.00000	0.000000	−0.54884
H_2O			
O	0.00000	0.00000	0.00000
H	0.00000	0.75667	−0.58588
H	0.00000	−0.75667	−0.58588
CH_2			
C	0.00000	0.00000	0.00000
H	0.87016	0.00000	0.69963
H	−0.87016	0.00000	0.69963

S2.4 Excited State Energies of Di- and Triatomic Molecules

The total energies and vertical excitation energies at FCI, CASPT2, and CASSCF levels of theory of the set of di- and triatomic molecules computed using the 6-31G and 6-311G basis sets are presented in Tables S4 and S5, respectively. Every state of the linear molecules is

classified according to its irreducible representation in the original linear molecular point group as well as the Abelian point group used in the calculations. In addition, for characterization, we give the transition corresponding to the leading configuration in the wave function expansion for each excited state as well as the average NOS of the wave function calculated at CASPT2 (IPEA) level of theory. The corresponding NOS values obtained at the other levels of theory are practically the same and therefore not reprinted here.

Table S4: Total energies E_X in a.u. and vertical excitation energies V_X in eV of di- and triatomic molecules (X = FCI, IPEA, NOIPEA, CASSCF) calculated using the 6-31G basis set.

Molecule	State	Irrep	Transition	NOS	$\mathbf{E}_\mathrm{FCI}$	$\mathbf{V}_\mathrm{FCI}$	$\mathbf{E}_\mathrm{IPEA}$	$\mathbf{V}_\mathrm{IPEA}$	$\mathbf{E}_\mathrm{NOIPEA}$	$\mathbf{V}_\mathrm{NOIPEA}$	$\mathbf{E}_\mathrm{CASSCF}$	$\mathbf{V}_\mathrm{CASSCF}$
HLi	$1\ ^1\Sigma^+$	$1\ ^1A_1$	ground state	0.27	−7.998288	0.00	−7.992655	0.00	−7.992874	0.00	−7.976800	0.00
HLi	$1\ ^3\Sigma^+$	$1\ ^3A_1$	$2\sigma^+$ $3\sigma^+$	2.00	−7.894657	2.82	−7.894628	2.67	−7.894649	2.67	−7.894194	2.25
HLi	$2\ ^1\Sigma^+$	$2\ ^1A_1$	$2\sigma^+$ $3\sigma^+$	1.88	−7.877487	3.29	−7.875388	3.19	−7.875664	3.19	−7.866460	3.00
HLi	$1\ ^3\Pi$	$1\ ^3B_1$	$2\sigma^+$ 1π	2.00	−7.850984	4.01	−7.850957	3.86	−7.850978	3.86	−7.850665	3.43
HLi	$1\ ^1\Pi$	$1\ ^1B_1$	$2\sigma^+$ 1π	2.00	−7.839268	4.33	−7.839206	4.18	−7.839259	4.18	−7.838661	3.76
HLi	$2\ ^3\Sigma^+$	$2\ ^3A_1$	$2\sigma^+$ $4\sigma^+$	2.00	−7.780935	5.91	−7.780903	5.76	−7.780936	5.77	−7.780437	5.34
HLi	$3\ ^1\Sigma^+$	$3\ ^1A_1$	$2\sigma^+$ $4\sigma^+$	1.85	−7.739166	7.05	−7.737090	6.95	−7.737338	6.95	−7.730082	6.71
HBe	$1\ ^2\Sigma^+$	$1\ ^2A_1$	ground state	1.00	−15.168530	0.00	−15.164473	0.00	−15.164866	0.00	−15.151019	0.00
HBe	$1\ ^2\Pi$	$1\ ^2B_1$	$3\sigma^+$ 1π	1.38	−15.071940	2.63	−15.070017	2.57	−15.070269	2.57	−15.064755	2.35
HBe	$2\ ^2\Sigma^+$	$2\ ^2A_1$	$2\sigma^+$ $3\sigma^+$	1.04	−14.959181	5.70	−14.956713	5.65	−14.957606	5.64	−14.944579	5.62
HBe	$1\ ^4\Pi$	$1\ ^4B_1$	$2\sigma^+$ 1π	3.00	−14.956132	5.78	−14.955511	5.69	−14.955753	5.69	−14.953289	5.38
HBe	$2\ ^2\Pi$	$2\ ^2B_1$	$2\sigma^+$ 1π	2.64	−14.883226	7.76	−14.881596	7.70	−14.882155	7.69	−14.876424	7.47
HBe	$1\ ^4\Sigma^-$	$1\ ^4A_2$	$2\sigma^+3\sigma^+$ $1\pi1\pi$	3.00	−14.857782	8.46	−14.856580	8.38	−14.857143	8.37	−14.853365	8.10
HBe	$3\ ^2\Sigma^+$	$3\ ^2A_1$	$3\sigma^+$ $4\sigma^+$	1.04	−14.844509	8.82	−14.837514	8.90	−14.839366	8.86	−14.819111	9.03
HB	$1\ ^1\Sigma^+$	$1\ ^1A_1$	ground state	0.16	−25.172658	0.00	−25.166538	0.00	−25.166932	0.00	−25.146715	0.00
HB	$1\ ^3\Pi$	$1\ ^3B_1$	$3\sigma^+$ 1π	2.02	−25.135195	1.02	−25.131391	0.96	−25.132062	0.95	−25.117839	0.79
HB	$1\ ^1\Pi$	$1\ ^1B_1$	$3\sigma^+$ 1π	2.05	−25.057804	3.13	−25.053161	3.09	−25.054220	3.07	−25.035203	3.03
HB	$1\ ^3\Sigma^-$	$1\ ^3A_2$	$3\sigma^+3\sigma^+$ $1\pi1\pi$	2.15	−25.011959	4.37	−25.009182	4.28	−25.010086	4.27	−24.998528	4.03
HB	$1\ ^1\Delta$	$1\ ^1A_2$	$3\sigma^+3\sigma^+$ $1\pi1\pi$	2.00	−24.938543	6.37	−24.934352	6.32	−24.935545	6.30	−24.922057	6.11
HB	$1\ ^1\Delta$	$2\ ^1A_1$	$3\sigma^+3\sigma^+$ $1\pi1\pi$	0.00	−24.938543	6.37	−24.932211	6.38	−24.933162	6.36	−24.911989	6.39
HB	$1\ ^5\Sigma^-$	$1\ ^5A_2$	$2\sigma^+3\sigma^+$ $1\pi1\pi$	4.00	−24.904910	7.29	−24.903268	7.16	−24.904327	7.15	−24.894818	6.85
HB	$2\ ^1\Sigma^+$	$3\ ^1A_1$	$3\sigma^+3\sigma^+$ $1\pi1\pi$	0.02	−24.897303	7.49	−24.890055	7.52	−24.891412	7.50	−24.864102	7.69
HB	$1\ ^3\Sigma^+$	$1\ ^3A_1$	$3\sigma^+$ $4\sigma^+$	2.00	−24.872277	8.17	−24.867938	8.13	−24.868977	8.11	−24.853783	7.97
HB	$2\ ^3\Pi$	$2\ ^3B_1$	$2\sigma^+$ 1π	2.03	−24.868032	8.29	−24.864302	8.22	−24.865521	8.20	−24.850008	8.07

Table S4: . . . continued

Molecule	State	Irrep	Transition		NOS	E_{FCI}	V_{FCI}	E_{IPEA}	V_{IPEA}	E_{NOIPEA}	V_{NOIPEA}	E_{CASSCF}	V_{CASSCF}
HB	$3\,^1\Sigma^+$	$4\,^1A_1$	$3\sigma^+$	$4\sigma^+$	1.84	−24.779361	10.70	−24.770581	10.77	−24.772025	10.75	−24.744664	10.94
HB	$2\,^1\Pi$	$2\,^1B_1$	$2\sigma^+$	1π	2.02	−24.773093	10.87	−24.768335	10.84	−24.770627	10.78	−24.748111	10.85
HC	$1\,^2\Pi$	$1\,^2B_1$	ground state		1.12	−38.317559	0.00	−38.308791	0.00	−38.309691	0.00	−38.275349	0.00
HC	$1\,^4\Sigma^-$	$1\,^4A_2$	$3\sigma^+$	1π	3.04	−38.314493	0.08	−38.309690	−0.02	−38.311301	−0.04	−38.284238	−0.24
HC	$1\,^2\Delta$	$1\,^2A_1$	$3\sigma^+$	1π	1.02	−38.203321	3.11	−38.196137	3.06	−38.198138	3.03	−38.163163	3.05
HC	$1\,^2\Delta$	$1\,^2A_2$	$3\sigma^+$	1π	3.02	−38.203321	3.11	−38.196769	3.05	−38.198935	3.01	−38.168323	2.91
HC	$1\,^2\Sigma^-$	$2\,^2A_2$	$3\sigma^+$	1π	3.05	−38.192365	3.41	−38.183537	3.41	−38.185315	3.38	−38.157630	3.20
HC	$1\,^2\Sigma^+$	$2\,^2A_1$	$3\sigma^+$	1π	1.02	−38.170008	4.02	−38.162050	3.99	−38.164553	3.95	−38.124533	4.10
HC	$2\,^2\Pi$	$2\,^2B_2$	$3\sigma^+3\sigma^+$	$1\pi1\pi$	1.06	−38.030392	7.81	−38.020737	7.84	−38.021813	7.83	−37.982165	7.98
HC	$2\,^2\Sigma^+$	$3\,^2A_1$	1π	$4\sigma^+$	1.02	−38.016478	8.19	−38.007106	8.22	−38.008447	8.20	−37.973916	8.27
HC	$1\,^4\Pi$	$1\,^4B_1$	$3\sigma^+$	$4\sigma^+$	3.00	−38.002555	8.57	−37.995977	8.51	−37.998066	8.48	−37.966952	8.39
HC	$2\,^4\Sigma^-$	$2\,^4A_2$	$2\sigma^+$	1π	3.00	−37.983167	9.10	−37.978372	8.99	−37.980152	8.97	−37.951370	8.82
HC	$3\,^2\Pi$	$3\,^2B_1$	$3\sigma^+$	$4\sigma^+$	2.98	−37.939841	10.28	−37.933685	10.21	−37.936593	10.15	−37.902946	10.13
HC	$4\,^2\Pi$	$4\,^2B_1$	$3\sigma^+$	$4\sigma^+$	2.92	−37.882900	11.83	−37.873849	11.84	−37.878405	11.74	−37.831697	12.07
HC	$2\,^2\Delta$	$3\,^2A_2$	$2\sigma^+$	1π	3.00	−37.860442	12.44	−37.847611	12.55	−37.850254	12.50	−37.820397	12.38
HC	$2\,^2\Delta$	$4\,^2A_1$	$2\sigma^+$	1π	1.03	−37.860442	12.44	−37.853649	12.38	−37.857009	12.31	−37.819169	12.37
HC	$2\,^4\Pi$	$2\,^4B_1$	$2\sigma^+3\sigma^+$	$1\pi1\pi$	3.00	−37.843183	12.91	−37.835232	12.89	−37.837092	12.86	−37.798862	12.97
HN	$1\,^3\Sigma^-$	$1\,^3A_2$	ground state		2.11	−55.012997	0.00	−55.001825	0.00	−55.003849	0.00	−54.943921	0.00
HN	$1\,^1\Delta$	$1\,^1A_1$	ground state		0.03	−54.940550	1.97	−54.932055	1.90	−54.933834	1.91	−54.875231	1.87
HN	$1\,^1\Delta$	$1\,^1A_2$	ground state		2.10	−54.940550	1.97	−54.930126	1.95	−54.932227	1.95	−54.871892	1.96
HN	$1\,^1\Sigma^+$	$2\,^1A_1$	ground state		0.05	−54.912466	2.74	−54.904094	2.66	−54.906234	2.66	−54.844115	2.72
HN	$1\,^3\Pi$	$1\,^3B_1$	$3\sigma^+$	1π	2.01	−54.873191	3.80	−54.863587	3.76	−54.865370	3.77	−54.806500	3.74
HN	$1\,^1\Pi$	$1\,^1B_2$	$3\sigma^+$	1π	2.02	−54.793632	5.97	−54.783156	5.95	−54.785278	5.95	−54.718202	6.14
HN	$1\,^5\Sigma^-$	$1\,^5A_2$	$3\sigma^+$	$4\sigma^+$	4.00	−54.682608	8.99	−54.676073	8.86	−54.678413	8.86	−54.638685	8.31
HN	$2\,^3\Pi$	$2\,^3B_2$	1π	$4\sigma^+$	2.01	−54.653530	9.78	−54.643710	9.74	−54.645781	9.74	−54.596211	9.46

Table S4: . . . continued

**Molecule**	**State**	**Irrep**	**Transition**		**NOS**	**E_{FCI}**	**V_{FCI}**	**E_{IPEA}**	**V_{IPEA}**	**E_{NOIPEA}**	**V_{NOIPEA}**	**E_{CASSCF}**	**V_{CASSCF}**
HN	$2\ ^1\Pi$	$2\ ^1B_1$	1π	$4\sigma^+$	2.01	−54.628334	10.47	−54.620289	10.38	−54.621755	10.40	−54.575868	10.02
HN	$2\ ^3\Sigma^-$	$2\ ^3A_2$	$3\sigma^+$	$4\sigma^+$	3.97	−54.606234	11.07	−54.602816	10.86	−54.606992	10.80	−54.553289	10.63
HN	$2\ ^1\Sigma^+$	$3\ ^1A_1$	$3\sigma^+3\sigma^+$	$1\pi1\pi$	0.01	−54.595484	11.36	−54.571286	11.72	−54.571775	11.76	−54.522499	11.47
HN	$1\ ^3\Delta$	$3\ ^3A_2$	$3\sigma^+$	$4\sigma^+$	4.00	−54.555943	12.44	−54.548549	12.33	−54.550929	12.32	−54.507259	11.88
HN	$1\ ^3\Delta$	$1\ ^3A_1$	$3\sigma^+$	$4\sigma^+$	2.00	−54.555943	12.44	−54.549594	12.31	−54.552159	12.29	−54.509907	11.81
HN	$1\ ^1\Sigma^-$	$2\ ^1A_2$	$3\sigma^+$	$4\sigma^+$	4.00	−54.535117	13.00	−54.527317	12.91	−54.530056	12.89	−54.481969	12.57
HN	$3\ ^3\Sigma^-$	$4\ ^3A_2$	$3\sigma^+$	$4\sigma^+$	3.96	−54.527655	13.21	−54.522835	13.03	−54.528938	12.92	−54.457496	13.24
HN	$1\ ^3\Sigma^+$	$2\ ^3A_1$	$3\sigma^+$	$4\sigma^+$	2.00	−54.523622	13.32	−54.516457	13.21	−54.519694	13.17	−54.474378	12.78
HN	$2\ ^1\Delta$	$4\ ^1A_1$	$3\sigma^+$	$4\sigma^+$	1.94	−54.465807	14.89	−54.463406	14.65	−54.468857	14.56	−54.387178	15.15
HN	$2\ ^1\Delta$	$3\ ^1A_2$	$3\sigma^+$	$4\sigma^+$	3.91	−54.465807	14.89	−54.457928	14.80	−54.462978	14.72	−54.393155	14.99
HN	$3\ ^3\Pi$	$3\ ^3B_2$	$2\sigma^+$	1π	2.05	−54.455919	15.16	−54.446022	15.12	−54.449447	15.09	−54.389739	15.08
HO	$1\ ^2\Pi$	$1\ ^2B_1$	ground state		1.09	−75.462853	0.00	−75.452928	0.00	−75.454547	0.00	−75.362404	0.00
HO	$1\ ^2\Sigma^+$	$1\ ^2A_1$	$3\sigma^+$	1π	1.01	−75.304058	4.32	−75.293485	4.34	−75.294939	4.34	−75.195876	4.53
HO	$1\ ^4\Sigma^-$	$1\ ^4A_2$	1π	$4\sigma^+$	3.00	−75.169726	7.98	−75.162359	7.91	−75.164385	7.90	−75.102141	7.08
HO	$1\ ^2\Sigma^-$	$1\ ^2A_2$	1π	$4\sigma^+$	3.00	−75.133520	8.96	−75.126199	8.89	−75.128271	8.88	−75.068876	7.99
HO	$1\ ^2\Delta$	$2\ ^2A_2$	1π	$4\sigma^+$	1.00	−75.069778	10.70	−75.065742	10.54	−75.067915	10.52	−74.998242	9.91
HO	$1\ ^2\Delta$	$2\ ^2A_1$	1π	$4\sigma^+$	3.00	−75.069778	10.70	−75.061872	10.64	−75.063574	10.64	−75.004438	9.74
HO	$1\ ^4\Pi$	$1\ ^4B_1$	$3\sigma^+$	$4\sigma^+$	3.00	−75.051577	11.19	−75.044207	11.12	−75.046148	11.11	−74.985722	10.25
HO	$2\ ^2\Sigma^+$	$3\ ^2A_1$	1π	$4\sigma^+$	1.01	−75.033315	11.69	−75.028516	11.55	−75.031289	11.52	−74.960318	10.94
HO	$2\ ^2\Pi$	$2\ ^2B_1$	$3\sigma^+$	$4\sigma^+$	2.97	−74.983683	13.04	−74.980647	12.85	−74.984061	12.80	−74.907053	12.39
HO	$3\ ^2\Pi$	$3\ ^2B_2$	$3\sigma^+$	$4\sigma^+$	2.94	−74.921943	14.72	−74.920548	14.49	−74.926040	14.38	−74.824471	14.64
HF	$1\ ^1\Sigma^+$	$1\ ^1A_1$	ground state		0.08	−100.115685	0.00	−100.110804	0.00	−100.111473	0.00	−99.981696	0.00
HF	$1\ ^3\Pi$	$1\ ^3B_1$	1π	$4\sigma^+$	2.00	−99.736831	10.31	−99.731707	10.32	−99.732999	10.30	−99.644708	9.17
HF	$1\ ^1\Pi$	$1\ ^1B_1$	1π	$4\sigma^+$	2.00	−99.713030	10.96	−99.708107	10.96	−99.709176	10.95	−99.623395	9.75
HF	$1\ ^3\Sigma^+$	$1\ ^3A_1$	$3\sigma^+$	$4\sigma^+$	2.00	−99.618485	13.53	−99.613212	13.54	−99.614382	13.53	−99.527784	12.35

Table S4: ... continued

**Molecule**	**State**	**Irrep**	**Transition**		**NOS**	$\mathbf{E}_{\text{FCI}}$	$\mathbf{V}_{\text{FCI}}$	$\mathbf{E}_{\text{IPEA}}$	$\mathbf{V}_{\text{IPEA}}$	$\mathbf{E}_{\text{NOIPEA}}$	$\mathbf{V}_{\text{NOIPEA}}$	$\mathbf{E}_{\text{CASSCF}}$	$\mathbf{V}_{\text{CASSCF}}$
HF	$2\ {}^1\Sigma^+$	$2\ {}^1A_1$	$3\sigma^+$	$4\sigma^+$	1.92	−99.520444	16.20	−99.525469	15.93	−99.528197	15.87	−99.394340	15.98
Li_2	$1\ {}^1\Sigma_g^+$	$1\ {}^1A_g$	ground state		0.04	−14.892789	0.00	−14.891582	0.00	−14.891670	0.00	−14.889939	0.00
Li_2	$1\ {}^3\Sigma_u^+$	$1\ {}^3B_{1u}$	$2\sigma_g^+$	$2\sigma_u^+$	2.00	−14.849371	1.18	−14.849363	1.15	−14.849366	1.15	−14.849336	1.10
Li_2	$1\ {}^3\Pi_u$	$1\ {}^3B_{3u}$	$2\sigma_g^+$	$1\pi_u$	2.00	−14.840609	1.42	−14.840355	1.39	−14.840439	1.39	−14.839860	1.36
Li_2	$1\ {}^1\Sigma_u^+$	$1\ {}^1B_{1u}$	$2\sigma_g^+$	$2\sigma_u^+$	2.00	−14.824579	1.86	−14.824564	1.82	−14.824572	1.83	−14.824507	1.78
Li_2	$2\ {}^1\Sigma_g^+$	$2\ {}^1A_g$	$2\sigma_g^+$	$3\sigma_g^+$	1.44	−14.787198	2.87	−14.786839	2.85	−14.787007	2.85	−14.785327	2.85
Li_2	$1\ {}^1\Pi_u$	$1\ {}^1B_{3u}$	$2\sigma_g^+$	$1\pi_u$	2.00	−14.784730	2.94	−14.784199	2.92	−14.784311	2.92	−14.783587	2.89
B_2	$1\ {}^3\Sigma_g^-$	$1\ {}^3B_{1g}$	ground state		2.13	−49.218929	0.00	−49.213932	0.00	−49.215453	0.00	−49.195990	0.00
B_2	$1\ {}^5\Sigma_u^-$	$1\ {}^5A_u$	$2\sigma_u^+$	$3\sigma_g^+$	4.00	−49.218158	0.02	−49.214288	−0.01	−49.215643	−0.01	−49.199098	−0.08
B_2	$1\ {}^1\Delta_g$	$1\ {}^1A_g$	ground state		0.25	−49.191996	0.73	−49.186849	0.74	−49.188326	0.74	−49.167795	0.77
B_2	$1\ {}^1\Delta_g$	$1\ {}^1B_{1g}$	ground state		2.12	−49.191996	0.73	−49.187420	0.72	−49.188843	0.72	−49.170573	0.69
B_2	$1\ {}^3\Pi_u$	$1\ {}^3B_{2u}$	$1\pi_u$	$3\sigma_g^+$	2.09	−49.185414	0.91	−49.179414	0.94	−49.181019	0.94	−49.159703	0.99
B_2	$1\ {}^1\Sigma_g^+$	$2\ {}^1A_g$	ground state		0.26	−49.184183	0.95	−49.178999	0.95	−49.180725	0.95	−49.158896	1.01
B_2	$1\ {}^3\Delta_u$	$1\ {}^3A_u$	$2\sigma_u^+$	$3\sigma_g^+$	2.01	−49.156269	1.71	−49.151459	1.70	−49.153473	1.69	−49.133997	1.69
B_2	$1\ {}^3\Delta_u$	$1\ {}^3B_{1u}$	$2\sigma_u^+$	$3\sigma_g^+$	3.86	−49.156269	1.71	−49.151508	1.70	−49.153555	1.68	−49.133931	1.69
B_2	$1\ {}^1\Pi_u$	$1\ {}^1B_{3u}$	$1\pi_u$	$3\sigma_g^+$	2.15	−49.148224	1.92	−49.141836	1.96	−49.143486	1.96	−49.118092	2.12
B_2	$1\ {}^3\Sigma_u^+$	$2\ {}^3B_{1u}$	$2\sigma_u^+$	$3\sigma_g^+$	2.01	−49.146652	1.97	−49.141673	1.97	−49.143956	1.95	−49.122506	2.00
B_2	$1\ {}^3\Sigma_u^-$	$2\ {}^3A_u$	$2\sigma_u^+$	$3\sigma_g^+$	3.84	−49.146202	1.98	−49.140214	2.01	−49.142826	1.98	−49.117661	2.13
B_2	$1\ {}^3\Pi_g$	$1\ {}^3B_{3g}$	$2\sigma_u^+$	$1\pi_u$	2.14	−49.137567	2.21	−49.132271	2.22	−49.133895	2.22	−49.112704	2.27
B_2	$2\ {}^1\Sigma_g^+$	$3\ {}^1A_g$	$1\pi_u 1\pi_u$	$3\sigma_g^+ 3\sigma_g^+$	0.28	−49.125735	2.54	−49.118656	2.59	−49.119352	2.62	−49.096758	2.70
B_2	$2\ {}^3\Pi_g$	$2\ {}^3B_{3g}$	$2\sigma_u^+ 1\pi_u$	$3\sigma_g^+ 3\sigma_g^+$	2.02	−49.104872	3.10	−49.099113	3.12	−49.101037	3.11	−49.078565	3.20
B_2	$1\ {}^1\Pi_g$	$1\ {}^1B_{3g}$	$2\sigma_u^+$	$1\pi_u$	2.17	−49.089482	3.52	−49.084055	3.53	−49.085593	3.53	−49.065529	3.55
C_2	$1\ {}^1\Sigma_g^+$	$1\ {}^1A_g$	ground state		0.39	−75.640639	0.00	−75.634133	0.00	−75.636007	0.00	−75.592449	0.00
C_2	$1\ {}^3\Pi_u$	$1\ {}^3B_{3u}$	$1\pi_u$	$3\sigma_g^+$	2.08	−75.610484	0.82	−75.601912	0.88	−75.603905	0.87	−75.562567	0.81
C_2	$1\ {}^3\Sigma_u^+$	$1\ {}^3B_{1u}$	$2\sigma_u^+$	$3\sigma_g^+$	2.09	−75.599259	1.13	−75.592370	1.14	−75.594630	1.13	−75.556431	0.98

Table S4: . . . continued

Molecule	State	Irrep	Transition		NOS	E_{FCI}	V_{FCI}	E_{IPEA}	V_{IPEA}	E_{NOIPEA}	V_{NOIPEA}	E_{CASSCF}	V_{CASSCF}
C_2	$1\,^1\Pi_u$	$1\,^1B_{3u}$	$1\pi_u$	$3\sigma_g^+$	2.10	−75.560604	2.18	−75.551441	2.25	−75.553373	2.25	−75.508413	2.29
C_2	$1\,^3\Sigma_g^-$	$1\,^3B_{1g}$	$1\pi_u 1\pi_u$	$3\sigma_g^+ 3\sigma_g^+$	2.04	−75.549347	2.48	−75.539666	2.57	−75.541060	2.58	−75.499408	2.53
C_2	$1\,^3\Pi_g$	$1\,^3B_{2g}$	$2\sigma_u^+ 1\pi_u$	$3\sigma_g^+ 3\sigma_g^+$	2.13	−75.529837	3.02	−75.522060	3.05	−75.524241	3.04	−75.482934	2.98
C_2	$2\,^1\Sigma_g^+$	$2\,^1A_g$	$1\pi_u 1\pi_u$	$3\sigma_g^+ 3\sigma_g^+$	0.20	−75.519913	3.29	−75.511146	3.35	−75.512630	3.36	−75.471243	3.30
C_2	$1\,^1\Delta_g$	$3\,^1A_g$	$1\pi_u 1\pi_u$	$3\sigma_g^+ 3\sigma_g^+$	0.20	−75.515287	3.41	−75.505881	3.49	−75.512630	3.36	−75.466282	3.43
C_2	$1\,^1\Delta_g$	$1\,^1B_{1g}$	$1\pi_u 1\pi_u$	$3\sigma_g^+ 3\sigma_g^+$	2.04	−75.515287	3.41	−75.506304	3.48	−75.507558	3.50	−75.467256	3.41
C_2	$1\,^5\Sigma_g^+$	$1\,^5A_g$	$2\sigma_u^+ 1\pi_u$	$3\sigma_g^+ 1\pi_g$	4.00	−75.451023	5.16	−75.445970	5.12	−75.449312	5.08	−75.405182	5.10
C_2	$1\,^1\Pi_g$	$1\,^1B_{2g}$	$2\sigma_u^+ 1\pi_u$	$3\sigma_g^+ 3\sigma_g^+$	2.25	−75.439775	5.47	−75.430134	5.55	−75.432500	5.54	−75.385622	5.63
C_2	$1\,^5\Pi_g$	$1\,^5B_{2g}$	$1\pi_u 1\pi_u$	$3\sigma_g^+ 1\pi_g$	4.01	−75.407262	6.35	−75.398560	6.41	−75.401470	6.38	−75.356992	6.41
N_2	$1\,^1\Sigma_g^+$	$1\,^1A_g$	ground state		0.13	−109.102922	0.00	−109.092001	0.00	−109.092717	0.00	−109.028519	0.00
N_2	$1\,^3\Sigma_u^+$	$1\,^3B_{1u}$	$1\pi_u$	$1\pi_g$	2.01	−108.816185	7.80	−108.805864	7.79	−108.808646	7.73	−108.728625	8.16
N_2	$1\,^3\Pi_g$	$1\,^3B_{2g}$	$3\sigma_g^+$	$1\pi_g$	2.08	−108.812942	7.89	−108.801750	7.90	−108.804657	7.84	−108.722200	8.34
N_2	$1\,^1\Pi_g$	$1\,^1B_{2g}$	$3\sigma_g^+$	$1\pi_g$	2.06	−108.766595	9.15	−108.755577	9.15	−108.758207	9.10	−108.676142	9.59
N_2	$1\,^3\Delta_u$	$1\,^3A_u$	$1\pi_u$	$1\pi_g$	2.01	−108.761260	9.30	−108.750665	9.29	−108.753634	9.23	−108.672268	9.69
N_2	$1\,^3\Delta_u$	$2\,^3B_{1u}$	$1\pi_u$	$1\pi_g$	2.00	−108.761260	9.30	−108.750779	9.29	−108.753639	9.23	−108.672355	9.69
N_2	$1\,^3\Sigma_u^-$	$2\,^3A_u$	$1\pi_u$	$1\pi_g$	2.04	−108.739696	9.88	−108.729194	9.87	−108.732523	9.80	−108.647731	10.36
N_2	$1\,^1\Sigma_u^-$	$1\,^1A_u$	$1\pi_u$	$1\pi_g$	2.00	−108.713806	10.59	−108.703798	10.56	−108.706473	10.51	−108.623284	11.03
N_2	$1\,^1\Delta_u$	$1\,^1B_{1u}$	$1\pi_u$	$1\pi_g$	2.00	−108.704655	10.84	−108.694087	10.83	−108.697670	10.75	−108.611938	11.34
N_2	$1\,^1\Delta_u$	$2\,^1A_u$	$1\pi_u$	$1\pi_g$	2.04	−108.704655	10.84	−108.694600	10.81	−108.697646	10.75	−108.611881	11.34
N_2	$1\,^3\Pi_u$	$1\,^3B_{3u}$	$2\sigma_u^+$	$1\pi_g$	2.16	−108.698048	11.02	−108.687285	11.01	−108.690529	10.94	−108.605983	11.50
N_2	$1\,^1\Pi_u$	$1\,^1B_{3u}$	$2\sigma_u^+$	$1\pi_g$	2.17	−108.608772	13.45	−108.597230	13.46	−108.600611	13.39	−108.516990	13.92
H_2O		$1\,^1A_1$	ground state		0.04	−76.119893	0.00	−76.110372	0.00	−76.110827	0.00	−76.012369	0.00
H_2O		$1\,^3B_1$	$1b_1$	$4a_1$	2.00	−75.834513	7.77	−75.825716	7.75	−75.827727	7.70	−75.746831	7.23
H_2O		$1\,^1B_1$	$1b_1$	$4a_1$	2.01	−75.807603	8.50	−75.799512	8.46	−75.801259	8.42	−75.721785	7.91
H_2O		$1\,^3A_1$	$3a_1$	$4a_1$	2.00	−75.752968	9.98	−75.744456	9.96	−75.746406	9.92	−75.665945	9.43

Table S4: ... continued

Molecule	State	Irrep	Transition		NOS	E_{FCI}	V_{FCI}	E_{IPEA}	V_{IPEA}	E_{NOIPEA}	V_{NOIPEA}	E_{CASSCF}	V_{CASSCF}
H_2O		$1\,^3A_2$	$1b_1$	$2b_2$	2.01	−75.743574	10.24	−75.734894	10.22	−75.736911	10.17	−75.655790	9.70
H_2O		$1\,^1A_2$	$1b_1$	$2b_2$	2.01	−75.725081	10.74	−75.717470	10.69	−75.719283	10.65	−75.640186	10.13
H_2O		$2\,^1A_1$	$3a_1$	$4a_1$	2.00	−75.715234	11.01	−75.709901	10.90	−75.712266	10.85	−75.617344	10.75
H_2O		$1\,^3B_2$	$3a_1$	$2b_2$	2.00	−75.674139	12.13	−75.667456	12.05	−75.669851	12.00	−75.575292	11.89
H_2O		$1\,^1B_2$	$3a_1$	$2b_2$	2.00	−75.626531	13.43	−75.619074	13.37	−75.620911	13.33	−75.534493	13.00
H_2O		$2\,^3B_2$	$1b_2$	$4a_1$	2.00	−75.603947	14.04	−75.596684	13.98	−75.599357	13.92	−75.503402	13.85
CH_2		$1\,^3B_2$	ground state		2.03	−38.964139	0.00	−38.956184	0.00	−38.957525	0.00	−38.919137	0.00
CH_2		$1\,^1A_1$	$1b_2$	$3a_1$	0.04	−38.942886	0.58	−38.933003	0.63	−38.933498	0.65	−38.894310	0.68
CH_2		$1\,^1B_2$	ground state		2.03	−38.879494	2.30	−38.870200	2.34	−38.871996	2.33	−38.828327	2.47
CH_2		$2\,^1A_1$	$3a_1$	$1b_2$	0.06	−38.779344	5.03	−38.768952	5.09	−38.769562	5.11	−38.727271	5.22
CH_2		$1\,^3A_2$	$1b_1$	$3a_1$	2.05	−38.748964	5.86	−38.740127	5.88	−38.741715	5.87	−38.699166	5.99
CH_2		$1\,^1A_2$	$1b_1$	$3a_1$	2.05	−38.723281	6.55	−38.715127	6.56	−38.716832	6.55	−38.673027	6.70
CH_2		$1\,^3B_1$	$1b_1$	$1b_2$	2.01	−38.696464	7.28	−38.686841	7.33	−38.688812	7.31	−38.637600	7.66
CH_2		$1\,^3A_1$	$1b_1 1b_2$	$3a_1 4a_1$	2.01	−38.649015	8.57	−38.640651	8.59	−38.642379	8.58	−38.607556	8.48
CH_2		$1\,^1B_1$	$1b_1$	$1b_2$	2.01	−38.640493	8.81	−38.631941	8.82	−38.633787	8.81	−38.587560	9.02
CH_2		$3\,^1A_1$	$3a_1$	$4a_1$	2.00	−38.622397	9.30	−38.613429	9.33	−38.615343	9.31	−38.572392	9.44
CH_2		$2\,^3B_1$	$1b_2$	$2b_1$	2.00	−38.620846	9.34	−38.611692	9.37	−38.613568	9.36	−38.570238	9.49
CH_2		$2\,^3B_2$	$3a_1$	$4a_1$	2.14	−38.569633	10.74	−38.559945	10.78	−38.562591	10.75	−38.521513	10.82
CH_2		$1\,^5A_2$	$1b_1$	$4a_1$	4.00	−38.563825	10.89	−38.557907	10.84	−38.560582	10.80	−38.525081	10.72
CH_2		$2\,^1B_1$	$1b_2$	$2b_1$	2.01	−38.553940	11.16	−38.544686	11.20	−38.546592	11.18	−38.504755	11.28
CH_2		$1\,^5B_2$	$1b_1$	$2b_1$	4.01	−38.551050	11.24	−38.545713	11.17	−38.548158	11.14	−38.515865	10.97
CH_2		$2\,^3A_2$	$3a_1$	$2b_1$	2.13	−38.536589	11.63	−38.523449	11.78	−38.525541	11.75	−38.482323	11.89
CH_2		$2\,^1B_2$	$3a_1$	$4a_1$	2.10	−38.535515	11.66	−38.526377	11.70	−38.528615	11.67	−38.488596	11.72
CH_2		$2\,^1A_2$	$3a_1$	$2b_1$	2.08	−38.516084	12.19	−38.503312	12.32	−38.505157	12.31	−38.462935	12.41
CH_2		$3\,^3B_1$	$1b_1 1b_2$	$3a_1 4a_1$	2.01	−38.510059	12.36	−38.499783	12.42	−38.502429	12.38	−38.456832	12.58

Table S5: Total energies E_{X} in a.u. and vertical excitation energies V_X in eV of di- and triatomic molecules (X = FCI, IPEA, NOIPEA, CASSCF) calculated using the 6-311G basis set.

**Molecule**	**State**	**Irrep**	**Transition**		**NOS**	$\mathbf{E}_{\mathrm{FCI}}$	$\mathbf{V}_{\mathrm{FCI}}$	$\mathbf{E}_{\mathrm{IPEA}}$	$\mathbf{V}_{\mathrm{IPEA}}$	$\mathbf{E}_{\mathrm{NOIPEA}}$	$\mathbf{V}_{\mathrm{NOIPEA}}$	$\mathbf{E}_{\mathrm{CASSCF}}$	$\mathbf{V}_{\mathrm{CASSCF}}$
HLi	$1\,^1\Sigma^+$	$1\,^1A_1$	ground state		0.22	−8.019525	0.00	−8.008866	0.00	−8.009380	0.00	−7.976628	0.00
HLi	$1\,^3\Sigma^+$	$1\,^3A_1$	$2\sigma^+$	$3\sigma^+$	2.00	−7.915866	2.82	−7.913407	2.60	−7.913554	2.61	−7.900877	2.06
HLi	$2\,^1\Sigma^+$	$2\,^1A_1$	$2\sigma^+$	$3\sigma^+$	1.88	−7.900887	3.23	−7.896243	3.06	−7.896729	3.07	−7.873119	2.82
HLi	$1\,^3\Pi$	$1\,^3B_1$	$2\sigma^+$	1π	2.00	−7.874773	3.94	−7.874589	3.65	−7.874662	3.67	−7.873458	2.81
HLi	$1\,^1\Pi$	$1\,^1B_1$	$2\sigma^+$	1π	2.00	−7.863997	4.23	−7.863756	3.95	−7.863884	3.96	−7.862313	3.11
HLi	$2\,^3\Sigma^+$	$2\,^3A_1$	$2\sigma^+$	$4\sigma^+$	2.00	−7.819749	5.44	−7.817281	5.21	−7.817443	5.22	−7.804795	4.68
HLi	$3\,^1\Sigma^+$	$3\,^1A_1$	$2\sigma^+$	$4\sigma^+$	1.90	−7.797155	6.05	−7.792357	5.89	−7.792827	5.89	−7.771721	5.58
HBe	$1\,^2\Sigma^+$	$1\,^2A_1$	ground state		1.00	−15.188912	0.00	−15.181397	0.00	−15.182568	0.00	−15.148608	0.00
HBe	$1\,^2\Pi$	$1\,^2B_1$	$3\sigma^+$	1π	1.37	−15.094608	2.57	−15.090732	2.47	−15.091060	2.49	−15.071210	2.11
HBe	$2\,^2\Sigma^+$	$2\,^2A_1$	$2\sigma^+$	$3\sigma^+$	1.02	−14.987453	5.48	−14.982360	5.42	−14.986846	5.33	−14.946673	5.49
HBe	$1\,^4\Pi$	$1\,^4B_1$	$2\sigma^+$	1π	3.00	−14.979083	5.71	−14.977960	5.54	−14.978499	5.55	−14.974104	4.75
HBe	$2\,^2\Pi$	$2\,^2B_1$	$2\sigma^+$	1π	2.65	−14.910144	7.59	−14.905959	7.50	−14.907101	7.50	−14.885880	7.15
HBe	$1\,^4\Sigma^-$	$1\,^4A_2$	$2\sigma^+3\sigma^+$	$1\pi1\pi$	1.01	−14.919174	7.34	−14.907424	7.46	−14.909723	7.42	−14.871138	7.55
HBe	$3\,^2\Sigma^+$	$3\,^2A_1$	$3\sigma^+$	$4\sigma^+$	3.00	−14.881553	8.36	−14.879776	8.21	−14.880689	8.21	−14.874441	7.46
HB	$1\,^1\Sigma^+$	$1\,^1A_1$	ground state		0.11	−25.199770	0.00	−25.189169	0.00	−25.189772	0.00	−25.147097	0.00
HB	$1\,^3\Pi$	$1\,^3B_1$	$3\sigma^+$	1π	2.02	−25.159726	1.09	−25.154208	0.95	−25.155051	0.94	−25.124549	0.61
HB	$1\,^1\Pi$	$1\,^1B_1$	$3\sigma^+$	1π	2.05	−25.088064	3.04	−25.080915	2.95	−25.082466	2.92	−25.043467	2.82
HB	$1\,^3\Sigma^-$	$1\,^3A_2$	$3\sigma^+3\sigma^+$	$1\pi1\pi$	2.15	−25.038621	4.39	−25.033999	4.22	−25.035138	4.21	−25.007077	3.81
HB	$1\,^1\Delta$	$1\,^1A_2$	$3\sigma^+3\sigma^+$	$1\pi1\pi$	2.00	−24.970999	6.23	−24.964122	6.12	−24.965761	6.10	−24.934039	5.80
HB	$1\,^1\Delta$	$2\,^1A_1$	$3\sigma^+3\sigma^+$	$1\pi1\pi$	0.00	−24.970999	6.23	−24.960390	6.23	−24.961733	6.21	−24.919478	6.19
HB	$1\,^5\Sigma^-$	$1\,^5A_2$	$2\sigma^+3\sigma^+$	$1\pi1\pi$	0.01	−24.931300	7.31	−24.928980	7.08	−24.930573	7.05	−24.916775	6.27
HB	$2\,^1\Sigma^+$	$3\,^1A_1$	$3\sigma^+3\sigma^+$	$1\pi1\pi$	4.00	−24.936058	7.18	−24.923778	7.22	−24.925952	7.18	−24.874066	7.43
HB	$1\,^3\Sigma^+$	$1\,^3A_1$	$3\sigma^+$	$4\sigma^+$	2.00	−24.929269	7.36	−24.921568	7.28	−24.923300	7.25	−24.889724	7.00
HB	$2\,^3\Pi$	$2\,^3B_1$	$2\sigma^+$	1π	2.03	−24.900487	8.14	−24.894290	8.02	−24.896148	7.99	−24.862312	7.75

Table S5: … continued

Molecule	State	Irrep	Transition		NOS	E_{FCI}	V_{FCI}	E_{IPEA}	V_{IPEA}	E_{NOIPEA}	V_{NOIPEA}	E_{CASSCF}	V_{CASSCF}
HB	3 $^1\Sigma^+$	4 $^1A_1$	$3\sigma^+$	$4\sigma^+$	1.91	−24.883969	8.59	−24.872520	8.62	−24.875171	8.56	−24.818320	8.95
HB	2 $^1\Pi$	2 $^1B_1$	$2\sigma^+$	1π	2.02	−24.815943	10.44	−24.806707	10.41	−24.811981	10.28	−24.765031	10.40
HC	1 $^2\Pi$	1 $^2B_1$	ground state		1.07	−38.349309	0.00	−38.336003	0.00	−38.337317	0.00	−38.275819	0.00
HC	1 $^4\Sigma^-$	1 $^4A_2$	$3\sigma^+$	1π	3.04	−38.342789	0.18	−38.336576	−0.02	−38.338433	−0.03	−38.292475	−0.45
HC	1 $^2\Delta$	1 $^2A_1$	$3\sigma^+$	1π	1.02	−38.236552	3.07	−38.226589	2.98	−38.229150	2.94	−38.170730	2.86
HC	1 $^2\Delta$	1 $^2A_2$	$3\sigma^+$	1π	3.02	−38.236552	3.07	−38.225745	3.00	−38.228600	2.96	−38.168457	2.92
HC	1 $^2\Sigma^-$	2 $^2A_2$	$3\sigma^+$	1π	3.04	−38.226197	3.35	−38.212932	3.35	−38.215508	3.31	−38.154477	3.30
HC	1 $^2\Sigma^+$	2 $^2A_1$	$3\sigma^+$	1π	1.02	−38.206148	3.90	−38.195205	3.83	−38.198482	3.78	−38.133316	3.88
HC	2 $^2\Pi$	2 $^2B_2$	$3\sigma^+3\sigma^+$	$1\pi1\pi$	1.02	−38.088072	7.11	−38.071696	7.19	−38.074576	7.15	−38.011136	7.20
HC	2 $^2\Sigma^+$	3 $^2A_1$	1π	$4\sigma^+$	1.05	−38.067742	7.66	−38.053877	7.68	−38.055308	7.67	−37.990502	7.76
HC	1 $^4\Pi$	1 $^4B_1$	$3\sigma^+$	$4\sigma^+$	3.00	−38.062593	7.80	−38.052218	7.72	−38.055270	7.67	−37.999093	7.53
HC	2 $^4\Sigma^-$	2 $^4A_2$	$2\sigma^+$	1π	2.97	−38.019818	8.97	−38.010526	8.86	−38.016315	8.73	−37.949519	8.88
HC	3 $^2\Pi$	3 $^2B_1$	$3\sigma^+$	$4\sigma^+$	3.00	−38.019341	8.98	−38.012340	8.81	−38.014605	8.78	−37.965427	8.45
HC	4 $^2\Pi$	4 $^2B_1$	$3\sigma^+$	$4\sigma^+$	2.98	−37.975552	10.17	−37.964508	10.11	−37.970366	9.99	−37.897205	10.30
HC	2 $^2\Delta$	3 $^2A_2$	$2\sigma^+$	1π	3.10	−37.900296	12.22	−37.884264	12.29	−37.889596	12.18	−37.827281	12.21
HC	2 $^2\Delta$	4 $^2A_1$	$2\sigma^+$	1π	1.03	−37.899935	12.23	−37.889291	12.16	−37.894467	12.05	−37.828648	12.17
HC	2 $^4\Pi$	2 $^4B_1$	$2\sigma^+3\sigma^+$	$1\pi1\pi$	3.00	−37.894545	12.37	−37.875407	12.53	−37.878097	12.50	−37.812894	12.60
HN	1 $^3\Sigma^-$	1 $^3A_2$	ground state		2.07	−55.050698	0.00	−55.035506	0.00	−55.037980	0.00	−54.947422	0.00
HN	1 $^1\Delta$	1 $^1A_1$	ground state		0.01	−54.978984	1.95	−54.967201	1.86	−54.969088	1.87	−54.884667	1.71
HN	1 $^1\Delta$	1 $^1A_2$	ground state		2.05	−54.978984	1.95	−54.964735	1.93	−54.967082	1.93	−54.877093	1.91
HN	1 $^1\Sigma^+$	2 $^1A_1$	ground state		0.04	−54.951915	2.69	−54.940357	2.59	−54.942677	2.59	−54.853899	2.54
HN	1 $^3\Pi$	1 $^3B_1$	$3\sigma^+$	1π	2.01	−54.912005	3.77	−54.900307	3.68	−54.902356	3.69	−54.818309	3.51
HN	1 $^1\Pi$	1 $^1B_2$	$3\sigma^+$	1π	2.02	−54.833399	5.91	−54.819617	5.87	−54.822150	5.87	−54.725812	6.03
HN	1 $^5\Sigma^-$	1 $^5A_2$	$3\sigma^+$	$4\sigma^+$	4.00	−54.743934	8.35	−54.734885	8.18	−54.737534	8.18	−54.675175	7.41
HN	2 $^3\Pi$	2 $^3B_2$	1π	$4\sigma^+$	2.01	−54.721808	8.95	−54.706648	8.95	−54.709494	8.94	−54.632579	8.57

Table S5: … continued

Molecule	State	Irrep	Transition		NOS	E_{FCI}	V_{FCI}	E_{IPEA}	V_{IPEA}	E_{NOIPEA}	V_{NOIPEA}	E_{CASSCF}	V_{CASSCF}
HN	$2\,^1\Pi$	$2\,^1B_1$	1π	$4\sigma^+$	2.01	−54.701452	9.50	−54.690247	9.39	−54.692066	9.41	−54.619553	8.92
HN	$2\,^3\Sigma^-$	$2\,^3A_2$	$3\sigma^+$	$4\sigma^+$	3.97	−54.688470	9.86	−54.683484	9.58	−54.689662	9.48	−54.603467	9.36
HN	$2\,^1\Sigma^+$	$3\,^1A_1$	$3\sigma^+3\sigma^+$	$1\pi1\pi$	0.00	−54.637114	11.25	−54.608181	11.63	−54.608771	11.68	−54.535435	11.21
HN	$1\,^3\Delta$	$3\,^3A_2$	$3\sigma^+$	$4\sigma^+$	2.00	−54.616175	11.82	−54.606128	11.68	−54.609005	11.67	−54.540362	11.08
HN	$1\,^3\Delta$	$1\,^3A_1$	$3\sigma^+$	$4\sigma^+$	4.00	−54.616175	11.82	−54.607636	11.64	−54.610523	11.63	−54.544474	10.96
HN	$1\,^1\Sigma^-$	$2\,^1A_2$	$3\sigma^+$	$4\sigma^+$	3.99	−54.599066	12.29	−54.588597	12.16	−54.592179	12.13	−54.515630	11.75
HN	$3\,^3\Sigma^-$	$4\,^3A_2$	$3\sigma^+$	$4\sigma^+$	4.00	−54.608475	12.03	−54.600166	11.85	−54.606414	11.74	−54.512218	11.84
HN	$1\,^3\Sigma^+$	$2\,^3A_1$	$3\sigma^+$	$4\sigma^+$	2.00	−54.582021	12.75	−54.572588	12.60	−54.576404	12.56	−54.506728	11.99
HN	$2\,^1\Delta$	$4\,^1A_1$	$3\sigma^+$	$4\sigma^+$	1.97	−54.564507	13.23	−54.562388	12.87	−54.568394	12.78	−54.455647	13.38
HN	$2\,^1\Delta$	$3\,^1A_2$	$3\sigma^+$	$4\sigma^+$	4.00	−54.564507	13.23	−54.554585	13.09	−54.560051	13.01	−54.467007	13.07
HN	$3\,^3\Pi$	$3\,^3B_2$	$2\sigma^+$	1π	2.05	−54.507656	14.78	−54.488045	14.90	−54.492786	14.84	−54.404541	14.77
HO	$1\,^2\Pi$	$1\,^2B_1$	ground state		1.05	−75.513547	0.00	−75.500605	0.00	−75.502476	0.00	−75.377434	0.00
HO	$1\,^2\Sigma^+$	$1\,^2A_1$	$3\sigma^+$	1π	1.01	−75.356521	4.27	−75.344103	4.26	−75.345906	4.26	−75.214952	4.42
HO	$1\,^4\Sigma^-$	$1\,^4A_2$	1π	$4\sigma^+$	3.00	−75.244126	7.33	−75.234490	7.24	−75.236625	7.23	−75.148526	6.23
HO	$1\,^2\Sigma^-$	$1\,^2A_2$	1π	$4\sigma^+$	3.00	−75.213407	8.17	−75.203770	8.08	−75.205994	8.07	−75.119931	7.01
HO	$1\,^2\Delta$	$2\,^2A_2$	1π	$4\sigma^+$	1.00	−75.144838	10.03	−75.134241	9.97	−75.136025	9.97	−75.051331	8.87
HO	$1\,^2\Delta$	$2\,^2A_1$	1π	$4\sigma^+$	3.00	−75.144838	10.03	−75.138889	9.84	−75.141274	9.83	−75.043344	9.09
HO	$1\,^4\Pi$	$1\,^4B_1$	$3\sigma^+$	$4\sigma^+$	3.00	−75.119819	10.71	−75.110659	10.61	−75.112770	10.60	−75.026355	9.55
HO	$2\,^2\Sigma^+$	$3\,^2A_1$	1π	$4\sigma^+$	1.01	−75.107689	11.04	−75.100926	10.88	−75.103839	10.85	−75.004859	10.14
HO	$2\,^2\Pi$	$2\,^2B_1$	$3\sigma^+$	$4\sigma^+$	2.97	−75.064207	12.23	−75.060923	11.96	−75.066276	11.87	−74.953524	11.54
HO	$3\,^2\Pi$	$3\,^2B_2$	$3\sigma^+$	$4\sigma^+$	2.98	−75.011088	13.67	−75.004930	13.49	−75.010699	13.38	−74.889308	13.28
HF	$1\,^1\Sigma^+$	$1\,^1A_1$	ground state		0.04	−100.184748	0.00	−100.177462	0.00	−100.178190	0.00	−100.012638	0.00
HF	$1\,^3\Pi$	$1\,^3B_1$	1π	$4\sigma^+$	2.00	−99.823124	9.84	−99.816575	9.82	−99.817932	9.80	−99.701075	8.48
HF	$1\,^1\Pi$	$1\,^1B_1$	1π	$4\sigma^+$	2.00	−99.802707	10.40	−99.796210	10.37	−99.797337	10.36	−99.682561	8.98
HF	$1\,^3\Sigma^+$	$1\,^3A_1$	$3\sigma^+$	$4\sigma^+$	2.00	−99.700137	13.19	−99.693806	13.16	−99.695076	13.15	−99.579371	11.79

Table S5: ... continued

**Molecule**	**State**	**Irrep**	**Transition**		**NOS**	$\mathbf{E_{FCI}}$	$\mathbf{V_{FCI}}$	$\mathbf{E_{IPEA}}$	$\mathbf{V_{IPEA}}$	$\mathbf{E_{NOIPEA}}$	$\mathbf{V_{NOIPEA}}$	$\mathbf{E_{CASSCF}}$	$\mathbf{V_{CASSCF}}$
HF	$2\ ^1\Sigma^+$	$2\ ^1A_1$	$3\sigma^+$	$4\sigma^+$	1.96	−99.631024	15.07	−99.634277	14.78	−99.637086	14.72	−99.476288	14.59
Li_2	$1\ ^1\Sigma_g^+$	$1\ ^1A_g$	ground state		0.05	−14.896800	0.00	−14.895173	0.00	−14.895293	0.00	−14.893272	0.00
Li_2	$1\ ^3\Sigma_u^+$	$1\ ^3B_{1u}$	$2\sigma_g^+$	$2\sigma_u^+$	2.00	−14.851911	1.22	−14.851897	1.18	−14.851902	1.18	−14.851853	1.13
Li_2	$1\ ^3\Pi_u$	$1\ ^3B_{3u}$	$2\sigma_g^+$	$1\pi_u$	2.00	−14.845424	1.40	−14.845112	1.36	−14.845235	1.36	−14.844494	1.33
Li_2	$1\ ^1\Sigma_u^+$	$1\ ^1B_{1u}$	$2\sigma_g^+$	$2\sigma_u^+$	2.00	−14.831347	1.78	−14.831320	1.74	−14.831333	1.74	−14.831219	1.69
Li_2	$2\ ^1\Sigma_g^+$	$2\ ^1A_g$	$2\sigma_g^+$	$3\sigma_g^+$	1.52	−14.794525	2.78	−14.793932	2.75	−14.794257	2.75	−14.791643	2.77
Li_2	$1\ ^1\Pi_u$	$1\ ^1B_{3u}$	$2\sigma_g^+$	$1\pi_u$	2.00	−14.795177	2.77	−14.794727	2.73	−14.794841	2.73	−14.794168	2.70
B_2	$1\ ^3\Sigma_g^-$	$1\ ^3B_{1g}$	ground state		2.13	−49.232489	0.00	−49.226833	0.00	−49.228720	0.00	−49.205717	0.00
B_2	$1\ ^5\Sigma_u^-$	$1\ ^5A_u$	$2\sigma_u^+$	$3\sigma_g^+$	4.00	−49.232036	0.01	−49.227606	−0.02	−49.229311	−0.02	−49.209203	−0.09
B_2	$1\ ^1\Delta_g$	$1\ ^1A_g$	ground state		2.12	−49.206820	0.70	−49.200764	0.71	−49.202676	0.71	−49.177326	0.77
B_2	$1\ ^1\Delta_g$	$1\ ^1B_{1g}$	ground state		0.25	−49.206820	0.70	−49.201507	0.69	−49.203347	0.69	−49.181033	0.67
B_2	$1\ ^3\Pi_u$	$1\ ^3B_{2u}$	$1\pi_u$	$3\sigma_g^+$	2.09	−49.204744	0.75	−49.197420	0.80	−49.199513	0.79	−49.173085	0.89
B_2	$1\ ^1\Sigma_g^+$	$2\ ^1A_g$	ground state		0.26	−49.199845	0.89	−49.193755	0.90	−49.196003	0.89	−49.168967	1.00
B_2	$1\ ^3\Delta_u$	$1\ ^3A_u$	$2\sigma_u^+$	$3\sigma_g^+$	3.87	−49.172011	1.65	−49.166385	1.64	−49.169094	1.62	−49.144988	1.65
B_2	$1\ ^3\Delta_u$	$1\ ^3B_{1u}$	$2\sigma_u^+$	$3\sigma_g^+$	2.01	−49.172011	1.65	−49.166321	1.65	−49.168966	1.63	−49.145086	1.65
B_2	$1\ ^1\Pi_u$	$1\ ^1B_{3u}$	$1\pi_u$	$3\sigma_g^+$	2.15	−49.168925	1.73	−49.161183	1.79	−49.163288	1.78	−49.132370	2.00
B_2	$1\ ^3\Sigma_u^+$	$2\ ^3B_{1u}$	$2\sigma_u^+$	$3\sigma_g^+$	2.01	−49.163055	1.89	−49.157243	1.89	−49.160253	1.86	−49.133952	1.95
B_2	$1\ ^3\Sigma_u^-$	$2\ ^3A_u$	$2\sigma_u^+$	$3\sigma_g^+$	3.84	−49.162030	1.92	−49.155022	1.95	−49.158414	1.91	−49.128475	2.10
B_2	$1\ ^3\Pi_g$	$1\ ^3B_{3g}$	$2\sigma_u^+$	$1\pi_u$	2.13	−49.153676	2.14	−49.147536	2.16	−49.149724	2.15	−49.123749	2.23
B_2	$2\ ^1\Sigma_g^+$	$3\ ^1A_g$	$1\pi_u 1\pi_u$	$3\sigma_g^+ 3\sigma_g^+$	0.27	−49.151624	2.20	−49.142402	2.30	−49.143306	2.32	−49.114850	2.47
B_2	$2\ ^3\Pi_g$	$2\ ^3B_{3g}$	$2\sigma_u^+ 1\pi_u$	$3\sigma_g^+ 3\sigma_g^+$	2.02	−49.125985	2.90	−49.118925	2.94	−49.121632	2.91	−49.092925	3.07
B_2	$1\ ^1\Pi_g$	$1\ ^1B_{3g}$	$2\sigma_u^+$	$1\pi_u$	2.16	−49.105724	3.45	−49.099466	3.47	−49.101408	3.46	−49.077355	3.49
C_2	$1\ ^1\Sigma_g^+$	$1\ ^1A_g$	ground state		0.39	−75.663343	0.00	−75.656204	0.00	−75.658234	0.00	−75.605670	0.00
C_2	$1\ ^3\Pi_u$	$1\ ^3B_{3u}$	$1\pi_u$	$3\sigma_g^+$	2.08	−75.636970	0.72	−75.626982	0.80	−75.629364	0.79	−75.578616	0.74
C_2	$1\ ^3\Sigma_u^+$	$1\ ^3B_{1u}$	$2\sigma_u^+$	$3\sigma_g^+$	2.09	−75.623521	1.08	−75.615851	1.10	−75.618458	1.08	−75.572586	0.90

Table S5: . . . continued

Molecule	State	Irrep	Transition		NOS	E_{FCI}	V_{FCI}	E_{IPEA}	V_{IPEA}	E_{NOIPEA}	V_{NOIPEA}	E_{CASSCF}	V_{CASSCF}
C_2	$1\,^1\Pi_u$	$1\,^1B_{3u}$	$1\pi_u$	$3\sigma_g^+$	2.10	−75.588328	2.04	−75.577725	2.14	−75.579989	2.13	−75.525050	2.19
C_2	$1\,^3\Sigma_g^-$	$1\,^3B_{1g}$	$1\pi_u 1\pi_u$	$3\sigma_g^+ 3\sigma_g^+$	2.04	−75.580534	2.25	−75.569208	2.37	−75.570831	2.38	−75.519233	2.35
C_2	$1\,^3\Pi_g$	$1\,^3B_{2g}$	$2\sigma_u^+ 1\pi_u$	$3\sigma_g^+ 3\sigma_g^+$	2.13	−75.559146	2.84	−75.549956	2.89	−75.552596	2.87	−75.501314	2.84
C_2	$2\,^1\Sigma_g^+$	$2\,^1A_g$	$1\pi_u 1\pi_u$	$3\sigma_g^+ 3\sigma_g^+$	0.20	−75.551164	3.05	−75.540804	3.14	−75.542960	3.14	−75.491196	3.11
C_2	$1\,^1\Delta_g$	$3\,^1A_g$	$1\pi_u 1\pi_u$	$3\sigma_g^+ 3\sigma_g^+$	0.05	−75.546792	3.17	−75.535610	3.28	−75.537095	3.30	−75.485943	3.26
C_2	$1\,^1\Delta_g$	$1\,^1B_{1g}$	$1\pi_u 1\pi_u$	$3\sigma_g^+ 3\sigma_g^+$	2.04	−75.546792	3.17	−75.536225	3.26	−75.537705	3.28	−75.487297	3.22
C_2	$1\,^5\Sigma_g^+$	$1\,^5A_g$	$2\sigma_u^+ 1\pi_u$	$3\sigma_g^+ 1\pi_g$	4.00	−75.474912	5.13	−75.469035	5.09	−75.472885	5.04	−75.420502	5.04
C_2	$1\,^1\Pi_g$	$1\,^1B_{2g}$	$2\sigma_u^+ 1\pi_u$	$3\sigma_g^+ 3\sigma_g^+$	2.23	−75.470425	5.25	−75.459037	5.37	−75.461867	5.34	−75.404184	5.48
C_2	$1\,^5\Pi_g$	$1\,^5B_{2g}$	$1\pi_u 1\pi_u$	$3\sigma_g^+ 1\pi_g$	4.01	−75.435562	6.20	−75.425234	6.29	−75.428676	6.25	−75.374174	6.30
N_2	$1\,^1\Sigma_g^+$	$1\,^1A_g$	ground state		0.13	−109.145744	0.00	−109.133354	0.00	−109.134153	0.00	−109.056141	0.00
N_2	$1\,^3\Sigma_u^+$	$1\,^3B_{1u}$	$1\pi_u$	$1\pi_g$	2.01	−108.861273	7.74	−108.849438	7.73	−108.852428	7.67	−108.757450	8.13
N_2	$1\,^3\Pi_g$	$1\,^3B_{2g}$	$3\sigma_g^+$	$1\pi_g$	2.08	−108.855871	7.89	−108.843218	7.90	−108.846363	7.83	−108.749186	8.35
N_2	$1\,^1\Pi_g$	$1\,^1B_{2g}$	$3\sigma_g^+$	$1\pi_g$	2.07	−108.810681	9.12	−108.798263	9.12	−108.801119	9.06	−108.703573	9.59
N_2	$1\,^3\Delta_u$	$1\,^3A_u$	$1\pi_u$	$1\pi_g$	2.00	−108.806085	9.24	−108.794039	9.23	−108.797084	9.17	−108.700655	9.67
N_2	$1\,^3\Delta_u$	$2\,^3B_{1u}$	$1\pi_u$	$1\pi_g$	2.01	−108.806085	9.24	−108.793907	9.24	−108.797060	9.17	−108.700540	9.68
N_2	$1\,^3\Sigma_u^-$	$2\,^3A_u$	$1\pi_u$	$1\pi_g$	2.04	−108.784985	9.82	−108.772947	9.81	−108.776508	9.73	−108.675957	10.35
N_2	$1\,^1\Sigma_u^-$	$1\,^1A_u$	$1\pi_u$	$1\pi_g$	2.00	−108.758765	10.53	−108.747145	10.51	−108.749983	10.45	−108.651369	11.01
N_2	$1\,^1\Delta_u$	$1\,^1B_{1u}$	$1\pi_u$	$1\pi_g$	2.04	−108.749856	10.77	−108.738320	10.75	−108.741559	10.68	−108.639979	11.32
N_2	$1\,^1\Delta_u$	$2\,^1A_u$	$1\pi_u$	$1\pi_g$	2.00	−108.749856	10.77	−108.737743	10.77	−108.741535	10.68	−108.640038	11.32
N_2	$1\,^3\Pi_u$	$1\,^3B_{3u}$	$2\sigma_u^+$	$1\pi_g$	2.16	−108.742842	10.96	−108.730574	10.96	−108.734154	10.88	−108.633680	11.50
N_2	$1\,^1\Pi_u$	$1\,^1B_{3u}$	$2\sigma_u^+$	$1\pi_g$	2.17	−108.654522	13.37	−108.641368	13.39	−108.645137	13.31	−108.544680	13.92
H_2O		$1\,^1A_1$	ground state		0.03	−76.157900	0.00	−76.146217	0.00	−76.146724	0.00	−76.032496	0.00
H_2O		$1\,^3B_1$	$1b_1$	$4a_1$	2.00	−75.897447	7.09	−75.887157	7.05	−75.889311	7.00	−75.796368	6.43
H_2O		$1\,^1B_1$	$1b_1$	$4a_1$	2.01	−75.875183	7.69	−75.865487	7.64	−75.867384	7.60	−75.775806	6.98
H_2O		$1\,^3A_1$	$3a_1$	$4a_1$	2.00	−75.816009	9.30	−75.806348	9.25	−75.808379	9.21	−75.716269	8.60

Table S5: ... continued

Molecule	State	Irrep	Transition		NOS	E_{FCI}	V_{FCI}	E_{IPEA}	V_{IPEA}	E_{NOIPEA}	V_{NOIPEA}	E_{CASSCF}	V_{CASSCF}
H_2O		$1\,^3A_2$	$1b_1$	$2b_2$	2.00	−75.809666	9.48	−75.799127	9.44	−75.801235	9.40	−75.707713	8.84
H_2O		$1\,^1A_2$	$1b_1$	$2b_2$	2.00	−75.794848	9.88	−75.785428	9.82	−75.787308	9.78	−75.695509	9.17
H_2O		$2\,^1A_1$	$3a_1$	$4a_1$	2.00	−75.788427	10.05	−75.782363	9.90	−75.784904	9.85	−75.676197	9.70
H_2O		$1\,^3B_2$	$3a_1$	$2b_2$	2.00	−75.737527	11.44	−75.728983	11.35	−75.731478	11.30	−75.622299	11.16
H_2O		$1\,^1B_2$	$3a_1$	$2b_2$	2.00	−75.707317	12.26	−75.698328	12.19	−75.700243	12.15	−75.602023	11.71
H_2O		$2\,^3B_2$	$1b_2$	$4a_1$	2.00	−75.664811	13.42	−75.655141	13.36	−75.657964	13.30	−75.548573	13.17
CH_2		$1\,^3B_2$	ground state		2.02	−38.977380	0.00	−38.966975	0.00	−38.968703	0.00	−38.921047	0.00
CH_2		$1\,^1A_1$	$1b_2$	$3a_1$	0.04	−38.959383	0.49	−38.946559	0.56	−38.947187	0.59	−38.898387	0.62
CH_2		$1\,^1B_2$	ground state		2.02	−38.896701	2.20	−38.884203	2.25	−38.886493	2.24	−38.830811	2.46
CH_2		$2\,^1A_1$	$3a_1$	$1b_2$	0.04	−38.799468	4.84	−38.786452	4.91	−38.787188	4.94	−38.735241	5.06
CH_2		$1\,^3A_2$	$1b_1$	$3a_1$	2.04	−38.770184	5.64	−38.759547	5.64	−38.761615	5.64	−38.709404	5.76
CH_2		$1\,^1A_2$	$1b_1$	$3a_1$	2.05	−38.744982	6.32	−38.735063	6.31	−38.737310	6.30	−38.682038	6.50
CH_2		$1\,^3B_1$	$1b_1$	$1b_2$	2.00	−38.718364	7.05	−38.706121	7.10	−38.708936	7.07	−38.644812	7.52
CH_2		$1\,^3A_1$	$1b_2$	$4a_1$	2.00	−38.708694	7.31	−38.698555	7.30	−38.700548	7.30	−38.659377	7.12
CH_2		$1\,^1B_1$	$1b_1$	$1b_2$	2.01	−38.662964	8.56	−38.652786	8.55	−38.655794	8.51	−38.596097	8.84
CH_2		$3\,^1A_1$	$1b_2$	$4a_1$	2.01	−38.691490	7.78	−38.680715	7.79	−38.683132	7.77	−38.629911	7.92
CH_2		$2\,^3B_1$	$1b_2$	$2b_1$	2.00	−38.659327	8.65	−38.646577	8.72	−38.649304	8.69	−38.593935	8.90
CH_2		$2\,^3B_2$	$3a_1$	$4a_1$	2.07	−38.625742	9.57	−38.614380	9.59	−38.617230	9.56	−38.569138	9.58
CH_2		$1\,^5A_2$	$1b_1$	$4a_1$	2.00	−38.610558	9.98	−38.602758	9.91	−38.606209	9.86	−38.562830	9.75
CH_2		$2\,^1B_1$	$1b_2$	$2b_1$	4.00	−38.620301	9.72	−38.609064	9.74	−38.612266	9.70	−38.557666	9.89
CH_2		$1\,^5B_2$	$1b_1$	$2b_1$	2.08	−38.579548	10.83	−38.572918	10.72	−38.576037	10.68	−38.536998	10.45
CH_2		$2\,^3A_2$	$3a_1$	$2b_1$	4.01	−38.577934	10.87	−38.559070	11.10	−38.561979	11.07	−38.508787	11.22
CH_2		$2\,^1B_2$	$3a_1$	$4a_1$	2.07	−38.605222	10.13	−38.594830	10.13	−38.597487	10.10	−38.549094	10.12
CH_2		$2\,^1A_2$	$3a_1$	$2b_1$	2.01	−38.559576	11.37	−38.544116	11.51	−38.546883	11.48	−38.493317	11.64
CH_2		$3\,^3B_1$	$1b_11b_2$	$3a_14a_1$	2.06	−38.562058	11.30	−38.548132	11.40	−38.551609	11.35	−38.494707	11.60

S2.5 The 2 $^1\Sigma^+$ State of NH

Inspection of Figure 3 in the main paper shows one data point, labeled ψ_1, at NOS ≈ 0 and ΔNOS ≈ -2, respectively, with an error considerably larger than that of the rest of the states. This state is the 2 $^1\Sigma^+$ (3 $^1A_1$) state of the NH molecule. NH possesses a 1 $^3\Sigma^-$ (1 $^3A_2$) open-shell ground state belonging to the electronic configuration $(1\sigma^+)^2(2\sigma^+)^2(3\sigma^+)^2(1\pi 1\pi)^2$, and the 2 $^1\Sigma^+$ state may be described by a $(3\sigma^+)^2 \rightarrow (1\pi 1\pi)^2$ double excitation. For this transition, ΔNOS ≈ -2, and the resulting state is described by the closed-shell electronic configuration $(1\sigma^+)^2(2\sigma^+)^2(3\sigma^+)^0(1\pi 1\pi)^4$. This is a rather unique transition. There is no corresponding double excitation from a open-shell ground state yielding a closed-shell excited state in our test set, so we simply report this unusual large error but refrain from speculating about its origin.

S2.6 Potential Energy Curves of Diatomic Molecules

For the first-row hydrides (HLi, HBe, HB, HC, HN, HO, HF) and the homodiatomic molecules (Li_2, B_2, C_2, N_2), the potential energy curves were computed using CASPT2 (NOIPEA, $\varepsilon = 0$) and different ANO-RCC basis sets (MB, VDZ, VDZP, VTZP, VQZP). The active spaces and correlated electrons were the same as described in section S2.1. In Table S6, the total energies of these molecules are reported for various interatomic distances.

Table S6: Total NOIPEA CASPT2 energies of diatomic molecules in a.u. obtained using different ANO-RCC basis sets for different interatomic distances R (in Å).

Molecule	R	MB	VDZ	VDZP	VTZP	VQZP
HLi	1.20	−7.952643	−7.974778	−7.995687	−8.022978	−8.028067
HLi	1.30	−7.971420	−7.992330	−8.012077	−8.039030	−8.044010
HLi	1.35	−7.978083	−7.998450	−8.017664	−8.044454	−8.049365
HLi	1.40	−7.983312	−8.003212	−8.021900	−8.048500	−8.053333
HLi	1.42	−7.985054	−8.004786	−8.023269	−8.049781	−8.054582
HLi	1.44	−7.986615	−8.006188	−8.024469	−8.050889	−8.055656
HLi	1.46	−7.988007	−8.007429	−8.025514	−8.051834	−8.056568
HLi	1.48	−7.989241	−8.008521	−8.026414	−8.052627	−8.057327
HLi	1.50	−7.990327	−8.009473	−8.027178	−8.053277	−8.057945
HLi	1.52	−7.991273	−8.010294	−8.027815	−8.053796	−8.058431
HLi	1.54	−7.992090	−8.010991	−8.028333	−8.054190	−8.058793
HLi	1.55	−7.992452	−8.011297	−8.028551	−8.054343	−8.058930
HLi	1.56	−7.992784	−8.011574	−8.028741	−8.054469	−8.059040
HLi	1.57	−7.993088	−8.011825	−8.028906	−8.054567	−8.059123

Table S6: . . . continued

Molecule	R	MB	VDZ	VDZP	VTZP	VQZP
HLi	1.58	−7.993364	−8.012050	−8.029045	−8.054639	−8.059180
HLi	1.59	−7.993613	−8.012250	−8.029160	−8.054686	−8.059212
HLi	1.60	−7.993836	−8.012425	−8.029252	−8.054709	−8.059219
HLi	1.61	−7.994034	−8.012577	−8.029321	−8.054708	−8.059204
HLi	1.62	−7.994207	−8.012706	−8.029367	−8.054684	−8.059166
HLi	1.63	−7.994357	−8.012814	−8.029393	−8.054639	−8.059106
HLi	1.64	−7.994483	−8.012900	−8.029398	−8.054572	−8.059026
HLi	1.66	−7.994670	−8.013011	−8.029348	−8.054378	−8.058805
HLi	1.68	−7.994774	−8.013046	−8.029224	−8.054108	−8.058509
HLi	1.70	−7.994799	−8.013009	−8.029030	−8.053767	−8.058144
HLi	1.75	−7.994547	−8.012633	−8.028267	−8.052636	−8.056957
HLi	1.80	−7.993902	−8.011905	−8.027157	−8.049948	−8.055433
HLi	1.85	−7.992919	−8.010883	−8.025755	−8.048205	−8.052470
HLi	1.90	−7.991651	−8.009616	−8.024109	−8.046233	−8.050459
HLi	2.00	−7.988429	−8.006510	−8.020248	−8.041774	−8.045933
HLi	2.10	−7.984534	−8.002866	−8.015847	−8.036854	−8.040956
HLi	2.20	−7.980200	−7.998889	−8.011115	−8.032751	−8.035731
HLi	2.40	−7.970933	−7.990532	−8.001256	−8.022120	−8.026019
HLi	2.60	−7.961757	−7.982294	−7.991534	−8.011851	−8.015639
HLi	3.00	−7.946366	−7.967974	−7.974321	−7.993985	−7.997525
HLi	3.50	−7.934985	−7.955984	−7.959172	−7.978361	−7.981560
HLi	4.00	−7.930133	−7.950002		−7.970145	−7.973096
HLi	5.00	−7.927612	−7.946385	−7.946596	−7.965488	−7.968297
HLi	6.00	−7.927273	−7.945657	−7.945707	−7.964849	−7.967641
HLi	8.00	−7.927223	−7.945362	−7.945369	−7.964743	−7.967516
HLi	10.00	−7.927223	−7.945326	−7.945329	−7.964736	−7.967504
HBe	0.80	−14.921501	−14.952765	−14.981891	−15.031257	−15.043053
HBe	0.90	−15.018920	−15.046340	−15.072299	−15.119515	−15.130621
HBe	1.00	−15.081327	−15.104896	−15.127343	−15.173100	−15.183595
HBe	1.10	−15.120784	−15.140215	−15.159533	−15.204158	−15.214055
HBe	1.15	−15.134309	−15.151681	−15.169647	−15.213784	−15.223384
HBe	1.20	−15.144607		−15.176752	−15.220440	−15.229752
HBe	1.21	−15.146327	−15.161345	−15.177864	−15.221465	−15.230721
HBe	1.22	−15.147944		−15.178881	−15.222398	−15.231599
HBe	1.23	−15.149460	−15.163735	−15.179808	−15.223243	−15.232389
HBe	1.24	−15.150879	−15.164792	−15.180649	−15.224002	−15.233095
HBe	1.25	−15.152205	−15.165762	−15.181407	−15.224680	−15.233721
HBe	1.26	−15.153441	−15.166648	−15.182086	−15.225279	−15.234269
HBe	1.27	−15.154590	−15.167455	−15.182688	−15.225803	−15.234742

Table S6: ... continued

Molecule	R	MB	VDZ	VDZP	VTZP	VQZP
HBe	1.28	−15.155655	−15.168185	−15.183217	−15.226255	−15.235145
HBe	1.30	−15.157545	−15.169426	−15.184066	−15.226955	−15.235751
HBe	1.32	−15.159133	−15.170397	−15.184657	−15.227400	−15.236107
HBe	1.35	−15.160996	−15.171392	−15.185104	−15.227637	−15.236221
HBe	1.40	−15.162874	−15.171984	−15.184830	−15.227031	−15.235440
HBe	1.45	−15.163447	−15.171476	−15.183501	−15.225392	−15.233662
HBe	1.50	−15.162946	−15.170094	−15.181332	−15.222932	−15.231094
HBe	1.60	−15.159495	−15.165441	−15.176594	−15.216224	−15.224242
HBe	1.70	−15.153824	−15.159222	−15.169405	−15.208007	−15.215935
HBe	1.80	−15.146936	−15.152298	−15.161569	−15.200511	−15.208480
HBe	2.00	−15.132506	−15.138727	−15.146177	−15.183956	−15.191703
HBe	2.20	−15.120745	−15.128085	−15.133615	−15.170349	−15.177819
HBe	2.40	−15.113757	−15.121701	−15.125407	−15.161291	−15.168458
HBe	2.60	−15.110896	−15.118920	−15.121257	−15.156589	−15.163508
HBe	3.00	−15.110420	−15.118093	−15.119095	−15.153986	−15.160653
HBe	4.00	−15.111604	−15.118818	−15.119007	−15.153804	−15.160338
HBe	5.00	−15.111758	−15.118803	−15.118894	−15.153756	−15.160271
HBe	6.00	−15.111768	−15.118719	−15.118784	−15.153721	−15.160228
HBe	8.00	−15.111768	−15.118669	−15.118710	−15.153704	−15.160207
HB	0.80	−24.917348	−25.006297	−25.052045	−25.103969	−25.120611
HB	0.90	−25.023100	−25.095271	−25.138561	−25.184759	−25.200380
HB	1.00	−25.088214	−25.144666	−25.186451	−25.228520	−25.243835
HB	1.10	−25.126568	−25.169825	−25.210275	−25.249796	−25.264724
HB	1.15	−25.138678	−25.176439	−25.216216	−25.254786	−25.269616
HB	1.20	−25.147232	−25.180237	−25.219312	−25.257084	−25.271875
HB	1.21	−25.148578	−25.180718	−25.219648	−25.257275	−25.272066
HB	1.22	−25.149813	−25.181118	−25.219901	−25.257388	−25.272180
HB	1.23	−25.150943	−25.181439	−25.220074	−25.257425	−25.272220
HB	1.24	−25.151972	−25.181688	−25.220171	−25.257392	−25.272191
HB	1.25	−25.152903	−25.181866	−25.220197	−25.257290	−25.272096
HB	1.26	−25.153742	−25.181979	−25.220156	−25.257124	−25.271939
HB	1.27	−25.154491	−25.182030	−25.220050	−25.256897	−25.271722
HB	1.28	−25.155156	−25.182021	−25.219882	−25.256613	−25.271449
HB	1.30	−25.156242	−25.181840	−25.219378	−25.255883	−25.270747
HB	1.32	−25.157029	−25.181459	−25.218666	−25.254957	−25.269854
HB	1.35	−25.157701	−25.180562	−25.217256	−25.253245	−25.268200
HB	1.40	−25.157655	−25.178352	−25.214154	−25.249681	−25.264753
HB	1.45	−25.156409	−25.175460	−25.210328	−25.245435	−25.260641
HB	1.50	−25.154219	−25.172080	−25.205981	−25.240705	−25.256051

Table S6: ... continued

Molecule	R	MB	VDZ	VDZP	VTZP	VQZP
HB	1.60	−25.147814	−25.164422	−25.196336	−25.230384	−25.246008
HB	1.70	−25.139726	−25.156204	−25.186141	−25.219624	−25.235482
HB	1.80	−25.130849	−25.147910	−25.175962	−25.208982	−25.225007
HB	2.00	−25.112999	−25.132092	−25.156870	−25.189227	−25.205405
HB	2.20	−25.097193	−25.118104	−25.140417	−25.172363	−25.188552
HB	2.40	−25.084592	−25.106383	−25.127018	−25.158711	−25.174864
HB	2.60	−25.075318	−25.097046	−25.116678	−25.148201	−25.164321
HB	3.00	−25.064709	−25.084892	−25.103906	−25.135147	−25.151272
HB	3.50	−25.059836	−25.078189	−25.097476	−25.128373	−25.144605
HB	4.00	−25.058448	−25.075912	−25.095494	−25.126133	−25.142507
HB	5.00	−25.057980	−25.074919	−25.094643	−25.125007	−25.141615
HB	6.00	−25.057950	−25.074783	−25.094514	−25.124761	−25.141494
HB	8.00	−25.057948	−25.074754	−25.094484		
HC	0.70	−37.882867	−38.052246	−38.120907	−38.181000	−38.208941
HC	0.80	−38.061750	−38.193427	−38.259097	−38.308450	−38.337274
HC	0.90	−38.166345	−38.267015	−38.330853	−38.371717	−38.401715
HC	0.95	−38.200242	−38.288077	−38.351049	−38.388625	−38.418727
HC	1.00	−38.225288	−38.301983	−38.364085	−38.398991	−38.428630
HC	1.02	−38.233262	−38.305954	−38.367698	−38.401704	−38.430983
HC	1.04	−38.240226	−38.309159	−38.370538	−38.403738	−38.432559
HC	1.06	−38.246270	−38.311682	−38.372689	−38.405172	−38.433446
HC	1.08	−38.251479	−38.313598	−38.374224	−38.406075	−38.433724
HC	1.09	−38.253795	−38.314350	−38.374782	−38.406347	−38.433658
HC	1.10	−38.255929	−38.314976	−38.375211	−38.406509	−38.433470
HC	1.11	−38.257891	−38.315481	−38.375518	−38.406567	−38.433166
HC	1.12	−38.259672	−38.315871	−38.375708	−38.406527	−38.432760
HC	1.13	−38.261330	−38.316162	−38.375793	−38.406395	−38.432247
HC	1.14	−38.262823	−38.316349	−38.375775	−38.406177	−38.431648
HC	1.15	−38.264173	−38.316443	−38.375660	−38.405877	−38.430967
HC	1.16	−38.265387	−38.316449	−38.375455	−38.405500	−38.430209
HC	1.18	−38.267437	−38.316217	−38.374796	−38.404536	−38.428495
HC	1.20	−38.269019	−38.315694	−38.373838	−38.403315	−38.426553
HC	1.22	−38.270178	−38.314914	−38.372616	−38.401869	−38.424428
HC	1.25	−38.271211	−38.313334	−38.370359	−38.399334	−38.420973
HC	1.30	−38.271346	−38.309828	−38.365703	−38.394327	−38.414729
HC	1.35	−38.269905	−38.305530	−38.360237	−38.388605	−38.408131
HC	1.40	−38.267276	−38.300711	−38.354249	−38.382415	−38.401362
HC	1.50	−38.259658	−38.290277	−38.341536	−38.369380	−38.387746
HC	1.60	−38.250371	−38.279647	−38.328786	−38.356341	−38.374497

Table S6: . . . continued

Molecule	R	MB	VDZ	VDZP	VTZP	VQZP
HC	1.80	−38.231320	−38.260085	−38.305695	−38.332673	−38.350563
HC	2.00	−38.215548	−38.244389	−38.287568	−38.313956	−38.331441
HC	2.50	−38.195793	−38.223114	−38.263513	−38.288595	−38.305240
HC	3.00	−38.191150	−38.216924	−38.256753	−38.281101	−38.297466
HC	4.00	−38.189958	−38.214913	−38.254538	−38.278406	−38.294807
HC	6.00	−38.189898	−38.214616	−38.254189	−38.277937	−38.294335
HC	8.00	−38.189898	−38.214598	−38.254168	−38.277897	−38.294265
HC	10.00	−38.189898	−38.214598	−38.254167		
HN	0.70	−54.674766	−54.855509	−54.945379	−54.992510	−55.020472
HN	0.80	−54.835425	−54.974562	−55.063853	−55.106077	−55.131301
HN	0.88	−54.908841	−55.022620	−55.110388	−55.150256	−55.173541
HN	0.90	−54.921946	−55.030393	−55.117646	−55.157064	−55.179916
HN	0.95	−54.947712	−55.044409	−55.130193	−55.168659	−55.190532
HN	0.98	−54.959153	−55.049795	−55.134599	−55.172588	−55.193948
HN	1.00	−54.965383	−55.052366	−55.136486	−55.174188	−55.195236
HN	1.01	−54.968122	−55.053382	−55.137153	−55.174720	−55.195619
HN	1.02	−54.970627	−55.054232	−55.137650	−55.175087	−55.195844
HN	1.03	−54.972911	−55.054927	−55.137989	−55.175300	−55.195921
HN	1.04	−54.974985	−55.055477	−55.138180	−55.175370	−55.195858
HN	1.05	−54.976861	−55.055890	−55.138231	−55.175304	−55.195666
HN	1.06	−54.978549	−55.056175	−55.138153	−55.175113	−55.195353
HN	1.08	−54.981399	−55.056393	−55.137641	−55.174385	−55.194396
HN	1.10	−54.983612	−55.056190	−55.136705	−55.173247	−55.193046
HN	1.15	−54.986777	−55.054171	−55.132869	−55.168949	−55.188290
HN	1.20	−54.987250	−55.050517	−55.127446	−55.163114	−55.182090
HN	1.30	−54.982788	−55.040266	−55.113897	−55.148843	−55.167311
HN	1.50	−54.964310	−55.016316	−55.084575	−55.118287	−55.136283
HN	1.80	−54.938574	−54.987755	−55.050880	−55.082907	−55.100356
HN	2.00	−54.928941	−54.976590	−55.037782	−55.068793	−55.085826
HN	3.00	−54.919970	−54.964145	−55.022795	−55.051639	−55.067847
HN	4.00	−54.919738	−54.963608	−55.022048	−55.050615	−55.066750
HN	5.00	−54.919730	−54.963518	−55.021929	−55.050468	−55.066552
HN	6.00	−54.919730	−54.963493	−55.021898	−55.050431	−55.066488
HN	8.00	−54.919730	−54.963487	−55.021890	−55.050418	−55.066463
HN	10.00	−54.919730	−54.963487	−55.021889	−55.050416	−55.066461
HO	0.70	−75.178757	−75.363700	−75.481842	−75.557161	−75.588309
HO	0.75	−75.263571	−75.428476	−75.543506	−75.614113	−75.644111
HO	0.80	−75.324685	−75.472337	−75.584241	−75.651096	−75.680119
HO	0.85	−75.368115	−75.501120	−75.609878	−75.673745	−75.701981

Table S6: . . . continued

Molecule	R	MB	VDZ	VDZP	VTZP	VQZP
HO	0.88	−75.387579	−75.512941	−75.619810	−75.682186	−75.710038
HO	0.90	−75.398290	−75.518997	−75.624613	−75.686102	−75.713730
HO	0.92	−75.407418	−75.523799	−75.628168	−75.688848	−75.716275
HO	0.94	−75.415131	−75.527498	−75.630631	−75.690571	−75.717818
HO	0.95	−75.418506	−75.528977	−75.631496	−75.691091	−75.718254
HO	0.96	−75.421583	−75.530230	−75.632138	−75.691403	−75.718486
HO	0.97	−75.424379	−75.531270	−75.632572	−75.691521	−75.718528
HO	0.98	−75.426910	−75.532112	−75.632811	−75.691458	−75.718392
HO	0.99	−75.429191	−75.532767	−75.632869	−75.691227	−75.718092
HO	1.00	−75.431235	−75.533248	−75.632757	−75.690839	−75.717637
HO	1.02	−75.434667	−75.533732	−75.632071	−75.689636	−75.716306
HO	1.05	−75.438352	−75.533411	−75.630038	−75.686906	−75.713402
HO	1.10	−75.441286	−75.530651	−75.624549	−75.680433	−75.706670
HO	1.20	−75.438739	−75.519662	−75.608627	−75.663026	−75.688827
HO	1.30	−75.429724	−75.505013	−75.589772	−75.643093	−75.668535
HO	1.50	−75.405699	−75.474416	−75.552783	−75.604604	−75.629495
HO	1.80	−75.376322	−75.439677	−75.512427	−75.562854	−75.587011
HO	2.00	−75.365292	−75.426120	−75.496944	−75.546779	−75.570473
HO	3.00	−75.353579	−75.410529	−75.479185	−75.528037	−75.550879
HO	4.00	−75.353145	−75.409773	−75.478233	−75.527073	−75.549870
HO	5.00	−75.353124	−75.409608	−75.478028	−75.526904	−75.549709
HO	6.00	−75.353123	−75.409544	−75.477956	−75.526840	−75.549644
HO	8.00	−75.353123	−75.409520	−75.477929	−75.526809	−75.549613
HO	10.00	−75.353123	−75.409520	−75.477928	−75.526807	−75.549610
HF	0.60	−99.660118	−99.890776	−100.038852	−100.150814	−100.192425
HF	0.70	−99.908280	−100.100202	−100.237925	−100.336751	−100.374817
HF	0.75	−99.980781	−100.157051	−100.289776	−100.383761	−100.420561
HF	0.80	−100.031089	−100.194115	−100.321953	−100.411977	−100.447878
HF	0.83	−100.053158	−100.209298	−100.334280	−100.422288	−100.457802
HF	0.85	−100.065124	−100.217078	−100.340197	−100.426994	−100.462299
HF	0.87	−100.075186	−100.223257	−100.344547	−100.430232	−100.465361
HF	0.88	−100.079572	−100.225809	−100.346200	−100.431362	−100.466414
HF	0.89	−100.083562	−100.228033	−100.347536	−100.432197	−100.467178
HF	0.90	−100.087180	−100.229951	−100.348575	−100.432756	−100.467672
HF	0.91	−100.090447	−100.231582	−100.349340	−100.433060	−100.467916
HF	0.92	−100.093384	−100.232945	−100.349847	−100.433126	−100.467926
HF	0.93	−100.096011	−100.234057	−100.350115	−100.432970	−100.467719
HF	0.94	−100.098346	−100.234934	−100.350160	−100.432610	−100.467310
HF	0.96	−100.102208	−100.236045	−100.349643	−100.431330	−100.465943

Table S6: ... continued

Molecule	R	MB	VDZ	VDZP	VTZP	VQZP
HF	0.98	−100.105100	−100.236388	−100.348408	−100.429394	−100.463929
HF	1.00	−100.107134	−100.236061	−100.346554	−100.426893	−100.461358
HF	1.05	−100.109105	−100.232853	−100.339756	−100.418685	−100.452998
HF	1.10	−100.107629	−100.227066	−100.330700	−100.408459	−100.442639
HF	1.20	−100.098058	−100.210784	−100.308789	−100.384724	−100.418659
HF	1.30	−100.083849	−100.191521	−100.284969	−100.359547	−100.393244
HF	1.40	−100.068179	−100.171706	−100.261488	−100.335037	−100.368498
HF	1.50	−100.052900	−100.152739	−100.239585	−100.312375	−100.345585
HF	1.60	−100.039040	−100.135423	−100.219937	−100.292212	−100.325139
HF	1.80	−100.017208	−100.107248	−100.188537	−100.260365	−100.292645
HF	2.00	−100.003125	−100.088008	−100.167476	−100.239301	−100.270986
HF	2.50	−99.989050	−100.067508	−100.145380	−100.217440	−100.248333
HF	3.00	−99.986212	−100.063200	−100.140699	−100.212869	−100.243566
HF	4.00	−99.985579	−100.062089	−100.139385	−100.211673	−100.242362
HF	5.00	−99.985555	−100.061921	−100.139175	−100.211512	−100.242212
HF	6.00	−99.985554	−100.061863	−100.139109	−100.211460	−100.242157
HF	8.00	−99.985554	−100.061843	−100.139087	−100.211438	−100.242135
HF	10.00	−99.985554	−100.061843	−100.139086	−100.211436	−100.242133
Li_2	1.50	−14.794881	−14.800108	−14.805324	−14.813445	−14.813933
Li_2	2.00	−14.865549	−14.868701	−14.876246	−14.881666	−14.882003
Li_2	2.20	−14.879434	−14.881897	−14.889489	−14.894406	−14.894737
Li_2	2.20	−14.879434	−14.881897	−14.889489	−14.894406	−14.894737
Li_2	2.30	−14.884196	−14.886351	−14.893822	−14.898501	−14.898836
Li_2	2.40	−14.887767	−14.889643	−14.896929	−14.901373	−14.901713
Li_2	2.50	−14.890322	−14.891948	−14.899006	−14.903219	−14.903564
Li_2	2.50	−14.890322	−14.891948	−14.899006	−14.903219	−14.903564
Li_2	2.55	−14.891269	−14.892782	−14.899714	−14.903812	−14.904160
Li_2	2.58	−14.891743	−14.893191	−14.900044	−14.904075	−14.904424
Li_2	2.60	−14.892021	−14.893428	−14.900228	−14.904213	−14.904563
Li_2	2.62	−14.892271	−14.893637	−14.900383	−14.904324	−14.904674
Li_2	2.64	−14.892493	−14.893820	−14.900511	−14.904408	−14.904758
Li_2	2.65	−14.892594	−14.893902	−14.900565	−14.904440	−14.904791
Li_2	2.66	−14.892689	−14.893978	−14.900613	−14.904466	−14.904817
Li_2	2.67	−14.892777	−14.894048	−14.900654	−14.904486	−14.904837
Li_2	2.68	−14.892860	−14.894111	−14.900690	−14.904500	−14.904851
Li_2	2.69	−14.892936	−14.894169	−14.900719	−14.904508	−14.904859
Li_2	2.70	−14.893006	−14.894221	−14.900743	−14.904510	−14.904861
Li_2	2.72	−14.893128	−14.894309	−14.900773	−14.904497	−14.904848
Li_2	2.75	−14.893270	−14.894399	−14.900776	−14.904437	−14.904789

Table S6: ... continued

Molecule	R	MB	VDZ	VDZP	VTZP	VQZP
Li_2	2.80	−14.893400	−14.894450	−14.900681	−14.904237	−14.904589
Li_2	2.90	−14.893314	−14.894222	−14.900152	−14.903508	−14.903861
Li_2	3.00	−14.892841	−14.893627	−14.899252	−14.902416	−14.902770
Li_2	4.00	−14.880095	−14.880331	−14.882958	−14.884660	−14.884992
Li_2	5.00	−14.870815	−14.870968	−14.871749	−14.872449	−14.872620
Li_2	6.00	−14.867702	−14.867860	−14.868055	−14.868289	−14.868350
Li_2	8.00	−14.866658	−14.866836	−14.866852	−14.866903	−14.866915
Li_2	10.00	−14.866558	−14.866742	−14.866745	−14.866774	−14.866782
B_2	0.80	−47.803430	−47.986930	−48.051972	−48.120571	−48.142814
B_2	1.00	−48.642764	−48.759167	−48.817820	−48.860983	−48.873377
B_2	1.20	−49.012372	−49.091329	−49.145509	−49.173168	−49.182503
B_2	1.30	−49.104302	−49.169294	−49.221714	−49.245034	−49.253495
B_2	1.40	−49.159996	−49.213269	−49.264159	−49.284431	−49.292104
B_2	1.45	−49.177924	−49.226400	−49.276626	−49.295769	−49.303108
B_2	1.50	−49.190862	−49.235130	−49.284750	−49.302957	−49.309984
B_2	1.52	−49.194852	−49.237593	−49.286985	−49.304862	−49.311781
B_2	1.54	−49.198245	−49.239544	−49.288716	−49.306285	−49.313102
B_2	1.56	−49.201088	−49.241029	−49.289988	−49.307269	−49.313992
B_2	1.57	−49.202318	−49.241608	−49.290465	−49.307609	−49.314287
B_2	1.58	−49.203426	−49.242089	−49.290842	−49.307854	−49.314490
B_2	1.59	−49.204420	−49.242473	−49.291125	−49.308009	−49.314603
B_2	1.60	−49.205302	−49.242764	−49.291318	−49.307876	−49.314632
B_2	1.61	−49.206078	−49.242970	−49.291425	−49.307864	−49.314582
B_2	1.62	−49.206753	−49.243092	−49.291342	−49.307774	−49.314455
B_2	1.64	−49.207815	−49.243104	−49.291166	−49.307377	−49.313987
B_2	1.67	−49.208753	−49.242492	−49.290387	−49.306291	−49.312595
B_2	1.68	−49.208906	−49.242198	−49.290003	−49.305812	−49.312089
B_2	1.69	−49.208985	−49.241847	−49.289564	−49.305280	−49.311530
B_2	1.70	−49.208994	−49.241442	−49.289072	−49.304697	−49.310922
B_2	1.71	−49.208935	−49.240986	−49.288529	−49.304068	−49.310268
B_2	1.72	−49.208813	−49.240482	−49.287939	−49.303393	−49.309571
B_2	1.73	−49.208630	−49.239932	−49.287305	−49.302676	−49.308832
B_2	1.74	−49.208390	−49.239340	−49.286629	−49.301920	−49.308055
B_2	1.75	−49.208094	−49.238708	−49.285913	−49.301127	−49.307242
B_2	1.80	−49.205884	−49.235031	−49.281824	−49.296683	−49.302713
B_2	1.90	−49.198650	−49.225889	−49.271888	−49.286165	−49.292085
B_2	2.00	−49.189367	−49.215643	−49.260874	−49.274694	−49.280560
B_2	3.00	−49.131460	−49.157213	−49.198750	−49.210264	−49.216131
B_2	4.00	−49.128125	−49.152524	−49.193957	−49.204963	−49.210675

Table S6: ... continued

Molecule	R	MB	VDZ	VDZP	VTZP	VQZP
B_2	5.00	−49.127838	−49.152041	−49.193458	−49.204433	−49.210147
B_2	6.00	−49.127796	−49.151960	−49.193362	−49.204324	−49.210030
B_2	7.00	−49.127787	−49.151938	−49.193329	−49.204294	−49.209992
B_2	8.00	−49.127784	−49.151933	−49.193325	−49.204290	−49.209985
B_2	10.00	−49.127783	−49.151931	−49.193327	−49.204292	−49.209985
C_2	0.80	−74.431468	−74.778154	−74.878870	−75.007478	−75.035350
C_2	0.90	−74.954002	−75.225166	−75.321941	−75.422863	−75.448716
C_2	1.00	−75.255524	−75.467332	−75.559595	−75.641958	−75.668748
C_2	1.05	−75.351554	−75.538897	−75.629108	−75.704906	−75.732007
C_2	1.10	−75.421389	−75.587374	−75.675724	−75.746377	−75.773498
C_2	1.14	−75.462268	−75.613212	−75.700215	−75.767513	−75.794504
C_2	1.16	−75.478550	−75.622605	−75.708981	−75.774784	−75.801690
C_2	1.18	−75.492410	−75.629973	−75.715752	−75.780146	−75.806972
C_2	1.20	−75.504080	−75.635531	−75.720743	−75.783789	−75.810551
C_2	1.21	−75.509162	−75.637693	−75.722633	−75.785021	−75.811761
C_2	1.22	−75.513775	−75.639475	−75.724150	−75.785887	−75.812612
C_2	1.23	−75.517943	−75.640898	−75.725314	−75.786405	−75.813126
C_2	1.24	−75.521688	−75.641983	−75.726148	−75.786595	−75.813321
C_2	1.25	−75.525033	−75.642750	−75.726671	−75.786473	−75.813217
C_2	1.26	−75.527997	−75.643216	−75.726900	−75.786056	−75.812829
C_2	1.27	−75.530602	−75.643401	−75.726855	−75.785362	−75.812176
C_2	1.28	−75.532865	−75.643321	−75.726552	−75.784407	−75.811272
C_2	1.30	−75.536439	−75.642430	−75.725235	−75.781775	−75.808775
C_2	1.32	−75.538855	−75.640665	−75.723071	−75.778284	−75.805450
C_2	1.34	−75.540237	−75.638135	−75.720169	−75.774059	−75.801401
C_2	1.36	−75.540698	−75.634941	−75.716628	−75.769216	−75.796723
C_2	1.40	−75.539262	−75.626914	−75.707984	−75.758126	−75.785818
C_2	1.45	−75.533949	−75.614589	−75.695028	−75.742625	−75.770062
C_2	1.50	−75.525798	−75.600631	−75.680588	−75.726340	−75.752907
C_2	1.60	−75.504528	−75.570731	−75.650168	−75.693940	−75.717766
C_2	1.80	−75.458934	−75.515503	−75.609574	−75.647449	−75.668407
C_2	2.00	−75.430874	−75.488879	−75.576381	−75.621180	−75.637380
C_2	2.20	−75.414348	−75.467857	−75.551404	−75.594283	−75.614609
C_2	2.50	−75.399718	−75.448363	−75.529645	−75.569522	−75.584394
C_2	3.00	−75.393232	−75.436564	−75.514544	−75.551328	−75.569712
C_2	4.00	−75.391922	−75.432769	−75.513005	−75.548586	−75.564836
C_2	5.00	−75.391804	−75.432377	−75.512537	−75.547842	−75.566555
C_2	6.00	−75.391791	−75.432332	−75.512472	−75.547683	−75.566394
C_2	8.00	−75.391784	−75.432331	−75.512463	−75.545600	−75.564115

Table S6: ... continued

Molecule	R	MB	VDZ	VDZP	VTZP	VQZP
C_2	10.00	−75.391787	−75.432332	−75.512463	−75.547639	−75.564122
N_2	0.70	−107.205057	−107.786869	−107.990564	−108.143147	−108.178157
N_2	0.80	−108.136409	−108.582169	−108.778463	−108.885683	−108.914201
N_2	0.90	−108.630119	−108.968474		−109.238538	−109.263621
N_2	0.95	−108.776878	−109.071621	−109.254598		−109.353440
N_2	1.00	−108.878630	−109.135820	−109.314359	−109.383887	−109.406640
N_2	1.03	−108.922921	−109.160258	−109.336131	−109.403123	−109.425344
N_2	1.06	−108.957096	−109.176443	−109.349639	−109.414560	−109.436310
N_2	1.08	−108.975086	−109.183418	−109.354820	−109.418579	−109.440045
N_2	1.09	−108.982807	−109.185913	−109.356415	−109.419651	−109.440983
N_2	1.10	−108.989745	−109.187808	−109.357406	−109.420155	−109.441358
N_2	1.11	−108.995947	−109.189144	−109.357835	−109.420131	−109.441210
N_2	1.12	−109.001456	−109.189962	−109.357743	−109.419617	−109.440575
N_2	1.13	−109.006315	−109.190299	−109.357168	−109.418649	−109.439491
N_2	1.14	−109.010561	−109.190191	−109.356144	−109.417260	−109.437989
N_2	1.15	−109.014234	−109.189671	−109.354705	−109.415481	−109.436101
N_2	1.17	−109.019995	−109.187522	−109.350707	−109.410873	−109.431285
N_2	1.20	−109.025155	−109.181943	−109.342330	−109.401729	−109.421854
N_2	1.23	−109.026810	−109.174122	−109.331689	−109.390461	−109.410327
N_2	1.27	−109.024677	−109.161154	−109.314938	−109.373032	−109.392596
N_2	1.42	−108.990535	−109.101031	−109.240994	−109.297268	−109.316014
N_2	1.58	−108.939544	−109.038627	−109.166200	−109.220526	−109.238571
N_2	1.74	−108.896315	−108.990787	−109.109660	−109.161296	−109.178510
N_2	1.90	−108.869632	−108.960180	−109.073752	−109.122122	−109.138344
N_2	2.00	−108.860726	−108.948377	−109.059928	−109.106426	−109.122064
N_2	2.50	−108.851605	−108.929188	−109.037187	−109.079144	−109.093322
N_2	3.00	−108.851668	−108.926749	−109.034086	−109.075063	−109.088838
N_2	4.00	−108.851646	−108.925978	−109.033451	−109.074189	−109.087860
N_2	6.00	−108.851630	−108.925893	−109.033593	−109.074295	−109.087974
N_2	8.00	−108.851629	−108.925890	−109.033592	−109.074290	−109.087967
N_2	10.00	−108.851628	−108.925842	−109.033025	−109.073726	−109.087416

S3 Thiel's Benchmark Set

S3.1 Comments of the Benchmark Calculations of Thiel

Here we report a small number of inconsistencies that we have found between our calculations and the results reported by Thiel and coworkers,[51] when attempting to reproduce their values. Table S7 provides a complete list of the excitation energies reported in the Supporting Information of Ref. 51 as well as our own results.

When calculating the vertical excitation energies, Thiel et al. used two different ground state energies as the reference energies: for states that possess the same symmetry as the ground state –in our case this applies always to the totally symmetric singlet states –the MS-CASPT2 energy of the ground state was used as the reference energy. However, for states possessing a different symmetry than the ground state, the energy of the ground states was calculated separately using the SS-CASPT2 variant. We believe, that for a number of non-totally symmetric excited states of the molecules butadiene, benzene, naphthalene, and acetamide, the authors unintentionally used the MS-CASPT2 ground state energy as the reference energy in contrast to their explanations. Only under this assumption were we able to reproduce their results. Table S8 in section S3.2 lists both SS-CASPT2 and MS-CASPT2 energies for all ground states.

Furthermore, for imidazole and pyridazine, the active spaces were erroneously reported in the SI of Ref. 51. The correct list of orbitals comprising the active space with all valence π/π^* as well as n orbitals is given as a comment in Table S7. As special case is tetrazine. Tetrazine possesses 14 electrons in the valence π and n orbitals. The active space reported in Ref. 51, however, contained only 12 electrons. Using the more reasonable active space with 14 electrons, ca. 2/3 of all excitation energies could be reproduced, while for the other excited states the deviations could become as large as 2 eV. For acetone, one state ($1\ ^1B_1$) is reported at 9.27 eV while our calculations predict it to be well above 10 eV. We found that using a smaller active space than reported in the original benchmark study, however, reproduces the reported excitation energy. For benzoquinone, we could not reproduce the energies of three states satisfactorily. For formamide, the excitation energy of the $1\ ^3A'$ state is reported at 5.58 eV while we obtain a excitation energy of 5.68 eV. In addition, we note that the vertical excitation energies of the $2\ ^1A_g$ and the $1\ ^3B_u$ states of butadiene are correctly reported in the Supporting Information while apparently two typos have been introduced in these energies in

the main paper.

Finally, we wish to address the characterization of the excited states of triazine. Thiel and co-workers report the lowest-lying $^1\pi\pi^*$ excited states to be the 1 $^1A_2'$, 2 $^1A_1'$, 1 $^1E'$, and 2 $^1E'$ states,[51] in accordance with the classification given in an earlier CASPT2 study by the Roos group.[2] However, by calculating the excited states of triazine in C_s symmetry, as was done by Thiel and co-workers, our results suggest a different characterization for some of the states. We agree that the first two $^1\pi\pi^*$ excited states are the 1 $^1A_2'$ and 2 $^1A_1'$ states, however, we question the characterization of the other two states as $^1E'$ states. These two states were reported to lie at 7.49 and 8.99 eV. We, too, find each one $^1\pi\pi^*$ state at 7.50 and 8.95 eV, however, the second component to both $^1E'$ states is missing. The next higher-excited state of A' symmetry appears at 9.08 eV. It is not a described by a $\pi \rightarrow \pi^*$ excitation but is rather given by a $(nn) \rightarrow (\pi^*\pi^*)$ double excitation. Since our calculations did not exploit the full D_{3h} symmetry of the molecule, one might wonder whether either one of the components of the $^1E'$ states may have been lost due to symmetry splitting. However, this is unlikely since using the very same approach, our calculations predicted both components of two $^1E''$ states of $n\pi^*$ character with an energetic splitting smaller than 0.01 eV. Thus, both $^1\pi\pi^*$ states may require a new classification, which is, however, not of importance for the rest of this work.

Table S7: Comparison of vertical excitation energies reported in the benchmark study by Thiel and co-workers[51] and vertical excitation energies computed in this work. Differences larger than 0.05 eV are marked as "significant" in the comments. The order of the molecules and excited states is adopted from the Supporting Information of Ref. 51.

**Molecule**	**State**	$\mathbf{V_i}$(Thiel SI)[a]	$\mathbf{V_i}$(MS)[b]	$\mathbf{V_i}$(SS)[c]	**Comment**
Ethene	1 $^1B_{1u}$	8.62	8.54	8.54	significant difference
Ethene	1 $^3B_{1u}$	4.60	4.48	4.48	significant difference
Butadiene	1 ^1B_u	6.47	6.47	6.42	MS instead of SS
Butadiene	2 ^1A_g	6.63	6.62	6.58	reported as $V_i = 6.83$ eV in main paper
Butadiene	1 ^3B_u	3.34	3.39	3.34	reported as $V_i = 3.44$ eV in main paper
Butadiene	1 ^3A_g	5.16	5.21	5.16	
Hexatriene	1 ^1B_u	5.31	5.35	5.32	
Hexatriene	2 ^1A_g	5.42	5.42	5.39	
Hexatriene	1 ^3B_u	2.71	2.74	2.71	
Hexatriene	1 ^3A_g	4.31	4.34	4.31	
Octatetraene	2 ^1A_g	4.64	4.64	4.59	
Octatetraene	1 ^1B_u	4.70	4.75	4.70	
Octatetraene	2 ^1B_u	5.74	5.79	5.74	
Octatetraene	3 ^1A_g	6.19	6.19	6.14	

Table S7: … continued

**Molecule**	**State**	$\mathbf{V_i}$(Thiel SI)[a]	$\mathbf{V_i}$(MS)[b]	$\mathbf{V_i}$(SS)[c]	**Comment**
Octatetraene	$4\ ^1A_g$	6.57	7.53	7.48	
Octatetraene	$3\ ^1B_u$	8.04	8.09	8.04	
Octatetraene	$1\ ^3B_u$	2.33	2.38	2.33	
Octatetraene	$1\ ^3A_g$	3.70	3.75	3.70	
Cyclopropene	$1\ ^1B_1$	6.76	6.76	6.76	
Cyclopropene	$1\ ^1B_2$	7.06	7.06	7.06	
Cyclopropene	$1\ ^3B_2$	4.35	4.35	4.35	
Cyclopropene	$1\ ^3B_1$	6.51	6.51	6.51	
Cyclopentadiene	$1\ ^1B_2$	5.51	5.67	5.51	
Cyclopentadiene	$2\ ^1A_1$	6.31	6.31	6.15	
Cyclopentadiene	$3\ ^1A_1$	8.52	8.52	8.37	
Cyclopentadiene	$1\ ^3B_2$	3.28	3.44	3.28	
Cyclopentadiene	$1\ ^3A_1$	5.10	5.26	5.11	
Norbornadiene	$1\ ^1A_2$	5.34	5.56	5.34	
Norbornadiene	$1\ ^1B_2$	6.11	6.33	6.11	
Norbornadiene	$2\ ^1B_2$	7.32	7.54	7.32	
Norbornadiene	$2\ ^1A_2$	7.44	7.67	7.45	
Norbornadiene	$2\ ^1A_1$	7.97	7.97	7.76	
Norbornadiene	$1\ ^3A_2$	3.75	3.97	3.75	
Norbornadiene	$1\ ^3B_2$	4.22	4.44	4.22	
Benzene	$2\ ^1A'$	5.05	5.04	5.01	
Benzene	$3\ ^1A'$	6.44	6.44	6.42	
Benzene	$4\ ^1A'$	7.07	7.13	7.11	
Benzene	$5\ ^1A'$	7.07	7.14	7.11	
Benzene	$6\ ^1A'$	8.21	8.17	8.15	
Benzene	$7\ ^1A'$	8.21	8.20	8.17	
Benzene	$1\ ^3A'$	4.17	4.17	4.15	MS instead of SS
Benzene	$2\ ^3A'$	4.90	4.90	4.87	MS instead of SS
Benzene	$3\ ^3A'$	4.90	4.90	4.88	MS instead of SS
Benzene	$4\ ^3A'$	5.76	5.77	5.75	MS instead of SS
Benzene	$5\ ^3A'$	7.41	7.37	7.35	MS instead of SS
Benzene	$6\ ^3A'$	7.41	7.38	7.36	MS instead of SS
Naphthalene	$1\ ^1B_{3u}$	4.24	4.27	4.24	
Naphthalene	$1\ ^1B_{2u}$	4.77	4.80	4.77	
Naphthalene	$2\ ^1A_g$	5.90	5.87	5.84	
Naphthalene	$1\ ^1B_{1g}$	6.00	6.02	5.99	
Naphthalene	$2\ ^1B_{3u}$	6.07	6.09	6.06	
Naphthalene	$2\ ^1B_{1g}$	6.48	6.51	6.48	
Naphthalene	$2\ ^1B_{2u}$	6.33	6.36	6.33	

Table S7: . . . continued

**Molecule**	**State**	$\mathbf{V_i}$(Thiel SI)[a]	$\mathbf{V_i}$(MS)[b]	$\mathbf{V_i}$(SS)[c]	**Comment**
Naphthalene	$3\ ^1A_g$	6.71	6.67	6.64	
Naphthalene	$3\ ^1B_{2u}$	8.18	8.20	8.18	
Naphthalene	$3\ ^1B_{3u}$	7.76	7.78	7.75	
Naphthalene	$1\ ^3B_{2u}$	3.20	3.20	3.17	MS instead of SS
Naphthalene	$1\ ^3B_{3u}$	4.29	4.29	4.26	MS instead of SS
Naphthalene	$1\ ^3B_{1g}$	4.55	4.55	4.52	MS instead of SS
Naphthalene	$2\ ^3B_{2u}$	4.71	4.71	4.68	MS instead of SS
Naphthalene	$2\ ^3B_{3u}$	5.00	5.00	4.97	MS instead of SS
Naphthalene	$1\ ^3A_g$	5.57	5.56	5.53	MS instead of SS
Naphthalene	$2\ ^3B_{1g}$	6.25	6.24	6.21	MS instead of SS
Naphthalene	$2\ ^3A_g$	6.42	6.42	6.39	MS instead of SS
Naphthalene	$3\ ^3A_g$	6.63	6.62	6.59	MS instead of SS
Naphthalene	$3\ ^3B_{1g}$	6.67	6.67	6.64	MS instead of SS
Furan	$1\ ^1B_2$	6.43	6.57	6.39	
Furan	$2\ ^1A_1$	6.52	6.50	6.32	
Furan	$3\ ^1A_1$	8.22	8.17	7.99	
Furan	$1\ ^3B_2$	4.17	4.35	4.18	
Furan	$1\ ^3A_1$	5.49	5.67	5.49	
Pyrrole	$2\ ^1A_1$	6.31	6.31	6.24	
Pyrrole	$1\ ^1B_2$	6.33	6.40	6.33	
Pyrrole	$3\ ^1A_1$	8.18	8.17	8.10	
Pyrrole	$1\ ^3B_2$	4.52	4.58	4.51	
Pyrrole	$1\ ^3A_1$	5.53	5.59	5.53	
Imidazole	$1\ ^1A''$	6.81	6.87	6.81	active space (1 5)8
Imidazole	$2\ ^1A'$	6.19	6.19	6.12	active space (1 5)8
Imidazole	$3\ ^1A'$	6.93	6.93	6.86	active space (1 5)8
Imidazole	$2\ ^1A''$	7.91	7.97	7.91	active space (1 5)8
Imidazole	$4\ ^1A'$	8.15	8.16	8.09	active space (1 5)8
Imidazole	$1\ ^3A'$	4.65	4.72	4.65	active space (1 5)8
Imidazole	$2\ ^3A'$	5.74	5.80	5.74	active space (1 5)8
Imidazole	$1\ ^3A''$	6.36	6.42	6.36	active space (1 5)8
Imidazole	$3\ ^3A'$	6.44	6.51	6.44	active space (1 5)8
Imidazole	$4\ ^3A'$	7.43	7.50	7.44	active space (1 5)8
Imidazole	$2\ ^3A''$	7.51	7.58	7.51	active space (1 5)8
Pyridine	$1\ ^1B_2$	5.02	5.08	5.04	
Pyridine	$2\ ^1A_1$	6.39	6.39	6.35	
Pyridine	$3\ ^1A_1$	7.46	7.46	7.42	
Pyridine	$2\ ^1B_2$	7.29	7.33	7.30	
Pyridine	$4\ ^1A_1$	8.70	8.69	8.66	

Table S7: ... continued

**Molecule**	**State**	$\mathbf{V_i}$(Thiel SI)[a]	$\mathbf{V_i}$(MS)[b]	$\mathbf{V_i}$(SS)[c]	**Comment**
Pyridine	$3\ ^1B_2$	8.62	8.66	8.62	
Pyridine	$1\ ^1B_1$	5.14	5.19	5.16	
Pyridine	$1\ ^1A_2$	5.47	5.53	5.50	
Pyridine	$1\ ^3A_1$	4.27	4.30	4.27	
Pyridine	$1\ ^3B_2$	4.72	4.75	4.72	
Pyridine	$2\ ^3A_1$	5.03	5.07	5.03	
Pyridine	$2\ ^3B_2$	6.02	6.06	6.03	
Pyridine	$3\ ^3A_1$	7.56	7.59	7.56	
Pyridine	$3\ ^3B_2$	7.88	7.91	7.88	
Pyridine	$1\ ^3B_1$	4.55	4.59	4.55	
Pyridine	$1\ ^3A_2$	5.48	5.54	5.50	
Pyrazine	$1\ ^1B_{2u}$	4.85	4.88	4.86	
Pyrazine	$1\ ^1B_{1u}$	6.89	6.92	6.89	
Pyrazine	$2\ ^1B_{1u}$	7.79	7.82	7.79	
Pyrazine	$2\ ^1B_{2u}$	7.65	7.68	7.66	
Pyrazine	$1\ ^1B_{3g}$	8.47	8.50	8.47	
Pyrazine	$2\ ^1A_g$	8.61	8.61	8.58	
Pyrazine	$1\ ^1B_{3u}$	4.12	4.14	4.12	
Pyrazine	$1\ ^1A_u$	4.70	4.72	4.69	
Pyrazine	$1\ ^1B_{2g}$	5.68	5.70	5.68	
Pyrazine	$1\ ^1B_{1g}$	6.41	6.43	6.41	
Pyrimidine	$1\ ^1B_2$	5.24	5.31	5.24	
Pyrimidine	$2\ ^1A_1$	6.64	6.63	6.56	
Pyrimidine	$3\ ^1A_1$	7.21	7.21	7.14	
Pyrimidine	$2\ ^1B_2$	7.64	7.71	7.64	
Pyrimidine	$3\ ^1B_2$	8.73	8.81	8.74	
Pyrimidine	$4\ ^1A_1$	9.19	9.19	9.12	
Pyrimidine	$1\ ^1B_1$	4.44	4.51	4.44	
Pyrimidine	$1\ ^1A_2$	4.81	4.87	4.80	
Pyridazine	$2\ ^1A_1$	5.18	5.18	5.09	active space (1 1 3 3)10
Pyridazine	$1\ ^1B_2$	6.31	6.40	6.31	active space (1 1 3 3)10
Pyridazine	$2\ ^1B_2$	7.29	7.38	7.29	active space (1 1 3 3)10
Pyridazine	$3\ ^1A_1$	7.62	7.62	7.53	active space (1 1 3 3)10
Pyridazine	$1\ ^1B_1$	3.78	3.87	3.78	active space (1 1 3 3)10
Pyridazine	$1\ ^1A_2$	4.32	4.40	4.31	active space (1 1 3 3)10
Pyridazine	$2\ ^1A_2$	5.77	5.86	5.77	active space (1 1 3 3)10
Pyridazine	$2\ ^1B_1$	6.52	6.60	6.52	active space (1 1 3 3)10
Triazine	$2\ ^1A'$	5.79	5.79	5.64	
Triazine	$3\ ^1A'$	7.25	7.26	7.11	

Table S7: ... continued

Molecule	State	$\mathbf{V_i}$(Thiel SI)[a]	$\mathbf{V_i}$(MS)[b]	$\mathbf{V_i}$(SS)[c]	Comment
Triazine	$4\ ^1A'$	7.49	7.50	7.35	
Triazine	$5\ ^1A'$	8.99	8.95	8.80	
Triazine	$1\ ^1A''$	4.60	4.74	4.59	
Triazine	$2\ ^1A''$	4.66	4.81	4.66	
Triazine	$3\ ^1A''$	4.71	4.85	4.70	
Triazine	$4\ ^1A''$	4.71	4.85	4.70	
Triazine	$5\ ^1A''$	7.72	7.86	7.71	
Triazine	$6\ ^1A''$	7.72	7.86	7.71	
Tetrazine	$1\ ^1B_{2u}$	4.91	4.94	4.93	active space (1 2 1 1 1 1 1 2)14
Tetrazine	$1\ ^1B_{1u}$	6.96	6.95	6.94	active space (1 2 1 1 1 1 1 2)14
Tetrazine	$2\ ^1B_{1u}$	7.43	7.43	7.42	active space (1 2 1 1 1 1 1 2)14
Tetrazine	$2\ ^1B_{2u}$	8.15	7.91	7.90	significant difference
Tetrazine	$2\ ^1B_{3g}$	8.32	7.86	7.85	significant difference
Tetrazine	$3\ ^1A_g$	8.97	8.03	8.02	significant difference
Tetrazine	$1\ ^1B_{3u}$	2.24	2.30	2.29	significant difference
Tetrazine	$1\ ^1A_u$	3.48	3.52	3.51	active space (1 2 1 1 1 1 1 2)14
Tetrazine	$2\ ^1A_g$	4.55	4.66	4.65	significant difference
Tetrazine	$1\ ^1B_{1g}$	4.73	4.74	4.73	active space (1 2 1 1 1 1 1 2)14
Tetrazine	$1\ ^1B_{2g}$	5.18	5.20	5.19	active space (1 2 1 1 1 1 1 2)14
Tetrazine	$1\ ^1B_{3g}$	5.79	5.87	5.86	significant difference
Tetrazine	$2\ ^1A_u$	5.47	5.50	5.49	active space (1 2 1 1 1 1 1 2)14
Tetrazine	$2\ ^1B_{2g}$	6.07	6.07	6.06	active space (1 2 1 1 1 1 1 2)14
Tetrazine	$2\ ^1B_{1g}$	6.38	6.46	6.45	significant difference
Tetrazine	$2\ ^1B_{3u}$	6.77	6.78	6.77	active space (1 2 1 1 1 1 1 2)14
Tetrazine	$3\ ^1B_{1g}$	6.74	6.73	6.72	active space (1 2 1 1 1 1 1 2)14
Tetrazine	$1\ ^3B_{3u}$	1.56	1.62	1.61	significant difference
Tetrazine	$1\ ^3A_u$	3.26	3.29	3.28	active space (1 2 1 1 1 1 1 2)14
Tetrazine	$1\ ^3B_{1g}$	4.14	4.14	4.13	active space (1 2 1 1 1 1 1 2)14
Tetrazine	$1\ ^3B_{1u}$	4.36	4.38	4.37	active space (1 2 1 1 1 1 1 2)14
Tetrazine	$1\ ^3B_{2u}$	4.56	4.40	4.39	significant difference
Tetrazine	$1\ ^3B_{2g}$	4.93	4.95	4.94	active space (1 2 1 1 1 1 1 2)14
Tetrazine	$2\ ^3A_u$	5.02	5.05	5.04	active space (1 2 1 1 1 1 1 2)14
Tetrazine	$1\ ^3B_{3g}$	5.50	7.62	7.61	significant difference
Tetrazine	$2\ ^3B_{1u}$	5.40	5.41	5.40	active space (1 2 1 1 1 1 1 2)14
Tetrazine	$2\ ^3B_{2g}$	5.97	5.97	5.96	active space (1 2 1 1 1 1 1 2)14
Tetrazine	$2\ ^3B_{1g}$	6.31	6.38	6.37	significant difference
Tetrazine	$2\ ^3B_{3u}$	6.54	6.55	6.54	active space (1 2 1 1 1 1 1 2)14
Tetrazine	$2\ ^3B_{2u}$	7.10	6.32	6.31	significant difference
Formaldehyde	$1\ ^1A_2$	3.98	4.14	3.99	

Table S7: ... continued

**Molecule**	**State**	$\mathbf{V_i}$(Thiel SI)[a]	$\mathbf{V_i}$(MS)[b]	$\mathbf{V_i}$(SS)[c]	**Comment**
Formaldehyde	$1\ ^1B_1$	9.14	9.29	9.13	
Formaldehyde	$2\ ^1A_1$	9.31	9.32	9.16	
Formaldehyde	$1\ ^3A_2$	3.58	3.74	3.58	
Formaldehyde	$1\ ^3A_1$	5.84	5.99	5.84	
Acetone	$1\ ^1A_2$	4.42	4.68	4.43	
Acetone	$1\ ^1B_1$	9.27	10.65	10.41	$V_i = 9.28$ eV with (1 2 0 1) active space
Acetone	$2\ ^1A_1$	9.31	9.29	9.05	
Acetone	$1\ ^3A_2$	4.08	4.34	4.10	
Acetone	$1\ ^3A_1$	6.03	6.28	6.04	
Benzoquinone	$1\ ^1B_{1g}$	2.78	2.76	2.76	
Benzoquinone	$1\ ^1A_u$	2.80	2.77	2.77	
Benzoquinone	$1\ ^1B_{3g}$	4.25	4.25	4.26	
Benzoquinone	$2\ ^1A_g$	4.51	4.49	4.49	
Benzoquinone	$1\ ^1B_{2g}$	5.66	5.64	5.65	
Benzoquinone	$1\ ^1B_{1u}$	5.29	5.14	5.14	significant difference
Benzoquinone	$1\ ^1B_{3u}$	5.60	5.64	5.64	
Benzoquinone	$2\ ^1B_{2g}$	6.60	6.59	6.59	
Benzoquinone	$2\ ^1B_{1g}$	6.15	6.13	6.13	
Benzoquinone	$2\ ^1A_u$	6.05	6.03	6.04	
Benzoquinone	$3\ ^1A_g$	6.08	6.06	6.07	
Benzoquinone	$2\ ^1B_{3g}$	6.98	6.96	6.96	
Benzoquinone	$1\ ^1B_{2u}$	7.32	7.32	7.32	
Benzoquinone	$2\ ^1B_{1u}$	7.91	7.04	7.05	significant difference
Benzoquinone	$3\ ^1B_{1u}$	7.35	7.58	7.58	significant difference
Benzoquinone	$3\ ^1B_{1g}$	7.90	7.88	7.88	
Benzoquinone	$3\ ^1A_u$	7.90	7.89	7.89	
Benzoquinone	$4\ ^1A_g$	8.07	8.05	8.05	
Benzoquinone	$5\ ^1A_g$	8.11	8.11	8.11	
Benzoquinone	$1\ ^3B_{1g}$	2.63	2.62	2.62	
Benzoquinone	$1\ ^3A_u$	2.68	2.66	2.66	
Benzoquinone	$1\ ^3B_{1u}$	2.99	2.98	2.99	
Benzoquinone	$1\ ^3B_{3g}$	3.31	3.32	3.32	
Formamide	$1\ ^1A''$	5.63	5.76	5.63	
Formamide	$2\ ^1A'$	7.44	7.39	7.26	
Formamide	$3\ ^1A'$	10.54	10.54	10.41	
Formamide	$1\ ^3A''$	5.40	5.52	5.40	
Formamide	$1\ ^3A'$	5.58	5.81	5.68	significant difference
Acetamide	$1\ ^1A''$	5.80	5.81	5.69	MS instead of SS
Acetamide	$2\ ^1A'$	7.27	7.27	7.15	

Table S7: ... continued

**Molecule**	**State**	$\mathbf{V_i}$(Thiel SI)[a]	$\mathbf{V_i}$(MS)[b]	$\mathbf{V_i}$(SS)[c]	**Comment**
Acetamide	$3\ ^1A'$	10.09	10.09	9.97	
Acetamide	$1\ ^3A''$	5.53	5.54	5.41	MS instead of SS
Acetamide	$1\ ^3A'$	5.75	5.76	5.63	MS instead of SS
Propanamide	$1\ ^1A''$	5.72	5.84	5.72	
Propanamide	$2\ ^1A'$	7.20	7.20	7.08	
Propanamide	$3\ ^1A'$	9.94	9.94	9.82	
Propanamide	$1\ ^3A''$	5.44	5.56	5.44	
Propanamide	$1\ ^3A'$	5.79	5.86	5.80	
Cytosine	$2\ ^1A'$	4.68	4.67	4.43	
Cytosine	$1\ ^1A''$	5.12	5.37	5.12	
Cytosine	$3\ ^1A'$	5.54	5.53	5.29	
Cytosine	$4\ ^1A'$	6.40	6.40	6.16	
Cytosine	$2\ ^1A''$	5.54	5.78	5.54	
Cytosine	$5\ ^1A'$	6.98	6.97	6.72	
Cytosine	$6\ ^1A'$	8.23	8.22	7.98	
Thymine	$1\ ^1A''$	4.94	5.21	4.95	
Thymine	$2\ ^1A'$	5.06	5.06	4.81	
Thymine	$3\ ^1A'$	6.15	6.15	5.90	
Thymine	$2\ ^1A''$	6.38	6.64	6.39	
Thymine	$4\ ^1A'$	6.52	6.53	6.28	
Thymine	$3\ ^1A''$	6.86	7.11	6.86	
Thymine	$4\ ^1A''$	7.43	7.69	7.44	
Thymine	$5\ ^1A'$	7.43	7.43	7.18	
Thymine	$6\ ^1A'$	8.48	8.48	8.23	
Uracil	$1\ ^1A''$	4.91	5.18	4.91	
Uracil	$2\ ^1A'$	5.23	5.23	4.96	
Uracil	$3\ ^1A'$	6.15	6.15	5.89	
Uracil	$2\ ^1A''$	6.27	6.54	6.28	
Uracil	$3\ ^1A''$	6.97	7.25	6.99	
Uracil	$4\ ^1A'$	6.75	6.74	6.48	
Uracil	$4\ ^1A''$	7.28	7.55	7.28	
Uracil	$5\ ^1A'$	7.42	7.42	7.15	
Adenine	$2\ ^1A'$	5.20	5.20	5.05	
Adenine	$3\ ^1A'$	5.29	5.29	5.14	
Adenine	$1\ ^1A''$	5.21	5.34	5.19	
Adenine	$4\ ^1A'$	6.35	6.34	6.20	
Adenine	$5\ ^1A'$	6.64	6.64	6.50	
Adenine	$2\ ^1A''$	5.97	6.10	5.96	
Adenine	$6\ ^1A'$	6.88	6.87	6.73	

Table S7: ... continued

Molecule	State	$\mathbf{V_i}$(Thiel SI)[a]	$\mathbf{V_i}$(MS)[b]	$\mathbf{V_i}$(SS)[c]	Comment
Adenine	$7\ ^1A'$	7.56	7.56	7.41	

[a] vertical excitation energy in eV reported in the SI of Ref. 51

[b] vertical excitation energy in eV with respect to the MS-CASPT2 ground state energy

[c] vertical excitation energy in eV with respect to the ground state calculated separately using SS-CASPT2

S3.2 Ground State Energies of all Molecules

In Table S8 the ground state energies of all the molecules comprising the Thiel benchmark set[51] are given. SS-CASPT2 energies are obtained from calculations separately performed for the ground state. MS-CASPT2 energies refer to the standard calculations considering also the totally-symmetric excited state. For ethene and cyclopropene, only one totally symmetric state was considered in the standard calculations, i.e., $E_0(\mathrm{SS}) = E_0(\mathrm{MS})$. For all other molecules $\Delta E = E_0(\mathrm{SS}) - E_0(\mathrm{MS}) < 0$ and in average $\Delta E = -0.11$ and -0.12 eV for IPEA and NOIPEA, respectively.

Table S8: Multi-state (MS) and single-state (SS) CASPT2 ground state energies E_0 in a.u. and energy differences $\Delta E = E_0(\mathrm{SS}) - E_0(\mathrm{MS})$ in eV computed using the standard (NOIPEA) and the IPEA-shifted CASPT2 schemes of the 28 organic molecules comprising the Thiel set.

CASPT2	IPEA			NOIPEA		
**Molecule**	$\mathbf{E_0}$**(MS)**	$\mathbf{E_0}$**(SS)**	$\mathbf{\Delta E}$	$\mathbf{E_0}$**(MS)**	$\mathbf{E_0}$**(SS)**	$\mathbf{\Delta E}$
Ethene	−78.351896	−78.351896	0	−78.354406	−78.354406	0
Butadiene	−155.539670	−155.537966	−0.05	−155.542562	−155.540620	−0.05
Hexatriene	−232.725149	−232.724049	−0.03	−232.729251	−232.727925	−0.04
Octatetraene	−309.907851	−309.906038	−0.05	−309.913359	−309.911202	−0.06
Cyclopropene	−116.281456	−116.281456	0	−116.284351	−116.284351	0
Cyclopentadiene	−193.554776	−193.549162	−0.15	−193.559650	−193.552062	−0.21
Norbornadiene	−270.730945	−270.722900	−0.22	−270.738004	−270.725892	−0.33
Benzene	−231.589659	−231.588814	−0.02	−231.593579	−231.592899	−0.02
Naphthalene	−384.806176	−384.805100	−0.03	−384.813351	−384.812090	−0.03
Furan	−229.450981	−229.444412	−0.18	−229.455435	−229.447597	−0.21
Pyrrole	−209.607140	−209.604619	−0.07	−209.610722	−209.607760	−0.08
Imidazole	−225.643731	−225.641324	−0.07	−225.646814	−225.641830	−0.14
Pyridine	−247.612808	−247.611572	−0.03	−247.617116	−247.615943	−0.03
Pyrazine	−263.632297	−263.631332	−0.03	−263.637382	−263.636169	−0.03
Pyrimidine	−263.640807	−263.638098	−0.07	−263.645589	−263.642647	−0.08
Pyridazine	−263.603223	−263.599990	−0.09	−263.608403	−263.605167	−0.09

Table S8: ... continued

**CASPT2**	**IPEA**			**NOIPEA**		
**Molecule**	**E_0(MS)**	**E_0(SS)**	**ΔE**	**E_0(MS)**	**E_0(SS)**	**ΔE**
Triazine	−279.673849	−279.668333	−0.15	−279.679333	−279.672942	−0.17
Tetrazine	−295.607594	−295.607229	−0.01	−295.613885	−295.613196	−0.02
Formaldehyde	−114.248428	−114.242714	−0.16	−114.253290	−114.245651	−0.21
Acetone	−192.666642	−192.657638	−0.24	−192.659680	−192.658967	−0.02
Benzoquinone	−380.522020	−380.522177	−0.00	−380.529017	−380.527491	−0.04
Formamide	−169.510604	−169.505774	−0.13	−169.514406	−169.508770	−0.15
Acetamide	−208.714964	−208.710485	−0.12	−208.718584	−208.713392	−0.14
Propanamide	−247.912175	−247.907767	−0.12	−247.915757	−247.910683	−0.14
Cytosine	−393.989164	−393.980088	−0.25	−393.993994	−393.983402	−0.29
Thymine	−453.066735	−453.057455	−0.25	−453.070870	−453.060908	−0.27
Uracil	−413.864525	−413.854729	−0.27	−413.868261	−413.858021	−0.28
Adenine	−466.175499	−466.170173	−0.14	−466.181942	−466.174967	−0.19

S3.3 Additional Computational Details

All calculations performed for the molecules in Thiel's benchmark set used the same parameters as given in the Supporting Information of Ref. 51 with the exceptions of imidazole, pyridazine, and tetrazine. For these molecules, the number of orbitals or number of electrons in the active spaces were reported erroneously and we use the active spaces that we believe had been intended to use (see section S3.1). For all molecules, calculations have been performed using the IPEA shift values $\varepsilon = -0.12$, 0, 0.08, 0.1337, 0.16, and 0.25 for the TZVP basis set[52] and shift values of $\varepsilon = 0$, 0.1, 0.2, 0.25, 0.3, 0.4 and 0.5 a.u. for the ANO-RCC basis set,[53] respectively.

S3.4 Intruder State Problems

S3.4.1 General Considerations

In Table S10 in section S3.5, we present the CASPT2 vertical excitation computed using the TZVP basis set and different values of the IPEA shift. With the exception of the IPEA shift value, all calculations used the same CASPT2 parameters, e.g., level shift or number of states considered for each irreducible representation, as in the original benchmark study by Thiel et al.[51] Using these parameters and the recommended IPEA shift value of $\varepsilon = 0.25$ a.u., all

calculations are free from intruder state problems. When the IPEA shift value is decreased, however, intruder state problems occurred in some cases. To understand this, we note that we consider only low-lying excited states. For these states, the energy difference to the first-order interaction space states, that appears in the denominator of the CASPT2 second-order energy correction, is usually positive (see section 2.1 in the main paper). Adding a positive energy contribution due to the IPEA shift increases these denominators. The IPEA shift, thus, partially takes the role of other shift techniques such as the level shift in damping the coupling to intruder states. The impact, however, is different for the states and depends on their character. When decreasing the IPEA shift value, the denominators in the second-order energy correction for some of the states will become smaller again, which, in the cases of denominators very close to zero, gives rise to intruder states. Since we do not want to change any other CASPT2 parameter of Thiel's benchmark calculations for the sake of comparability –except the IPEA shift value –we need to exclude a number of states from our statistical evaluation due to intruder state problems, and we will discuss the individual cases in sections S3.4.2 and S3.4.3. Note that, from the beginning, our intention was to only consider the states reported in the Supporting Information of Ref. 51 although for some molecules, more states are computed.

The presence of intruder states was recognized by comparing the reference weights of the CASSCF wave function in the CASPT2 wave function. A reference weight that is significantly lower than that of the ground state can indicate intruder state problems. In the MOLCAS manual, when describing the investigation of the excited states of thiophene as an exemplary CASPT2 study, it is suggested that a difference of 10 % may be seen as significant when no level shift is used. We note that this suggestion seems to apply to the case where no IPEA shift is used as it was already presented for MOLCAS versions previous the MOLCAS6.4. Using a non-zero IPEA shift can already damp the coupling to intruder states, and, thus, an even smaller difference in the reference weights may have to be used as an indicator of the presence of intruder states when employing an IPEA-shifted CASPT2 variant. However, we emphasize at this stage that one should not use the IPEA shift technique as a substitute for the level shift technique. For both, the IPEA and the level shift technique, a "small" energy contribution is added to the energy difference of the reference state and the energies of the FOI space states. This energy contribution can damp the coupling to potential intruder states which is the reason why the level shift technique was introduced. Here, however, the size of the shift

does not possess any physical motivation. The addition of the energy contribution is at the cost of a change in the energy of the state. Using the level-shift as implemented in MOLCAS, this unphysical change in energy is removed –approximately at least –once the coupling with the intruder states is reduced to be conveniently small. The IPEA shift technique was not introduced to remove the coupling with intruder states but to correct for a proposed systematic underestimation of energies of open-shell states. Consequently, the energy shift introduced when using the IPEA-modified CASPT2 variant is not removed and the energy contributions due to the shift are not added evenly to all states (see section 2.2 in the main paper).

In the following, we report the individual cases of intruder state problems that appeared in the CASPT2 calculations using different IPEA shift values. For the case $\varepsilon = 0$, we will give detailed discussions in section S3.4.2 on how we handled each problem individually to establish a pattern of action. In section S3.4.3, we only report the individual states that we excluded from our data set due to intruder state problems for the cases $0 < \varepsilon < 0.25$ a.u., and we briefly comment on the case of $\varepsilon = -0.12$ a.u.

S3.4.2 Intruder State Problems in NOIPEA-CASPT2 Calculations

Hexatriene The SS-CASPT2 1 ^1B_u state of hexatriene possesses only a reference weight of $\omega = 0.62$, while for the ground state $\omega(1\ ^1A_g) = 0.79$. This indicates intruder-state problems in the description of the 1 ^1B_u SS-CASPT2 state. At MS-CASPT2 level, this states changes in order with the 2 ^1B_u SS-CASPT2 state (only two states were calculated in ^1B_u symmetry). For our data set, we are only interested in the lowest-energy MS-CASPT2 state of this symmetry, i.e., the 2 ^1B_u SS-CASPT2 state. We show the matrix of ^1B_u MS-CASPT2 state vectors in eq. (S3). Looking at the state vectors, one sees that both ^1B_u SS-CASPT2 states mix at MS-CASPT2 level and the coefficient of the first SS-CASPT2 state in the state vector of the 1 ^1B_u MS-CASPT2 state amounts to $c = 0.15$ (corresponding to a weight of $|c|^2 = 2$ %). However, the difference in the total energies between the second SS-CASPT2 and the first MS-CASPT2 root is only 0.02 eV so that the mixing does not affect the energy significantly. Thus, we keep the 1 ^1B_u state in our data set.

$$^1B_u : \quad \begin{pmatrix} 0.1493 & -0.9887 \\ -0.9887 & -0.1493 \end{pmatrix} \tag{S3}$$

Norbornadiene In the SS-CASPT2 calculations of norbornadiene, the 3 $^1A_1$ state possesses a reference weight of only $\omega = 0.51$ while for the ground state $\omega(1\ ^1A_1) = 0.75$. Unfortunately, the 3 $^1A_1$ state notably mixes with the ground state at MS-CASPT2 level [see eq. (S4)], resulting in an energy change of -0.28 eV for the ground state when going from SS-CASPT2 to MS-CASPT2. Since the energy of the ground state may be corrupted by the presence of the worse described 3 $^1A_1$ state, we have to exclude all states of the norbornadiene molecule.

$$^1A_1: \quad \begin{pmatrix} -0.9850 & -0.0790 & 0.1533 \\ 0.0268 & -0.9483 & -0.3160 \\ -0.1704 & 0.3071 & -0.9362 \end{pmatrix} \tag{S4}$$

Benzene The CASPT2 calculations of benzene were conducted in the reduced C_s point-group symmetry. In the SS-CASPT2 calculations, we find low reference weights for the states 4 $^1A'$ (0.28), 5 $^1A'$ (0.23), 4 $^3A'$ (0.00), and 5 $^3A'$ (0.02). However, none of these states shows considerable mixing with the SS-CASPT2 of the same symmetry at the multi-state level [see eqs. (S5) and (S6)], so that the other states are not affected by their presence. The largest coefficient of any of these states in the MS-CASPT2 state vector is only $c = 0.01$. Thus, we will exclude the badly described states but keep all the other states in our data set.

$$^1A': \quad \begin{pmatrix} 0.9999 & -0.0000 & 0.0000 & 0.0000 & 0.0000 & -0.0109 & -0.0000 \\ 0.0000 & 0.9999 & 0.0000 & -0.0000 & 0.0036 & -0.0000 & 0.0000 \\ 0.0000 & 0.0000 & -0.9987 & 0.0493 & 0.0000 & -0.0000 & -0.0000 \\ -0.0000 & 0.0000 & 0.0000 & 0.0000 & -0.0000 & 0.0000 & -1.0000 \\ 0.0109 & 0.0000 & -0.0000 & -0.0000 & 0.0000 & 0.9999 & 0.0000 \\ 0.0000 & 0.0036 & -0.0000 & 0.0000 & -0.9999 & 0.0000 & 0.0000 \\ -0.0000 & 0.0000 & 0.0493 & 0.9987 & 0.0000 & 0.0000 & 0.0000 \end{pmatrix} \tag{S5}$$

$$^3A': \quad \begin{pmatrix} -0.9999 & 0.0000 & -0.0000 & -0.0099 & -0.0000 & -0.0000 \\ -0.0000 & -0.0000 & 0.9999 & -0.0000 & -0.0024 & -0.0000 \\ -0.0099 & 0.0000 & 0.0000 & 0.9999 & 0.0000 & -0.0000 \\ 0.0000 & 1.0000 & 0.0000 & -0.0000 & -0.0000 & -0.0000 \\ -0.0000 & 0.0000 & 0.0000 & 0.0000 & 0.0000 & 1.0000 \\ -0.0000 & -0.0000 & -0.0024 & 0.0000 & -0.9999 & 0.0000 \end{pmatrix} \tag{S6}$$

Naphthalene For naphthalene, two states with low reference weights were found in the calculations of the $^1B_{2u}$ states, the SS-CASPT2 states 3 $^1B_{2u}$ ($\omega = 0.28$) and 4 $^1B_{2u}$ ($\omega = 0.14$). These states again show only minor mixing with the other SS-CASPT2 states at the MS-

CASPT2 level [see eq. (S7)]. Since we are only interested in describing the three other excited states, we can safely include these into our data set. For the purpose of illustration we report the change in the total energy of these three states when going from SS-CASPT2 to MS-CASPT2 treatment: 0.03, 0.04, and 0.07 eV for SS-CASPT2 roots 1, 2, and 5, respectively.

$$
{}^1B_{2u}: \quad \begin{pmatrix} -0.0044 & 0.0034 & 0.9930 & -0.0935 & -0.0705 \\ 0.0026 & -0.0149 & 0.0800 & 0.9811 & -0.1750 \\ 0.0283 & 0.9994 & -0.0017 & 0.0156 & 0.0019 \\ 0.9995 & -0.0283 & 0.0046 & -0.0027 & 0.0044 \\ -0.0044 & -0.0042 & 0.0855 & 0.1682 & 0.9820 \end{pmatrix} \tag{S7}
$$

Furan For furan, the 3 $^1B_2$ SS-CASPT2 state possesses a reference weight of only $\omega = 0.59$. It remains the 3 $^1B_2$ state in the multi-state treatment [see eq. (S8)]. We are only interested in describing the 1 $^1B_2$ state, which shows only a mixing with the 3 $^1B_2$ state, however, both states mix with the 2 $^1B_2$ state yielding energy changes when going from SS-CASPT2 to MS-CASPT2 of −0.15, −0.06, and 0.21 eV for the roots 1, 2, and 3, respectively. Since the energy change for the 1 $^1B_2$ state is notable and we cannot know how much of it is caused by the indirect mixing to the 3 $^1B_2$ state, we will exclude the 1 $^1B_2$ state from our data set.

$$
{}^1B_2: \quad \begin{pmatrix} 0.9693 & 0.2456 & 0.0096 \\ -0.2398 & 0.9361 & 0.2570 \\ 0.0541 & -0.2515 & 0.9663 \end{pmatrix} \tag{S8}
$$

Pyrrole In the calculation of the $^1B_2$ states of pyrrole, the third root showed a very small reference weight close to $\omega = 0.00$. Since this state mixes with the other two states of $^1B_2$ symmetry at the multi-state CASPT2 level [see eq. (S9)], we exclude the 1 $^1B_2$ state of pyrrole from our analysis.

$$
{}^1B_2: \quad \begin{pmatrix} 0.9549 & -0.1941 & -0.2246 \\ 0.2579 & 0.9170 & 0.3039 \\ 0.1469 & -0.3481 & 0.9258 \end{pmatrix} \tag{S9}
$$

Imidazole For imidazole, two excited $^1A'$ states show notably smaller reference weights than the ground state: $\omega(4\ ^1A') = 0.62$ and $\omega(5\ ^1A') = 0.57$ whereas $\omega(1\ ^1A') = 0.81$. These two states show only very small mixing with the ground state [see eq. (S10)] and the total energy of the ground state changes only by 0.01 eV when going from SS-CASPT2 to MS-CASPT2. Thus, the ground state is rather unaffected by the presence of the two worse described states.

The other $^1A'$ excited states show non-negligible mixing and there energy changes by 0.2-0.6 eV, and we, thus, exclude all $^1A'$ excited states from our data set. Yet, we keep the ground state (1 $^1A'$) in our data allowing us to also keep the other states ($^1A''$, $^3A'$, and $^3A''$).

$$^1A' : \begin{pmatrix} -0.9990 & -0.0094 & 0.0340 & -0.0183 & 0.0151 \\ -0.0292 & 0.6566 & -0.7306 & 0.0450 & 0.1792 \\ 0.0126 & 0.6447 & 0.6491 & 0.3513 & 0.1981 \\ 0.0255 & 0.2201 & 0.2019 & -0.9200 & 0.2520 \\ 0.0122 & -0.3235 & -0.0527 & 0.1661 & 0.9299 \end{pmatrix} \tag{S10}$$

Pyridine In pyridine, there are two states that show a small reference weight: the SS-CASPT2 states 3 $^3A_1$ ($\omega = 0.52$) and 2 $^3B_2$ ($\omega = 0.33$). The 3 $^3A_1$ state shows only very small mixing with the other two $^3A_1$ states, so we exclude only the 3 $^3A_1$ state [see eq. (S11)]. The 2 $^3B_2$ state, however, mixes considerably with the other $^3B_2$ states, so that we exclude all $^3B_2$ states from the analysis.

$$^3A_1 : \begin{pmatrix} -0.9823 & -0.1859 & -0.0224 \\ 0.1866 & -0.9817 & -0.0356 \\ -0.0154 & -0.0391 & 0.9991 \end{pmatrix} \tag{S11}$$

$$^3B_2 : \begin{pmatrix} -0.8652 & 0.4972 & 0.0648 \\ -0.2856 & -0.5949 & 0.7512 \\ 0.4121 & 0.6314 & 0.6568 \end{pmatrix} \tag{S12}$$

Pyrazine In pyrazine, the 2 $^1B_{1g}$ exhibits a reference of only $\omega = 0.60$ compared to that of $\omega = 0.80$ of the ground state. Since there is mixing at the MS-CASPT2 level with the 1 $^1B_{1g}$, we exclude the $^1B_{1g}$ states from our data set.

$$^1B_{1g} : \begin{pmatrix} 0.9298 & -0.3678 \\ 0.3678 & 0.9298 \end{pmatrix} \tag{S13}$$

Triazine In the SS-CASPT2 calculations of triazine, two states are found with low reference weights: 5 $^1A''$ ($\omega = 0.13$) and 6 $^1A''$ ($\omega = 0.12$). Since there is only small mixing with the other states of $^1A''$ symmetry [see eq. (S14)], we exclude these two states but keep the rest of

the $^1A''$ states in our data set.

$$^1A'': \quad \begin{pmatrix} -0.0110 & 0.0141 & -0.0059 & -0.9670 & -0.0982 & 0.2339 \\ -0.0032 & -0.0223 & 0.0238 & -0.0224 & 0.9505 & 0.3079 \\ 0.0376 & 0.0194 & -0.0386 & -0.2523 & 0.2937 & -0.9201 \\ 0.0110 & 0.0126 & 0.9988 & -0.0148 & -0.0117 & -0.0409 \\ 0.1270 & 0.9911 & -0.0124 & 0.0179 & 0.0157 & 0.0268 \\ 0.9910 & -0.1278 & -0.0081 & -0.0034 & -0.0110 & 0.0356 \end{pmatrix} \tag{S14}$$

Acetamide For acetamide, we find the 2 $^3A'$ SS-CASPT2 state with a reference weight of $\omega = 0.30$. It shows a small mixing with the 1 $^3A'$ state [see eq. (S15)]. However, since we are only interested in the 1 $^3A'$ state, and its change in the total energy when going from SS-CASPT2 to MS-CASPT2 is only 0.02 eV, we keep the 1 $^3A'$ in our data set.

$$^3A': \quad \begin{pmatrix} 0.9961 & -0.0878 \\ 0.0878 & 0.9961 \end{pmatrix} \tag{S15}$$

Thymine For thymine, we find two SS-CASPT2 states with low reference weights: 3 $^1A''$ ($\omega = 0.55$) and 4 $^1A''$ ($\omega = 0.14$). The 4 $^1A''$ state does not mix with the first two roots, however, the 3 $^1A''$ state does [see eq. (S16)]. Thus, we exclude all $^1A''$ states from our data set.

$$^1A'': \quad \begin{pmatrix} -0.0079 & 0.9381 & 0.3447 & -0.0303 \\ -0.0045 & 0.0743 & -0.2863 & -0.9552 \\ -0.0205 & -0.3381 & 0.8937 & -0.2941 \\ 0.9997 & 0.0008 & 0.0197 & -0.0106 \end{pmatrix} \tag{S16}$$

Adenine For adenine, we find two SS-CASPT2 states with low reference weights: 3 $^1A''$ ($\omega = 0.18$) and 4 $^1A''$ ($\omega = 0.01$). However, since these states do not mix with the other $^1A''$ states [see eq.(S17)] and we are only interested in the first two roots, we do not need to exclude any states of interest from our data set.

$$^1A'': \quad \begin{pmatrix} 0.0004 & -0.0007 & -0.9747 & 0.2231 \\ -0.0001 & -0.0048 & 0.2231 & 0.9747 \\ 0.0008 & 0.9999 & 0.0003 & 0.0048 \\ -0.9999 & 0.0008 & -0.0005 & -0.0000 \end{pmatrix} \tag{S17}$$

S3.4.3 Intruder State Problems in IPEA-CASPT2 Calculations

In Table S9 we show a list of excited states that we exclude from our data set due to possible intruder state problems. These excited states were obtained from CASPT2 calculations using IPEA shifts of $\varepsilon = 0.08$, 0.1337, and 0.16 a.u. In the previous section, we have discussed in detail all intruder state problems that occur when the IPEA shift is set to zero. We note again, that our interest was only directed at states reported in the Supporting Information of Ref. 51 In some cases, the intruder state problem affected only states that did not lie in our range of interest. When there was only negligible coupling with these states at the multi-state CASPT2 level, we felt it safe to keep the rest of states computed in the same calculation in our data set. However, when the coupling was larger or one of states under our consideration was directly affected with an intruder state problem, we excluded these states (Table S9) from our test set (for additional discussion how to handle intruder state problem see section 5.1 in the main paper).

Table S9: Individual states from calculations using IPEA shift values of $\varepsilon = 0.08$, 0.1337, and 0.16 a.u., that were excluded from our data set due to intruder state problems.

Molecule	State	IPEA Shift ε [a.u.]
Octatetraene	$3\ ^1B_u$	0.08
Norbornadiene	all states	0.08, 0.1337, and 0.16
Naphthalene	$n\ ^1B_{3u}$ $(n = 2, 3)$	0.16
Furan	$1\ ^1B_2$	0.08, 0.1337, and 0.16
Pyrrole	$1\ ^1B_2$	0.08, 0.1337, and 0.16
Imidazole	$n\ ^1A'$ $(n = 2 - 4)$	0.08 and 0.16
Pyridine	$3\ ^3A_1$	0.08
Pyrazine	$1\ ^1B_{1g}$	0.08 and 0.1337
Benzoquinone	$2\ ^1A_u$	0.1337 and 0.16
Thymine	$6\ ^1A'$	0.08
Uracil	$n\ ^1A''$ $(n = 1 - 4)$	0.1337

We have also performed calculations with a negative IPEA shift parameter, namely, $\varepsilon = -0.12$ a.u. Here, we could obtain meaningful data only for a very small number of molecules due to significant intruder state problems. The intruder state problem almost always affected also the ground state of the molecule making it necessary to exclude all excited states of most molecules. Thus, instead of listing all the states we had to exclude, we ask the reader to simply

inspect the small number of states reported in Table S10 for $\varepsilon = -0.12$ a.u.

S3.5 CASPT2 Results for Thiel's Benchmark Set

In Table S10 we present the MS-CASPT2 vertical excitation energies of the states included in the Thiel benchmark set obtained using the TZVP basis set and different values of the IPEA shift parameter ε. Except the IPEA shift, all other parameters were adopted from the initial CASPT2 study of Thiel et al.[51] As discussed in the preceding section, the variation of the IPEA shift value from the recommended $\varepsilon = 0.25$ a.u. leads to intruder state problems for a small number of states. Their energies are still reported in Table S10 (marked with an asterisk) but are excluded from any statistical evaluation. Note that we use only MS-CASPT2 energies as the reference energies of the ground states in contrast to the approach by Thiel et al. (see section 5.1 in the main paper). Alongside the MS-CASPT2 vertical excitation energies, we also print the experimental excitation energies compiled by Thiel et al.[51] As was done for our literature survey (see section 3 in the main paper), when several experimental excitation energies were available, we use the average value for comparison.

Table S10: Vertical excitation energies at the MS-CASPT2 level of theory computed using the TVZP basis set and different values of the IPEA shift as well as experimental excitation energies for the 28 organic molecules of the Thiel benchmark set. All energies are given in eV. Experimental energies were adopted from Ref. 51 (see Supporting Information of Ref. 51 for references to the original publications). Entries marked with an asterisk (*) correspond to states experiencing intruder state problems and will be excluded from statistical evaluation.

**TZVP Basis Set**		**CASPT2 with IPEA shift ε [a.u.] =**						
**Molecule**	**State**	**−0.12**	**0**	**0.08**	**0.1337**	**0.16**	**0.25**	**Experiment**
Ethene	$1\,^1B_{1u}$	8.23	8.34	8.48	8.45	8.41	8.54	7.82
Ethene	$1\,^3B_{1u}$	4.34	4.39	4.45	4.44	4.42	4.48	4.48
Butadiene	$1\,^1B_u$		6.11	6.24	6.31	6.35	6.47	5.83
Butadiene	$2\,^1A_g$	6.07	6.32	6.43	6.49	6.53	6.62	
Butadiene	$1\,^3B_u$		3.19	3.26	3.30	3.32	3.39	3.22
Butadiene	$1\,^3A_g$	4.82	4.97	5.05	5.10	5.13	5.21	4.91
Hexatriene	$1\,^1B_u$		4.96	5.09	5.18	5.22	5.35	4.93
Hexatriene	$2\,^1A_g$		5.10	5.22	5.29	5.32	5.42	5.21
Hexatriene	$1\,^3B_u$		2.52	2.61	2.66	2.68	2.74	2.61
Hexatriene	$1\,^3A_g$		4.06	4.17	4.23	4.25	4.34	4.11
Octatetraene	$2\,^1A_g$		4.33	4.45	4.52	4.55	4.64	3.59
Octatetraene	$1\,^1B_u$		4.37	4.51	4.59	4.63	4.75	4.41
Octatetraene	$2\,^1B_u$		5.34	5.53	5.61	5.65	5.79	5.88

Table S10: ... continued

**TZVP Basis Set**		**CASPT2 with IPEA shift ε [a.u.] =**						
**Molecule**	**State**	**−0.12**	**0**	**0.08**	**0.1337**	**0.16**	**0.25**	**Experiment**
Octatetraene	$3\ ^1A_g$		5.40	5.76	5.92	5.98	6.19	
Octatetraene	$4\ ^1A_g$		6.95	7.20	7.31	7.37	7.53	
Octatetraene	$3\ ^1B_u$		7.29	1.47*	7.36	7.72	8.09	
Octatetraene	$1\ ^3B_u$		2.15	2.25	2.29	2.31	2.38	2.10
Octatetraene	$1\ ^3A_g$		3.44	3.57	3.63	3.66	3.75	3.55
Cyclopropene	$1\ ^1B_1$	6.44	6.56	6.63	6.67	6.69	6.76	6.45
Cyclopropene	$1\ ^1B_2$	6.59	6.77	6.87	6.94	6.97	7.06	7.10
Cyclopropene	$1\ ^3B_2$	4.16	4.23	4.27	4.30	4.31	4.35	4.16
Cyclopropene	$1\ ^3B_1$	6.15	6.29	6.36	6.41	6.43	6.51	6.10
Cyclopentadiene	$1\ ^1B_2$		5.36	5.46	5.53	5.56	5.67	5.30
Cyclopentadiene	$2\ ^1A_1$		5.82	6.00	6.11	6.16	6.31	6.20
Cyclopentadiene	$3\ ^1A_1$		7.80	8.07	8.22	8.30	8.52	7.90
Cyclopentadiene	$1\ ^3B_2$		3.27	3.31	3.35	3.37	3.44	3.10
Cyclopentadiene	$1\ ^3A_1$		5.04	5.10	5.15	5.18	5.26	
Norbornadiene	$1\ ^1A_2$		5.26*	5.34*	5.42*	5.45*	5.56	5.24
Norbornadiene	$1\ ^1B_2$		5.90*	6.03*	6.13*	6.17*	6.33	5.96
Norbornadiene	$2\ ^1B_2$		7.05*	7.22*	7.33*	7.38*	7.54	6.68
Norbornadiene	$2\ ^1A_2$		7.30*	7.40*	7.49*	7.53*	7.67	7.50
Norbornadiene	$2\ ^1A_1$		7.63*	7.78*	7.85*	7.86*	7.97	
Norbornadiene	$1\ ^3A_2$		3.76*	3.83*	3.88*	3.90*	3.97	3.44
Norbornadiene	$1\ ^3B_2$		4.22*	4.28*	4.33*	4.36*	4.44	3.90
Benzene	$2\ ^1A'$		4.73	4.84	4.90	4.93	5.04	4.90
Benzene	$3\ ^1A'$		5.95	6.13	6.23	6.28	6.44	6.20
Benzene	$4\ ^1A'$		6.43	6.69	6.84	6.91	7.13	6.94
Benzene	$5\ ^1A'$		6.43	6.69	6.84	6.91	7.14	6.94
Benzene	$6\ ^1A'$		7.50*	7.89	7.99	8.03	8.17	7.80
Benzene	$7\ ^1A'$		7.54*	7.89	8.00	8.05	8.20	7.80
Benzene	$1\ ^3A'$		3.91	4.00	4.06	4.09	4.17	3.94
Benzene	$2\ ^3A'$		4.50	4.66	4.74	4.78	4.90	4.76
Benzene	$3\ ^3A'$		4.50	4.67	4.75	4.79	4.90	4.76
Benzene	$4\ ^3A'$		5.27	5.44	5.56	5.61	5.77	5.60
Benzene	$5\ ^3A'$		4.11*	7.07	7.18	7.23	7.37	7.49
Benzene	$6\ ^3A'$		5.70*	7.08	7.19	7.24	7.38	7.49
Naphthalene	$1\ ^1B_{3u}$		3.93	4.06	4.13	4.17	4.27	3.99
Naphthalene	$1\ ^1B_{2u}$		4.44	4.56	4.65	4.68	4.80	4.58
Naphthalene	$2\ ^1A_g$		5.36	5.57	5.67	5.72	5.87	5.51
Naphthalene	$1\ ^1B_{1g}$		5.45	5.71	5.82	5.87	6.02	5.25
Naphthalene	$2\ ^1B_{3u}$		5.43	5.69	5.84	5.77*	6.09	5.69

Table S10: ... continued

**TZVP Basis Set**		**CASPT2 with IPEA shift ε [a.u.] =**						
**Molecule**	**State**	**−0.12**	**0**	**0.08**	**0.1337**	**0.16**	**0.25**	**Experiment**
Naphthalene	$2\ ^1B_{1g}$		5.98	6.17	6.29	6.34	6.51	
Naphthalene	$2\ ^1B_{2u}$		5.92	6.06	6.17	6.22	6.36	6.07
Naphthalene	$3\ ^1A_{g}$		6.06	6.32	6.45	6.50	6.67	6.03
Naphthalene	$3\ ^1B_{2u}$		7.53	6.19	7.71	8.00	8.20	7.57
Naphthalene	$3\ ^1B_{3u}$		7.21	7.45	7.56	6.05*	7.78	
Naphthalene	$1\ ^3B_{2u}$		2.86	3.01	3.08	3.11	3.20	
Naphthalene	$1\ ^3B_{3u}$		3.88	4.06	4.14	4.17	4.29	
Naphthalene	$1\ ^3B_{1g}$		4.20	4.35	4.41	4.45	4.55	
Naphthalene	$2\ ^3B_{2u}$		4.40	4.52	4.58	4.61	4.71	
Naphthalene	$2\ ^3B_{3u}$		4.55	4.71	4.81	4.85	5.00	
Naphthalene	$1\ ^3A_{g}$		5.12	5.31	5.40	5.44	5.56	
Naphthalene	$2\ ^3B_{1g}$		5.68	5.89	6.01	6.07	6.24	
Naphthalene	$2\ ^3A_{g}$		5.85	6.09	6.20	6.25	6.42	
Naphthalene	$3\ ^3A_{g}$		6.06	6.28	6.40	6.45	6.62	
Naphthalene	$3\ ^3B_{1g}$		6.16	6.37	6.47	6.52	6.67	
Furan	$1\ ^1B_{2}$		6.12*	6.29*	6.32*	9.23*	6.57	6.06
Furan	$2\ ^1A_{1}$		6.08	6.23	6.32	6.37	6.50	
Furan	$3\ ^1A_{1}$		7.17	7.59	7.80	7.89	8.17	7.82
Furan	$1\ ^3B_{2}$		4.19	4.24	4.28	4.30	4.35	4.02
Furan	$1\ ^3A_{1}$		5.39	5.49	5.55	5.58	5.67	5.22
Pyrrole	$2\ ^1A_{1}$		5.89	6.04	6.13	6.17	6.31	
Pyrrole	$1\ ^1B_{2}$		5.95*	6.22*	6.34*	6.40*	6.40*	5.98
Pyrrole	$3\ ^1A_{1}$		7.44	7.73	7.89	7.95	8.17	7.54
Pyrrole	$1\ ^3B_{2}$		4.39	4.45	4.49	4.51	4.58	4.21
Pyrrole	$1\ ^3A_{1}$		5.25	5.38	5.45	5.48	5.59	5.10
Imidazole	$1\ ^1A''$		6.64	6.72	6.77	6.79	6.87	
Imidazole	$2\ ^1A'$		5.55*	5.80*	5.94	6.00*	6.19	6.00
Imidazole	$3\ ^1A'$		6.49*	6.65*	6.74	6.79*	6.93	6.53
Imidazole	$2\ ^1A''$		7.71	7.80	7.85	7.88	7.97	
Imidazole	$4\ ^1A'$		7.24*	7.65*	7.83	7.91*	8.16	
Imidazole	$1\ ^3A'$		4.45	4.55	4.61	4.63	4.72	
Imidazole	$2\ ^3A'$		5.44	5.57	5.65	5.69	5.80	
Imidazole	$1\ ^3A''$		6.19	6.27	6.32	6.34	6.42	
Imidazole	$3\ ^3A'$		6.03	6.22	6.32	6.37	6.51	
Imidazole	$4\ ^3A'$		7.20	7.30	7.37	7.40	7.50	
Imidazole	$2\ ^3A''$		7.30	7.40	7.46	7.49	7.58	
Pyridine	$1\ ^1B_{2}$		4.87	4.95	4.99	5.01	5.08	4.99
Pyridine	$2\ ^1A_{1}$		5.60	5.95	6.11	6.18	6.39	6.38

Table S10: … continued

TZVP Basis Set		CASPT2 with IPEA shift ε [a.u.] =						
**Molecule**	**State**	**−0.12**	**0**	**0.08**	**0.1337**	**0.16**	**0.25**	**Experiment**
Pyridine	$3\,^1A_1$		6.73	7.00	7.16	7.23	7.46	7.22
Pyridine	$2\,^1B_2$		6.91	7.08	7.17	7.21	7.33	
Pyridine	$4\,^1A_1$		8.37	8.47	8.54	8.58	8.69	
Pyridine	$3\,^1B_2$		8.46	8.52	8.57	8.59	8.66	
Pyridine	$1\,^1B_1$		4.97	5.05	5.10	5.12	5.19	4.59
Pyridine	$1\,^1A_2$		5.29	5.38	5.43	5.45	5.53	5.43
Pyridine	$1\,^3A_1$		4.03	4.13	4.19	4.22	4.30	4.10
Pyridine	$1\,^3B_2$		4.26*	4.47	4.57	4.62	4.75	4.84
Pyridine	$2\,^3A_1$		4.66	4.83	4.91	4.95	5.07	
Pyridine	$2\,^3B_2$		5.34*	5.72	5.84	5.89	6.06	
Pyridine	$3\,^3A_1$		6.96*	7.25*	7.40	7.44	7.59	
Pyridine	$3\,^3B_2$		7.62*	7.71	7.78	7.81	7.91	
Pyridine	$1\,^3B_1$		4.35	4.43	4.48	4.51	4.59	
Pyridine	$1\,^3A_2$		5.26	5.36	5.42	5.45	5.54	
Pyrazine	$1\,^1B_{2u}$		4.49	4.64	4.72	4.76	4.88	4.81
Pyrazine	$1\,^1B_{1u}$		6.51	6.65	6.74	6.78	6.92	6.51
Pyrazine	$2\,^1B_{1u}$		7.20	7.43	7.56	7.62	7.82	7.67
Pyrazine	$2\,^1B_{2u}$		7.06	7.28	7.42	7.48	7.68	7.67
Pyrazine	$1\,^1B_{3g}$		8.13	8.29	8.38	8.42	8.50	
Pyrazine	$2\,^1A_g$		8.10	8.31	8.41	8.46	8.61	
Pyrazine	$1\,^1B_{3u}$		3.83	3.94	4.01	4.04	4.14	3.83
Pyrazine	$1\,^1A_u$		4.22	4.42	4.52	4.57	4.72	
Pyrazine	$1\,^1B_{2g}$		5.40	5.51	5.58	5.60	5.70	5.46
Pyrazine	$1\,^1B_{1g}$		5.85*	6.12*	6.22*	6.26	6.43	6.10
Pyrimidine	$1\,^1B_2$		4.89	5.10	5.17	5.20	5.31	5.12
Pyrimidine	$2\,^1A_1$		6.08	6.33	6.44	6.48	6.63	6.70
Pyrimidine	$3\,^1A_1$		6.56	6.81	6.95	7.01	7.21	7.57
Pyrimidine	$2\,^1B_2$		6.21	7.30	7.44	7.51	7.71	7.57
Pyrimidine	$3\,^1B_2$		8.37	8.55	8.64	8.68	8.81	
Pyrimidine	$4\,^1A_1$		8.83	8.95	9.03	9.07	9.19	
Pyrimidine	$1\,^1B_1$		4.24	4.34	4.40	4.42	4.51	4.16
Pyrimidine	$1\,^1A_2$		4.56	4.67	4.74	4.77	4.87	4.62
Pyridazine	$2\,^1A_1$		4.84	4.96	5.04	5.07	5.18	4.95
Pyridazine	$1\,^1B_2$		5.76	6.03	6.16	6.22	6.40	6.35
Pyridazine	$2\,^1B_2$		6.63	6.93	7.09	7.16	7.38	7.20
Pyridazine	$3\,^1A_1$		6.84	7.15	7.31	7.39	7.62	7.20
Pyridazine	$1\,^1B_1$		3.56	3.67	3.74	3.77	3.87	3.30
Pyridazine	$1\,^1A_2$		3.99	4.15	4.24	4.28	4.40	

Table S10: ... continued

TZVP Basis Set		CASPT2 with IPEA shift ε [a.u.] =						
**Molecule**	**State**	**−0.12**	**0**	**0.08**	**0.1337**	**0.16**	**0.25**	**Experiment**
Pyridazine	$2\ ^1A_2$		5.47	5.62	5.70	5.74	5.86	5.30
Pyridazine	$2\ ^1B_1$		6.15	6.33	6.42	6.47	6.60	5.75
Triazine	$2\ ^1A'$		5.50	5.60	5.66	5.69	5.79	5.70
Triazine	$3\ ^1A'$		6.47	6.94	7.06	7.11	7.26	6.86
Triazine	$4\ ^1A'$		6.77	6.98	7.16	7.25	7.50	7.76
Triazine	$5\ ^1A'$		7.97	8.54	8.70	8.77	8.95	
Triazine	$1\ ^1A''$		4.18	4.41	4.52	4.58	4.74	
Triazine	$2\ ^1A''$		4.38	4.52	4.62	4.66	4.81	4.59
Triazine	$3\ ^1A''$		4.41	4.54	4.65	4.69	4.85	3.97
Triazine	$4\ ^1A''$		4.45	4.56	4.67	4.71	4.85	3.97
Triazine	$5\ ^1A''$		−12.64*	7.31	7.61	7.68	7.86	6.15
Triazine	$6\ ^1A''$		−10.36*	7.42	7.63	7.69	7.86	6.15
Tetrazine	$1\ ^1B_{2u}$		4.54	4.69	4.78	4.82	4.94	4.99
Tetrazine	$1\ ^1B_{1u}$		6.33	6.56	6.70	6.76	6.95	7.10
Tetrazine	$2\ ^1B_{1u}$		6.90	7.09	7.20	7.25	7.43	7.60
Tetrazine	$2\ ^1B_{2u}$		7.24	7.52	7.66	7.72	7.91	8.30
Tetrazine	$2\ ^1B_{3g}$		7.01	7.34	7.52	7.60	7.86	
Tetrazine	$3\ ^1A_g$		6.97	7.44	7.66	7.75	8.03	
Tetrazine	$1\ ^1B_{3u}$		1.98	2.10	2.17	2.20	2.30	2.30
Tetrazine	$1\ ^1A_u$		3.06	3.25	3.34	3.38	3.52	3.40
Tetrazine	$2\ ^1A_g$		4.44	4.52	4.57	4.59	4.66	
Tetrazine	$1\ ^1B_{1g}$		4.09	4.40	4.53	4.58	4.74	
Tetrazine	$1\ ^1B_{2g}$		4.79	4.96	5.04	5.08	5.20	
Tetrazine	$1\ ^1B_{3g}$		5.27	5.51	5.63	5.69	5.87	
Tetrazine	$2\ ^1A_u$		5.09	5.25	5.33	5.37	5.50	
Tetrazine	$2\ ^1B_{2g}$		5.50	5.72	5.84	5.89	6.07	5.50
Tetrazine	$2\ ^1B_{1g}$		5.83	6.06	6.19	6.26	6.46	5.90
Tetrazine	$2\ ^1B_{3u}$		6.23	6.44	6.56	6.61	6.78	6.47
Tetrazine	$3\ ^1B_{1g}$		6.03	6.33	6.47	6.54	6.73	
Tetrazine	$1\ ^3B_{3u}$		1.27	1.40	1.47	1.51	1.62	
Tetrazine	$1\ ^3A_u$		2.81	2.99	3.09	3.14	3.29	
Tetrazine	$1\ ^3B_{1g}$		3.74	3.90	3.98	4.02	4.14	
Tetrazine	$1\ ^3B_{1u}$		4.19	4.25	4.29	4.31	4.38	1.69
Tetrazine	$1\ ^3B_{2u}$		3.94	4.13	4.23	4.27	4.40	2.90
Tetrazine	$1\ ^3B_{2g}$		4.50	4.68	4.77	4.81	4.95	3.60
Tetrazine	$2\ ^3A_u$		4.55	4.74	4.85	4.90	5.05	
Tetrazine	$1\ ^3B_{3g}$		7.28	7.41	7.48	7.51	7.62	
Tetrazine	$2\ ^3B_{1u}$		5.04	5.20	5.27	5.31	5.41	

Table S10: ... continued

**TZVP Basis Set**		**CASPT2 with IPEA shift ε [a.u.] =**						
**Molecule**	**State**	**−0.12**	**0**	**0.08**	**0.1337**	**0.16**	**0.25**	**Experiment**
Tetrazine	$2\ ^3B_{2g}$		5.33	5.59	5.72	5.78	5.97	
Tetrazine	$2\ ^3B_{1g}$		5.76	6.00	6.13	6.19	6.38	
Tetrazine	$2\ ^3B_{3u}$		5.99	6.21	6.33	6.38	6.55	
Tetrazine	$2\ ^3B_{2u}$		5.88	6.04	6.14	6.18	6.32	
Formaldehyde	$1\ ^1A_2$	4.08	4.11	4.11	4.12	4.13	4.14	4.02
Formaldehyde	$1\ ^1B_1$	9.21	9.25	9.25	9.27	9.27	9.29	
Formaldehyde	$2\ ^1A_1$	8.67	8.93	9.07	9.15	9.19	9.32	
Formaldehyde	$1\ ^3A_2$	3.58	3.65	3.68	3.70	3.71	3.74	3.50
Formaldehyde	$1\ ^3A_1$	5.97	5.99	5.98	5.99	5.99	5.99	5.82
Acetone	$1\ ^1A_2$	4.25	4.28	4.57	4.61	4.62	4.68	4.38
Acetone	$1\ ^1B_1$	10.42	9.10	10.56	10.59	10.61	10.65	
Acetone	$2\ ^1A_1$	8.84	9.05	9.08	9.15	9.19	9.29	
Acetone	$1\ ^3A_2$	3.97	3.91	4.21	4.25	4.27	4.34	4.16
Acetone	$1\ ^3A_1$	6.05	5.91	6.18	6.21	6.23	6.28	5.88
Benzoquinone	$1\ ^1B_{1g}$		2.38	2.52	2.60	2.64	2.76	2.60
Benzoquinone	$1\ ^1A_u$		2.43	2.55	2.62	2.66	2.77	2.59
Benzoquinone	$1\ ^1B_{3g}$		3.99	3.96	4.07	4.11	4.25	4.29
Benzoquinone	$2\ ^1A_g$		4.32	4.36	4.40	4.42	4.49	
Benzoquinone	$1\ ^1B_{2g}$		4.38	5.17	5.37	5.44	5.64	
Benzoquinone	$1\ ^1B_{1u}$		4.77	4.87	4.96	5.00	5.14	5.31
Benzoquinone	$1\ ^1B_{3u}$		4.41	5.22	5.37	5.44	5.64	
Benzoquinone	$2\ ^1B_{2g}$		5.71	6.07	6.25	6.33	6.59	
Benzoquinone	$2\ ^1B_{1g}$		5.52	5.76	5.89	5.95	6.13	
Benzoquinone	$2\ ^1A_u$		4.15	5.66	4.61*	−10.33*	6.03	
Benzoquinone	$3\ ^1A_g$		5.75	5.86	5.93	5.96	6.06	
Benzoquinone	$2\ ^1B_{3g}$		6.83	6.62	6.74	6.80	6.96	
Benzoquinone	$1\ ^1B_{2u}$		7.02	7.01	7.11	7.16	7.32	
Benzoquinone	$2\ ^1B_{1u}$		7.09	6.49	6.68	6.77	7.04	7.30
Benzoquinone	$3\ ^1B_{1u}$		7.54	7.26	7.37	7.42	7.58	
Benzoquinone	$3\ ^1B_{1g}$		6.55	7.16	7.54	7.63	7.88	
Benzoquinone	$3\ ^1A_u$		6.77	7.39	7.56	7.63	7.89	
Benzoquinone	$4\ ^1A_g$		7.52	7.78	7.87	7.91	8.05	
Benzoquinone	$5\ ^1A_g$		7.67	7.78	7.89	7.94	8.11	
Benzoquinone	$1\ ^3B_{1g}$		2.88	2.36	2.45	2.49	2.62	2.30
Benzoquinone	$1\ ^3A_u$		2.28	2.42	2.50	2.54	2.66	2.34
Benzoquinone	$1\ ^3B_{1u}$		2.20	2.83	2.89	2.91	2.98	
Benzoquinone	$1\ ^3B_{3g}$		2.99	3.12	3.19	3.22	3.32	
Formamide	$1\ ^1A''$	5.66	5.70	5.72	5.73	5.74	5.76	5.50

Table S10: ... continued

**TZVP Basis Set**		**CASPT2 with IPEA shift ε [a.u.] =**						
**Molecule**	**State**	**−0.12**	**0**	**0.08**	**0.1337**	**0.16**	**0.25**	**Experiment**
Formamide	$2\,^1A'$	6.69	7.00	7.14	7.23	7.27	7.39	7.40
Formamide	$3\,^1A'$	9.91	10.18	10.31	10.39	10.42	10.54	
Formamide	$1\,^3A''$		5.42	5.46	5.48	5.49	5.52	5.30
Formamide	$1\,^3A'$	5.63	5.69	5.74	5.76	5.77	5.81	
Acetamide	$1\,^1A''$		5.73	5.75	5.77	5.78	5.81	5.44
Acetamide	$2\,^1A'$		6.87	7.01	7.10	7.14	7.27	7.40
Acetamide	$3\,^1A'$		9.67	9.82	9.91	9.95	10.09	9.90
Acetamide	$1\,^3A''$		5.38	5.44	5.47	5.49	5.54	
Acetamide	$1\,^3A'$		5.58	5.64	5.68	5.70	5.76	
Propanamide	$1\,^1A''$		5.75	5.78	5.80	5.81	5.84	5.44
Propanamide	$2\,^1A'$		6.79	6.94	7.03	7.07	7.20	7.40
Propanamide	$3\,^1A'$		9.48	9.65	9.75	9.79	9.94	9.90
Propanamide	$1\,^3A''$		5.41	5.45	5.49	5.51	5.56	
Propanamide	$1\,^3A'$		5.73	5.77	5.80	5.81	5.86	
Cytosine	$2\,^1A'$		4.35	4.47	4.54	4.57	4.67	4.60
Cytosine	$1\,^1A''$		5.04	5.16	5.23	5.26	5.37	5.15
Cytosine	$3\,^1A'$		5.03	5.23	5.33	5.38	5.53	5.60
Cytosine	$4\,^1A'$		5.70	5.99	6.13	6.20	6.40	6.23
Cytosine	$2\,^1A''$		5.46	5.58	5.65	5.68	5.78	
Cytosine	$5\,^1A'$		6.38	6.62	6.74	6.80	6.97	6.90
Cytosine	$6\,^1A'$		7.56	7.84	7.97	8.03	8.22	
Thymine	$1\,^1A''$		4.91*	5.06	5.11	5.13	5.21	5.05
Thymine	$2\,^1A'$		4.64	4.83	4.89	4.93	5.06	4.88
Thymine	$3\,^1A'$		5.63	5.92	5.94	6.00	6.15	5.95
Thymine	$2\,^1A''$		5.79*	6.47	6.53	6.55	6.64	
Thymine	$4\,^1A'$		5.97	6.25	6.29	6.35	6.53	6.20
Thymine	$3\,^1A''$		6.54*	6.80	6.93	6.97	7.11	
Thymine	$5\,^1A'$		6.80	7.08	7.18	7.24	7.43	7.05
Thymine	$6\,^1A'$		7.81	−18.04*	7.89	8.15	8.48	
Uracil	$1\,^1A''$		4.95	5.04	5.07*	5.11*	5.18	4.83
Uracil	$2\,^1A'$		4.81	4.97	5.06	5.10	5.23	5.10
Uracil	$3\,^1A'$		5.71	5.88	5.97	6.02	6.15	6.00
Uracil	$2\,^1A''$		6.19	6.36	5.93*	6.45*	6.54	
Uracil	$3\,^1A''$		6.69	7.02	6.54*	7.14*	7.25	
Uracil	$4\,^1A'$		6.18	6.41	6.52	6.58	6.74	6.60
Uracil	$4\,^1A''$		7.07	7.25	6.99*	7.39	7.55	
Uracil	$5\,^1A'$		6.80	7.05	7.18	7.24	7.42	6.95
Adenine	$2\,^1A'$		4.72	4.91	5.01	5.06	5.20	4.63

Table S10: ... continued

TZVP Basis Set		CASPT2 with IPEA shift ε [a.u.] =						
**Molecule**	**State**	**−0.12**	**0**	**0.08**	**0.1337**	**0.16**	**0.25**	**Experiment**
Adenine	$3\ ^1A'$		4.81	5.01	5.11	5.15	5.29	4.92
Adenine	$1\ ^1A''$		5.00	5.15	5.16	5.22	5.34	5.40
Adenine	$4\ ^1A'$		5.62	5.92	6.07	6.14	6.34	5.99
Adenine	$5\ ^1A'$		5.87	6.24	6.39	6.45	6.64	5.33
Adenine	$2\ ^1A''$		5.83	5.92	5.98	6.01	6.10	
Adenine	$6\ ^1A'$		6.15	6.47	6.61	6.67	6.87	6.81
Adenine	$7\ ^1A'$		6.84	7.14	7.28	7.35	7.56	7.75

Let us now comment on a few states for which we had identified intruder state problems. In some cases, the intruder states were straightforward, since the states possessed low reference weights and appeared at suspiciously low energies. This was the case, e.g., of the $3\ 1B_u$ state of octatetraene or the $6\ ^1A'$ state of thymine for $\varepsilon = 0.08$ a.u. In other states, this assignment was not that clear, for example if they were only coupled to a state clearly experiencing intruder state problems in the multi-state CASPT2 treatment. To what extent this coupling corrupted the energies of these states cannot be easily estimated. We have already discussed in section S3.4.1 that the IPEA shift should not be employed as a substitute for the level shift for removing the coupling to intruder states. However, its effect can be the same: variation of the IPEA shift value changes the energy denominators in the first-order wave function and second-order energy contributions and can, thus, decrease the coupling to intruder states. When this coupling is sufficiently damped, a further increase of the IPEA shift should further increase the energy of the state with the size of the increase related to the open-shell character of the state. This is naturally also the behavior for states that do not experience any intruder state problems. Keeping this in mind, we can find a number of states that we have ascribed intruder state problems to (see sections S3.4.2 and S3.4.3) whose excitation energies converge with increasing IPEA shift value to the excitation energy with $\varepsilon = 0.25$ a.u. where there is no intruder state problem present, e.g., the $^1A'$ states of imidazole or the $1\ ^1B_{1g}$ state of pyrazine. These states did not suffer from intruder state problems at the SS-CASPT2 level but rather were mixed with affected states at the MS-CASTP2 level. We chose to exclude them from our data set with the concern that these energies might be corrupted by the coupling to the affected states. However, the normally appearing trend of increasing energy with increasing IPEA shift value

–without any spike as, e.g., seen for the 1 $^1B_2$ state of furan –might raise the question if we had been overly cautious in excluding these states from our statistical analysis. Unfortunately, we see no way of clarifying this question and, thus, decide to rather stay on the safe side.

In Table S11 we show the NOS and the approximate dynamical correlation energy $\widetilde{E}^{\text{dyn}}$ for the states in the Thiel benchmark set. We only show the NOS value computed using the IPEA shift value of $\varepsilon = 0.25$ a.u. The NOS values obtained using the other shift values are practically identical and, thus, not reported. The approximate dynamical correlation energy is given by the difference $\widetilde{E}^{\text{dyn}} = E_{\text{CASSCF}} - E_{\text{CASPT2}}$.

Table S11: Number of open shells (NOS) and approximate dynamical correlation energy $\widetilde{E}^{\text{dyn}}$ in eV for the electronic states in Thiel's benchmark set.

**TZVP Basis Set**			**CASPT2 with IPEA shift ε [a.u.] =**					
**Molecule**	**State**	**NOS**	**−0.12**	**0**	**0.08**	**0.1337**	**0.16**	**0.25**
Ethene	1 ^1A_g	0.00	7.25	7.21	7.17	7.17	7.19	7.14
Ethene	1 $^1B_{1u}$	2.00	8.46	8.31	8.13	8.16	8.22	8.04
Ethene	1 $^3B_{1u}$	2.00	7.19	7.10	7.00	7.01	7.05	6.94
Butadiene	1 ^1A_g	0.21	14.47	14.45	14.42	14.41	14.40	14.38
Butadiene	1 ^1B_u	1.84		16.37	16.21	16.12	16.07	15.93
Butadiene	2 ^1A_g	1.37	14.85	14.58	14.44	14.36	14.32	14.20
Butadiene	1 ^3B_u	2.00		14.17	14.07	14.01	13.99	13.90
Butadiene	1 ^3A_g	2.00	14.32	14.15	14.04	13.97	13.94	13.83
Hexatriene	1 ^1A_g	0.37		20.92	20.87	20.85	20.84	20.81
Hexatriene	1 ^1B_u	2.01		23.33	23.14	23.03	22.99	22.83
Hexatriene	2 ^1A_g	1.38		21.33	21.17	21.08	21.04	20.91
Hexatriene	1 ^3B_u	2.09		21.05	20.91	20.85	20.82	20.72
Hexatriene	1 ^3A_g	2.05		21.06	20.90	20.82	20.78	20.66
Octatetraene	1 ^1A_g	0.50		27.68	27.61	27.59	27.57	27.53
Octatetraene	2 ^1A_g	1.46		28.03	27.85	27.76	27.71	27.57
Octatetraene	1 ^1B_u	2.04		30.03	29.82	29.71	29.66	29.50
Octatetraene	2 ^1B_u	2.19		28.23	27.98	27.87	27.81	27.63
Octatetraene	3 ^1A_g	2.23		28.86	28.43	28.25	28.17	27.91
Octatetraene	4 ^1A_g	1.87		28.22	27.91	27.77	27.70	27.49
Octatetraene	3 ^1B_u	2.43		28.62		28.46	28.09	27.68
Octatetraene	1 ^3B_u	2.09		27.77	27.60	27.53	27.49	27.38
Octatetraene	1 ^3A_g	2.05		27.79	27.60	27.51	27.47	27.33
Cyclopropene	1 $^1A_1$	0.00	10.81	10.77	10.74	10.73	10.72	10.69
Cyclopropene	1 $^1B_1$	2.00	11.66	11.51	11.41	11.35	11.32	11.23
Cyclopropene	1 $^1B_2$	2.00	12.84	12.62	12.49	12.41	12.37	12.25
Cyclopropene	1 $^3B_2$	2.00	10.86	10.74	10.67	10.63	10.61	10.55

Table S11: . . . continued

TZVP Basis Set			CASPT2 with IPEA shift ε [a.u.] =					
**Molecule**	**State**	**NOS**	**−0.12**	**0**	**0.08**	**0.1337**	**0.16**	**0.25**
Cyclopropene	$1\,^3B_1$	2.00	11.50	11.32	11.22	11.15	11.12	11.02
Cyclopentadiene	$1\,^1A_1$	0.11		18.24	18.18	18.16	18.14	18.11
Cyclopentadiene	$1\,^1B_2$	2.00		20.06	19.90	19.81	19.76	19.62
Cyclopentadiene	$2\,^1A_1$	1.51		18.89	18.65	18.52	18.46	18.28
Cyclopentadiene	$3\,^1A_1$	1.92		21.17	20.85	20.67	20.58	20.32
Cyclopentadiene	$1\,^3B_2$	2.00		17.78	17.67	17.60	17.57	17.47
Cyclopentadiene	$1\,^3A_1$	2.00		17.77	17.64	17.57	17.53	17.42
Norbornadiene	$1\,^1A_1$	0.09		26.23	26.14	26.11	26.09	26.04
Norbornadiene	$1\,^1A_2$	2.00		28.20	28.03	27.92	27.87	27.70
Norbornadiene	$1\,^1B_2$	2.00		29.07	28.85	28.72	28.66	28.45
Norbornadiene	$2\,^1B_2$	2.00		29.01	28.75	28.60	28.53	28.32
Norbornadiene	$2\,^1A_2$	2.00		28.74	28.54	28.42	28.36	28.18
Norbornadiene	$2\,^1A_1$	0.65		26.14	25.89	25.79	25.76	25.60
Norbornadiene	$1\,^3A_2$	2.00		25.88	25.73	25.65	25.61	25.48
Norbornadiene	$1\,^3B_2$	2.00		25.80	25.66	25.57	25.53	25.39
Benzene	$1\,^1A'$	0.22		20.60	20.55	20.53	20.52	20.49
Benzene	$2\,^1A'$	2.17		20.68	20.53	20.44	20.40	20.26
Benzene	$3\,^1A'$	2.04		22.56	22.34	22.22	22.16	21.97
Benzene	$4\,^1A'$	2.04		23.44	23.13	22.96	22.88	22.63
Benzene	$5\,^1A'$	2.07		23.44	23.13	22.96	22.88	22.62
Benzene	$6\,^1A'$	2.14		21.11	20.67	20.56	20.50	20.33
Benzene	$7\,^1A'$	1.71		21.07	20.67	20.54	20.49	20.31
Benzene	$1\,^3A'$	2.08		20.37	20.23	20.15	20.12	20.00
Benzene	$2\,^3A'$	2.25		20.90	20.69	20.59	20.54	20.40
Benzene	$3\,^3A'$	2.21		20.90	20.69	20.58	20.54	20.39
Benzene	$4\,^3A'$	2.00		22.61	22.38	22.25	22.19	22.00
Naphthalene	$1\,^1A_g$	0.38		33.53	33.44	33.41	33.39	33.33
Naphthalene	$1\,^1B_{3u}$	2.27		33.76	33.55	33.44	33.39	33.23
Naphthalene	$1\,^1B_{2u}$	2.11		35.58	35.37	35.25	35.20	35.02
Naphthalene	$2\,^1A_g$	2.11		33.94	33.65	33.51	33.44	33.23
Naphthalene	$1\,^1B_{1g}$	2.16		34.49	34.15	34.00	33.94	33.73
Naphthalene	$2\,^1B_{3u}$	2.14		36.21	35.86	35.68	35.73	35.35
Naphthalene	$2\,^1B_{1g}$	2.12		35.98	35.70	35.55	35.48	35.26
Naphthalene	$2\,^1B_{2u}$	2.08		35.68	35.46	35.31	35.25	35.05
Naphthalene	$3\,^1A_g$	2.27		34.25	33.90	33.74	33.67	33.44
Naphthalene	$3\,^1B_{2u}$	2.32		36.26	37.52	35.97	35.65	35.39
Naphthalene	$3\,^1B_{3u}$	2.13		34.11	33.79	33.64	35.14	33.35
Naphthalene	$1\,^3B_{2u}$	2.11		33.62	33.38	33.28	33.23	33.09

Table S11: . . . continued

**TZVP Basis Set**			**CASPT2 with IPEA shift ε [a.u.] =**					
**Molecule**	**State**	**NOS**	**−0.12**	**0**	**0.08**	**0.1337**	**0.16**	**0.25**
Naphthalene	$1\,^3B_{3u}$	2.19		33.90	33.64	33.52	33.47	33.30
Naphthalene	$1\,^3B_{1g}$	2.08		33.70	33.48	33.37	33.33	33.17
Naphthalene	$2\,^3B_{2u}$	2.29		33.68	33.48	33.38	33.33	33.18
Naphthalene	$2\,^3B_{3u}$	2.04		35.30	35.06	34.92	34.86	34.65
Naphthalene	$1\,^3A_g$	2.14		33.94	33.67	33.54	33.49	33.30
Naphthalene	$2\,^3B_{1g}$	2.13		35.85	35.55	35.39	35.32	35.09
Naphthalene	$2\,^3A_g$	2.65		34.02	33.71	33.55	33.48	33.26
Naphthalene	$3\,^3A_g$	2.20		35.57	35.27	35.12	35.04	34.82
Naphthalene	$3\,^3B_{1g}$	2.23		33.95	33.65	33.51	33.45	33.24
Furan	$1\,^1A_1$	0.10		19.45	19.39	19.37	19.36	19.33
Furan	$1\,^1B_2$	2.02		21.40	21.18	21.12	18.21	20.83
Furan	$2\,^1A_1$	2.10		19.97	19.76	19.65	19.59	19.42
Furan	$3\,^1A_1$	1.93		22.39	21.91	21.68	21.58	21.27
Furan	$1\,^3B_2$	2.00		18.89	18.78	18.72	18.70	18.60
Furan	$1\,^3A_1$	2.01		19.19	19.04	18.96	18.92	18.79
Pyrrole	$1\,^1A_1$	0.10		18.84	18.80	18.78	18.77	18.74
Pyrrole	$2\,^1A_1$	2.14		19.40	19.20	19.09	19.04	18.88
Pyrrole	$1\,^1B_2$	2.04		20.73	20.42	20.28	20.21	20.18
Pyrrole	$3\,^1A_1$	1.54		20.92	20.58	20.41	20.33	20.09
Pyrrole	$1\,^3B_2$	2.00		18.49	18.39	18.32	18.30	18.20
Pyrrole	$1\,^3A_1$	2.01		18.95	18.78	18.69	18.65	18.51
Imidazole	$1\,^1A'$	0.07		19.55	19.51	19.50	19.49	19.47
Imidazole	$1\,^1A''$	2.04		19.63	19.51	19.45	19.42	19.32
Imidazole	$2\,^1A'$	2.06		20.85	20.56	20.40	20.34	20.13
Imidazole	$3\,^1A'$	2.00		21.11	20.92	20.81	20.75	20.59
Imidazole	$2\,^1A''$	2.08		19.94	19.82	19.74	19.71	19.60
Imidazole	$4\,^1A'$	1.88		22.06	21.61	21.42	21.33	21.06
Imidazole	$1\,^3A'$	2.01		19.59	19.45	19.38	19.35	19.24
Imidazole	$2\,^3A'$	2.16		19.76	19.59	19.50	19.45	19.32
Imidazole	$1\,^3A''$	2.01		19.57	19.45	19.39	19.35	19.25
Imidazole	$3\,^3A'$	2.03		20.50	20.27	20.16	20.11	19.95
Imidazole	$4\,^3A'$	2.04		20.10	19.96	19.88	19.84	19.71
Imidazole	$2\,^3A''$	2.14		19.92	19.78	19.70	19.67	19.55
Pyridine	$1\,^1A_1$	0.22		21.27	21.22	21.20	21.19	21.15
Pyridine	$1\,^1B_2$	2.17		20.71	20.58	20.51	20.48	20.38
Pyridine	$2\,^1A_1$	2.05		23.61	23.21	23.03	22.95	22.71
Pyridine	$3\,^1A_1$	2.10		22.94	22.62	22.44	22.36	22.10
Pyridine	$2\,^1B_2$	2.05		22.65	22.43	22.32	22.27	22.11

Table S11: ... continued

TZVP Basis Set			CASPT2 with IPEA shift ε [a.u.] =					
**Molecule**	**State**	**NOS**	**−0.12**	**0**	**0.08**	**0.1337**	**0.16**	**0.25**
Pyridine	$4\ ^1A_1$	1.63		22.48	22.34	22.24	22.20	22.05
Pyridine	$3\ ^1B_2$	2.13		20.29	20.18	20.11	20.08	19.98
Pyridine	$1\ ^1B_1$	2.17		21.25	21.12	21.05	21.02	20.92
Pyridine	$1\ ^1A_2$	2.09		21.68	21.54	21.46	21.43	21.32
Pyridine	$1\ ^3A_1$	2.12		20.97	20.81	20.73	20.69	20.57
Pyridine	$1\ ^3B_2$	2.14		21.86	21.60	21.48	21.42	21.26
Pyridine	$2\ ^3A_1$	2.23		21.51	21.29	21.19	21.14	20.99
Pyridine	$2\ ^3B_2$	2.08		23.59	23.17	23.02	22.96	22.75
Pyridine	$3\ ^3A_1$	2.60		21.37	21.04	20.87	20.81	20.63
Pyridine	$3\ ^3B_2$	2.13		20.75	20.61	20.52	20.48	20.34
Pyridine	$1\ ^3B_1$	2.12		21.29	21.16	21.08	21.05	20.94
Pyridine	$1\ ^3A_2$	2.08		21.70	21.54	21.46	21.42	21.30
Pyrazine	$1\ ^1A_g$	0.27		21.70	21.64	21.61	21.60	21.56
Pyrazine	$1\ ^1B_{2u}$	2.20		22.18	21.97	21.86	21.81	21.65
Pyrazine	$1\ ^1B_{1u}$	2.08		23.69	23.49	23.37	23.32	23.14
Pyrazine	$2\ ^1B_{1u}$	2.05		24.83	24.54	24.38	24.31	24.07
Pyrazine	$2\ ^1B_{2u}$	2.12		24.57	24.28	24.12	24.05	23.81
Pyrazine	$1\ ^1B_{3g}$	2.13		21.81	21.58	21.47	21.41	21.30
Pyrazine	$2\ ^1A_g$	1.65		21.92	21.65	21.52	21.46	21.27
Pyrazine	$1\ ^1B_{3u}$	2.13		22.61	22.43	22.34	22.30	22.16
Pyrazine	$1\ ^1A_u$	2.20		23.63	23.37	23.24	23.18	22.99
Pyrazine	$1\ ^1B_{2g}$	2.16		22.00	21.83	21.74	21.70	21.57
Pyrazine	$1\ ^1B_{1g}$	2.31		23.19	22.86	22.74	22.68	22.47
Pyrimidine	$1\ ^1A_1$	0.21		21.89	21.83	21.81	21.80	21.76
Pyrimidine	$1\ ^1B_2$	2.17		22.09	21.82	21.72	21.68	21.53
Pyrimidine	$2\ ^1A_1$	2.08		23.90	23.59	23.46	23.40	23.22
Pyrimidine	$3\ ^1A_1$	2.04		25.17	24.87	24.70	24.63	24.39
Pyrimidine	$2\ ^1B_2$	2.07		25.69	24.55	24.39	24.31	24.07
Pyrimidine	$3\ ^1B_2$	2.15		22.01	21.77	21.66	21.61	21.45
Pyrimidine	$4\ ^1A_1$	1.71		21.71	21.53	21.42	21.37	21.22
Pyrimidine	$1\ ^1B_1$	2.19		22.34	22.18	22.10	22.06	21.93
Pyrimidine	$1\ ^1A_2$	2.21		22.68	22.51	22.42	22.38	22.24
Pyridazine	$1\ ^1A_1$	0.24		21.85	21.79	21.76	21.75	21.71
Pyridazine	$2\ ^1A_1$	2.21		21.96	21.78	21.68	21.63	21.49
Pyridazine	$1\ ^1B_2$	2.04		23.97	23.63	23.47	23.40	23.18
Pyridazine	$2\ ^1B_2$	2.07		24.79	24.43	24.25	24.16	23.90
Pyridazine	$3\ ^1A_1$	1.90		25.00	24.64	24.45	24.36	24.09
Pyridazine	$1\ ^1B_1$	2.13		22.76	22.58	22.49	22.44	22.31

Table S11: ... continued

TZVP Basis Set			CASPT2 with IPEA shift ε [a.u.] =					
**Molecule**	**State**	**NOS**	**−0.12**	**0**	**0.08**	**0.1337**	**0.16**	**0.25**
Pyridazine	$1\ ^1A_2$	2.26		22.83	22.60	22.49	22.44	22.28
Pyridazine	$2\ ^1A_2$	2.12		22.59	22.38	22.27	22.22	22.06
Pyridazine	$2\ ^1B_1$	2.32		22.81	22.58	22.45	22.40	22.22
Triazine	$1\ ^1A'$	0.21		22.66	22.59	22.56	22.55	22.51
Triazine	$2\ ^1A'$	2.17		22.45	22.28	22.19	22.15	22.01
Triazine	$3\ ^1A'$	2.11		24.99	24.45	24.31	24.25	24.05
Triazine	$4\ ^1A'$	2.02		26.37	26.10	25.88	25.79	25.49
Triazine	$5\ ^1A'$	2.20		23.33	22.69	22.50	22.42	22.20
Triazine	$1\ ^1A''$	2.23		24.32	24.03	23.88	23.82	23.61
Triazine	$2\ ^1A''$	2.27		23.32	23.11	22.99	22.93	22.74
Triazine	$3\ ^1A''$	2.21		23.68	23.49	23.36	23.30	23.10
Triazine	$4\ ^1A''$	2.21		23.65	23.47	23.34	23.28	23.09
Tetrazine	$1\ ^1A_g$	0.32		23.13	23.06	23.02	23.01	22.96
Tetrazine	$1\ ^1B_{2u}$	2.18		23.41	23.19	23.07	23.02	22.85
Tetrazine	$1\ ^1B_{1u}$	2.05		26.87	26.55	26.39	26.31	26.07
Tetrazine	$2\ ^1B_{1u}$	2.11		25.44	25.17	25.03	24.96	24.74
Tetrazine	$2\ ^1B_{2u}$	2.12		24.41	24.06	23.89	23.81	23.57
Tetrazine	$2\ ^1B_{3g}$	2.17		25.50	25.10	24.89	24.79	24.49
Tetrazine	$3\ ^1A_g$	1.16		25.38	24.84	24.58	24.48	24.15
Tetrazine	$1\ ^1B_{3u}$	2.22		24.30	24.10	24.00	23.96	23.81
Tetrazine	$1\ ^1A_u$	2.31		24.63	24.37	24.24	24.19	24.00
Tetrazine	$2\ ^1A_g$	0.64		24.34	24.19	24.11	24.07	23.96
Tetrazine	$1\ ^1B_{1g}$	2.23		24.33	23.94	23.78	23.71	23.50
Tetrazine	$1\ ^1B_{2g}$	2.29		23.47	23.23	23.11	23.06	22.89
Tetrazine	$1\ ^1B_{3g}$	2.28		24.57	24.25	24.10	24.02	23.79
Tetrazine	$2\ ^1A_u$	2.33		24.14	23.91	23.79	23.73	23.56
Tetrazine	$2\ ^1B_{2g}$	2.79		24.30	24.01	23.86	23.78	23.56
Tetrazine	$2\ ^1B_{1g}$	2.32		24.27	23.96	23.79	23.72	23.47
Tetrazine	$2\ ^1B_{3u}$	2.55		24.47	24.18	24.03	23.96	23.74
Tetrazine	$3\ ^1B_{1g}$	2.51		24.52	24.15	23.97	23.89	23.65
Tetrazine	$1\ ^3B_{3u}$	2.20		24.36	24.16	24.05	24.00	23.85
Tetrazine	$1\ ^3A_u$	2.26		24.51	24.25	24.11	24.05	23.85
Tetrazine	$1\ ^3B_{1g}$	2.22		23.85	23.62	23.50	23.45	23.28
Tetrazine	$1\ ^3B_{1u}$	2.14		22.41	22.27	22.19	22.16	22.05
Tetrazine	$1\ ^3B_{2u}$	2.13		23.78	23.51	23.38	23.33	23.15
Tetrazine	$1\ ^3B_{2g}$	2.25		23.45	23.19	23.07	23.01	22.83
Tetrazine	$2\ ^3A_u$	2.28		24.40	24.14	24.00	23.94	23.74
Tetrazine	$1\ ^3B_{3g}$	2.15		22.57	22.37	22.26	22.22	22.06

Table S11: ... continued

**TZVP Basis Set**			**CASPT2 with IPEA shift ε [a.u.] =**					
**Molecule**	**State**	**NOS**	**−0.12**	**0**	**0.08**	**0.1337**	**0.16**	**0.25**
Tetrazine	$2\ ^3B_{1u}$	2.31		23.15	22.92	22.81	22.76	22.61
Tetrazine	$2\ ^3B_{2g}$	2.75		24.31	23.97	23.80	23.73	23.49
Tetrazine	$2\ ^3B_{1g}$	2.46		24.12	23.81	23.65	23.57	23.33
Tetrazine	$2\ ^3B_{3u}$	2.50		24.35	24.05	23.90	23.83	23.61
Tetrazine	$2\ ^3B_{2u}$	2.51		24.30	24.06	23.93	23.87	23.69
Formaldehyde	$1\ ^1A_1$	0.48	8.97	8.91	8.86	8.83	8.82	8.78
Formaldehyde	$1\ ^1A_2$	2.00	8.36	8.27	8.21	8.18	8.16	8.10
Formaldehyde	$1\ ^1B_1$	2.00	8.45	8.35	8.30	8.26	8.24	8.18
Formaldehyde	$2\ ^1A_1$	1.38	12.68	12.37	12.19	12.07	12.02	11.85
Formaldehyde	$1\ ^3A_2$	2.00	8.50	8.37	8.29	8.25	8.22	8.15
Formaldehyde	$1\ ^3A_1$	2.00	7.77	7.70	7.65	7.62	7.60	7.56
Acetone	$1\ ^1A_1$	0.10	16.99	16.75	16.96	16.96	16.95	16.94
Acetone	$1\ ^1A_2$	2.01		15.78	15.71	15.66	15.64	15.57
Acetone	$1\ ^1B_1$	2.00	16.23	17.31	16.05	16.02	16.00	15.94
Acetone	$2\ ^1A_1$	1.80	19.51	19.07	19.24	19.17	19.13	19.01
Acetone	$1\ ^3A_2$	2.01	16.01	15.83	15.74	15.69	15.66	15.58
Acetone	$1\ ^3A_1$	2.00	15.96	15.86	15.81	15.77	15.75	15.68
Benzoquinone	$1\ ^1A_g$	0.47		29.08	28.99	28.96	28.94	28.89
Benzoquinone	$1\ ^1B_{1g}$	2.20		29.77	29.53	29.41	29.36	29.20
Benzoquinone	$1\ ^1A_u$	2.17		29.71	29.49	29.38	29.33	29.17
Benzoquinone	$1\ ^1B_{3g}$	2.15		30.74	30.67	30.53	30.47	30.29
Benzoquinone	$2\ ^1A_g$	0.28		29.41	29.28	29.21	29.17	29.06
Benzoquinone	$1\ ^1B_{2g}$	2.82		30.73	29.85	29.62	29.53	29.28
Benzoquinone	$1\ ^1B_{1u}$	2.05		31.85	31.66	31.53	31.47	31.29
Benzoquinone	$1\ ^1B_{3u}$	2.81		30.71	29.80	29.62	29.54	29.29
Benzoquinone	$2\ ^1B_{2g}$	2.36		32.06	31.61	31.39	31.29	30.99
Benzoquinone	$2\ ^1B_{1g}$	2.51		29.76	29.43	29.26	29.19	28.96
Benzoquinone	$2\ ^1A_u$	2.51		31.13	29.52	30.54	45.47	29.05
Benzoquinone	$3\ ^1A_g$	1.34		29.23	29.03	28.92	28.88	28.73
Benzoquinone	$2\ ^1B_{3g}$	2.32		30.38	30.49	30.34	30.27	30.06
Benzoquinone	$1\ ^1B_{2u}$	2.32		29.29	29.22	29.08	29.01	28.81
Benzoquinone	$2\ ^1B_{1u}$	2.05		33.25	33.75	33.53	33.43	33.10
Benzoquinone	$3\ ^1B_{1u}$	2.36		29.43	29.62	29.47	29.41	29.21
Benzoquinone	$3\ ^1B_{1g}$	3.12		32.33	31.63	31.21	31.11	30.82
Benzoquinone	$3\ ^1A_u$	3.12		30.39	29.67	29.46		29.08
Benzoquinone	$4\ ^1A_g$	4.05		29.60	29.24	29.12	29.07	28.88
Benzoquinone	$5\ ^1A_g$	1.93		29.69	29.48	29.33	29.27	29.06
Benzoquinone	$1\ ^3B_{1g}$	2.14		29.06	29.49	29.36	29.31	29.13

Table S11: . . . continued

**TZVP Basis Set**			**CASPT2 with IPEA shift ε [a.u.] =**					
**Molecule**	**State**	**NOS**	**−0.12**	**0**	**0.08**	**0.1337**	**0.16**	**0.25**
Benzoquinone	$1\ ^3A_u$	2.12		29.66	29.43	29.31	29.26	29.10
Benzoquinone	$1\ ^3B_{1u}$	2.06		29.61	28.88	28.79	28.75	28.63
Benzoquinone	$1\ ^3B_{3g}$	2.10		29.56	29.33	29.23	29.18	29.04
Formamide	$1\ ^1A'$	0.06	13.90	13.85	13.81	13.79	13.78	13.74
Formamide	$1\ ^1A''$	2.00	13.13	13.04	12.98	12.94	12.93	12.87
Formamide	$2\ ^1A'$	1.98	15.97	15.61	15.43	15.32	15.27	15.11
Formamide	$3\ ^1A'$	1.38	15.80	15.47	15.31	15.21	15.16	15.00
Formamide	$1\ ^3A''$	2.00		13.07	13.00	12.95	12.93	12.86
Formamide	$1\ ^3A'$	2.00	13.50	13.38	13.30	13.26	13.23	13.16
Acetamide	$1\ ^1A'$	0.08		17.93	17.90	17.88	17.87	17.83
Acetamide	$1\ ^1A''$	2.00		17.13	17.07	17.03	17.01	16.95
Acetamide	$2\ ^1A'$	1.97		19.78	19.60	19.49	19.44	19.28
Acetamide	$3\ ^1A'$	1.53		19.97	19.79	19.67	19.62	19.45
Acetamide	$1\ ^3A''$	2.00		17.66	17.57	17.52	17.49	17.41
Acetamide	$1\ ^3A'$	2.00		18.07	17.97	17.91	17.88	17.79
Propanamide	$1\ ^1A'$	0.08		22.04	22.01	21.99	21.98	21.95
Propanamide	$1\ ^1A''$	2.00		21.23	21.17	21.13	21.11	21.04
Propanamide	$2\ ^1A'$	1.98		23.94	23.76	23.65	23.60	23.44
Propanamide	$3\ ^1A'$	1.56		24.20	24.00	23.88	23.83	23.64
Propanamide	$1\ ^3A''$	2.00		21.76	21.69	21.62	21.60	21.51
Propanamide	$1\ ^3A'$	2.00		21.79	21.71	21.66	21.63	21.55
Cytosine	$1\ ^1A'$	0.09		32.59	32.53	32.50	32.49	32.46
Cytosine	$2\ ^1A'$	2.09		33.30	33.12	33.03	32.99	32.85
Cytosine	$1\ ^1A''$	2.25		32.89	32.71	32.62	32.58	32.44
Cytosine	$3\ ^1A'$	2.01		33.87	33.61	33.48	33.42	33.24
Cytosine	$4\ ^1A'$	2.15		34.75	34.41	34.24	34.16	33.92
Cytosine	$2\ ^1A''$	2.27		32.19	32.01	31.92	31.88	31.74
Cytosine	$5\ ^1A'$	1.90		34.21	33.91	33.76	33.70	33.49
Cytosine	$6\ ^1A'$	2.12		33.89	33.56	33.40	33.33	33.10
Thymine	$1\ ^1A'$	0.09		37.04	36.99	36.97	36.96	36.93
Thymine	$1\ ^1A''$	2.17		36.99	36.79	36.72	36.69	36.58
Thymine	$2\ ^1A'$	2.03		38.82	38.58	38.50	38.45	38.29
Thymine	$3\ ^1A'$	2.10		38.58	38.24	38.19	38.13	37.94
Thymine	$2\ ^1A''$	2.18		37.63	36.90	36.82	36.78	36.67
Thymine	$4\ ^1A'$	1.92		39.14	38.81	38.75	38.68	38.47
Thymine	$3\ ^1A''$	2.23		38.35	38.04	37.89	37.84	37.67
Thymine	$5\ ^1A'$	2.10		39.25	38.91	38.80	38.72	38.50
Thymine	$6\ ^1A'$	2.05		38.52		38.36	38.10	37.74

Table S11: . . . continued

TZVP Basis Set			CASPT2 with IPEA shift ε [a.u.] =					
**Molecule**	**State**	**NOS**	**−0.12**	**0**	**0.08**	**0.1337**	**0.16**	**0.25**
Uracil	$1\ ^1A'$	0.07		32.94	32.89	32.87	32.86	32.84
Uracil	$1\ ^1A''$	2.17		32.77	32.64	32.58	32.54	32.44
Uracil	$2\ ^1A'$	2.04		34.50	34.29	34.19	34.14	33.98
Uracil	$3\ ^1A'$	2.04		34.33	34.11	34.00	33.95	33.78
Uracil	$2\ ^1A''$	2.18		33.03	32.82	33.23	32.70	32.58
Uracil	$3\ ^1A''$	2.21		34.09	33.71	34.17	33.57	33.43
Uracil	$4\ ^1A'$	1.98		34.84	34.57	34.43	34.37	34.18
Uracil	$4\ ^1A''$	2.16		33.65	33.42	33.67	33.25	33.07
Uracil	$5\ ^1A'$	2.08		34.98	34.68	34.53	34.47	34.26
Adenine	$1\ ^1A'$	0.14		39.05	38.97	38.94	38.92	38.87
Adenine	$2\ ^1A'$	2.07		39.58	39.31	39.18	39.12	38.93
Adenine	$3\ ^1A'$	2.06		40.72	40.44	40.31	40.25	40.07
Adenine	$1\ ^1A''$	2.12		39.83	39.60	39.56	39.49	39.32
Adenine	$4\ ^1A'$	2.02		41.44	41.06	40.87	40.79	40.54
Adenine	$5\ ^1A'$	1.87		40.23	39.79	39.60	39.53	39.28
Adenine	$2\ ^1A''$	2.13		39.54	39.37	39.28	39.23	39.09
Adenine	$6\ ^1A'$	2.11		40.48	40.08	39.91	39.83	39.58
Adenine	$7\ ^1A'$	2.17		40.54	40.17	39.99	39.91	39.65

S3.6 Basis Set Effects

Table S12 collects the contraction scheme of different basis sets used in this study and discussed in section 5.4.1 of the main paper. In Table S23 we list the MSEE of the CASPT2 excitation energies compared to experiment for the Thiel benchmark set (see also Figure 9 in the main paper) for different ANO-RCC basis sets (MB, VDZ, VDZP, VTZP, VQZP) and IPEA shift values ($\varepsilon = 0$, 0.10, 0.20, 0.25, 0.30, 0.40, and 0.50 a.u.). In Tables S13-S22 we show the individual CASPT2 vertical excitation energies V^{calc} and approximate dynamical correlation energies $\widetilde{E}^{\text{dyn}}$. States that experienced intruder state problems are not reported, see section S3.4.

Table S12: Size of contracted basis sets for hydrogen and second-row atoms.

Basis Set	Second-Row Atoms	Hydrogen
ANO-RCC-MB	$2s1p$	$1s$
ANO-RCC-VDZ	$3s2p$	$2s$
ANO-RCC-VDZP	$3s2p1d$	$2s1p$

Table S12: ... continued

Basis Set	Second-Row Atoms	Hydrogen
ANO-RCC-VTZP	$4s3p2d1f$	$3s2p1d$
ANO-RCC-VQZP	$5s4p3d2f1g$	$4s3p2d1f$
ANO-RCC-IPEA[a]	$8s7p4d3f2g$	$6s4p3d1f$
ANO-L-BENCHMARK[b]	$5s4p3d2s$	$3s2p1d$
ANO-L-LITERATURE[c,d]	$4s3p1d$	$2s(1p)$
TZVP	$5s3p1d$	$3s1p$

[a] basis set used by Roos and co-workers in the determination of the optimal IPEA shift value[54]

[b] basis set used by Andersson and Roos in benchmark on atomization energies of small molecules[55]

[c] most commonly used basis set in the studies included in the literature survey in section 3

[d] for Rydberg states typically an additional set of ($8s8p8d/1s1p1d$) diffuse functions was employed

Table S13: CASPT2 vertical excitation energies for the Thiel benchmark set obtained using the ANO-RCC-MB basis set and different IPEA shift parameters (ε = 0, 0.10, 0.20, 0.25, 0.30, 0.40, and 0.50 a.u.).

MB Basis Set		Excitation Energy V^{calc} [eV]						
**Molecule**	**State**	**0**	**0.10**	**0.20**	**0.25**	**0.30**	**0.40**	**0.50**
Ethene	$1\,^1B_{1u}$	10.93	10.99	11.05	11.08	11.1	11.15	11.2
Ethene	$1\,^3B_{1u}$	4.97	4.97	4.98	4.98	4.98	4.98	4.97
Butadiene	$1\,^1B_u$	8.49	8.59	8.68	8.72	8.76	8.83	8.89
Butadiene	$1\,^3B_u$	3.64	3.66	3.69	3.71	3.72	3.74	3.76
Butadiene	$1\,^3A_g$	5.64	5.66	5.69	5.70	5.72	5.74	5.76
Hexatriene	$1\,^1B_u$	7.19	7.28	7.34	7.37	7.39	7.44	7.48
Hexatriene	$2\,^1A_g$	6.01	6.06	6.10	6.12	6.14	6.17	6.2
Hexatriene	$1\,^3B_u$	2.92	2.97	3.00	3.01	3.02	3.04	3.06
Hexatriene	$1\,^3A_g$	4.65	4.70	4.73	4.75	4.76	4.79	4.81
Octatetraene	$2\,^1A_g$	5.08	5.15	5.19	5.21	5.23	5.26	5.29
Octatetraene	$1\,^1B_u$	6.33	6.41	6.47	6.49	6.51	6.56	6.6
Octatetraene	$2\,^1B_u$	6.45	6.55	6.64	6.67	6.71	6.77	6.82
Octatetraene	$1\,^3B_u$	2.50	2.55	2.58	2.59	2.6	2.62	2.64
Octatetraene	$1\,^3A_g$	3.93	4.00	4.03	4.05	4.06	4.09	4.11
Cyclopropene	$1\,^1B_1$	8.09	8.12	8.16	8.17	8.19	8.22	8.24
Cyclopropene	$1\,^1B_2$	9.25	9.32	9.39	9.42	9.45	9.5	9.55
Cyclopropene	$1\,^3B_2$	4.76	4.77	4.77	4.78	4.78	4.78	4.78
Cyclopropene	$1\,^3B_1$	7.84	7.88	7.92	7.93	7.94	7.97	7.99
Cyclopentadiene	$1\,^1B_2$	7.53	7.64	7.73	7.78	7.82	7.9	7.97
Cyclopentadiene	$2\,^1A_1$	7.09	7.19	7.26	7.29	7.32	7.37	7.41
Cyclopentadiene	$3\,^1A_1$	10.30	10.50	10.65	10.72	10.78	10.89	10.98
Cyclopentadiene	$1\,^3B_2$	3.73	3.75	3.78	3.80	3.81	3.83	3.85
Norbornadiene	$1\,^1A_2$	7.54	7.74	7.85	7.90	7.95	8.05	8.14

Table S13: . . . continued

MB Basis Set		Excitation Energy V^{calc} [eV]						
**Molecule**	**State**	**0**	**0.10**	**0.20**	**0.25**	**0.30**	**0.40**	**0.50**
Norbornadiene	$1\,^1B_2$	8.08	8.31	8.44	8.51	8.57	8.68	8.79
Norbornadiene	$2\,^1B_2$	9.98	10.30	10.47	10.54	10.61	10.73	10.85
Norbornadiene	$2\,^1A_2$	9.52	9.75	9.88	9.94	10	10.11	10.21
Norbornadiene	$1\,^3A_2$	4.39	4.52	4.55	4.56	4.57	4.6	4.62
Norbornadiene	$1\,^3B_2$	4.66	4.78	4.81	4.83	4.84	4.87	4.89
Benzene	$2\,^1A'$	5.41	5.45	5.50	5.52	5.53	5.57	5.6
Benzene	$3\,^1A'$	8.33	8.45	8.56	8.60	8.65	8.73	8.8
Benzene	$4\,^1A'$	8.92	8.99	9.04	9.07	9.09	9.13	9.16
Benzene	$5\,^1A'$	8.92	9.01	9.07	9.09	9.12	9.16	9.2
Benzene	$6\,^1A'$	9.04	9.20	9.34	9.40	9.46	9.56	9.66
Benzene	$7\,^1A'$	9.04	9.20	9.34	9.40	9.46	9.57	9.66
Benzene	$1\,^3A'$	4.29	4.32	4.35	4.36	4.38	4.4	4.42
Benzene	$2\,^3A'$	5.46	5.54	5.59	5.61	5.63	5.67	5.7
Benzene	$3\,^3A'$	5.46	5.54	5.59	5.62	5.64	5.68	5.71
Benzene	$4\,^3A'$	7.59	7.70	7.80	7.84	7.88	7.96	8.02
Naphthalene	$1\,^1B_{3u}$	4.50	4.57	4.61	4.63	4.65	4.69	4.72
Naphthalene	$1\,^1B_{2u}$	6.35	6.44	6.51	6.55	6.58	6.64	6.69
Naphthalene	$2\,^1A_g$	6.28	6.37	6.42	6.45	6.48	6.53	6.57
Naphthalene	$1\,^1B_{1g}$	6.85	7.01	7.08	7.11	7.15	7.2	7.25
Naphthalene	$2\,^1B_{3u}$	7.59	7.75	7.88	7.93	7.99	8.08	8.17
Naphthalene	$2\,^1B_{2u}$	7.96	8.06	8.15	8.20	8.23	8.3	8.37
Naphthalene	$3\,^1A_g$	7.28	7.41	7.49	7.52	7.56	7.61	7.67
Naphthalene	$3\,^1B_{2u}$	9.33	9.46	9.53	9.57	9.6	9.66	9.72
Furan	$1\,^1B_2$	8.23	8.36	8.46	8.50	8.54	8.61	8.68
Furan	$3\,^1A_1$	9.62	9.85	10.01	10.08	10.14	10.26	10.36
Furan	$1\,^3B_2$	4.69	4.71	4.73	4.74	4.76	4.78	4.8
Furan	$1\,^3A_1$	5.99	6.04	6.08	6.10	6.12	6.15	6.18
Pyrrole	$1\,^1B_2$	8.19	8.32	8.42	8.47	8.51	8.58	8.65
Pyrrole	$3\,^1A_1$	9.66	9.87	10.01	10.07	10.13	10.23	10.32
Pyrrole	$1\,^3B_2$	5.15	5.18	5.21	5.22	5.23	5.26	5.28
Pyrrole	$1\,^3A_1$	6.17	6.23	6.28	6.31	6.33	6.37	6.4
Imidazole	$2\,^1A'$	7.32	7.41	7.49	7.53	7.56	7.63	7.69
Imidazole	$3\,^1A'$	8.36	8.47	8.56	8.60	8.64	8.71	8.77
Pyridine	$2\,^1A_1$	8.44	8.59	8.70	8.74	8.79	8.87	8.94
Pyridine	$3\,^1A_1$	9.09	9.20	9.27	9.31	9.34	9.39	9.44
Pyridine	$1\,^1B_1$	4.83	4.87	4.90	4.92	4.93	4.96	4.98
Pyridine	$1\,^1A_2$	5.09	5.14	5.18	5.20	5.22	5.25	5.28
Pyridine	$1\,^3A_1$	4.35	4.39	4.42	4.43	4.44	4.47	4.49

Table S13: . . . continued

MB Basis Set		Excitation Energy V^{calc} [eV]						
**Molecule**	**State**	**0**	**0.10**	**0.20**	**0.25**	**0.30**	**0.40**	**0.50**
Pyridine	$1\,^3B_2$	5.56	5.65	5.70	5.73	5.75	5.79	5.83
Pyrazine	$1\,^1B_{2u}$	5.62	5.67	5.71	5.73	5.75	5.79	5.82
Pyrazine	$1\,^1B_{1u}$	8.52	8.63	8.73	8.77	8.81	8.88	8.95
Pyrazine	$2\,^1B_{1u}$	9.49	9.67	9.82	9.88	9.95	10.06	10.16
Pyrazine	$2\,^1B_{2u}$	9.34	9.44	9.52	9.55	9.58	9.65	9.7
Pyrazine	$1\,^1B_{3u}$	3.96	4.02	4.07	4.09	4.11	4.15	4.19
Pyrazine	$1\,^1B_{2g}$	5.51	5.55	5.59	5.61	5.63	5.66	5.69
Pyrazine	$1\,^1B_{1g}$	5.93	6.06	6.13	6.16	6.2	6.25	6.3
Pyrimidine	$1\,^1B_2$	5.64	5.69	5.73	5.75	5.77	5.81	5.84
Pyrimidine	$2\,^1A_1$	8.61	8.77	8.87	8.92	8.96	9.04	9.11
Pyrimidine	$3\,^1A_1$	8.77	8.90	9.00	9.04	9.08	9.15	9.22
Pyrimidine	$2\,^1B_2$	8.76	8.87	8.94	8.98	9.01	9.06	9.11
Pyrimidine	$1\,^1B_1$	4.11	4.16	4.21	4.23	4.25	4.28	4.32
Pyrimidine	$1\,^1A_2$	4.28	4.35	4.40	4.43	4.45	4.49	4.53
Pyridazine	$2\,^1A_1$	5.49	5.53	5.58	5.60	5.62	5.65	5.69
Pyridazine	$1\,^1B_2$	8.16	8.45	8.62	8.69	8.75	8.86	8.95
Pyridazine	$2\,^1B_2$	8.83	8.99	9.09	9.13	9.16	9.23	9.28
Pyridazine	$3\,^1A_1$	7.79	7.81	7.84	7.85	7.87	7.89	7.91
Pyridazine	$1\,^1B_1$	3.67	3.73	3.77	3.79	3.81	3.85	3.88
Pyridazine	$2\,^1A_2$	5.52	5.62	5.69	5.72	4.08	4.12	4.16
Pyridazine	$2\,^1B_1$	6.16	6.28	6.36	6.39	6.43	6.48	6.54
Triazine	$2\,^1A'$	5.86	5.91	5.95	5.97	5.99	6.03	6.07
Triazine	$3\,^1A'$	7.61	7.74	7.84	7.88	7.92	8	8.07
Triazine	$4\,^1A'$	8.35	8.51	8.61	8.65	8.7	8.77	8.85
Triazine	$2\,^1A''$	4.21	4.32	4.41	4.45	4.48	4.54	4.6
Triazine	$3\,^1A''$	4.22	4.33	4.41	4.45	4.48	4.55	4.6
Triazine	$4\,^1A''$	4.24	4.40	4.52	4.56	4.61	4.69	4.77
Tetrazine	$1\,^1B_{2u}$	5.57	5.61	5.65	5.67	5.69	5.73	5.76
Tetrazine	$1\,^1B_{1u}$	7.34	7.48	7.58	7.63	7.66	7.74	7.8
Tetrazine	$2\,^1B_{1u}$	8.60	8.86	9.03	9.10	9.16	9.28	9.39
Tetrazine	$2\,^1B_{2u}$	7.39	7.52	7.61	7.65	7.69	7.76	7.82
Tetrazine	$1\,^1B_{3u}$	2.26	2.32	2.37	2.39	2.41	2.45	2.48
Tetrazine	$1\,^1A_u$	2.81	2.89	2.96	2.98	3.01	3.06	3.11
Tetrazine	$2\,^1B_{2g}$	5.81	5.95	6.04	6.08	6.12	6.19	6.25
Tetrazine	$2\,^1B_{1g}$	6.23	6.35	6.45	6.49	6.53	6.61	6.67
Tetrazine	$2\,^1B_{3u}$	6.21	6.31	6.41	6.45	6.49	6.56	6.62
Tetrazine	$1\,^3B_{3u}$	1.40	1.46	1.51	1.53	1.55	1.6	1.64
Tetrazine	$1\,^3A_u$	2.46	2.55	2.62	2.65	2.68	2.74	2.79

Table S13: ... continued

**MB Basis Set**		**Excitation Energy V^{calc} [eV]**						
**Molecule**	**State**	**0**	**0.10**	**0.20**	**0.25**	**0.30**	**0.40**	**0.50**
Tetrazine	$1\,^3B_{1g}$	3.98	4.06	4.11	4.14	4.16	4.2	4.24
Formaldehyde	$1\,^1A_2$	3.76	3.76	3.76	3.77	3.77	3.77	3.77
Formaldehyde	$1\,^3A_2$	3.24	3.24	3.25	3.26	3.26	3.26	3.27
Formaldehyde	$1\,^3A_1$	5.77	5.76	5.76	5.75	5.75	5.74	5.73
Acetone	$1\,^1A_2$	4.13	4.15	4.17	4.18	4.19	4.2	4.22
Acetone	$1\,^3A_2$	3.74	3.77	3.79	3.80	3.81	3.83	3.85
Acetone	$1\,^3A_1$	5.75	5.76	5.77	5.78	5.78	5.79	5.8
Benzoquinone	$1\,^1B_{1g}$	2.46	2.55	2.60	2.62	2.64	2.68	2.71
Benzoquinone	$1\,^1A_u$	2.53	2.60	2.64	2.66	2.68	2.72	2.75
Benzoquinone	$1\,^1B_{3g}$	5.63	5.81	5.91	5.95	5.99	6.06	6.11
Benzoquinone	$1\,^1B_{1u}$	6.88	7.05	7.16	7.21	7.25	7.33	7.4
Benzoquinone	$2\,^1B_{1u}$	7.91	8.15	8.25	8.28	8.31	8.37	8.41
Benzoquinone	$1\,^3B_{1g}$	2.22	2.31	2.37	2.40	2.42	2.46	2.5
Benzoquinone	$1\,^3A_u$	2.31	2.39	2.44	2.46	2.48	2.52	2.55
Formamide	$1\,^1A''$	5.19	5.18	5.17	5.17	5.17	5.16	5.15
Formamide	$2\,^1A'$	8.38	8.46	8.54	8.57	8.6	8.66	8.71
Acetamide	$1\,^1A''$	5.28	5.27	5.26	5.26	5.26	5.25	5.25
Acetamide	$2\,^1A'$	8.28	8.37	8.44	8.47	8.5	8.56	8.61
Acetamide	$3\,^1A'$	11.78	11.85	11.91	11.94	11.97	12.02	12.07
Propanamide	$1\,^1A''$	5.32	5.31	5.31	5.31	5.3	5.3	5.3
Propanamide	$2\,^1A'$	8.30	8.39	8.46	8.50	8.53	8.59	8.64
Propanamide	$3\,^1A'$	11.77	11.84	11.91	11.94	11.97	12.02	12.07
Cytosine	$2\,^1A'$	4.68	4.75	4.80	4.83	4.85	4.89	4.92
Cytosine	$1\,^1A''$	4.67	4.73	4.78	4.80	4.82	4.86	4.89
Cytosine	$3\,^1A'$	5.80	5.94	6.02	6.06	6.1	6.17	6.23
Cytosine	$4\,^1A'$	7.01	7.18	7.29	7.34	7.38	7.46	7.53
Cytosine	$5\,^1A'$	7.26	7.39	7.49	7.53	7.57	7.65	7.72
Thymine	$1\,^1A''$	4.56	4.58	4.61	4.62	4.63	4.66	4.68
Thymine	$2\,^1A'$	5.62	5.74	5.82	5.85	5.88	5.94	5.99
Thymine	$3\,^1A'$	6.35	6.48	6.57	6.60	6.63	6.69	6.74
Thymine	$4\,^1A'$	7.17	7.36	7.48	7.53	7.57	7.65	7.73
Thymine	$5\,^1A'$	7.99	8.19	8.32	8.38	8.44	8.53	8.62
Uracil	$1\,^1A''$	4.48	4.51	4.54	4.56	4.57	4.59	4.61
Uracil	$2\,^1A'$	5.63	5.74	5.82	5.85	5.88	5.94	5.99
Uracil	$3\,^1A'$	6.25	6.39	6.46	6.50	6.53	6.59	6.64
Uracil	$4\,^1A'$	7.30	7.48	7.60	7.65	7.69	7.77	7.85
Uracil	$5\,^1A'$	8.65	8.80	8.90	8.94	8.98	9.05	9.11
Adenine	$2\,^1A'$	5.07	5.19	5.27	5.30	5.33	5.39	5.44

Table S13: . . . continued

MB Basis Set		Excitation Energy V^{calc} [eV]						
**Molecule**	**State**	**0**	**0.10**	**0.20**	**0.25**	**0.30**	**0.40**	**0.50**
Adenine	$3\ ^1A'$	6.11	6.28	6.38	6.42	6.46	6.53	6.6
Adenine	$1\ ^1A''$	4.68	4.75	4.80	4.83			
Adenine	$4\ ^1A'$	7.19	7.36	7.46	7.50	7.54	7.61	7.68
Adenine	$5\ ^1A'$	7.45	7.61	7.72	7.76	7.8	7.88	7.95
Adenine	$6\ ^1A'$	7.66	7.85	7.97	8.02	8.07	8.15	8.23
Adenine	$7\ ^1A'$	8.27	8.42	8.53	8.58	8.62	8.7	8.78

Table S14: CASPT2 vertical excitation energies for the Thiel benchmark set obtained using the ANO-RCC-VDZ basis set and different IPEA shift parameters (ε = 0, 0.10, 0.20, 0.25, 0.30, 0.40, and 0.50 a.u.).

VDZ Basis Set		Excitation Energy V^{calc} [eV]						
**Molecule**	**State**	**0**	**0.10**	**0.20**	**0.25**	**0.30**	**0.40**	**0.50**
Ethene	$1\ ^1B_{1u}$	8.92	9.00	9.08	9.11	9.15	9.21	9.27
Ethene	$1\ ^3B_{1u}$	4.56	4.57	4.59	4.59	4.60	4.61	4.62
Butadiene	$1\ ^1B_u$	6.76	6.89	7.01	7.06	7.11	7.21	7.29
Butadiene	$1\ ^3B_u$	3.33	3.37	3.47	3.45	3.48	3.53	3.57
Butadiene	$1\ ^3A_g$	5.23	5.28	5.35	5.37	5.40	5.45	5.49
Hexatriene	$1\ ^1B_u$	5.56	5.71	5.83	5.89	5.94	6.04	6.12
Hexatriene	$2\ ^1A_g$	5.39	5.48	5.56	5.60	5.63	5.70	5.76
Hexatriene	$1\ ^3B_u$	2.64	2.72	2.77	2.80	2.82	2.86	2.90
Hexatriene	$1\ ^3A_g$	4.28	4.37	4.43	4.47	4.50	4.55	4.60
Octatetraene	$2\ ^1A_g$	4.55	4.66	4.73	4.77	4.80	4.87	4.92
Octatetraene	$1\ ^3B_u$	2.26	2.35	2.40	2.42	2.45	2.49	2.52
Octatetraene	$1\ ^3A_g$	3.62	3.74	3.81	3.84	3.87	3.92	3.97
Cyclopropene	$1\ ^1B_1$	7.05	7.12	7.18	7.20	7.23	7.28	7.33
Cyclopropene	$1\ ^1B_2$	7.35	7.46	7.56	7.61	7.66	7.74	7.82
Cyclopropene	$1\ ^3B_2$	4.35	4.38	4.40	4.42	4.43	4.45	4.46
Cyclopropene	$1\ ^3B_1$	6.78	6.86	6.92	6.91	6.97	7.02	7.07
Cyclopentadiene	$1\ ^1B_2$	5.97	6.08	6.20	6.25	6.30	6.40	6.49
Cyclopentadiene	$2\ ^1A_1$	6.25	6.40	6.54	6.59	6.65	6.74	6.82
Cyclopentadiene	$3\ ^1A_1$	8.81	9.04	9.23	9.31	9.38	9.52	9.64
Cyclopentadiene	$1\ ^3B_2$	3.42	3.47	3.52	3.55	3.57	3.62	3.66
Norbornadiene	$1\ ^1A_2$	5.85	5.95	6.07	6.10	6.19	6.29	6.39
Norbornadiene	$1\ ^1B_2$	6.43	6.59	6.75	6.80	6.91	7.05	7.18
Norbornadiene	$2\ ^1B_2$	7.78	7.99	8.17	8.22	8.33	8.47	8.60
Norbornadiene	$2\ ^1A_2$	7.90	8.03	8.17	8.21	8.31	8.43	8.55
Norbornadiene	$1\ ^3A_2$	3.99	4.05	4.11	4.11	4.17	4.22	4.27

Table S14: . . . continued

VDZ Basis Set		Excitation Energy V^{calc} [eV]						
Molecule	State	0	0.10	0.20	0.25	0.30	0.40	0.50
Norbornadiene	$1\ ^3B_2$	4.40	4.44	4.51	4.51	4.57	4.63	4.68
Benzene	$2\ ^1A'$	5.01	5.10	5.18	5.21	5.25	5.32	5.38
Benzene	$3\ ^1A'$	6.73	6.90	7.04	7.11	7.18	7.29	7.40
Benzene	$4\ ^1A'$	7.31	7.56	7.76	7.85	7.94	8.10	8.24
Benzene	$5\ ^1A'$	7.31	7.56	7.76	7.85	7.94	8.10	8.25
Benzene	$6\ ^1A'$	8.29	8.43	8.53	8.58	8.62	8.70	8.78
Benzene	$7\ ^1A'$	8.29	8.44	8.55	8.61	8.65	8.74	8.83
Benzene	$1\ ^3A'$	4.10	4.18	4.24	4.27	4.30	4.35	4.40
Benzene	$2\ ^3A'$	4.90	5.04	5.13	5.17	5.21	5.29	5.36
Benzene	$3\ ^3A'$	4.90	5.04	5.14	5.18	5.22	5.30	5.37
Benzene	$4\ ^3A'$	6.05	6.23	6.38	6.45	6.51	6.63	6.74
Naphthalene	$1\ ^1B_{3u}$	4.17	4.28	4.36	4.40	4.44	4.50	4.57
Naphthalene	$1\ ^1B_{2u}$	5.02	5.15	5.26	5.31	5.35	5.44	5.52
Naphthalene	$2\ ^1A_g$	5.82	5.97	6.08	6.13	6.18	6.28	6.36
Naphthalene	$1\ ^1B_{1g}$	6.03	6.25	6.38	6.44	6.49	6.59	6.68
Naphthalene	$2\ ^1B_{3u}$	6.15	6.40	6.59	6.68	6.76	6.90	7.04
Naphthalene	$2\ ^1B_{2u}$	6.61	6.76	6.88	6.94	7.00	7.10	7.19
Naphthalene	$3\ ^1A_g$	6.61	6.83	6.97	7.03	7.09	7.20	7.31
Naphthalene	$3\ ^1B_{2u}$	8.50	8.74	8.93	9.01	9.09	9.23	9.36
Furan	$1\ ^1B_2$	7.05	7.21	7.34	7.33	7.46	7.56	7.65
Furan	$3\ ^1A_1$	8.19	8.56	8.82	8.93	9.03	9.21	9.37
Furan	$1\ ^3B_2$	4.39	4.43	4.48	4.50	4.52	4.56	4.60
Furan	$1\ ^3A_1$	5.68	5.76	5.84	5.87	5.90	5.97	6.03
Pyrrole	$1\ ^1B_2$	6.86	7.02	7.16	7.22	7.28	7.38	7.48
Pyrrole	$3\ ^1A_1$	8.33	8.60	8.79	8.87	8.95	9.09	9.22
Pyrrole	$1\ ^3B_2$	4.64	4.70	4.75	4.78	4.80	4.85	4.89
Pyrrole	$1\ ^3A_1$	5.62	5.73	5.82	5.86	5.90	5.97	6.04
Imidazole	$2\ ^1A'$	6.38	6.56	6.70	6.76	6.83	6.93	7.03
Imidazole	$3\ ^1A'$	7.05	7.21	7.34	7.40	7.46	7.57	7.67
Pyridine	$2\ ^1A_1$	6.41	6.82	7.00	7.09	7.18	7.33	7.47
Pyridine	$3\ ^1A_1$	7.52	7.79	8.01	8.10	8.19	8.35	8.50
Pyridine	$1\ ^1B_1$	5.04	5.11	5.17	5.20	5.58	5.64	5.69
Pyridine	$1\ ^1A_2$	5.36	5.44	5.51	5.50	5.32	5.36	5.40
Pyridine	$1\ ^3A_1$	4.22	4.30	4.36	4.39	4.42	4.48	4.53
Pyridine	$1\ ^3B_2$	4.82	5.00	5.11	5.16	5.20	5.28	5.36
Pyrazine	$1\ ^1B_{2u}$	4.80	4.92	5.01	5.05	5.10	5.17	5.24
Pyrazine	$1\ ^1B_{1u}$	7.06	7.21	7.34	7.40	7.46	7.57	7.67
Pyrazine	$2\ ^1B_{1u}$	7.77	8.01	8.21	8.29	8.38	8.54	8.68

Table S14: . . . continued

VDZ Basis Set		Excitation Energy V^{calc} [eV]						
**Molecule**	**State**	**0**	**0.10**	**0.20**	**0.25**	**0.30**	**0.40**	**0.50**
Pyrazine	$2\ ^1B_{2u}$	7.58	7.81	8.01	8.09	8.18	8.33	8.48
Pyrazine	$1\ ^1B_{3u}$	3.80	3.90	3.99	4.03	4.07	4.15	4.22
Pyrazine	$1\ ^1B_{2g}$	5.46	5.55	5.63	5.66	4.60	4.71	4.81
Pyrazine	$1\ ^1B_{1g}$	5.92	6.19	6.31	6.38	6.45	6.56	6.67
Pyrimidine	$1\ ^1B_2$	5.33	5.42	5.51	5.55	5.59	5.67	5.74
Pyrimidine	$2\ ^1A_1$	6.74	7.03	7.20	7.27	7.34	7.46	7.57
Pyrimidine	$3\ ^1A_1$	7.46	7.66	7.84	7.92	7.99	8.13	8.25
Pyrimidine	$2\ ^1B_2$	7.76	8.03	8.24	8.34	8.43	8.59	8.74
Pyrimidine	$1\ ^1B_1$	4.35	4.44	4.52	4.56	4.59	4.66	4.72
Pyrimidine	$1\ ^1A_2$	4.65	4.76	4.85	4.90	4.94	5.02	5.09
Pyridazine	$2\ ^1A_1$	5.13	5.22	5.30	5.34	5.38	5.45	5.52
Pyridazine	$1\ ^1B_2$	6.55	6.84	7.03	7.11	7.19	7.33	7.45
Pyridazine	$2\ ^1B_2$	7.35	7.59	7.79	7.88	7.96	8.11	8.25
Pyridazine	$3\ ^1A_1$	8.03	8.09	8.14	8.16	8.19	8.24	8.28
Pyridazine	$1\ ^1B_1$	3.55	3.65	3.74	3.78	3.82	3.89	3.96
Pyridazine	$2\ ^1A_2$	5.41	5.57	5.68	5.74	5.78	5.88	5.96
Pyridazine	$2\ ^1B_1$	6.17	6.35	6.48	6.54	6.59	6.69	6.78
Triazine	$2\ ^1A'$	5.73	5.81	5.89	5.92	5.96	6.03	6.09
Triazine	$3\ ^1A'$	7.37	7.56	7.72	7.79	7.85	7.97	8.08
Triazine	$4\ ^1A'$	8.41	8.61	8.77	8.85	8.92	9.05	9.17
Triazine	$2\ ^1A''$	4.25	4.55	4.68	4.73	4.79	4.91	5.03
Triazine	$3\ ^1A''$	4.42	4.57	4.71	4.77	4.83	4.94	5.04
Triazine	$4\ ^1A''$	4.46	4.59	4.72	4.78	4.84	4.95	5.05
Tetrazine	$1\ ^1B_{2u}$	4.90	5.03	5.13	5.18	5.23	5.31	5.39
Tetrazine	$1\ ^1B_{1u}$	6.84	7.18	7.43	7.53	7.63	7.80	7.96
Tetrazine	$2\ ^1B_{1u}$	7.02	7.29	7.51	7.60	7.69	7.86	8.01
Tetrazine	$2\ ^1B_{2u}$	7.23	7.45	7.61	7.68	7.75	7.88	8.00
Tetrazine	$1\ ^1B_{3u}$	1.94	2.06	2.15	2.19	2.23	2.31	2.38
Tetrazine	$1\ ^1A_u$	2.90	3.08	3.21	3.27	3.32	3.42	3.52
Tetrazine	$2\ ^1B_{2g}$	5.52	5.73	5.90	5.97	6.04	6.18	6.29
Tetrazine	$2\ ^1B_{1g}$	5.89	6.08	6.26	6.34	6.42	6.56	6.69
Tetrazine	$2\ ^1B_{3u}$	6.12	6.33	6.49	6.56	6.62	6.75	6.86
Tetrazine	$1\ ^3B_{3u}$	1.10	1.22	1.33	1.37	1.42	1.50	1.58
Tetrazine	$1\ ^3A_u$	2.64	2.83	2.97	3.03	3.09	3.21	3.31
Tetrazine	$1\ ^3B_{1g}$	3.67	3.82	3.93	3.98	4.03	4.12	4.21
Formaldehyde	$1\ ^1A_2$	4.07	4.07	4.08	4.09	4.09	4.10	4.11
Formaldehyde	$1\ ^3A_2$	3.62	3.64	3.67	3.68	3.69	3.71	3.72
Formaldehyde	$1\ ^3A_1$	6.04	6.03	6.02	6.02	6.01	6.00	5.99

Table S14: ... continued

**VDZ Basis Set**		**Excitation Energy V^{calc} [eV]**						
**Molecule**	**State**	**0**	**0.10**	**0.20**	**0.25**	**0.30**	**0.40**	**0.50**
Acetone	$1\ ^1A_2$	4.45	4.49	4.54	4.56	4.57	4.61	4.64
Acetone	$1\ ^3A_2$	4.18	4.24	4.29	4.28	4.33	4.37	4.41
Acetone	$1\ ^3A_1$	6.15	6.19	6.21	6.23	6.24	6.26	6.29
Benzoquinone	$1\ ^1B_{1g}$	2.46	2.62	2.73	2.80	2.83	2.92	2.99
Benzoquinone	$1\ ^1A_u$		2.68	2.78	2.85	2.87	2.95	3.02
Benzoquinone	$1\ ^1B_{3g}$	4.19	4.48	4.64	4.72	4.76	4.87	4.97
Benzoquinone	$1\ ^1B_{1u}$	4.72	5.00	5.18	5.29	5.34	5.47	5.60
Benzoquinone	$2\ ^1B_{1u}$	6.55	6.91	7.21	7.36	7.46	7.69	7.90
Benzoquinone	$1\ ^3B_{1g}$	2.28	2.46	2.59	2.66	2.69	2.78	2.86
Benzoquinone	$1\ ^3A_u$	2.41	2.55	2.66	2.73	2.76	2.84	2.92
Formamide	$1\ ^1A''$	5.79	5.80	5.82	5.82	5.83	5.84	5.85
Formamide	$2\ ^1A'$	7.07	7.27	7.42	7.48	7.54	7.65	7.74
Formamide	$1\ ^3A''$	5.51	5.55	5.58	5.59	5.60	5.63	5.65
Acetamide	$1\ ^1A''$	5.82	5.84	5.86	5.86	5.87	5.89	5.90
Acetamide	$2\ ^1A'$	6.87	7.05	7.20	7.26	7.32	7.43	7.53
Acetamide	$3\ ^1A'$	10.0	10.22	10.36	10.42	10.48	10.60	10.70
Propanamide	$1\ ^1A''$	5.86	5.87	5.89	5.90	5.91	5.92	5.94
Propanamide	$2\ ^1A'$	6.80	6.99	7.14	7.21	7.27	7.39	7.49
Propanamide	$3\ ^1A'$	9.85	10.04	10.19	10.26	10.33	10.45	10.56
Cytosine	$2\ ^1A'$	4.57	4.68	4.77	4.81	4.85	4.92	4.99
Cytosine	$1\ ^1A''$	5.16	5.28	5.37	5.41	5.45	5.53	5.61
Cytosine	$3\ ^1A'$	5.34	5.55	5.70	5.76	5.82	5.94	6.05
Cytosine	$4\ ^1A'$	6.05	6.31	6.49	6.57	6.65	6.79	6.92
Cytosine	$5\ ^1A'$	6.79	7.02	7.18	7.25	7.32	7.44	7.56
Thymine	$2\ ^1A'$	4.95	5.12	5.25	5.31	5.36	5.47	5.56
Thymine	$3\ ^1A'$	5.91	6.16	6.32	6.39	6.46	6.58	6.69
Thymine	$4\ ^1A'$	6.14	6.40	6.59	6.67	6.74	6.88	7.01
Thymine	$5\ ^1A'$	6.93	7.21	7.41	7.49	7.57	7.72	7.85
Uracil	$1\ ^1A''$	5.13	5.21	5.27	5.30	5.33	5.38	5.43
Uracil	$2\ ^1A'$	5.07	5.24	5.37	5.43	5.49	5.59	5.69
Uracil	$3\ ^1A'$	5.98	6.17	6.31	6.38	6.44	6.55	6.65
Uracil	$4\ ^1A'$	6.35	6.60	6.78	6.86	6.93	7.07	7.19
Uracil	$5\ ^1A'$	6.89	7.18	7.37	7.45	7.53	7.68	7.81
Adenine	$2\ ^1A'$	4.98	5.16	5.29	5.35	5.41	5.51	5.60
Adenine	$3\ ^1A'$	5.27	5.46	5.59	5.65	5.70	5.80	5.90
Adenine	$4\ ^1A'$	6.10	6.56	6.77	6.85	6.94	7.09	7.23
Adenine	$5\ ^1A'$	6.36	6.88	7.06	7.13	7.19	7.32	7.42
Adenine	$6\ ^1A'$	6.75	7.02	7.20	7.28	7.35	7.49	7.61

Table S14: ... continued

VDZ Basis Set		Excitation Energy V^{calc} [eV]						
**Molecule**	**State**	**0**	**0.10**	**0.20**	**0.25**	**0.30**	**0.40**	**0.50**
Adenine	$7\,^1A'$	7.05	7.79	7.98	8.06	8.14	8.28	8.42

Table S15: CASPT2 vertical excitation energies for the Thiel benchmark set obtained using the ANO-RCC-VDZP basis set and different IPEA shift parameters (ε = 0, 0.10, 0.20, 0.25, 0.30, 0.40, and 0.50 a.u.).

VDZP Basis Set		Excitation Energy V^{calc} [eV]						
**Molecule**	**State**	**0**	**0.10**	**0.20**	**0.25**	**0.30**	**0.40**	**0.50**
Ethene	$1\,^1B_{1u}$	8.36	8.47	8.57	8.61	8.66	8.74	8.82
Ethene	$1\,^3B_{1u}$	4.40	4.45	4.48	4.50	4.52	4.55	4.58
Butadiene	$1\,^1B_u$	6.18	6.35	6.50	6.57	6.63	6.76	6.87
Butadiene	$1\,^3B_u$	3.19	3.28	3.36	3.40	3.44	3.51	3.58
Butadiene	$1\,^3A_g$	5.00	5.10	5.20	5.25	5.30	5.38	5.46
Hexatriene	$1\,^1B_u$	4.95	5.14	5.30	5.37	5.43	5.55	5.66
Hexatriene	$2\,^1A_g$	5.15	5.29	5.41	5.46	5.52	5.62	5.72
Hexatriene	$1\,^3B_u$	2.69	2.75	2.77	2.81	2.79	2.86	2.92
Hexatriene	$1\,^3A_g$	4.08	4.22	4.33	4.38	4.42	4.52	4.60
Octatetraene	$2\,^1A_g$	4.34	4.49	4.61	4.67	4.72	4.82	4.91
Octatetraene	$1\,^1B_u$	4.35	4.52	4.67	4.73	4.80	4.91	5.02
Octatetraene	$2\,^1B_u$	5.43	5.63	5.79	5.86	5.93	6.06	6.18
Octatetraene	$1\,^3B_u$	2.15	2.27	2.35	2.39	2.43	2.50	2.56
Octatetraene	$1\,^3A_g$	3.45	3.61	3.72	3.78	3.82	3.92	4.00
Cyclopropene	$1\,^1B_1$	6.61	6.70	6.78	6.82	6.86	6.94	7.01
Cyclopropene	$1\,^1B_2$	6.70	6.85	6.97	7.04	7.09	7.20	7.30
Cyclopropene	$1\,^3B_2$	4.21	4.26	4.31	4.34	4.36	4.40	4.44
Cyclopropene	$1\,^3B_1$	6.29	6.40	6.49	6.53	6.58	6.65	6.72
Cyclopentadiene	$1\,^1B_2$	5.39	5.53	5.67	5.74	5.80	5.92	6.03
Cyclopentadiene	$2\,^1A_1$	5.87	6.09	6.28	6.36	6.43	6.57	6.70
Cyclopentadiene	$3\,^1A_1$	8.17	8.49	8.88	8.97	8.96	9.16	9.33
Cyclopentadiene	$1\,^3B_2$	3.28	3.35	3.44	3.47	3.51	3.58	3.65
Norbornadiene	$1\,^1A_2$	5.07	5.38	5.50	5.57	5.61	5.76	5.87
Norbornadiene	$1\,^1B_2$	5.98	6.37	6.56	6.66	6.73	6.93	7.10
Norbornadiene	$2\,^1B_2$	7.62	7.99	8.14	8.22	8.27	8.44	8.57
Norbornadiene	$2\,^1A_2$	7.21	7.55	7.70	7.77	7.83	8.00	8.13
Benzene	$2\,^1A'$	4.81	4.94	5.06	5.12	5.18	5.29	5.39
Benzene	$3\,^1A'$	6.01	6.23	6.42	6.51	6.59	6.75	6.89
Benzene	$4\,^1A'$	6.65	6.95	7.21	7.33	7.44	7.64	7.82
Benzene	$5\,^1A'$	6.65	6.95	7.21	7.33	7.44	7.64	7.83

Table S15: ... continued

**VDZP Basis Set**		**Excitation Energy V^{calc} [eV]**						
**Molecule**	**State**	**0**	**0.10**	**0.20**	**0.25**	**0.30**	**0.40**	**0.50**
Benzene	$6\ ^1A'$	7.87	8.08	8.23	8.30	8.37	8.50	8.62
Benzene	$7\ ^1A'$	7.87	8.09	8.25	8.33	8.40	8.54	8.67
Benzene	$1\ ^3A'$	3.94	4.07	4.17	4.22	4.27	4.36	4.44
Benzene	$2\ ^3A'$	4.54	4.73	4.87	4.93	5.00	5.11	5.22
Benzene	$3\ ^3A'$	4.54	4.73	4.88	4.94	5.01	5.12	5.23
Benzene	$4\ ^3A'$	5.25	5.47	5.67	5.76	5.85	6.02	6.16
Naphthalene	$1\ ^1B_{3u}$	3.95	4.12	4.24	4.30	4.36	4.47	4.57
Naphthalene	$1\ ^1B_{2u}$	4.39	4.56	4.70	4.77	4.83	4.95	5.06
Naphthalene	$2\ ^1A_g$	5.55	5.76	5.93	6.01	6.09	6.23	6.37
Naphthalene	$1\ ^1B_{1g}$	5.54	5.83	6.02	6.10	6.18	6.33	6.47
Naphthalene	$2\ ^1B_{3u}$	5.52	5.83	6.08	6.19	6.29	6.48	6.66
Naphthalene	$2\ ^1B_{2u}$	5.95	6.15	6.32	6.40	6.47	6.61	6.74
Naphthalene	$3\ ^1A_g$	6.16	6.46	6.66	6.76	5.67	5.80	5.92
Naphthalene	$3\ ^1B_{2u}$	7.75	8.06	8.30	8.41	8.51	8.70	8.87
Furan	$1\ ^1B_2$	6.32	6.59	6.75	6.83	6.90	7.03	7.15
Furan	$3\ ^1A_1$	7.58	8.02	8.33	8.46	8.59	8.81	9.02
Furan	$1\ ^3B_2$	4.22	4.29	4.36	4.39	4.42	4.48	4.54
Furan	$1\ ^3A_1$	5.44	5.56	5.67	5.72	5.77	5.86	5.95
Pyrrole	$1\ ^1B_2$	6.31	6.52	6.71	6.79	6.86	7.01	7.14
Pyrrole	$3\ ^1A_1$	7.78	8.10	8.34	8.45	8.55	8.74	8.91
Pyrrole	$1\ ^3B_2$	4.44	4.52	4.60	4.64	4.68	4.75	4.82
Pyrrole	$1\ ^3A_1$	5.31	5.46	5.59	5.65	5.71	5.82	5.93
Imidazole	$2\ ^1A'$	5.84	6.08	6.17	6.35	6.44	6.60	6.74
Imidazole	$3\ ^1A'$	6.64	6.82	6.82	7.07	7.14	7.28	7.41
Pyridine	$2\ ^1A_1$	5.59	6.05	6.31	6.42	6.53	6.72	6.90
Pyridine	$3\ ^1A_1$	6.90	7.23	7.49	7.61	7.72	7.93	8.13
Pyridine	$1\ ^1B_1$	5.01	5.11	5.19	5.23	5.60	5.68	5.76
Pyridine	$1\ ^1A_2$	5.30	5.41	5.51	5.56	5.20	5.27	5.34
Pyridine	$1\ ^3A_1$	4.04	4.17	4.28	4.33	4.38	4.47	4.56
Pyridine	$1\ ^3B_2$	4.21	4.48	4.66	4.73	4.80	4.93	5.05
Pyrazine	$1\ ^1B_{2u}$	4.56	4.73	4.87	4.94	5.00	5.12	5.22
Pyrazine	$1\ ^1B_{1u}$	6.47	6.65	6.82	6.90	6.97	7.11	7.24
Pyrazine	$2\ ^1B_{1u}$	7.29	7.57	7.81	7.92	8.02	8.21	8.38
Pyrazine	$2\ ^1B_{2u}$	7.20	7.46	7.69	7.80	7.90	8.08	8.25
Pyrazine	$1\ ^1B_{3u}$	3.84	3.99	4.11	4.17	4.23	4.33	4.43
Pyrazine	$1\ ^1B_{2g}$	5.43	5.57	5.69	5.74	5.80	5.90	6.00
Pyrazine	$1\ ^1B_{1g}$	5.86	6.15	6.36	6.45	6.54	6.71	6.86
Pyrimidine	$1\ ^1B_2$	5.02	5.18	5.32	5.39	5.45	5.57	5.68

Table S15: ... continued

**VDZP Basis Set**		**Excitation Energy V^{calc} [eV]**						
**Molecule**	**State**	**0**	**0.10**	**0.20**	**0.25**	**0.30**	**0.40**	**0.50**
Pyrimidine	$2\ ^1A_1$	6.10	6.40	6.58	6.66	6.74	6.88	7.01
Pyrimidine	$3\ ^1A_1$	6.73	7.00	7.23	7.34	7.44	7.63	7.80
Pyrimidine	$2\ ^1B_2$	6.81	7.21	7.51	7.66	7.79	8.03	8.24
Pyrimidine	$1\ ^1B_1$	4.27	4.39	4.50	4.55	4.60	4.70	4.79
Pyrimidine	$1\ ^1A_2$	4.58	4.72	4.84	4.90	4.96	5.06	5.17
Pyridazine	$2\ ^1A_1$	4.90	5.04	5.17	5.23	5.29	5.40	5.50
Pyridazine	$1\ ^1B_2$	5.75	6.09	6.31	6.41	6.50	6.67	6.83
Pyridazine	$2\ ^1B_2$	6.72	7.07	7.34	7.46	7.57	7.77	7.96
Pyridazine	$3\ ^1A_1$	5.76	7.34	7.61	7.73	7.84	8.05	8.23
Pyridazine	$1\ ^1B_1$	3.55	3.69	3.81	3.87	3.92	4.03	4.12
Pyridazine	$2\ ^1A_2$	5.52	5.70	5.84	5.91	5.98	6.10	6.22
Pyridazine	$2\ ^1B_1$	6.19	6.41	6.57	6.65	6.72	6.86	6.99
Triazine	$2\ ^1A'$	5.51	5.64	5.76	5.82	5.88	5.98	6.08
Triazine	$3\ ^1A'$	6.80	7.03	7.21	7.30	7.38	7.52	7.66
Triazine	$4\ ^1A'$	6.88	7.23	7.50	7.62	7.74	7.95	8.14
Triazine	$2\ ^1A''$	4.21	4.56	4.74	4.83	4.90	5.05	5.19
Triazine	$3\ ^1A''$	4.37	4.59	4.78	4.86	4.95	5.10	5.25
Triazine	$4\ ^1A''$	4.43	4.61	4.80	4.88	4.96	5.11	5.26
Tetrazine	$1\ ^1B_{2u}$	4.57	4.75	4.90	4.97	5.03	5.15	5.27
Tetrazine	$1\ ^1B_{1u}$	6.32	6.58	6.81	6.91	7.01	7.19	7.36
Tetrazine	$2\ ^1B_{1u}$	6.80	7.10	7.34	7.44	7.55	7.74	7.91
Tetrazine	$2\ ^1B_{2u}$	7.31	7.65	7.89	8.00	8.10	8.29	8.46
Tetrazine	$1\ ^1B_{3u}$	1.96	2.11	2.24	2.30	2.35	2.46	2.56
Tetrazine	$1\ ^1A_u$	3.06	3.29	3.46	3.54	3.61	3.75	3.87
Tetrazine	$2\ ^1B_{2g}$	5.35	5.77	5.99	6.09	6.19	6.37	6.54
Tetrazine	$2\ ^1B_{1g}$	5.85	6.13	6.38	6.49	6.60	6.80	6.98
Tetrazine	$2\ ^1B_{3u}$	6.27	6.53	6.74	6.84	6.93	7.10	7.26
Tetrazine	$1\ ^3B_{3u}$	1.26	1.42	1.55	1.61	1.67	1.79	1.89
Tetrazine	$1\ ^3A_u$	2.79	3.02	3.21	3.30	3.38	3.53	3.67
Tetrazine	$1\ ^3B_{1g}$	3.74	3.94	4.10	4.17	4.23	4.36	4.48
Formaldehyde	$1\ ^1A_2$	4.15	4.16	4.18	4.19	4.20	4.22	4.24
Formaldehyde	$1\ ^3A_2$	3.67	3.71	3.75	3.77	3.79	3.83	3.86
Formaldehyde	$1\ ^3A_1$	6.04	6.04	6.05	6.05	6.06	6.07	6.07
Acetone	$1\ ^1A_2$	4.46	4.54	4.61	4.64	4.67	4.73	4.79
Acetone	$1\ ^3A_2$	4.14	4.24	4.33	4.37	4.41	4.49	4.55
Acetone	$1\ ^3A_1$	6.17	6.23	6.29	6.32	6.34	6.39	6.44
Benzoquinone	$1\ ^1B_{1g}$	2.36	2.55	2.71	2.77	2.84	2.96	3.07
Benzoquinone	$1\ ^1A_u$	2.42	2.58	2.73	2.79	2.85	2.97	3.09

Table S15: ... continued

**VDZP Basis Set**		**Excitation Energy V^{calc} [eV]**						
**Molecule**	**State**	**0**	**0.10**	**0.20**	**0.25**	**0.30**	**0.40**	**0.50**
Benzoquinone	$1\ ^1B_{3g}$	3.65	3.90	4.08	4.15	4.23	4.36	4.47
Benzoquinone	$1\ ^1B_{1u}$	4.64	4.85	5.02	5.09	5.16	5.30	5.42
Benzoquinone	$2\ ^1B_{1u}$	6.11	6.52	6.86	7.01	7.16	7.43	7.68
Benzoquinone	$1\ ^3B_{1g}$	2.19	2.40	2.57	2.64	2.71	2.84	2.96
Benzoquinone	$1\ ^3A_u$	2.27	2.46	2.61	2.68	2.75	2.87	2.99
Formamide	$1\ ^1A''$	5.80	5.83	5.86	5.88	5.89	5.92	5.95
Formamide	$2\ ^1A'$	7.02	7.21	7.36	7.43	7.50	7.62	7.73
Formamide	$1\ ^3A''$	5.53	5.59	5.64	5.67	5.69	5.74	5.78
Acetamide	$1\ ^1A''$	5.79	5.82	5.86	5.88	5.90	5.93	5.97
Acetamide	$2\ ^1A'$	6.80	6.99	7.14	7.22	7.28	7.41	7.52
Acetamide	$3\ ^1A'$	9.53	9.74	9.92	10.00	10.08	10.22	10.36
Propanamide	$1\ ^1A''$	5.81	5.85	5.89	5.91	5.92	5.96	6.00
Propanamide	$2\ ^1A'$	6.73	6.92	7.09	7.16	7.23	7.36	7.48
Propanamide	$3\ ^1A'$	9.34	9.57	9.76	9.85	9.93	10.09	10.23
Cytosine	$2\ ^1A'$	4.40	4.55	4.68	4.73	4.79	4.90	5.00
Cytosine	$1\ ^1A''$	5.09	5.26	5.39	5.45	5.51	5.63	5.73
Cytosine	$3\ ^1A'$	5.13	5.36	5.54	5.63	5.71	5.85	5.99
Cytosine	$4\ ^1A'$	5.83	6.14	6.36	6.46	6.56	6.74	6.91
Cytosine	$5\ ^1A'$	6.49	6.78	7.00	7.10	7.20	7.37	7.54
Thymine	$1\ ^1A''$	5.03	5.14	5.23	5.27	5.31	5.39	5.46
Thymine	$2\ ^1A'$	4.64	4.84	5.00	5.07	5.14	5.28	5.40
Thymine	$3\ ^1A'$	5.64	5.89	6.08	6.17	6.25	6.40	6.54
Thymine	$4\ ^1A'$	5.95	6.22	6.42	6.51	6.60	6.76	6.91
Thymine	$5\ ^1A'$	6.77	7.09	7.31	7.42	7.51	7.69	7.86
Uracil	$1\ ^1A''$	5.00	5.12	5.21	5.25	5.30	5.38	5.45
Uracil	$2\ ^1A'$	4.79	4.99	5.15	5.22	5.29	5.43	5.55
Uracil	$3\ ^1A'$	5.73	5.94	6.10	6.18	6.25	6.39	6.52
Uracil	$4\ ^1A'$	6.18	6.45	6.65	6.74	6.83	6.99	7.13
Uracil	$5\ ^1A'$	6.78	7.09	7.31	7.42	7.51	7.69	7.85
Adenine	$2\ ^1A'$	4.80	5.03	5.20	5.27	5.35	5.49	5.62
Adenine	$3\ ^1A'$	4.90	5.13	5.29	5.36	5.44	5.57	5.70
Adenine	$1\ ^1A''$	5.01	5.08	5.31	5.37	5.43	5.55	5.66
Adenine	$4\ ^1A'$	5.78	6.16	6.40	6.51	6.62	6.81	6.99
Adenine	$5\ ^1A'$	6.21	6.55	6.74	6.83	6.91	7.07	7.21
Adenine	$6\ ^1A'$	6.33	6.68	6.91	7.01	7.11	7.29	7.46
Adenine	$7\ ^1A'$	6.93	7.42	7.65	7.76	7.86	8.06	8.24

Table S16: CASPT2 vertical excitation energies for the Thiel benchmark set obtained using the ANO-RCC-VTZP basis set and different IPEA shift parameters (ε = 0, 0.10, 0.20, 0.25, 0.30, 0.40, and 0.50 a.u.).

**VTZP Basis Set**		**Excitation Energy V^{calc} [eV]**						
**Molecule**	**State**	**0**	**0.10**	**0.20**	**0.25**	**0.30**	**0.40**	**0.50**
Ethene	$1\,^1B_{1u}$	8.08	8.18	8.28	8.32	8.36	8.44	8.52
Ethene	$1\,^3B_{1u}$	4.37	4.42	4.46	4.48	4.50	4.54	4.57
Butadiene	$1\,^1B_u$	5.89	6.07	6.23	6.30	6.37	6.50	6.62
Butadiene	$1\,^3B_u$	3.19	3.28	3.37	3.42	3.46	3.53	3.60
Butadiene	$1\,^3A_g$	4.95	5.07	5.18	5.23	5.28	5.38	5.46
Hexatriene	$1\,^1B_u$	4.47	4.91	5.06	5.14	5.21	5.34	5.46
Hexatriene	$2\,^1A_g$	5.05	5.21	5.35	5.41	5.47	5.59	5.69
Hexatriene	$1\,^3B_u$	2.49	2.61	2.70	2.74	2.78	2.85	2.92
Hexatriene	$1\,^3A_g$	4.03	4.17	4.30	4.35	4.41	4.51	4.60
Octatetraene	$2\,^1A_g$	4.28	4.44	4.57	4.63	4.69	4.80	4.90
Octatetraene	$1\,^1B_u$	4.13	4.32	4.48	4.54	4.61	4.74	4.85
Octatetraene	$2\,^1B_u$	5.27	5.51	5.69	5.77	5.85	6.00	6.14
Octatetraene	$1\,^3B_u$	2.12	2.25	2.34	2.38	2.42	2.49	2.56
Octatetraene	$1\,^3A_g$	3.40	3.58	3.70	3.76	3.81	3.91	4.01
Cyclopropene	$1\,^1B_1$	6.35	6.46	6.55	6.59	6.64	6.72	6.79
Cyclopropene	$1\,^1B_2$	6.45	6.59	6.72	6.78	6.84	6.95	7.05
Cyclopropene	$1\,^3B_2$	4.14	4.21	4.26	4.29	4.32	4.36	4.41
Cyclopropene	$1\,^3B_1$	6.06	6.18	6.28	6.33	6.38	6.46	6.54
Cyclopentadiene	$2\,^1A_1$	5.68	5.92	6.14	6.23	6.32	6.48	6.62
Cyclopentadiene	$3\,^1A_1$	7.42	7.91	8.21	8.34	8.47	8.70	8.91
Norbornadiene	$1\,^1A_2$	5.97	6.02	6.12	6.17	6.21	6.25	6.37
Norbornadiene	$1\,^1B_2$	7.12	7.17	7.27	7.32	7.36	7.40	7.52
Norbornadiene	$2\,^1B_2$	7.43	7.49	7.60	7.67	7.71	7.77	7.90
Norbornadiene	$2\,^1A_2$	7.87	7.93	8.03	8.09	8.12	8.17	8.29
Benzene	$2\,^1A'$	4.09	4.43	4.70	4.82	4.94	5.15	5.35
Benzene	$3\,^1A'$	5.86	6.13	6.35	6.45	6.54	6.72	6.88
Benzene	$4\,^1A'$	6.29	6.61	6.88	7.01	7.13	7.35	7.56
Benzene	$5\,^1A'$	6.29	6.62	6.90	7.03	7.15	7.38	7.59
Benzene	$6\,^1A'$	6.88	7.45	7.82	7.97	8.11	8.36	8.58
Benzene	$7\,^1A'$	6.90	7.47	7.85	8.00	8.14	8.39	8.61
Benzene	$1\,^3A'$	3.48	3.74	3.95	4.05	4.14	4.32	4.48
Benzene	$2\,^3A'$	4.24	4.50	4.71	4.81	4.90	5.07	5.23
Benzene	$3\,^3A'$	4.24	4.50	4.72	4.82	4.91	5.09	5.25
Benzene	$4\,^3A'$	5.19	5.42	5.62	5.72	5.81	5.97	6.13
Naphthalene	$1\,^1B_{3u}$	3.88	4.05	4.19	4.26	4.32	4.44	4.56
Naphthalene	$1\,^1B_{2u}$	4.18	4.36	4.51	4.58	4.65	4.78	4.90

Table S16: ... continued

VTZP Basis Set		Excitation Energy V^{calc} [eV]						
**Molecule**	**State**	**0**	**0.10**	**0.20**	**0.25**	**0.30**	**0.40**	**0.50**
Naphthalene	$2\ ^1A_g$	5.29	5.58	5.79	5.88	5.97	6.15	6.31
Naphthalene	$1\ ^1B_{1g}$	5.26	5.59	5.81	5.91	6.00	6.17	6.33
Naphthalene	$2\ ^1B_{3u}$	5.18	5.54	5.82	5.92	5.98	6.26	6.46
Naphthalene	$2\ ^1B_{2u}$	5.69	5.89	6.09	6.17	6.26	6.41	6.55
Naphthalene	$3\ ^1A_g$	5.95	6.29	6.53	6.64	6.74	6.93	7.10
Naphthalene	$3\ ^1B_{2u}$	7.27		7.24	8.01	8.13	8.34	8.53
Furan	$1\ ^1B_2$	5.99	6.21	6.39	6.47	6.54	6.68	6.82
Furan	$3\ ^1A_1$	6.97	7.50	7.87	8.03	8.17	8.44	8.67
Furan	$1\ ^3B_2$	4.16	4.23	4.31	4.35	4.38	4.45	4.51
Furan	$1\ ^3A_1$	5.35	5.49	5.61	5.67	5.72	5.82	5.92
Pyrrole	$1\ ^1B_2$	5.88	6.15	6.37	6.46	6.55	6.72	6.86
Pyrrole	$3\ ^1A_1$	7.28	7.66	7.94	8.06	8.18	8.40	8.60
Pyrrole	$1\ ^3B_2$	4.36	4.45	4.53	4.58	4.62	4.69	4.77
Pyrrole	$1\ ^3A_1$	5.19	5.36	5.51	5.58	5.64	5.77	5.88
Imidazole	$2\ ^1A'$	5.32	5.70	5.97	6.10	6.17	6.36	6.52
Imidazole	$3\ ^1A'$	6.40	6.57	6.77	6.86	6.94	7.09	7.23
Pyridine	$2\ ^1A_1$	5.40		6.09	6.22	6.33	6.53	6.72
Pyridine	$3\ ^1A_1$	6.48		7.17	7.30	7.43	7.66	7.86
Pyridine	$1\ ^1B_1$	4.88		5.08	5.13	5.50	5.59	5.67
Pyridine	$1\ ^1A_2$	5.18		5.40	5.45	5.15	5.23	5.30
Pyridine	$1\ ^3A_1$	4.00		4.27	4.33	4.38	4.49	4.58
Pyridine	$1\ ^3B_2$	3.99		4.52	4.60	4.68	4.83	4.96
Pyrazine	$1\ ^1B_{2u}$	4.38	4.59	4.76	4.83	4.90	5.04	5.16
Pyrazine	$1\ ^1B_{1u}$	6.22	6.42	6.60	6.68	6.76	6.91	7.05
Pyrazine	$2\ ^1B_{1u}$	6.92	7.24	7.50	7.62	7.73	7.93	8.12
Pyrazine	$2\ ^1B_{2u}$	6.89	7.18	7.43	7.55	7.66	7.87	8.05
Pyrazine	$1\ ^1B_{3u}$	3.68	3.83	3.97	4.03	4.09	4.21	4.32
Pyrazine	$1\ ^1B_{2g}$	5.26	5.42	5.55	5.61	5.67	5.78	5.89
Pyrazine	$1\ ^1B_{1g}$	5.44	5.97	6.20	6.31	6.41	6.59	6.76
Pyrimidine	$1\ ^1B_2$	0.00	5.10	5.26	5.33	5.40	5.54	5.66
Pyrimidine	$2\ ^1A_1$	0.00	6.18	6.37	6.46	6.54	6.70	6.84
Pyrimidine	$3\ ^1A_1$	0.00	6.62	6.91	7.03	7.15	7.37	7.57
Pyrimidine	$2\ ^1B_2$	0.00	6.92	7.25	7.40	7.54	7.80	8.03
Pyrimidine	$1\ ^1B_1$	0.00	4.26	4.38	4.44	4.49	4.59	4.69
Pyrimidine	$1\ ^1A_2$	0.00	4.59	4.72	4.78	4.85	4.96	5.07
Pyridazine	$2\ ^1A_1$	4.79	4.96	5.11	5.18	5.24	5.37	5.48
Pyridazine	$1\ ^1B_2$	5.44	5.80	6.04	6.14	6.25	6.43	6.60
Pyridazine	$2\ ^1B_2$	6.38	6.79	7.09	7.22	7.34	7.57	7.77

Table S16: ... continued

**VTZP Basis Set**		**Excitation Energy** $\mathbf{V}^{calc}$ **[eV]**						
**Molecule**	**State**	**0**	**0.10**	**0.20**	**0.25**	**0.30**	**0.40**	**0.50**
Pyridazine	$3\ ^1A_1$	6.64	7.03	7.33	7.46	7.59	7.81	8.02
Pyridazine	$1\ ^1B_1$	3.43	3.59	3.71	3.77	3.83	3.94	4.05
Pyridazine	$2\ ^1A_2$	5.38	5.56	5.72	5.80	5.87	6.00	6.13
Pyridazine	$2\ ^1B_1$	6.02	6.26	6.44	6.52	6.60	6.75	6.88
Triazine	$2\ ^1A'$	5.45	5.60	5.73	5.79	5.85	5.97	6.08
Triazine	$3\ ^1A'$	6.45	6.81	7.02	7.11	7.19	7.35	7.50
Triazine	$4\ ^1A'$	6.48	6.89	7.19	7.33	7.46	7.69	7.91
Triazine	$2\ ^1A''$	4.11	4.45	4.65	4.74	4.80	4.99	5.14
Triazine	$3\ ^1A''$	4.25	4.47	4.68	4.77	4.85	5.03	5.19
Triazine	$4\ ^1A''$	4.32	4.49	4.69	4.78	4.86	5.04	5.19
Tetrazine	$1\ ^1B_{2u}$	4.43	4.64	4.81	4.89	4.96	5.09	5.22
Tetrazine	$1\ ^1B_{1u}$	6.06	6.35	6.59	6.70	6.80	6.99	7.16
Tetrazine	$2\ ^1B_{1u}$	6.61	6.89	7.12	7.23	7.33	7.53	7.71
Tetrazine	$2\ ^1B_{2u}$	7.02	7.43	7.69	7.81	7.92	8.12	8.31
Tetrazine	$1\ ^1B_{3u}$	1.85	2.01	2.14	2.20	2.26	2.38	2.48
Tetrazine	$1\ ^1A_u$	2.94	3.18	3.36	3.44	3.52	3.67	3.80
Tetrazine	$2\ ^1B_{2g}$	5.35	5.07	5.87	5.98	6.09	6.28	6.47
Tetrazine	$2\ ^1B_{1g}$	5.65	5.96	6.23	6.36	6.47	6.69	6.89
Tetrazine	$2\ ^1B_{3u}$	6.09	6.37	6.59	6.70	6.80	6.98	7.15
Tetrazine	$1\ ^3B_{3u}$	1.18	1.35	1.49	1.56	1.63	1.75	1.86
Tetrazine	$1\ ^3A_u$	2.69	2.94	3.14	3.23	3.32	3.48	3.63
Tetrazine	$1\ ^3B_{1g}$	3.64	3.86	4.02	4.10	4.17	4.31	4.43
Formaldehyde	$1\ ^1A_2$	4.09	4.11	4.13	4.14	4.15	3.86	4.19
Formaldehyde	$1\ ^3A_2$	3.65	3.69	3.73	3.75	3.77	3.49	3.83
Formaldehyde	$1\ ^3A_1$	6.03	6.03	6.04	6.04	6.05	5.75	6.07
Acetone	$1\ ^1A_2$	4.50	4.59	4.66	4.69	4.73	4.79	4.85
Acetone	$1\ ^3A_2$	4.15	4.25	4.34	4.38	4.42	4.50	4.56
Acetone	$1\ ^3A_1$	6.17	6.24	6.30	6.32	6.35	6.40	6.45
Benzoquinone	$1\ ^1B_{1g}$	2.29	2.47	2.63	2.70	2.77	2.90	3.02
Benzoquinone	$1\ ^1A_u$	2.32	2.50	2.65	2.72	2.78	2.91	3.03
Benzoquinone	$1\ ^1B_{3g}$	3.50	3.76	3.94	4.02	4.10	4.24	4.36
Benzoquinone	$1\ ^1B_{1u}$	4.48	4.69	4.86	4.95	5.02	5.16	5.29
Benzoquinone	$2\ ^1B_{1u}$	5.85	6.25	6.60	6.76	6.15	6.35	6.52
Benzoquinone	$1\ ^3B_{1g}$	2.12	2.33	2.50	2.58	2.65	2.79	2.92
Benzoquinone	$1\ ^3A_u$	2.20	2.38	2.55	2.62	2.69	2.82	2.94
Formamide	$1\ ^1A''$	5.60	5.64	5.68	5.69	5.71	5.75	5.78
Formamide	$2\ ^1A'$	6.58	6.85	7.03	7.11	7.18	7.32	7.44
Formamide	$1\ ^3A''$	5.34	5.40	5.45	5.47	5.49	5.53	5.57

Table S16: ... continued

VTZP Basis Set		Excitation Energy V^{calc} [eV]						
**Molecule**	**State**	**0**	**0.10**	**0.20**	**0.25**	**0.30**	**0.40**	**0.50**
Acetamide	$1\,^1A''$	5.64	5.67	5.71	5.73	5.75	5.80	5.84
Acetamide	$2\,^1A'$		6.48	6.77	6.86	6.95	7.11	7.24
Acetamide	$3\,^1A'$	9.22	9.18	9.55	9.66	9.76	9.93	10.09
Propanamide	$1\,^1A''$	5.58	5.66	5.74	5.77	5.81	5.88	5.95
Propanamide	$2\,^1A'$	7.27	7.45	7.61	7.68	7.75	7.87	7.99
Propanamide	$3\,^1A'$	9.42	9.59	9.75	9.82	9.88	10.00	10.12
Cytosine	$2\,^1A'$	4.25	4.41	4.55	4.61	4.67	4.79	4.90
Cytosine	$1\,^1A''$	4.95	5.11	5.25	5.31	5.37	5.49	5.61
Cytosine	$3\,^1A'$	4.87	5.14	5.35	5.44	5.53	5.70	5.86
Cytosine	$4\,^1A'$	5.36	5.73	6.03	6.15	6.27	6.48	6.67
Cytosine	$5\,^1A'$	6.15	6.47	6.73	6.84	6.95	7.15	7.33
Thymine	$1\,^1A''$	4.89	5.01	5.11	5.15	5.19	5.28	5.36
Thymine	$2\,^1A'$	4.39	4.61	4.78	4.89	4.98	5.08	5.23
Thymine	$3\,^1A'$	5.48	5.52	5.74	6.04	6.11	6.21	6.38
Thymine	$4\,^1A'$		5.72	5.94	6.17			
Thymine	$5\,^1A'$		6.43	6.74	6.79			
Uracil	$1\,^1A''$	4.78	4.98	5.08	5.13	5.18	5.26	5.35
Uracil	$2\,^1A'$	4.60	4.82	4.99	5.07	5.14	5.29	5.42
Uracil	$3\,^1A'$	5.53	5.76	5.94	6.02	6.10	6.24	6.38
Uracil	$4\,^1A'$	5.88	6.18	6.40	6.50	6.60	6.78	6.94
Uracil	$5\,^1A'$	6.48	6.83	7.08	7.20	7.30	7.50	7.68
Adenine	$2\,^1A'$	4.56	4.85	5.03	5.11	5.19	5.34	5.48
Adenine	$3\,^1A'$	4.68	4.93	5.13	5.22	5.30	5.45	5.60
Adenine	$1\,^1A''$	4.46	4.93	5.17	5.24	5.30	5.43	5.54
Adenine	$4\,^1A'$	5.30	5.76	6.06	6.19	6.31	6.53	6.73
Adenine	$5\,^1A'$	5.87	6.26	6.51	6.62	6.71	6.89	7.06
Adenine	$6\,^1A'$		6.37	6.66	6.79	6.90	7.10	7.29
Adenine	$7\,^1A'$		6.99	7.33	7.45	7.57	7.79	7.99

Table S17: CASPT2 vertical excitation energies and approximate dynamical correlation energies $\widetilde{E}^{\text{dyn}}$ for the Thiel benchmark set obtained using the ANO-RCC-VQZP basis set and different IPEA shift parameters (ε = 0, 0.10, 0.20, 0.25, 0.30, 0.40, and 0.50 a.u.).

VQZP Basis Set		Excitation Energy V^{calc} [eV]						
**Molecule**	**State**	**0**	**0.10**	**0.20**	**0.25**	**0.30**	**0.40**	**0.50**
Ethene	$1\,^1B_{1u}$	8.09	8.18	8.26	8.30	8.34	8.40	8.47
Ethene	$1\,^3B_{1u}$	4.36	4.41	4.46	4.48	4.50	4.53	4.57
Butadiene	$1\,^1B_u$	5.79	5.97	6.13	6.20	6.27	6.40	6.52

Table S17: . . . continued

**VQZP Basis Set**		**Excitation Energy V^{calc} [eV]**						
**Molecule**	**State**	**0**	**0.10**	**0.20**	**0.25**	**0.30**	**0.40**	**0.50**
Butadiene	$1\,^3B_u$	3.21	3.30	3.39	3.44	3.48	3.55	3.62
Butadiene	$1\,^3A_g$	4.95	5.07	5.19	5.24	5.29	5.39	5.47
Hexatriene	$1\,^1B_u$	4.60	4.75	5.02	4.77	4.97	5.31	5.40
Hexatriene	$2\,^1A_g$	4.87	5.17	5.32	5.39	5.45	5.57	5.68
Hexatriene	$1\,^3B_u$	2.48	2.61	2.70	2.74	2.78	2.86	2.93
Hexatriene	$1\,^3A_g$	4.00	4.16	4.29	4.35	4.41	4.51	4.61
Octatetraene	$2\,^1A_g$	4.26	4.43	4.56	4.62	4.68	4.80	4.90
Octatetraene	$1\,^1B_u$	4.07	4.27	4.42	4.50	4.56	4.69	4.80
Octatetraene	$2\,^1B_u$	5.13	5.43	5.63	5.72	5.81	5.97	6.11
Octatetraene	$1\,^3B_u$	2.11	2.25	2.34	2.38	2.42	2.50	2.57
Octatetraene	$1\,^3A_g$	3.40	3.58	3.70	3.76	3.82	3.92	4.01
Cyclopropene	$1\,^1B_1$	6.29	6.40	6.49	6.54	6.58	6.66	6.74
Cyclopropene	$1\,^1B_2$	6.53	6.66	6.78	6.84	6.89	7.00	7.09
Cyclopropene	$1\,^3B_2$	4.13	4.20	4.25	4.28	4.31	4.36	4.40
Cyclopropene	$1\,^3B_1$	6.01	6.13	6.24	6.29	6.33	6.42	6.50
Cyclopentadiene	$1\,^1B_2$	5.06		5.34	5.41	5.47	5.59	5.71
Cyclopentadiene	$2\,^1A_1$	5.53		6.08	6.17	6.27	6.43	6.58
Cyclopentadiene	$3\,^1A_1$	7.24		7.97	8.11	8.25	8.49	8.71
Cyclopentadiene	$1\,^3B_2$	3.27		3.43	3.48	3.52	3.59	3.66
Norbornadiene	$1\,^1A_2$	6.07	6.10	6.20	6.20	6.27	6.35	6.42
Norbornadiene	$1\,^1B_2$	7.15	7.18	7.27	7.27	7.34	7.41	7.49
Norbornadiene	$2\,^1B_2$	7.44	7.48	7.59	7.60	7.68	7.76	7.85
Norbornadiene	$2\,^1A_2$	7.90	7.94	8.04	8.04	8.12	8.19	8.27
Norbornadiene	$1\,^3A_2$	3.74	3.81	3.93	3.94	4.02	4.11	4.19
Norbornadiene	$1\,^3B_2$	4.23	4.30	4.42	4.68	4.52	4.61	4.71
Benzene	$2\,^1A'$	4.07	4.41	4.68	4.80	4.92	5.14	5.33
Benzene	$3\,^1A'$	5.78	6.05	6.27	6.37	6.47	6.65	6.81
Benzene	$4\,^1A'$	6.17	6.50	6.78	6.91	7.03	7.26	7.47
Benzene	$5\,^1A'$	6.17	6.51	6.79	6.92	7.05	7.28	7.50
Benzene	$6\,^1A'$	6.75	7.39	7.78	7.94	8.09	8.34	8.57
Benzene	$7\,^1A'$	6.76	7.42	7.81	7.97	8.11	8.37	8.59
Benzene	$1\,^3A'$	3.47	3.73	3.95	4.04	4.14	4.31	4.48
Benzene	$2\,^3A'$	4.22	4.48	4.70	4.79	4.89	5.06	5.22
Benzene	$3\,^3A'$	4.22	4.49	4.71	4.81	4.90	5.08	5.24
Benzene	$4\,^3A'$	5.13	5.37	5.58	5.67	5.76	5.93	6.09
Naphthalene	$1\,^1B_{3u}$	3.85	4.03	4.17	4.24	4.31	4.43	4.54
Naphthalene	$1\,^1B_{2u}$	4.12	4.29	4.46	4.53	4.60		4.85
Naphthalene	$2\,^1A_g$	4.23	5.28	5.61	5.74	5.85	6.06	6.24

Table S17: ... continued

**VQZP Basis Set**		**Excitation Energy V^{calc} [eV]**						
**Molecule**	**State**	**0**	**0.10**	**0.20**	**0.25**	**0.30**	**0.40**	**0.50**
Naphthalene	$1\,^1B_{1g}$	5.07	5.46	5.70	5.80	5.90	6.08	6.25
Naphthalene	$2\,^1B_{3u}$	5.01	5.39	5.72	5.85	5.98	6.18	6.39
Naphthalene	$2\,^1B_{2u}$	5.64	5.81	6.00	6.09	6.18	6.33	
Naphthalene	$3\,^1A_g$	5.24	6.06	6.39	6.52	5.58	5.73	5.86
Furan	$1\,^1B_2$	5.69	5.99	6.12	6.23	6.37	6.53	6.67
Furan	$3\,^1A_1$		7.06	7.56	7.75	7.92	8.23	8.49
Furan	$1\,^3B_2$	4.08	4.20	4.32	4.34	4.38	4.44	4.51
Furan	$1\,^3A_1$	5.26	5.44	5.61	5.66	5.72	5.82	5.92
Pyrrole	$1\,^1B_2$	5.42	5.85	6.06	6.22	6.33	6.52	6.70
Pyrrole	$3\,^1A_1$	6.83	7.34	7.70	7.85	7.98	8.23	8.45
Pyrrole	$1\,^3B_2$	4.33	4.43	4.52	4.56	4.60	4.68	4.75
Pyrrole	$1\,^3A_1$	5.15	5.33	5.48	5.55	5.62	5.75	5.86
Pyridine	$2\,^1A_1$		5.76	6.04	6.23	6.24	6.46	6.65
Pyridine	$3\,^1A_1$		6.72	7.03	6.79	7.30	7.54	7.76
Pyridine	$1\,^1B_1$		4.97	5.07	5.11	5.49	5.59	5.67
Pyridine	$1\,^1A_2$		5.29	5.40	5.45	5.14	5.22	5.30
Pyridine	$1\,^3A_1$		4.15	4.27	4.33	4.39	4.49	4.59
Pyridine	$1\,^3B_2$		4.40	4.51	4.56		4.80	4.93
Pyrazine	$1\,^1B_{2u}$	4.16	4.54	4.68	4.77	4.84	4.98	5.11
Pyrazine	$1\,^1B_{1u}$	6.15	6.41	6.54	6.62	6.70	6.86	6.99
Pyrazine	$2\,^1B_{1u}$	6.79	7.16	7.38	7.50	7.61	7.82	8.02
Pyrazine	$2\,^1B_{2u}$	6.75	7.11	7.34	7.46	7.58	7.79	7.99
Pyrazine	$1\,^1B_{3u}$	3.63	3.84	3.94	4.00	4.06	4.18	4.29
Pyrazine	$1\,^1B_{2g}$	5.22	5.43	5.52	5.58	5.64	5.75	5.86
Pyrazine	$1\,^1B_{1g}$	5.61	5.98	6.17	6.28	6.38	6.57	6.74
Pyrimidine	$1\,^1B_2$	4.87	5.05	5.20	5.27	5.34	5.47	5.59
Pyrimidine	$2\,^1A_1$	5.69	6.10	6.30	6.39	6.48	6.63	6.78
Pyrimidine	$3\,^1A_1$	5.97	6.42	6.74	6.88	7.02	7.25	7.47
Pyrimidine	$2\,^1B_2$	6.75	7.07	7.35	7.47	7.59	7.81	8.01
Pyrimidine	$1\,^1B_1$	4.11	4.25	4.37	4.42	4.48	4.58	4.68
Pyrimidine	$1\,^1A_2$	4.43	4.58	4.71	4.78	4.84	4.95	5.06
Pyridazine	$2\,^1A_1$	4.75	4.93	5.08	5.15	5.22	5.35	5.47
Pyridazine	$1\,^1B_2$	5.34	5.68	5.95	6.06	6.16	6.35	6.52
Pyridazine	$2\,^1B_2$	6.26	6.64	6.99	7.13	7.26	7.49	7.70
Pyridazine	$3\,^1A_1$	6.64	6.91	7.03	7.36	7.49	7.73	7.94
Pyridazine	$1\,^1B_1$	3.41	3.56	3.70	3.76	3.82	3.93	4.03
Pyridazine	$2\,^1A_2$	5.35	5.54	5.71	5.78	5.85	5.99	6.12
Pyridazine	$2\,^1B_1$	5.95	6.24	6.42	6.51	6.59	6.74	6.88

Table S17: . . . continued

VQZP Basis Set		Excitation Energy V^{calc} [eV]						
**Molecule**	**State**	**0**	**0.10**	**0.20**	**0.25**	**0.30**	**0.40**	**0.50**
Triazine	$2\ ^1A'$	5.72	5.79	5.86	5.91	5.96	6.06	6.16
Triazine	$3\ ^1A'$		6.96	7.12				
Triazine	$2\ ^1A''$		4.63	4.77	4.85	4.92	5.07	5.22
Triazine	$3\ ^1A''$		4.64	4.80	4.88	4.96	5.12	5.27
Triazine	$4\ ^1A''$		4.68	4.82	4.90	4.98	5.13	5.28
Tetrazine	$1\ ^1B_{2u}$	4.37	4.60	4.77	4.85	4.93	5.06	5.19
Tetrazine	$1\ ^1B_{1u}$	5.98	6.28	6.52	6.63	6.73	6.93	7.10
Tetrazine	$2\ ^1B_{1u}$	6.55	6.83	7.06	7.17	7.27	7.47	7.65
Tetrazine	$2\ ^1B_{2u}$	6.98	7.36	7.63	7.75	7.86	8.07	8.26
Tetrazine	$1\ ^1B_{3u}$	1.82	1.99	2.12	2.18	2.24	2.36	2.46
Tetrazine	$1\ ^1A_u$	2.91	3.16	3.34	3.43	3.51	3.65	3.79
Tetrazine	$2\ ^1B_{2g}$	5.31	5.61	5.85	5.96	6.07	6.27	6.45
Tetrazine	$2\ ^1B_{1g}$	5.58	5.91	6.19	6.31	6.43	6.66	6.86
Tetrazine	$2\ ^1B_{3u}$	6.05	6.26	6.57	6.68	6.78	6.96	7.14
Tetrazine	$1\ ^3B_{3u}$	1.16	1.34	1.48	1.55	1.61	1.74	1.85
Tetrazine	$1\ ^3A_u$	2.68	2.94	3.14	3.23	3.31	3.48	3.63
Tetrazine	$1\ ^3B_{1g}$	3.62	3.84	4.00	4.08	4.15	4.29	4.42
Formaldehyde	$1\ ^1A_2$	4.13	4.14	4.16	4.17	3.20	3.22	3.24
Formaldehyde	$1\ ^3A_2$	3.69	3.73	3.77	3.79	2.83	2.86	2.90
Formaldehyde	$1\ ^3A_1$	6.08	6.08	6.09	6.09	5.12	5.13	5.14
Acetone	$1\ ^1A_2$	4.53	4.62	4.70	4.73	4.76	4.83	4.89
Acetone	$1\ ^3A_2$	4.18	4.29	4.38	4.42	4.46	4.54	4.61
Acetone	$1\ ^3A_1$	6.21	6.28	6.34	6.37	6.40	6.45	6.50
Benzoquinone	$1\ ^1B_{1g}$	2.29	2.47	2.63	2.71	2.78	2.91	3.03
Benzoquinone	$1\ ^1A_u$	2.34	2.50	2.65	2.72	2.79	2.91	3.04
Benzoquinone	$1\ ^1B_{3g}$	3.46	3.72	3.91	3.99	4.07	4.21	4.33
Benzoquinone	$1\ ^1B_{1u}$	4.58	4.81	4.97	5.05	5.13	5.27	5.40
Benzoquinone	$1\ ^3B_{1g}$	2.13	2.34	2.51	2.59	2.66	2.80	2.93
Benzoquinone	$1\ ^3A_u$	2.21	2.39	2.55	2.63	2.70	2.83	2.95
Formamide	$1\ ^1A''$	5.59	5.63	5.67	5.69	5.70	5.74	5.78
Formamide	$2\ ^1A'$	6.49	6.77	6.96	7.04	7.12	7.26	7.39
Formamide	$1\ ^3A''$	5.33	5.39	5.44	5.46	5.49	5.53	5.56
Acetamide	$1\ ^1A''$	5.61		5.71	5.73	5.75	5.80	5.84
Acetamide	$2\ ^1A'$	7.23		6.70	6.80	6.90	7.06	7.20
Acetamide	$3\ ^1A'$	9.01		9.50	9.62	9.72	9.90	10.06
Propanamide	$1\ ^1A''$	5.60	5.68	5.76	5.80	5.84	5.91	5.98
Propanamide	$2\ ^1A'$	7.36	7.53	7.68	7.75	7.82	7.94	8.06
Propanamide	$3\ ^1A'$	9.50	9.67	9.82	9.89	9.95	10.07	10.18

Table S17: ... continued

VQZP Basis Set		Excitation Energy $\mathbf{V}^{\text{calc}}$ [eV]						
**Molecule**	**State**	**0**	**0.10**	**0.20**	**0.25**	**0.30**	**0.40**	**0.50**
Cytosine	$2\ ^1A'$	4.36		4.51	4.58	4.65	4.76	4.88
Cytosine	$1\ ^1A''$	4.94		5.24	5.39	5.37	5.49	5.61
Cytosine	$3\ ^1A'$	4.84		5.27	5.38	5.47	5.65	5.81
Cytosine	$4\ ^1A'$	5.51		5.77	5.94	6.08	6.29	6.54
Cytosine	$5\ ^1A'$	6.01		6.56	6.69	6.81	7.02	7.23
Thymine	$1\ ^1A''$	4.89	5.01	5.11	5.16	5.20	5.29	5.37
Thymine	$2\ ^1A'$	4.19	4.58	4.79	4.91	4.86	5.04	5.19
Thymine	$3\ ^1A'$	5.26	5.33	5.87	5.99	5.97	6.19	6.36
Thymine	$4\ ^1A'$	5.38	5.87	6.19	6.28	6.27	6.45	6.66
Thymine	$5\ ^1A'$	6.20		6.97	7.08	7.09	7.25	7.61
Uracil	$1\ ^1A''$	4.84	4.98	5.08	5.14	5.18	5.27	5.36
Uracil	$2\ ^1A'$	4.55	4.77	4.95	5.03	5.11	5.26	5.40
Uracil	$3\ ^1A'$	5.46	5.72	5.91	6.00	6.08	6.23	6.37
Uracil	$4\ ^1A'$	5.73	6.05	6.29	6.40	6.50	6.69	6.87
Uracil	$5\ ^1A'$	6.33	6.72	7.00	7.12	7.24	7.45	7.64
Adenine	$2\ ^1A'$			4.95	5.04	5.13	5.28	
Adenine	$3\ ^1A'$			5.06	5.16	5.24	5.41	

Table S18: CASPT2 approximate dynamical correlation energies $\widetilde{E}^{\text{dyn}}$ for the Thiel benchmark set obtained using the ANO-RCC-MB basis set and different IPEA shift parameters (ε = 0, 0.10, 0.20, 0.25, 0.30, 0.40, and 0.50 a.u.).

MB Basis Set		Dynamical Correlation $\widetilde{\mathbf{E}}^{\text{dyn}}$ [eV]						
**Molecule**	**State**	**0**	**0.10**	**0.20**	**0.25**	**0.30**	**0.40**	**0.50**
Ethene	$1\ ^1B_{1u}$	3.64	3.56	3.49	3.45	3.42	3.35	3.29
Ethene	$1\ ^3B_{1u}$	1.80	1.77	1.76	1.75	1.74	1.73	1.71
Butadiene	$1\ ^1B_u$	5.81	5.69	5.59	5.55	5.51	5.43	5.36
Butadiene	$1\ ^3B_u$	4.11	4.06	4.02	4.01	3.99	3.96	3.93
Butadiene	$1\ ^3A_g$	3.93	3.89	3.85	3.83	3.82	3.78	3.76
Hexatriene	$1\ ^1B_u$	6.30	6.18	6.10	6.06	6.03	5.97	5.92
Hexatriene	$2\ ^1A_g$	6.33	6.24	6.19	6.16	6.13	6.09	6.04
Hexatriene	$1\ ^3B_u$	6.34	6.26	6.22	6.20	6.18	6.15	6.11
Hexatriene	$1\ ^3A_g$	6.19	6.12	6.07	6.05	6.02	5.98	5.95
Octatetraene	$2\ ^1A_g$	8.49	8.39	8.32	8.29	8.27	8.21	8.16
Octatetraene	$1\ ^1B_u$	8.42	8.29	8.22	8.18	8.15	8.09	8.03
Octatetraene	$2\ ^1B_u$	9.95	9.81	9.71	9.67	9.62	9.54	9.47
Octatetraene	$1\ ^3B_u$	8.47	8.38	8.33	8.31	8.29	8.25	8.21
Octatetraene	$1\ ^3A_g$	8.36	8.26	8.20	8.17	8.15	8.10	8.06

Table S18: ... continued

**MB Basis Set**		**Dynamical Correlation $\widetilde{\mathbf{E}}^{\mathrm{dyn}}$ [eV]**						
**Molecule**	**State**	**0**	**0.10**	**0.20**	**0.25**	**0.30**	**0.40**	**0.50**
Cyclopropene	$1\,^1B_1$	3.53	3.47	3.42	3.39	3.37	3.32	3.28
Cyclopropene	$1\,^1B_2$	4.57	4.48	4.39	4.35	4.31	4.24	4.17
Cyclopropene	$1\,^3B_2$	3.17	3.14	3.12	3.10	3.09	3.07	3.05
Cyclopropene	$1\,^3B_1$	3.38	3.32	3.27	3.24	3.22	3.17	3.13
Cyclopentadiene	$1\,^1B_2$	7.56	7.42	7.31	7.25	7.20	7.11	7.02
Cyclopentadiene	$2\,^1A_1$	5.77	5.64	5.55	5.51	5.48	5.41	5.36
Cyclopentadiene	$3\,^1A_1$	7.80	7.57	7.40	7.33	7.26	7.13	7.02
Cyclopentadiene	$1\,^3B_2$	5.43	5.37	5.32	5.30	5.28	5.24	5.21
Norbornadiene	$1\,^1A_2$	10.67	10.52	10.38	10.31	10.25	10.14	10.03
Norbornadiene	$1\,^1B_2$	10.98	10.80	10.64	10.57	10.50	10.36	10.24
Norbornadiene	$2\,^1B_2$	11.40	11.13	10.93	10.84	10.76	10.62	10.49
Norbornadiene	$2\,^1A_2$	10.83	10.65	10.49	10.42	10.35	10.22	10.10
Norbornadiene	$1\,^3A_2$	8.19	8.11	8.05	8.03	8.00	7.96	7.91
Norbornadiene	$1\,^3B_2$	8.15	8.08	8.02	7.99	7.97	7.92	7.87
Benzene	$2\,^1A'$	6.24	6.16	6.10	6.07	6.04	5.99	5.94
Benzene	$3\,^1A'$	7.68	7.52	7.40	7.35	7.29	7.20	7.11
Benzene	$4\,^1A'$	6.05	5.94	5.87	5.84	5.81	5.75	5.70
Benzene	$5\,^1A'$	6.05	5.93	5.85	5.81	5.78	5.72	5.66
Benzene	$6\,^1A'$	8.08	7.89	7.73	7.66	7.59	7.47	7.36
Benzene	$7\,^1A'$	8.08	7.88	7.73	7.66	7.59	7.47	7.36
Benzene	$1\,^3A'$	6.07	6.01	5.96	5.94	5.92	5.88	5.84
Benzene	$2\,^3A'$	6.33	6.22	6.15	6.12	6.09	6.04	5.99
Benzene	$3\,^3A'$	6.33	6.21	6.14	6.11	6.08	6.03	5.98
Benzene	$4\,^3A'$	7.53	7.39	7.27	7.22	7.17	7.08	7.00
Naphthalene	$1\,^1B_{3u}$	10.63	10.51	10.43	10.40	10.36	10.30	10.24
Naphthalene	$1\,^1B_{2u}$	12.01	11.86	11.75	11.70	11.65	11.56	11.48
Naphthalene	$2\,^1A_g$	10.47	10.33	10.24	10.20	10.16	10.08	10.01
Naphthalene	$1\,^1B_{1g}$	10.85	10.63	10.53	10.48	10.43	10.35	10.27
Naphthalene	$2\,^1B_{3u}$	12.17	11.95	11.79	11.72	11.65	11.53	11.42
Naphthalene	$2\,^1B_{2u}$	12.00	11.84	11.71	11.66	11.60	11.51	11.42
Naphthalene	$3\,^1A_g$	10.60	10.40	10.29	10.24	10.20	10.11	10.03
Naphthalene	$3\,^1B_{2u}$	10.29	10.11	9.99	9.94	9.90	9.81	9.73
Furan	$1\,^1B_2$	6.51	6.34	6.22	6.17	6.13	6.04	5.96
Furan	$3\,^1A_1$	6.84	6.56	6.38	6.31	6.23	6.11	5.99
Furan	$1\,^3B_2$	5.07	5.02	4.97	4.95	4.93	4.90	4.86
Furan	$1\,^3A_1$	5.20	5.11	5.05	5.03	5.00	4.95	4.91
Pyrrole	$1\,^1B_2$	6.66	6.50	6.38	6.33	6.28	6.20	6.12
Pyrrole	$3\,^1A_1$	6.97	6.73	6.57	6.50	6.44	6.32	6.22

Table S18: . . . continued

MB Basis Set		Dynamical Correlation $\widetilde{\mathbf{E}}^{\rm dyn}$ [eV]						
**Molecule**	**State**	**0**	**0.10**	**0.20**	**0.25**	**0.30**	**0.40**	**0.50**
Pyrrole	$1\,^3B_2$	5.43	5.37	5.32	5.30	5.28	5.24	5.21
Pyrrole	$1\,^3A_1$	5.65	5.56	5.49	5.46	5.43	5.38	5.33
Imidazole	$2\,^1A'$	6.07	5.96	5.86	5.81	5.77	5.69	5.62
Imidazole	$3\,^1A'$	6.76	6.62	6.51	6.46	6.42	6.34	6.26
Pyridine	$2\,^1A_1$	7.89	7.70	7.57	7.51	7.46	7.36	7.27
Pyridine	$3\,^1A_1$	6.05	5.91	5.81	5.76	5.72	5.65	5.59
Pyridine	$1\,^1B_1$	6.42	6.34	6.28	6.26	6.24	6.19	6.15
Pyridine	$1\,^1A_2$	6.66	6.57	6.51	6.48	6.46	6.41	6.36
Pyridine	$1\,^3A_1$	6.08	6.01	5.96	5.94	5.91	5.87	5.83
Pyridine	$1\,^3B_2$	6.38	6.25	6.17	6.14	6.11	6.05	6.00
Pyrazine	$1\,^1B_{2u}$	6.26	6.18	6.11	6.08	6.05	5.99	5.94
Pyrazine	$1\,^1B_{1u}$	7.77	7.62	7.50	7.45	7.40	7.30	7.22
Pyrazine	$2\,^1B_{1u}$	8.65	8.43	8.26	8.18	8.11	7.98	7.86
Pyrazine	$2\,^1B_{2u}$	6.74	6.60	6.50	6.45	6.41	6.33	6.25
Pyrazine	$1\,^1B_{3u}$	6.74	6.64	6.57	6.53	6.50	6.45	6.39
Pyrazine	$1\,^1B_{2g}$	6.26	6.16	6.10	6.08	6.05	6.00	5.95
Pyrazine	$1\,^1B_{1g}$	6.89	6.72	6.62	6.58	6.54	6.46	6.39
Pyrimidine	$1\,^1B_2$	6.21	6.12	6.05	6.02	5.99	5.93	5.88
Pyrimidine	$2\,^1A_1$	7.96	7.76	7.63	7.57	7.52	7.42	7.34
Pyrimidine	$3\,^1A_1$	6.96	6.79	6.67	6.62	6.57	6.47	6.39
Pyrimidine	$2\,^1B_2$	6.56	6.41	6.31	6.27	6.23	6.15	6.08
Pyrimidine	$1\,^1B_1$	6.65	6.56	6.49	6.46	6.43	6.37	6.32
Pyrimidine	$1\,^1A_2$	6.88	6.78	6.70	6.66	6.63	6.57	6.51
Pyridazine	$2\,^1A_1$	6.20	6.11	6.04	6.01	5.98	5.92	5.87
Pyridazine	$1\,^1B_2$	8.31	7.97	7.78	7.70	7.63	7.50	7.40
Pyridazine	$2\,^1B_2$	6.09	5.88	5.76	5.71	5.66	5.58	5.50
Pyridazine	$3\,^1A_1$	6.30	6.22	6.17	6.15	6.12	6.08	6.04
Pyridazine	$1\,^1B_1$	6.77	6.67	6.60	6.57	6.54	6.49	6.44
Pyridazine	$2\,^1A_2$	6.69	6.55	6.45	6.41	8.04	7.98	7.93
Pyridazine	$2\,^1B_1$	6.83	6.66	6.56	6.52	6.47	6.40	6.33
Triazine	$2\,^1A'$	6.22	6.12	6.06	6.02	5.99	5.93	5.88
Triazine	$3\,^1A'$	7.08	6.90	6.77	6.72	6.67	6.58	6.49
Triazine	$4\,^1A'$	6.74	6.54	6.41	6.36	6.30	6.20	6.12
Triazine	$2\,^1A''$	7.08	6.92	6.80	6.76	6.71	6.63	6.55
Triazine	$3\,^1A''$	7.06	6.91	6.80	6.75	6.71	6.63	6.55
Triazine	$4\,^1A''$	7.61	7.40	7.26	7.20	7.15	7.04	6.95
Tetrazine	$1\,^1B_{2u}$	6.16	6.06	5.99	5.96	5.93	5.87	5.81
Tetrazine	$1\,^1B_{1u}$	7.03	6.84	6.71	6.65	6.60	6.50	6.42

Table S18: ... continued

**MB Basis Set**		**Dynamical Correlation $\widetilde{\mathbf{E}}^{\text{dyn}}$ [eV]**						
**Molecule**	**State**	**0**	**0.10**	**0.20**	**0.25**	**0.30**	**0.40**	**0.50**
Tetrazine	$2\,^1B_{1u}$	8.06	7.75	7.55	7.47	7.39	7.25	7.12
Tetrazine	$2\,^1B_{2u}$	6.55	6.36	6.24	6.19	6.14	6.05	5.97
Tetrazine	$1\,^1B_{3u}$	7.02	6.91	6.84	6.80	6.77	6.70	6.65
Tetrazine	$1\,^1A_u$	7.29	7.16	7.06	7.02	6.98	6.91	6.84
Tetrazine	$2\,^1B_{2g}$	6.93	6.74	6.62	6.57	6.52	6.42	6.34
Tetrazine	$2\,^1B_{1g}$	6.79	6.61	6.49	6.43	6.38	6.28	6.19
Tetrazine	$2\,^1B_{3u}$	7.10	6.94	6.82	6.77	6.72	6.62	6.54
Tetrazine	$1\,^3B_{3u}$	8.77	8.66	8.58	8.54	8.51	8.44	8.38
Tetrazine	$1\,^3A_u$	9.38	9.23	9.13	9.09	9.05	8.97	8.89
Tetrazine	$1\,^3B_{1g}$	6.91	6.78	6.70	6.66	6.63	6.56	6.50
Formaldehyde	$1\,^1A_2$	1.58	1.56	1.53	1.52	1.51	1.49	1.47
Formaldehyde	$1\,^3A_2$	1.59	1.56	1.53	1.52	1.51	1.48	1.46
Formaldehyde	$1\,^3A_1$	1.17	1.16	1.15	1.14	1.14	1.13	1.12
Acetone	$1\,^1A_2$	3.86	3.83	3.81	3.79	3.78	3.76	3.74
Acetone	$1\,^3A_2$	3.87	3.84	3.81	3.79	3.78	3.75	3.73
Acetone	$1\,^3A_1$	3.52	3.50	3.48	3.47	3.46	3.45	3.43
Benzoquinone	$1\,^1B_{1g}$	7.71	7.58	7.50	7.47	7.44	7.38	7.32
Benzoquinone	$1\,^1A_u$	7.66	7.54	7.47	7.44	7.41	7.35	7.30
Benzoquinone	$1\,^1B_{3g}$	8.16	7.93	7.80	7.75	7.70	7.61	7.53
Benzoquinone	$1\,^1B_{1u}$	9.48	9.26	9.13	9.07	9.02	8.92	8.83
Benzoquinone	$2\,^1B_{1u}$	10.33	10.05	9.92	9.88	9.84	9.76	9.70
Benzoquinone	$1\,^3B_{1g}$	7.73	7.59	7.50	7.47	7.43	7.37	7.31
Benzoquinone	$1\,^3A_u$	7.66	7.53	7.46	7.43	7.39	7.34	7.28
Formamide	$1\,^1A''$	2.77	2.75	2.72	2.71	2.70	2.68	2.66
Formamide	$2\,^1A'$	4.48	4.36	4.26	4.21	4.17	4.09	4.01
Acetamide	$1\,^1A''$	4.02	3.99	3.97	3.96	3.95	3.93	3.91
Acetamide	$2\,^1A'$	5.76	5.64	5.54	5.49	5.45	5.37	5.29
Acetamide	$3\,^1A'$	5.73	5.62	5.53	5.49	5.45	5.37	5.30
Propanamide	$1\,^1A''$	5.33	5.30	5.28	5.27	5.26	5.24	5.22
Propanamide	$2\,^1A'$	7.11	6.99	6.89	6.84	6.80	6.71	6.63
Propanamide	$3\,^1A'$	7.10	6.99	6.90	6.85	6.81	6.73	6.66
Cytosine	$2\,^1A'$	9.42	9.29	9.21	9.18	9.15	9.08	9.02
Cytosine	$1\,^1A''$	9.16	9.04	8.97	8.93	8.90	8.84	8.78
Cytosine	$3\,^1A'$	9.66	9.46	9.34	9.29	9.24	9.15	9.07
Cytosine	$4\,^1A'$	10.33	10.10	9.96	9.90	9.85	9.75	9.66
Cytosine	$5\,^1A'$	9.83	9.66	9.53	9.47	9.42	9.32	9.23
Thymine	$1\,^1A''$	9.81	9.75	9.69	9.67	9.65	9.60	9.56
Thymine	$2\,^1A'$	10.78	10.62	10.52	10.47	10.43	10.35	10.28

Table S18: ... continued

MB Basis Set		Dynamical Correlation $\widetilde{\mathbf{E}}^{\mathrm{dyn}}$ [eV]						
**Molecule**	**State**	**0**	**0.10**	**0.20**	**0.25**	**0.30**	**0.40**	**0.50**
Thymine	$3\,^1A'$	10.64	10.46	10.35	10.30	10.26	10.18	10.11
Thymine	$4\,^1A'$	11.09	10.86	10.72	10.66	10.60	10.50	10.41
Thymine	$5\,^1A'$	11.37	11.13	10.96	10.89	10.83	10.71	10.61
Uracil	$1\,^1A''$	8.52	8.45	8.39	8.37	8.35	8.30	8.26
Uracil	$2\,^1A'$	9.40	9.25	9.15	9.11	9.06	8.98	8.91
Uracil	$3\,^1A'$	9.29	9.11	9.01	8.96	8.92	8.84	8.77
Uracil	$4\,^1A'$	9.72	9.49	9.35	9.29	9.24	9.13	9.04
Uracil	$5\,^1A'$	8.84	8.65	8.52	8.47	8.42	8.33	8.24
Adenine	$2\,^1A'$	11.94	11.77	11.65	11.60	11.55	11.47	11.39
Adenine	$3\,^1A'$	12.94	12.72	12.58	12.52	12.46	12.36	12.27
Adenine	$1\,^1A''$	12.06	11.93	11.84	11.79			
Adenine	$4\,^1A'$	11.99	11.77	11.63	11.57	11.52	11.42	11.32
Adenine	$5\,^1A'$	12.06	11.84	11.69	11.63	11.58	11.47	11.38
Adenine	$6\,^1A'$	12.49	12.24	12.09	12.02	11.96	11.84	11.73
Adenine	$7\,^1A'$	12.06	11.85	11.71	11.65	11.59	11.48	11.38

Table S19: CASPT2 approximate dynamical correlation energies $\widetilde{E}^{\mathrm{dyn}}$ for the Thiel benchmark set obtained using the ANO-RCC-VDZ basis set and different IPEA shift parameters ($\varepsilon = 0$, 0.10, 0.20, 0.25, 0.30, 0.40, and 0.50 a.u.).

VDZ Basis Set		Dynamical Correlation $\widetilde{\mathbf{E}}^{\mathrm{dyn}}$ [eV]						
**Molecule**	**State**	**0**	**0.10**	**0.20**	**0.25**	**0.30**	**0.40**	**0.50**
Ethene	$1\,^1B_{1u}$	5.57	5.46	5.37	5.32	5.28	5.19	5.11
Ethene	$1\,^3B_{1u}$	3.78	3.74	3.71	3.69	3.67	3.64	3.62
Butadiene	$1\,^1B_u$	10.12	9.95	9.82	9.76	9.70	9.59	9.49
Butadiene	$1\,^3B_u$	8.07	7.99	7.88	7.88	7.85	7.79	7.73
Butadiene	$1\,^3A_g$	7.96	7.87	7.80	7.76	7.72	7.66	7.60
Hexatriene	$1\,^1B_u$	13.35	13.15	13.00	12.93	12.87	12.75	12.64
Hexatriene	$2\,^1A_g$	12.32	12.18	12.07	12.02	11.98	11.89	11.80
Hexatriene	$1\,^3B_u$	12.20	12.07	11.99	11.96	11.92	11.86	11.80
Hexatriene	$1\,^3A_g$	12.12	11.99	11.90	11.85	11.81	11.73	11.65
Octatetraene	$2\,^1A_g$	16.33	16.16	16.05	16.00	15.95	15.85	15.76
Octatetraene	$1\,^3B_u$	16.18	16.03	15.94	15.90	15.86	15.79	15.72
Octatetraene	$1\,^3A_g$	16.14	15.97	15.86	15.81	15.77	15.68	15.59
Cyclopropene	$1\,^1B_1$	6.86	6.76	6.68	6.64	6.60	6.53	6.46
Cyclopropene	$1\,^1B_2$	8.30	8.16	8.03	7.97	7.92	7.81	7.71
Cyclopropene	$1\,^3B_2$	6.19	6.14	6.09	6.06	6.04	6.00	5.96
Cyclopropene	$1\,^3B_1$	6.68	6.58	6.49	6.49	6.41	6.34	6.27

Table S19: ... continued

**VDZ Basis Set**		**Dynamical Correlation $\widetilde{\mathbf{E}}^{\mathrm{dyn}}$ [eV]**						
**Molecule**	**State**	**0**	**0.10**	**0.20**	**0.25**	**0.30**	**0.40**	**0.50**
Cyclopentadiene	$1\,^1B_2$	12.57	12.40	12.26	12.19	12.13	12.01	11.90
Cyclopentadiene	$2\,^1A_1$	11.14	10.94	10.78	10.71	10.65	10.53	10.43
Cyclopentadiene	$3\,^1A_1$	13.19	12.91	12.69	12.60	12.51	12.35	12.21
Cyclopentadiene	$1\,^3B_2$	10.34	10.25	10.16	10.12	10.09	10.02	9.95
Norbornadiene	$1\,^1A_2$	17.76	17.58	17.41	17.33	17.26	17.12	16.99
Norbornadiene	$1\,^1B_2$	18.52	18.29	18.08	17.98	17.89	17.71	17.55
Norbornadiene	$2\,^1B_2$	18.55	18.27	18.04	17.94	17.84	17.67	17.51
Norbornadiene	$2\,^1A_2$	18.19	17.98	17.79	17.71	17.62	17.46	17.32
Norbornadiene	$1\,^3A_2$	15.39	15.26	15.15	15.10	15.05	14.97	14.89
Norbornadiene	$1\,^3B_2$	15.32	15.19	15.08	15.03	14.98	14.89	14.80
Benzene	$2\,^1A'$	11.98	11.85	11.74	11.69	11.64	11.55	11.46
Benzene	$3\,^1A'$	13.76	13.54	13.37	13.29	13.21	13.07	12.94
Benzene	$4\,^1A'$	14.41	14.12	13.89	13.78	13.68	13.50	13.33
Benzene	$5\,^1A'$	14.41	14.12	13.89	13.78	13.68	13.50	13.33
Benzene	$6\,^1A'$	11.98	11.79	11.66	11.60	11.55	11.44	11.34
Benzene	$7\,^1A'$	11.98	11.78	11.64	11.58	11.52	11.40	11.30
Benzene	$1\,^3A'$	11.72	11.60	11.51	11.47	11.43	11.35	11.28
Benzene	$2\,^3A'$	12.16	11.98	11.86	11.80	11.75	11.65	11.56
Benzene	$3\,^3A'$	12.16	11.97	11.85	11.79	11.74	11.64	11.55
Benzene	$4\,^3A'$	13.75	13.53	13.35	13.27	13.19	13.05	12.92
Naphthalene	$1\,^1B_{3u}$	19.96	19.77	19.64	19.58	19.52	19.41	19.30
Naphthalene	$1\,^1B_{2u}$	21.70	21.50	21.33	21.26	21.19	21.06	20.94
Naphthalene	$2\,^1A_g$	19.90	19.67	19.51	19.43	19.36	19.22	19.10
Naphthalene	$1\,^1B_{1g}$	20.45	20.15	19.97	19.89	19.82	19.67	19.54
Naphthalene	$2\,^1B_{3u}$	21.63	21.31	21.06	20.96	20.85	20.66	20.49
Naphthalene	$2\,^1B_{2u}$	21.73	21.50	21.32	21.24	21.16	21.02	20.88
Naphthalene	$3\,^1A_g$	20.18	19.87	19.68	19.60	19.52	19.36	19.22
Naphthalene	$3\,^1B_{2u}$	20.15	19.83	19.59	19.49	19.39	19.21	19.04
Furan	$1\,^1B_2$	12.97	12.75	12.59	12.59	12.45	12.32	12.20
Furan	$3\,^1A_1$	13.71	13.27	12.99	12.86	12.75	12.54	12.36
Furan	$1\,^3B_2$	10.96	10.86	10.78	10.74	10.71	10.64	10.58
Furan	$1\,^3A_1$	11.18	11.04	10.93	10.88	10.84	10.75	10.66
Pyrrole	$1\,^1B_2$	12.51	12.31	12.15	12.07	12.00	11.88	11.76
Pyrrole	$3\,^1A_1$	12.66	12.35	12.13	12.03	11.94	11.78	11.63
Pyrrole	$1\,^3B_2$	10.73	10.63	10.55	10.51	10.48	10.41	10.35
Pyrrole	$1\,^3A_1$	11.08	10.93	10.81	10.76	10.71	10.61	10.53
Imidazole	$2\,^1A'$	12.42	12.21	12.04	11.96	11.89	11.77	11.64
Imidazole	$3\,^1A'$	13.19	12.99	12.84	12.76	12.70	12.57	12.45

Table S19: ... continued

VDZ Basis Set		Dynamical Correlation $\widetilde{\mathbf{E}}^{\mathrm{dyn}}$ [eV]						
**Molecule**	**State**	**0**	**0.10**	**0.20**	**0.25**	**0.30**	**0.40**	**0.50**
Pyridine	$2\,^1A_1$	14.48	14.01	13.80	13.70	13.59	13.41	13.25
Pyridine	$3\,^1A_1$	15.13	14.81	14.56	14.45	14.35	14.16	13.99
Pyridine	$1\,^1B_1$	12.48	12.36	12.27	12.23	11.83	11.75	11.67
Pyridine	$1\,^1A_2$	12.88	12.75	12.64	12.64	12.81	12.74	12.68
Pyridine	$1\,^3A_1$	12.20	12.07	11.98	11.93	11.89	11.81	11.73
Pyridine	$1\,^3B_2$	12.88	12.65	12.51	12.45	12.39	12.28	12.18
Pyrazine	$1\,^1B_{2u}$	13.13	12.95	12.82	12.76	12.70	12.60	12.50
Pyrazine	$1\,^1B_{1u}$	14.82	14.61	14.44	14.36	14.29	14.15	14.02
Pyrazine	$2\,^1B_{1u}$	15.90	15.60	15.37	15.26	15.16	14.97	14.80
Pyrazine	$2\,^1B_{2u}$	15.52	15.23	15.00	14.89	14.79	14.61	14.44
Pyrazine	$1\,^1B_{3u}$	13.58	13.42	13.29	13.23	13.18	13.08	12.98
Pyrazine	$1\,^1B_{2g}$	12.94	12.78	12.67	12.62	13.66	13.52	13.39
Pyrazine	$1\,^1B_{1g}$	14.04	13.70	13.55	13.46	13.38	13.23	13.10
Pyrimidine	$1\,^1B_2$	12.99	12.83	12.71	12.65	12.60	12.50	12.40
Pyrimidine	$2\,^1A_1$	14.76	14.41	14.21	14.12	14.04	13.88	13.75
Pyrimidine	$3\,^1A_1$	14.75	14.48	14.28	14.18	14.09	13.93	13.78
Pyrimidine	$2\,^1B_2$	15.94	15.61	15.37	15.26	15.15	14.96	14.78
Pyrimidine	$1\,^1B_1$	13.34	13.19	13.08	13.03	12.98	12.89	12.80
Pyrimidine	$1\,^1A_2$	13.67	13.50	13.37	13.31	13.26	13.15	13.05
Pyridazine	$2\,^1A_1$	12.97	12.82	12.70	12.65	12.59	12.49	12.39
Pyridazine	$1\,^1B_2$	15.75	15.40	15.17	15.07	14.98	14.81	14.65
Pyridazine	$2\,^1B_2$	15.73	15.42	15.18	15.08	14.98	14.80	14.63
Pyridazine	$3\,^1A_1$	13.15	13.03	12.94	12.90	12.86	12.78	12.71
Pyridazine	$1\,^1B_1$	13.74	13.57	13.45	13.39	13.34	13.23	13.14
Pyridazine	$2\,^1A_2$	13.62	13.41	13.25	13.18	13.12	13.00	12.88
Pyridazine	$2\,^1B_1$	13.77	13.53	13.36	13.28	13.21	13.08	12.96
Triazine	$2\,^1A'$	13.38	13.22	13.11	13.05	13.00	12.90	12.80
Triazine	$3\,^1A'$	15.88	15.62	15.42	15.33	15.24	15.09	14.95
Triazine	$4\,^1A'$	14.65	14.38	14.17	14.08	13.99	13.83	13.67
Triazine	$2\,^1A''$	15.18	14.81	14.63	14.56	14.49	14.33	14.18
Triazine	$3\,^1A''$	14.11	13.89	13.71	13.63	13.55	13.41	13.27
Triazine	$4\,^1A''$	14.49	14.28	14.11	14.03	13.95	13.81	13.68
Tetrazine	$1\,^1B_{2u}$	14.20	13.99	13.84	13.77	13.70	13.58	13.47
Tetrazine	$1\,^1B_{1u}$	15.83	15.41	15.12	14.99	14.87	14.66	14.47
Tetrazine	$2\,^1B_{1u}$	16.91	16.56	16.29	16.18	16.07	15.87	15.68
Tetrazine	$2\,^1B_{2u}$	14.79	14.49	14.29	14.19	14.10	13.93	13.78
Tetrazine	$1\,^1B_{3u}$	15.13	14.94	14.81	14.74	14.68	14.57	14.46
Tetrazine	$1\,^1A_u$	15.43	15.18	15.00	14.92	14.85	14.71	14.58

Table S19: ... continued

VDZ Basis Set		Dynamical Correlation $\widetilde{\mathbf{E}}^{\mathrm{dyn}}$ [eV]						
**Molecule**	**State**	**0**	**0.10**	**0.20**	**0.25**	**0.30**	**0.40**	**0.50**
Tetrazine	$2\,^1B_{2g}$	15.07	14.77	14.56	14.47	14.38	14.21	14.06
Tetrazine	$2\,^1B_{1g}$	14.98	14.71	14.48	14.38	14.28	14.10	13.93
Tetrazine	$2\,^1B_{3u}$	15.19	14.91	14.70	14.61	14.52	14.36	14.21
Tetrazine	$1\,^3B_{3u}$	16.63	16.42	16.28	16.21	16.14	16.02	15.90
Tetrazine	$1\,^3A_u$	16.43	16.16	15.97	15.89	15.81	15.66	15.52
Tetrazine	$1\,^3B_{1g}$	15.09	14.86	14.70	14.63	14.56	14.43	14.31
Formaldehyde	$1\,^1A_2$	4.45	4.40	4.35	4.33	4.30	4.26	4.22
Formaldehyde	$1\,^3A_2$	4.55	4.47	4.41	4.38	4.35	4.29	4.24
Formaldehyde	$1\,^3A_1$	3.84	3.81	3.78	3.76	3.75	3.72	3.70
Acetone	$1\,^1A_2$	9.05	8.98	8.92	8.89	8.86	8.81	8.76
Acetone	$1\,^3A_2$	8.66	8.58	8.51	8.51	8.45	8.39	8.34
Acetone	$1\,^3A_1$	8.51	8.46	8.41	8.39	8.37	8.33	8.29
Benzoquinone	$1\,^1B_{1g}$	17.50	17.25	17.09	17.02	16.95	16.83	16.71
Benzoquinone	$1\,^1A_u$		17.19	17.04	16.98	16.91	16.80	16.68
Benzoquinone	$1\,^1B_{3g}$	18.56	18.19	17.99	17.90	17.82	17.67	17.53
Benzoquinone	$1\,^1B_{1u}$	20.12	19.76	19.53	19.43	19.33	19.16	19.00
Benzoquinone	$2\,^1B_{1u}$	21.51	21.05	20.71	20.56	20.41	20.14	19.90
Benzoquinone	$1\,^3B_{1g}$	17.49	17.22	17.05	16.98	16.91	16.77	16.65
Benzoquinone	$1\,^3A_u$	17.37	17.14	16.99	16.92	16.85	16.73	16.61
Formamide	$1\,^1A''$	7.26	7.20	7.15	7.13	7.10	7.06	7.02
Formamide	$2\,^1A'$	10.12	9.87	9.69	9.61	9.53	9.39	9.26
Formamide	$1\,^3A''$	7.27	7.19	7.12	7.09	7.06	7.00	6.95
Acetamide	$1\,^1A''$	9.70	9.65	9.59	9.57	9.54	9.50	9.45
Acetamide	$2\,^1A'$	12.62	12.40	12.22	12.13	12.06	11.91	11.78
Acetamide	$3\,^1A'$	12.57	12.37	12.19	12.11	12.03	11.89	11.75
Propanamide	$1\,^1A''$	12.17	12.11	12.06	12.04	12.01	11.96	11.92
Propanamide	$2\,^1A'$	15.16	14.93	14.75	14.66	14.58	14.44	14.31
Propanamide	$3\,^1A'$	15.18	14.95	14.76	14.68	14.59	14.44	14.30
Cytosine	$2\,^1A'$	20.25	20.07	19.94	19.89	19.83	19.73	19.63
Cytosine	$1\,^1A''$	19.73	19.55	19.42	19.36	19.30	19.19	19.08
Cytosine	$3\,^1A'$	20.82	20.54	20.35	20.26	20.18	20.04	19.90
Cytosine	$4\,^1A'$	21.82	21.49	21.27	21.17	21.07	20.90	20.74
Cytosine	$5\,^1A'$	20.87	20.58	20.38	20.29	20.21	20.05	19.90
Thymine	$2\,^1A'$	23.98	23.75	23.58	23.51	23.44	23.30	23.18
Thymine	$3\,^1A'$	23.64	23.33	23.13	23.04	22.96	22.81	22.67
Thymine	$4\,^1A'$	24.33	24.01	23.79	23.69	23.60	23.43	23.28
Thymine	$5\,^1A'$	24.43	24.09	23.87	23.77	23.67	23.50	23.34
Uracil	$1\,^1A''$	19.40	19.27	19.18	19.14	19.09	19.02	18.94

Table S19: . . . continued

VDZ Basis Set		Dynamical Correlation $\widetilde{\mathbf{E}}^{\text{dyn}}$ [eV]						
**Molecule**	**State**	**0**	**0.10**	**0.20**	**0.25**	**0.30**	**0.40**	**0.50**
Uracil	$2\ ^1A'$	21.31	21.09	20.93	20.86	20.79	20.66	20.54
Uracil	$3\ ^1A'$	21.01	20.76	20.59	20.51	20.44	20.30	20.17
Uracil	$4\ ^1A'$	21.67	21.37	21.16	21.06	20.98	20.82	20.67
Uracil	$5\ ^1A'$	21.84	21.51	21.28	21.18	21.09	20.92	20.76
Adenine	$2\ ^1A'$	24.28	24.01	23.83	23.75	23.67	23.53	23.40
Adenine	$3\ ^1A'$	25.44	25.16	24.98	24.90	24.82	24.68	24.55
Adenine	$4\ ^1A'$	26.00	25.47	25.21	25.09	24.99	24.80	24.62
Adenine	$5\ ^1A'$	24.82	24.22	23.99	23.90	23.81	23.65	23.50
Adenine	$6\ ^1A'$	24.89	24.53	24.31	24.20	24.11	23.93	23.76
Adenine	$7\ ^1A'$	25.38	24.56	24.32	24.22	24.12	23.93	23.76

Table S20: CASPT2 approximate dynamical correlation energies $\widetilde{E}^{\text{dyn}}$ for the Thiel benchmark set obtained using the ANO-RCC-VDZP basis set and different IPEA shift parameters ($\varepsilon = 0$, 0.10, 0.20, 0.25, 0.30, 0.40, and 0.50 a.u.).

VDZP Basis Set		Dynamical Correlation $\widetilde{\mathbf{E}}^{\text{dyn}}$ [eV]						
**Molecule**	**State**	**0**	**0.10**	**0.20**	**0.25**	**0.30**	**0.40**	**0.50**
Ethene	$1\ ^1B_{1u}$	8.53	8.39	8.27	8.21	8.15	8.04	7.94
Ethene	$1\ ^3B_{1u}$	6.86	6.79	6.72	6.69	6.66	6.60	6.55
Butadiene	$1\ ^1B_u$	15.98	15.77	15.59	15.51	15.43	15.29	15.15
Butadiene	$1\ ^3B_u$	13.80	13.67	13.56	13.51	13.46	13.36	13.27
Butadiene	$1\ ^3A_g$	13.75	13.60	13.48	13.42	13.36	13.25	13.15
Hexatriene	$1\ ^1B_u$	22.82	22.57	22.37	22.29	22.20	22.05	21.90
Hexatriene	$2\ ^1A_g$	20.77	20.57	20.41	20.34	20.26	20.13	20.00
Hexatriene	$1\ ^3B_u$	20.36	20.24	20.18	20.13	20.13	20.03	19.93
Hexatriene	$1\ ^3A_g$	20.51	20.32	20.17	20.11	20.04	19.91	19.79
Octatetraene	$2\ ^1A_g$	27.35	27.12	26.95	26.87	26.79	26.64	26.50
Octatetraene	$1\ ^1B_u$	29.36	29.11	28.91	28.82	28.73	28.57	28.42
Octatetraene	$2\ ^1B_u$	28.28	28.00	27.79	27.69	27.60	27.42	27.25
Octatetraene	$1\ ^3B_u$	28.41	28.21	28.07	28.01	27.95	27.83	27.72
Octatetraene	$1\ ^3A_g$	25.79	25.55	25.39	25.31	25.24	25.10	24.96
Cyclopropene	$1\ ^1B_1$	11.19	11.07	10.95	10.90	10.84	10.74	10.64
Cyclopropene	$1\ ^1B_2$	12.60	12.42	12.26	12.19	12.11	11.98	11.85
Cyclopropene	$1\ ^3B_2$	10.42	10.33	10.25	10.21	10.17	10.10	10.04
Cyclopropene	$1\ ^3B_1$	11.02	10.87	10.75	10.69	10.63	10.53	10.43
Cyclopentadiene	$1\ ^1B_2$	19.64	19.43	19.26	19.18	19.10	18.95	18.81
Cyclopentadiene	$2\ ^1A_1$	18.35	18.06	17.84	17.74	17.65	17.48	17.33
Cyclopentadiene	$3\ ^1A_1$	20.48	20.11	19.68	19.57	19.56	19.33	19.13

Table S20: ... continued

**VDZP Basis Set**		**Dynamical Correlation $\widetilde{\mathbf{E}}^{\mathrm{dyn}}$ [eV]**						
**Molecule**	**State**	**0**	**0.10**	**0.20**	**0.25**	**0.30**	**0.40**	**0.50**
Cyclopentadiene	$1\,^3B_2$	17.29	17.15	17.03	16.98	16.92	16.82	16.72
Norbornadiene	$1\,^1A_2$	27.63	27.41	27.21	27.11	27.02	26.85	26.69
Norbornadiene	$1\,^1B_2$	28.24	27.94	27.67	27.55	27.43	27.20	26.99
Norbornadiene	$2\,^1B_2$	27.49	27.21	26.98	26.87	26.77	26.58	26.41
Norbornadiene	$2\,^1A_2$	28.13	27.88	27.66	27.55	27.45	27.26	27.09
Benzene	$2\,^1A'$	20.14	19.95	19.79	19.71	19.64	19.50	19.37
Benzene	$3\,^1A'$	22.08	21.81	21.58	21.47	21.37	21.18	21.01
Benzene	$4\,^1A'$	22.83	22.47	22.17	22.04	21.91	21.67	21.46
Benzene	$5\,^1A'$	22.82	22.47	22.17	22.03	21.91	21.67	21.45
Benzene	$6\,^1A'$	20.29	20.02	19.83	19.74	19.66	19.49	19.34
Benzene	$7\,^1A'$	20.28	20.01	19.81	19.71	19.62	19.45	19.29
Benzene	$1\,^3A'$	19.87	19.69	19.55	19.48	19.42	19.29	19.18
Benzene	$2\,^3A'$	22.61	22.37	22.18	22.10	22.02	21.87	21.73
Benzene	$3\,^3A'$	22.61	22.36	22.18	22.09	22.01	21.86	21.72
Benzene	$4\,^3A'$	22.14	21.86	21.63	21.52	21.41	21.22	21.04
Naphthalene	$1\,^1B_{3u}$	33.03	32.77	32.57	32.48	32.39	32.23	32.07
Naphthalene	$1\,^1B_{2u}$	34.89	34.62	34.41	34.31	34.22	34.04	33.87
Naphthalene	$2\,^1A_g$	33.05	32.73	32.49	32.38	32.27	32.07	31.87
Naphthalene	$1\,^1B_{1g}$	33.69	33.30	33.05	32.93	32.82	32.61	32.41
Naphthalene	$2\,^1B_{3u}$	35.44	35.02	34.71	34.56	34.43	34.18	33.95
Naphthalene	$2\,^1B_{2u}$	34.95	34.65	34.41	34.30	34.19	34.00	33.81
Naphthalene	$3\,^1A_g$	33.43	33.03	32.76	32.64	33.69	33.50	33.33
Naphthalene	$3\,^1B_{2u}$	35.43	35.02	34.71	34.57	34.44	34.19	33.96
Furan	$1\,^1B_2$	20.69	20.35	20.15	20.06	19.97	19.81	19.65
Furan	$3\,^1A_1$	21.45	20.94	20.59	20.43	20.29	20.03	19.80
Furan	$1\,^3B_2$	18.25	18.11	18.00	17.95	17.90	17.80	17.71
Furan	$1\,^3A_1$	18.55	18.36	18.20	18.13	18.06	17.94	17.82
Pyrrole	$1\,^1B_2$	20.03	19.76	19.55	19.45	19.36	19.18	19.02
Pyrrole	$3\,^1A_1$	20.27	19.90	19.62	19.50	19.38	19.16	18.96
Pyrrole	$1\,^3B_2$	18.06	17.93	17.81	17.76	17.71	17.61	17.51
Pyrrole	$1\,^3A_1$	18.51	18.31	18.14	18.06	17.99	17.85	17.72
Imidazole	$2\,^1A'$	20.20	19.91	19.49	19.59	19.50	19.32	19.15
Imidazole	$3\,^1A'$	20.62	20.39	20.07	20.11	20.02	19.86	19.71
Pyridine	$2\,^1A_1$	23.15	22.63	22.33	22.19	22.07	21.84	21.63
Pyridine	$3\,^1A_1$	23.54	23.15	22.84	22.70	22.57	22.32	22.10
Pyridine	$1\,^1B_1$	20.81	20.65	20.52	20.46	20.08	19.96	19.85
Pyridine	$1\,^1A_2$	21.27	21.09	20.95	20.89	21.23	21.12	21.02
Pyridine	$1\,^3A_1$	20.49	20.29	20.15	20.08	20.01	19.88	19.76

Table S20: . . . continued

VDZP Basis Set		Dynamical Correlation $\widetilde{\mathbf{E}}^{\mathrm{dyn}}$ [eV]						
**Molecule**	**State**	**0**	**0.10**	**0.20**	**0.25**	**0.30**	**0.40**	**0.50**
Pyridine	$1\,^3B_2$	21.43	21.10	20.88	20.79	20.70	20.54	20.39
Pyrazine	$1\,^1B_{2u}$	21.65	21.40	21.21	21.12	21.04	20.89	20.74
Pyrazine	$1\,^1B_{1u}$	23.30	23.03	22.82	22.72	22.62	22.45	22.28
Pyrazine	$2\,^1B_{1u}$	24.30	23.95	23.67	23.54	23.41	23.18	22.97
Pyrazine	$2\,^1B_{2u}$	24.00	23.67	23.40	23.27	23.15	22.92	22.71
Pyrazine	$1\,^1B_{3u}$	22.19	21.97	21.80	21.72	21.64	21.50	21.36
Pyrazine	$1\,^1B_{2g}$	21.57	21.36	21.19	21.12	21.04	20.90	20.77
Pyrazine	$1\,^1B_{1g}$	22.77	22.41	22.15	22.03	21.92	21.71	21.52
Pyrimidine	$1\,^1B_2$	21.52	21.29	21.10	21.02	20.94	20.78	20.64
Pyrimidine	$2\,^1A_1$	23.40	23.04	22.81	22.71	22.61	22.43	22.27
Pyrimidine	$3\,^1A_1$	23.35	23.00	22.72	22.60	22.48	22.25	22.05
Pyrimidine	$2\,^1B_2$	24.79	24.33	23.98	23.81	23.66	23.39	23.14
Pyrimidine	$1\,^1B_1$	21.89	21.70	21.54	21.47	21.40	21.27	21.15
Pyrimidine	$1\,^1A_2$	22.26	22.05	21.88	21.80	21.73	21.58	21.44
Pyridazine	$2\,^1A_1$	21.48	21.26	21.09	21.00	20.92	20.77	20.63
Pyridazine	$1\,^1B_2$	24.35	23.94	23.67	23.55	23.44	23.22	23.03
Pyridazine	$2\,^1B_2$	24.33	23.90	23.59	23.45	23.31	23.07	22.85
Pyridazine	$3\,^1A_1$	24.05	22.39	22.07	21.93	21.80	21.55	21.33
Pyridazine	$1\,^1B_1$	22.38	22.16	22.00	21.92	21.84	21.70	21.57
Pyridazine	$2\,^1A_2$	22.17	21.92	21.73	21.64	21.55	21.38	21.23
Pyridazine	$2\,^1B_1$	22.40	22.11	21.89	21.79	21.70	21.52	21.36
Triazine	$2\,^1A'$	21.97	21.77	21.60	21.52	21.44	21.30	21.16
Triazine	$3\,^1A'$	24.05	23.75	23.52	23.41	23.31	23.13	22.95
Triazine	$4\,^1A'$	25.75	25.33	25.01	24.87	24.73	24.48	24.24
Triazine	$2\,^1A''$	23.05	22.62	22.40	22.29	22.19	22.01	21.83
Triazine	$3\,^1A''$	23.29	23.00	22.76	22.65	22.55	22.35	22.17
Triazine	$4\,^1A''$	23.23	22.98	22.74	22.64	22.54	22.34	22.16
Tetrazine	$1\,^1B_{2u}$	23.01	22.74	22.53	22.43	22.34	22.17	22.01
Tetrazine	$1\,^1B_{1u}$	25.61	25.25	24.97	24.84	24.72	24.48	24.27
Tetrazine	$2\,^1B_{1u}$	26.00	25.62	25.32	25.19	25.06	24.82	24.60
Tetrazine	$2\,^1B_{2u}$	24.08	23.66	23.36	23.22	23.10	22.86	22.64
Tetrazine	$1\,^1B_{3u}$	23.96	23.73	23.54	23.46	23.38	23.22	23.08
Tetrazine	$1\,^1A_u$	24.30	23.98	23.76	23.65	23.55	23.37	23.20
Tetrazine	$2\,^1B_{2g}$	25.20	24.69	24.41	24.28	24.16	23.93	23.71
Tetrazine	$2\,^1B_{1g}$	23.91	23.54	23.23	23.09	22.96	22.71	22.48
Tetrazine	$2\,^1B_{3u}$	24.10	23.75	23.48	23.36	23.24	23.02	22.81
Tetrazine	$1\,^3B_{3u}$	24.97	24.72	24.53	24.44	24.35	24.19	24.04
Tetrazine	$1\,^3A_u$	24.51	24.18	23.94	23.83	23.72	23.52	23.33

Table S20: ... continued

**VDZP Basis Set**		**Dynamical Correlation $\widetilde{\mathbf{E}}^{\mathrm{dyn}}$ [eV]**						
**Molecule**	**State**	**0**	**0.10**	**0.20**	**0.25**	**0.30**	**0.40**	**0.50**
Tetrazine	$1\,^3B_{1g}$	23.87	23.58	23.37	23.27	23.18	23.00	22.84
Formaldehyde	$1\,^1A_2$	7.83	7.75	7.68	7.64	7.61	7.54	7.48
Formaldehyde	$1\,^3A_2$	7.95	7.85	7.75	7.71	7.66	7.58	7.51
Formaldehyde	$1\,^3A_1$	7.24	7.17	7.11	7.08	7.05	7.00	6.95
Acetone	$1\,^1A_2$	15.48	15.39	15.31	15.27	15.23	15.16	15.09
Acetone	$1\,^3A_2$	15.22	15.10	15.00	14.95	14.90	14.82	14.73
Acetone	$1\,^3A_1$	14.94	14.86	14.78	14.75	14.72	14.65	14.59
Benzoquinone	$1\,^1B_{1g}$	28.89	28.60	28.38	28.29	28.20	28.02	27.86
Benzoquinone	$1\,^1A_u$	28.83	28.56	28.36	28.26	28.17	28.00	27.84
Benzoquinone	$1\,^1B_{3g}$	30.10	29.74	29.50	29.40	29.30	29.12	28.95
Benzoquinone	$1\,^1B_{1u}$	30.99	30.68	30.45	30.34	30.24	30.06	29.88
Benzoquinone	$2\,^1B_{1u}$	33.30	32.79	32.39	32.21	32.04	31.71	31.42
Benzoquinone	$1\,^3B_{1g}$	28.87	28.54	28.32	28.22	28.12	27.94	27.77
Benzoquinone	$1\,^3A_u$	28.78	28.49	28.27	28.18	28.08	27.91	27.74
Formamide	$1\,^1A''$	12.54	12.47	12.39	12.36	12.32	12.26	12.19
Formamide	$2\,^1A'$	15.14	14.90	14.70	14.62	14.53	14.37	14.23
Formamide	$1\,^3A''$	12.54	12.44	12.34	12.29	12.25	12.16	12.09
Acetamide	$1\,^1A''$	20.24	20.16	20.08	20.05	20.01	19.94	19.87
Acetamide	$2\,^1A'$	22.27	22.04	21.85	21.76	21.67	21.51	21.36
Acetamide	$3\,^1A'$	12.80	12.55	12.33	12.23	12.14	11.96	11.79
Propanamide	$1\,^1A''$	20.56	20.47	20.40	20.36	20.32	20.25	20.18
Propanamide	$2\,^1A'$	23.31	23.07	22.87	22.78	22.69	22.53	22.38
Propanamide	$3\,^1A'$	23.68	23.40	23.17	23.07	22.97	22.78	22.61
Cytosine	$2\,^1A'$	32.59	32.37	32.20	32.13	32.05	31.91	31.78
Cytosine	$1\,^1A''$	32.20	31.96	31.79	31.71	31.63	31.48	31.34
Cytosine	$3\,^1A'$	33.11	32.81	32.58	32.48	32.38	32.20	32.03
Cytosine	$4\,^1A'$	33.92	33.55	33.28	33.16	33.05	32.83	32.63
Cytosine	$5\,^1A'$	33.41	33.04	32.78	32.66	32.55	32.33	32.13
Thymine	$1\,^1A''$	35.93	35.76	35.63	35.57	35.51	35.41	35.30
Thymine	$2\,^1A'$	37.84	37.57	37.38	37.29	37.21	37.04	36.89
Thymine	$3\,^1A'$	37.64	37.32	37.09	36.99	36.90	36.72	36.55
Thymine	$4\,^1A'$	38.11	37.78	37.54	37.43	37.33	37.14	36.96
Thymine	$5\,^1A'$	38.23	37.86	37.59	37.47	37.36	37.15	36.95
Uracil	$1\,^1A''$	31.89	31.72	31.59	31.53	31.47	31.36	31.26
Uracil	$2\,^1A'$	33.63	33.37	33.18	33.09	33.00	32.84	32.69
Uracil	$3\,^1A'$	33.49	33.23	33.03	32.94	32.85	32.69	32.53
Uracil	$4\,^1A'$	33.90	33.58	33.35	33.24	33.14	32.95	32.77
Uracil	$5\,^1A'$	34.07	33.71	33.45	33.33	33.22	33.02	32.82

Table S20: ... continued

VDZP Basis Set		Dynamical Correlation $\widetilde{\mathbf{E}}^{\mathrm{dyn}}$ [eV]						
**Molecule**	**State**	**0**	**0.10**	**0.20**	**0.25**	**0.30**	**0.40**	**0.50**
Adenine	$2\ ^1A'$	38.87	38.55	38.31	38.21	38.11	37.92	37.74
Adenine	$3\ ^1A'$	40.00	39.68	39.46	39.36	39.26	39.07	38.90
Adenine	$1\ ^1A''$	39.22	39.05	38.77	38.68	38.59	38.42	38.27
Adenine	$4\ ^1A'$	40.67	40.20	39.90	39.76	39.63	39.39	39.16
Adenine	$5\ ^1A'$	39.24	38.81	38.55	38.44	38.33	38.12	37.93
Adenine	$6\ ^1A'$	39.66	39.22	38.93	38.80	38.68	38.45	38.23
Adenine	$7\ ^1A'$	39.83	39.25	38.95	38.82	38.69	38.44	38.22

Table S21: CASPT2 approximate dynamical correlation energies $\widetilde{E}^{\mathrm{dyn}}$for the Thiel benchmark set obtained using the ANO-RCC-VTZP basis set and different IPEA shift parameters ($\varepsilon = 0$, 0.10, 0.20, 0.25, 0.30, 0.40, and 0.50 a.u.).

VTZP Basis Set		Dynamical Correlation $\widetilde{\mathbf{E}}^{\mathrm{dyn}}$ [eV]						
**Molecule**	**State**	**0**	**0.10**	**0.20**	**0.25**	**0.30**	**0.40**	**0.50**
Ethene	$1\ ^1B_{1u}$	9.82	9.68	9.56	9.50	9.44	9.33	9.24
Ethene	$1\ ^3B_{1u}$	8.42	8.34	8.27	8.24	8.20	8.14	8.08
Butadiene	$1\ ^1B_u$	19.07	18.85	18.66	18.57	18.49	18.33	18.19
Butadiene	$1\ ^3B_u$	16.72	16.58	16.46	16.41	16.35	16.25	16.15
Butadiene	$1\ ^3A_g$	16.71	16.55	16.41	16.35	16.28	16.16	16.05
Hexatriene	$1\ ^1B_u$	27.46	26.96	26.76	26.67	26.58	26.41	26.25
Hexatriene	$2\ ^1A_g$	25.13	24.91	24.73	24.64	24.56	24.41	24.27
Hexatriene	$1\ ^3B_u$	24.82	24.64	24.50	24.44	24.38	24.27	24.17
Hexatriene	$1\ ^3A_g$	24.84	24.62	24.46	24.38	24.31	24.17	24.04
Octatetraene	$2\ ^1A_g$	33.03	32.78	32.59	32.51	32.42	32.26	32.10
Octatetraene	$1\ ^1B_u$	35.13	34.85	34.64	34.54	34.45	34.27	34.11
Octatetraene	$2\ ^1B_u$	33.25	32.93	32.69	32.58	32.47	32.27	32.09
Octatetraene	$1\ ^3B_u$	32.73	32.52	32.37	32.30	32.23	32.11	31.99
Octatetraene	$1\ ^3A_g$	32.77	32.51	32.33	32.25	32.17	32.01	31.87
Cyclopropene	$1\ ^1B_1$	13.47	13.34	13.21	13.15	13.09	12.98	12.88
Cyclopropene	$1\ ^1B_2$	14.62	14.44	14.28	14.20	14.12	13.99	13.86
Cyclopropene	$1\ ^3B_2$	12.64	12.55	12.46	12.41	12.37	12.30	12.22
Cyclopropene	$1\ ^3B_1$	13.29	13.13	13.00	12.93	12.87	12.76	12.65
Cyclopentadiene	$2\ ^1A_1$	22.19	21.86	21.60	21.48	21.38	21.18	21.01
Cyclopentadiene	$3\ ^1A_1$	24.64	24.07	23.72	23.57	23.42	23.15	22.91
Norbornadiene	$1\ ^1A_2$	31.65	31.48	31.32	31.25	31.18	31.04	30.92
Norbornadiene	$1\ ^1B_2$	31.12	30.94	30.79	30.72	30.65	30.51	30.39
Norbornadiene	$2\ ^1B_2$	31.74	31.56	31.39	31.30	31.23	31.08	30.94
Norbornadiene	$2\ ^1A_2$	31.80	31.63	31.47	31.39	31.32	31.19	31.06

Table S21: . . . continued

VTZP Basis Set		Dynamical Correlation $\widetilde{\mathbf{E}}^{\mathrm{dyn}}$ [eV]						
**Molecule**	**State**	**0**	**0.10**	**0.20**	**0.25**	**0.30**	**0.40**	**0.50**
Benzene	$2\,^1A'$	26.74	26.37	26.07	25.93	25.81	25.57	25.35
Benzene	$3\,^1A'$	26.39	26.09	25.85	25.74	25.63	25.43	25.24
Benzene	$4\,^1A'$	27.75	27.40	27.10	26.96	26.83	26.58	26.35
Benzene	$5\,^1A'$	27.75	27.38	27.08	26.94	26.81	26.56	26.32
Benzene	$6\,^1A'$	27.33	26.73	26.33	26.17	26.01	25.74	25.50
Benzene	$7\,^1A'$	27.31	26.71	26.31	26.14	25.99	25.71	25.47
Benzene	$1\,^3A'$	25.97	25.68	25.44	25.33	25.23	25.03	24.84
Benzene	$2\,^3A'$	25.87	25.58	25.34	25.23	25.13	24.93	24.75
Benzene	$3\,^3A'$	25.87	25.57	25.33	25.22	25.11	24.91	24.73
Benzene	$4\,^3A'$	26.25	25.98	25.75	25.65	25.55	25.35	25.18
Naphthalene	$1\,^1B_{3u}$	39.84	39.56	39.34	39.24	39.14	38.96	38.78
Naphthalene	$1\,^1B_{2u}$	41.77	41.48	41.26	41.15	41.05	40.86	40.68
Naphthalene	$2\,^1A_g$	40.04	39.65	39.36	39.23	39.11	38.87	38.65
Naphthalene	$1\,^1B_{1g}$	40.69	40.24	39.95	39.82	39.69	39.46	39.24
Naphthalene	$2\,^1B_{3u}$	42.45	41.98	41.63	41.49	41.40	41.05	40.80
Naphthalene	$2\,^1B_{2u}$	41.87	41.56	41.29	41.17	41.06	40.84	40.64
Naphthalene	$3\,^1A_g$	40.38	39.93	39.62	39.47	39.34	39.09	38.85
Naphthalene	$3\,^1B_{2u}$	42.51		42.35	41.55	41.40	41.12	40.87
Furan	$1\,^1B_2$	24.94	24.65	24.43	24.33	24.23	24.06	23.89
Furan	$3\,^1A_1$	26.02	25.42	25.01	24.83	24.66	24.36	24.09
Furan	$1\,^3B_2$	22.34	22.20	22.08	22.02	21.97	21.86	21.76
Furan	$1\,^3A_1$	22.67	22.47	22.30	22.22	22.15	22.01	21.88
Pyrrole	$1\,^1B_2$	24.11	23.78	23.53	23.42	23.31	23.11	22.94
Pyrrole	$3\,^1A_1$	24.41	23.98	23.66	23.51	23.38	23.13	22.91
Pyrrole	$1\,^3B_2$	21.87	21.73	21.60	21.54	21.49	21.38	21.28
Pyrrole	$1\,^3A_1$	22.37	22.14	21.96	21.87	21.79	21.64	21.50
Imidazole	$2\,^1A'$	25.75	25.32	25.02	24.87	24.79	24.58	24.39
Imidazole	$3\,^1A'$	23.53	23.31	23.07	22.97	22.88	22.70	22.53
Pyridine	$2\,^1A_1$	27.58		26.78	26.63	26.50	26.26	26.04
Pyridine	$3\,^1A_1$	27.01		26.22	26.07	25.92	25.65	25.41
Pyridine	$1\,^1B_1$	25.14		24.83	24.77	24.38	24.25	24.13
Pyridine	$1\,^1A_2$	25.59		25.26	25.19	25.47	25.35	25.24
Pyridine	$1\,^3A_1$	24.83		24.45	24.37	24.30	24.16	24.03
Pyridine	$1\,^3B_2$	25.94		25.31	25.20	25.10	24.91	24.75
Pyrazine	$1\,^1B_{2u}$	26.27	25.98	25.76	25.67	25.57	25.40	25.24
Pyrazine	$1\,^1B_{1u}$	27.86	27.59	27.36	27.25	27.15	26.96	26.78
Pyrazine	$2\,^1B_{1u}$	28.99	28.60	28.29	28.15	28.02	27.77	27.54
Pyrazine	$2\,^1B_{2u}$	28.67	28.30	28.00	27.86	27.73	27.48	27.25

Table S21: ... continued

VTZP Basis Set		Dynamical Correlation $\widetilde{\mathbf{E}}^{\mathrm{dyn}}$ [eV]						
**Molecule**	**State**	**0**	**0.10**	**0.20**	**0.25**	**0.30**	**0.40**	**0.50**
Pyrazine	$1\,^1B_{3u}$	26.73	26.50	26.31	26.23	26.15	25.99	25.84
Pyrazine	$1\,^1B_{2g}$	26.12	25.89	25.71	25.63	25.55	25.39	25.24
Pyrazine	$1\,^1B_{1g}$	27.60	27.00	26.71	26.58	26.46	26.24	26.03
Pyrimidine	$1\,^1B_2$		25.83	25.62	25.52	25.43	25.26	25.11
Pyrimidine	$2\,^1A_1$		27.66	27.41	27.31	27.20	27.01	26.83
Pyrimidine	$3\,^1A_1$		28.97	28.64	28.50	28.36	28.10	27.86
Pyrimidine	$2\,^1B_2$		28.99	28.61	28.44	28.28	27.98	27.71
Pyrimidine	$1\,^1B_1$		26.20	26.04	25.96	25.89	25.75	25.61
Pyrimidine	$1\,^1A_2$		26.56	26.38	26.29	26.21	26.06	25.91
Pyridazine	$2\,^1A_1$	26.02	25.78	25.58	25.49	25.40	25.23	25.07
Pyridazine	$1\,^1B_2$	28.24	27.80	27.51	27.38	27.25	27.02	26.81
Pyridazine	$2\,^1B_2$	29.00	28.52	28.16	28.01	27.86	27.59	27.35
Pyridazine	$3\,^1A_1$	29.14	28.67	28.32	28.16	28.01	27.74	27.50
Pyridazine	$1\,^1B_1$	26.90	26.67	26.49	26.40	26.32	26.17	26.03
Pyridazine	$2\,^1A_2$	26.71	26.44	26.23	26.14	26.04	25.86	25.70
Pyridazine	$2\,^1B_1$	26.96	26.64	26.41	26.30	26.20	26.01	25.83
Triazine	$2\,^1A'$	26.64	26.42	26.23	26.15	26.06	25.90	25.75
Triazine	$3\,^1A'$	28.93	28.49	28.23	28.11	28.00	27.80	27.61
Triazine	$4\,^1A'$	30.70	30.21	29.86	29.70	29.55	29.27	29.01
Triazine	$2\,^1A''$	28.12	27.71	27.45	27.34	27.26	27.03	26.84
Triazine	$3\,^1A''$	27.98	27.68	27.42	27.31	27.20	26.98	26.79
Triazine	$4\,^1A''$	27.52	27.27	27.02	26.90	26.80	26.59	26.39
Tetrazine	$1\,^1B_{2u}$	27.87	27.56	27.33	27.23	27.13	26.94	26.77
Tetrazine	$1\,^1B_{1u}$	30.47	30.09	29.79	29.65	29.52	29.28	29.06
Tetrazine	$2\,^1B_{1u}$	30.83	30.46	30.16	30.03	29.90	29.65	29.42
Tetrazine	$2\,^1B_{2u}$	29.05	28.55	28.22	28.08	27.94	27.68	27.45
Tetrazine	$1\,^1B_{3u}$	28.76	28.50	28.31	28.22	28.13	27.97	27.81
Tetrazine	$1\,^1A_u$	29.12	28.78	28.53	28.42	28.32	28.12	27.93
Tetrazine	$2\,^1B_{2g}$	28.80	28.98	28.12	27.98	27.85	27.60	27.37
Tetrazine	$2\,^1B_{1g}$	28.79	28.39	28.06	27.90	27.76	27.49	27.24
Tetrazine	$2\,^1B_{3u}$	28.96	28.58	28.29	28.16	28.03	27.80	27.58
Tetrazine	$1\,^3B_{3u}$	29.73	29.47	29.26	29.17	29.07	28.90	28.74
Tetrazine	$1\,^3A_u$	29.28	28.94	28.68	28.56	28.44	28.23	28.03
Tetrazine	$1\,^3B_{1g}$	28.65	28.34	28.12	28.01	27.91	27.73	27.55
Formaldehyde	$1\,^1A_2$	9.88	9.80	9.72	9.69	9.65	9.58	9.51
Formaldehyde	$1\,^3A_2$	9.99	9.88	9.78	9.73	9.69	9.60	9.53
Formaldehyde	$1\,^3A_1$	9.28	9.21	9.14	9.11	9.08	9.02	8.97
Acetone	$1\,^1A_2$	18.77	18.67	18.57	18.53	18.49	18.41	18.33

Table S21: . . . continued

VTZP Basis Set		Dynamical Correlation $\widetilde{\mathbf{E}}^{\rm dyn}$ [eV]						
**Molecule**	**State**	**0**	**0.10**	**0.20**	**0.25**	**0.30**	**0.40**	**0.50**
Acetone	$1\,^3A_2$	18.81	18.69	18.58	18.53	18.48	18.39	18.30
Acetone	$1\,^3A_1$	18.54	18.46	18.38	18.34	18.31	18.24	18.17
Benzoquinone	$1\,^1B_{1g}$	35.33	35.02	34.80	34.70	34.60	34.42	34.24
Benzoquinone	$1\,^1A_u$	35.29	34.99	34.77	34.68	34.58	34.40	34.23
Benzoquinone	$1\,^1B_{3g}$	36.62	36.24	35.99	35.88	35.78	35.58	35.41
Benzoquinone	$1\,^1B_{1u}$	37.50	37.17	36.92	36.81	36.71	36.51	36.33
Benzoquinone	$2\,^1B_{1u}$	39.92	39.39	38.98	38.79	39.37	39.12	38.90
Benzoquinone	$1\,^3B_{1g}$	35.29	34.96	34.72	34.61	34.51	34.32	34.13
Benzoquinone	$1\,^3A_u$	35.21	34.90	34.68	34.58	34.48	34.29	34.12
Formamide	$1\,^1A''$	15.62	15.54	15.46	15.42	15.38	15.31	15.24
Formamide	$2\,^1A'$	18.36	18.04	17.82	17.72	17.63	17.45	17.29
Formamide	$1\,^3A''$	15.72	15.61	15.52	15.48	15.44	15.36	15.28
Acetamide	$1\,^1A''$	20.41	20.33	20.24	20.20	20.16	20.09	20.01
Acetamide	$2\,^1A'$		23.11	22.78	22.67	22.56	22.38	22.21
Acetamide	$3\,^1A'$	23.49	23.48	23.06	22.93	22.82	22.61	22.43
Propanamide	$1\,^1A''$	25.20	25.11	25.03	24.99	24.95	24.87	24.80
Propanamide	$2\,^1A'$	27.42	27.23	27.06	26.99	26.92	26.78	26.66
Propanamide	$3\,^1A'$	27.78	27.60	27.44	27.36	27.29	27.16	27.04
Cytosine	$2\,^1A'$	39.44	39.20	39.01	38.93	38.85	38.70	38.55
Cytosine	$1\,^1A''$	39.02	38.79	38.60	38.52	38.43	38.28	38.13
Cytosine	$3\,^1A'$	40.08	39.73	39.48	39.36	39.25	39.05	38.86
Cytosine	$4\,^1A'$	41.08	40.63	40.28	40.14	40.01	39.76	39.53
Cytosine	$5\,^1A'$	40.43	40.04	39.73	39.60	39.47	39.24	39.02
Thymine	$1\,^1A''$	43.80	43.63	43.49	43.43	43.37	43.25	43.14
Thymine	$2\,^1A'$	45.80	45.52	45.31	45.18	45.08	44.95	44.77
Thymine	$3\,^1A'$	45.51	45.41	45.15	44.83	44.75	44.62	44.42
Thymine	$4\,^1A'$		46.11	45.83	45.56			
Thymine	$5\,^1A'$		46.33	45.96	45.87			
Uracil	$1\,^1A''$	39.06	38.80	38.66	38.60	38.54	38.42	38.31
Uracil	$2\,^1A'$	40.75	40.47	40.27	40.17	40.08	39.91	39.74
Uracil	$3\,^1A'$	40.64	40.35	40.14	40.04	39.95	39.77	39.60
Uracil	$4\,^1A'$	41.16	40.81	40.55	40.43	40.32	40.11	39.92
Uracil	$5\,^1A'$	41.32	40.91	40.63	40.50	40.37	40.14	39.93
Adenine	$2\,^1A'$	48.02	47.69	47.45	47.34	47.23	47.03	46.84
Adenine	$3\,^1A'$	46.72	46.44	46.18	46.06	45.95	45.74	45.55
Adenine	$1\,^1A''$	47.46	46.95	46.65	46.55	46.45	46.28	46.11
Adenine	$4\,^1A'$	48.84	48.35	47.97	47.82	47.67	47.39	47.14
Adenine	$5\,^1A'$	47.30	46.87	46.56	46.42	46.30	46.06	45.85

Table S21: . . . continued

VTZP Basis Set		Dynamical Correlation $\widetilde{\mathbf{E}}^{\mathrm{dyn}}$ [eV]						
**Molecule**	**State**	**0**	**0.10**	**0.20**	**0.25**	**0.30**	**0.40**	**0.50**
Adenine	$6\,^1A'$		47.31	46.95	46.80	46.66	46.39	46.15
Adenine	$7\,^1A'$		47.43	47.03	46.87	46.73	46.46	46.21

Table S22: CASPT2 approximate dynamical correlation energies $\widetilde{E}^{\mathrm{dyn}}$for the Thiel benchmark set obtained using the ANO-RCC-VQZP basis set and different IPEA shift parameters ($\varepsilon = 0$, 0.10, 0.20, 0.25, 0.30, 0.40, and 0.50 a.u.).

VQZP Basis Set		Dynamical Correlation $\widetilde{\mathbf{E}}^{\mathrm{dyn}}$ [eV]						
**Molecule**	**State**	**0**	**0.10**	**0.20**	**0.25**	**0.30**	**0.40**	**0.50**
Ethene	$1\,^1B_{1u}$	9.92	9.80	9.69	9.63	9.58	9.49	9.40
Ethene	$1\,^3B_{1u}$	8.90	8.82	8.74	8.71	8.67	8.61	8.55
Butadiene	$1\,^1B_u$	20.07	19.85	19.65	19.56	19.48	19.32	19.17
Butadiene	$1\,^3B_u$	17.64	17.50	17.38	17.32	17.27	17.16	17.06
Butadiene	$1\,^3A_g$	17.65	17.48	17.34	17.27	17.20	17.08	16.96
Hexatriene	$1\,^1B_u$	28.66	28.45	28.13	28.36	28.14	27.76	27.64
Hexatriene	$2\,^1A_g$	26.67	26.31	26.11	26.02	25.94	25.78	25.63
Hexatriene	$1\,^3B_u$	26.18	25.99	25.86	25.80	25.74	25.62	25.52
Hexatriene	$1\,^3A_g$	26.21	25.99	25.82	25.74	25.66	25.52	25.39
Octatetraene	$2\,^1A_g$	34.84	34.58	34.40	34.31	34.22	34.05	33.90
Octatetraene	$1\,^1B_u$	36.96	36.69	36.47	36.37	36.27	36.10	35.93
Octatetraene	$2\,^1B_u$	35.19	34.81	34.55	34.43	34.31	34.10	33.91
Octatetraene	$1\,^3B_u$	34.53	34.31	34.16	34.09	34.03	33.90	33.78
Octatetraene	$1\,^3A_g$	34.57	34.31	34.13	34.04	33.96	33.80	33.66
Cyclopropene	$1\,^1B_1$	14.18	14.04	13.91	13.85	13.79	13.68	13.57
Cyclopropene	$1\,^1B_2$	14.98	14.81	14.66	14.58	14.52	14.38	14.26
Cyclopropene	$1\,^3B_2$	13.33	13.23	13.14	13.09	13.05	12.97	12.90
Cyclopropene	$1\,^3B_1$	13.98	13.83	13.69	13.63	13.56	13.45	13.34
Cyclopentadiene	$1\,^1B_2$	24.48		24.08	24.00	23.91	23.75	23.60
Cyclopentadiene	$2\,^1A_1$	23.50		22.82	22.70	22.59	22.39	22.21
Cyclopentadiene	$3\,^1A_1$	25.82		24.98	24.81	24.66	24.38	24.12
Cyclopentadiene	$1\,^3B_2$	22.03		21.74	21.68	21.62	21.51	21.40
Norbornadiene	$1\,^1A_2$	33.06	32.90	32.75	32.68	32.61	32.48	32.36
Norbornadiene	$1\,^1B_2$	32.58	32.41	32.26	32.19	32.13	32.00	31.88
Norbornadiene	$2\,^1B_2$	33.20	33.03	32.86	32.78	32.71	32.57	32.44
Norbornadiene	$2\,^1A_2$	33.24	33.07	32.91	32.84	32.77	32.64	32.52
Norbornadiene	$1\,^3A_2$	31.85	31.64	31.47	31.40	31.32	31.18	31.05
Norbornadiene	$1\,^3B_2$	31.76	31.55	31.37	31.04	31.21	31.06	30.92
Benzene	$2\,^1A'$	28.08	27.71	27.41	27.27	27.15	26.91	26.68

Table S22: ... continued

**VQZP Basis Set**		**Dynamical Correlation** $\widetilde{\mathbf{E}}^{\mathrm{dyn}}$ **[eV]**						
**Molecule**	**State**	**0**	**0.10**	**0.20**	**0.25**	**0.30**	**0.40**	**0.50**
Benzene	$3\ ^1A'$	27.76	27.45	27.20	27.09	26.98	26.78	26.59
Benzene	$4\ ^1A'$	29.10	28.74	28.43	28.29	28.16	27.91	27.67
Benzene	$5\ ^1A'$	29.10	28.73	28.42	28.27	28.14	27.88	27.64
Benzene	$6\ ^1A'$	28.77	28.09	27.67	27.50	27.34	27.06	26.82
Benzene	$7\ ^1A'$	28.75	28.06	27.64	27.47	27.32	27.04	26.79
Benzene	$1\ ^3A'$	27.31	27.02	26.78	26.67	26.56	26.36	26.18
Benzene	$2\ ^3A'$	27.21	26.91	26.67	26.56	26.46	26.26	26.08
Benzene	$3\ ^3A'$	27.21	26.91	26.66	26.55	26.44	26.24	26.06
Benzene	$4\ ^3A'$	27.61	27.34	27.10	26.99	26.89	26.70	26.52
Naphthalene	$1\ ^1B_{3u}$	42.04	41.75	41.53	41.43	41.33	41.14	40.96
Naphthalene	$1\ ^1B_{2u}$	44.00	43.71	43.47	43.36	43.26		42.89
Naphthalene	$2\ ^1A_g$	43.28	42.11	41.71	41.54	41.40	41.13	40.88
Naphthalene	$1\ ^1B_{1g}$	43.03	42.54	42.22	42.08	41.95	41.70	41.47
Naphthalene	$2\ ^1B_{3u}$	44.78	44.29	43.88	43.72	43.55	43.29	43.02
Naphthalene	$2\ ^1B_{2u}$	44.08	43.79	43.53	43.40	43.28	43.06	
Naphthalene	$3\ ^1A_g$	43.26	42.33	41.92	41.75	42.67	42.45	42.26
Furan	$1\ ^1B_2$	26.50	26.17	26.03	25.90	25.74	25.54	25.36
Furan	$3\ ^1A_1$		23.69	23.18	22.96	22.77	22.42	22.12
Furan	$1\ ^3B_2$	23.71	23.57	23.45	23.39	23.34	23.23	23.13
Furan	$1\ ^3A_1$	24.06	23.85	23.68	23.60	23.52	23.38	23.25
Pyrrole	$1\ ^1B_2$	25.73	25.25	25.00	24.83	24.69	24.47	24.26
Pyrrole	$3\ ^1A_1$	26.02	25.46	25.07	24.90	24.75	24.47	24.22
Pyrrole	$1\ ^3B_2$	23.10	22.95	22.82	22.76	22.70	22.60	22.49
Pyrrole	$1\ ^3A_1$	23.61	23.38	23.19	23.10	23.02	22.86	22.71
Pyridine	$2\ ^1A_1$		28.54	28.22	28.00	27.98	27.72	27.49
Pyridine	$3\ ^1A_1$		29.21	28.86	29.07	28.55	28.27	28.02
Pyridine	$1\ ^1B_1$		26.38	26.24	26.17	25.77	25.64	25.52
Pyridine	$1\ ^1A_2$		26.81	26.66	26.59	26.87	26.76	26.64
Pyridine	$1\ ^3A_1$		26.03	25.86	25.78	25.71	25.56	25.43
Pyridine	$1\ ^3B_2$		26.87	26.72	26.65		26.35	26.18
Pyrazine	$1\ ^1B_{2u}$	27.96	27.56	27.31	27.21	27.11	26.92	26.75
Pyrazine	$1\ ^1B_{1u}$	29.37	29.09	28.86	28.75	28.65	28.46	28.28
Pyrazine	$2\ ^1B_{1u}$	30.55	30.14	29.83	29.68	29.55	29.29	29.06
Pyrazine	$2\ ^1B_{2u}$	30.26	29.87	29.55	29.40	29.26	29.00	28.77
Pyrazine	$1\ ^1B_{3u}$	28.24	28.00	27.81	27.72	27.64	27.48	27.33
Pyrazine	$1\ ^1B_{2g}$	27.62	27.39	27.20	27.12	27.04	26.88	26.73
Pyrazine	$1\ ^1B_{1g}$	28.91	28.51	28.22	28.08	27.96	27.73	27.52
Pyrimidine	$1\ ^1B_2$	27.60	27.35	27.14	27.05	26.96	26.79	26.63

Table S22: ... continued

**VQZP Basis Set**		**Dynamical Correlation $\widetilde{\mathbf{E}}^{\mathrm{dyn}}$ [eV]**						
**Molecule**	**State**	**0**	**0.10**	**0.20**	**0.25**	**0.30**	**0.40**	**0.50**
Pyrimidine	$2\,^1A_1$	29.69	29.21	28.96	28.84	28.74	28.54	28.36
Pyrimidine	$3\,^1A_1$	29.99	29.47	29.09	28.93	28.78	28.50	28.25
Pyrimidine	$2\,^1B_2$	30.54	30.14	29.81	29.67	29.53	29.27	29.03
Pyrimidine	$1\,^1B_1$	27.91	27.70	27.53	27.45	27.38	27.23	27.10
Pyrimidine	$1\,^1A_2$	28.27	28.05	27.86	27.78	27.70	27.54	27.39
Pyridazine	$2\,^1A_1$	27.55	27.29	27.09	26.99	26.90	26.72	26.56
Pyridazine	$1\,^1B_2$	29.82	29.40	29.08	28.95	28.82	28.58	28.37
Pyridazine	$2\,^1B_2$	30.56	30.10	29.70	29.54	29.39	29.11	28.85
Pyridazine	$3\,^1A_1$	30.60	30.24	30.07	29.72	29.56	29.28	29.03
Pyridazine	$1\,^1B_1$	28.41	28.17	27.98	27.90	27.82	27.66	27.51
Pyridazine	$2\,^1A_2$	28.21	27.94	27.72	27.63	27.53	27.35	27.18
Pyridazine	$2\,^1B_1$	28.51	28.14	27.90	27.79	27.69	27.50	27.32
Triazine	$2\,^1A'$	28.18	27.95	27.76	27.67	27.59	27.43	27.28
Triazine	$3\,^1A'$		31.81	31.53				
Triazine	$2\,^1A''$		29.29	29.03	28.92	28.81	28.60	28.41
Triazine	$3\,^1A''$		29.27	29.01	28.89	28.77	28.56	28.36
Triazine	$4\,^1A''$		28.84	28.59	28.47	28.36	28.15	27.95
Tetrazine	$1\,^1B_{2u}$	29.54	29.22	28.99	28.88	28.78	28.58	28.41
Tetrazine	$1\,^1B_{1u}$	33.07	32.67	32.36	32.23	32.09	31.85	31.62
Tetrazine	$2\,^1B_{1u}$	31.57	31.20	30.91	30.77	30.64	30.39	30.16
Tetrazine	$2\,^1B_{2u}$	30.70	30.22	29.89	29.74	29.60	29.34	29.10
Tetrazine	$1\,^1B_{3u}$	30.40	30.15	29.95	29.86	29.77	29.60	29.44
Tetrazine	$1\,^1A_u$	30.76	30.42	30.17	30.06	29.95	29.75	29.56
Tetrazine	$2\,^1B_{2g}$	30.45	30.06	29.75	29.61	29.48	29.22	28.99
Tetrazine	$2\,^1B_{1g}$	30.48	30.05	29.71	29.56	29.41	29.13	28.88
Tetrazine	$2\,^1B_{3u}$	30.61	30.30	29.93	29.79	29.67	29.42	29.20
Tetrazine	$1\,^3B_{3u}$	31.36	31.10	30.89	30.79	30.70	30.52	30.36
Tetrazine	$1\,^3A_u$	30.91	30.56	30.30	30.18	30.06	29.85	29.64
Tetrazine	$1\,^3B_{1g}$	30.29	29.98	29.75	29.64	29.54	29.35	29.17
Formaldehyde	$1\,^1A_2$	10.59	10.51	10.43	10.39	10.35	10.28	10.22
Formaldehyde	$1\,^3A_2$	10.69	10.58	10.48	10.43	10.39	10.30	10.22
Formaldehyde	$1\,^3A_1$	9.97	9.90	9.83	9.80	9.77	9.71	9.66
Acetone	$1\,^1A_2$	19.96	19.86	19.76	19.72	19.68	19.59	19.52
Acetone	$1\,^3A_2$	20.00	19.88	19.77	19.72	19.67	19.57	19.48
Acetone	$1\,^3A_1$	19.73	19.64	19.56	19.52	19.49	19.42	19.35
Benzoquinone	$1\,^1B_{1g}$	37.56	37.25	37.02	36.92	36.82	36.63	36.45
Benzoquinone	$1\,^1A_u$	37.50	37.21	36.99	36.89	36.80	36.61	36.43
Benzoquinone	$1\,^1B_{3g}$	38.89	38.50	38.25	38.14	38.03	37.84	37.65

Table S22: ... continued

VQZP Basis Set		Dynamical Correlation $\widetilde{\mathbf{E}}^{\mathrm{dyn}}$ [eV]						
**Molecule**	**State**	**0**	**0.10**	**0.20**	**0.25**	**0.30**	**0.40**	**0.50**
Benzoquinone	$1\,^1B_{1u}$	39.64	39.28	39.06	38.94	38.83	38.63	38.45
Benzoquinone	$1\,^3B_{1g}$	37.51	37.17	36.93	36.82	36.72	36.53	36.34
Benzoquinone	$1\,^3A_u$	37.43	37.12	36.89	36.79	36.69	36.50	36.32
Formamide	$1\,^1A''$	16.66	16.57	16.49	16.45	16.41	16.34	16.27
Formamide	$2\,^1A'$	19.43	19.10	18.88	18.77	18.68	18.50	18.33
Formamide	$1\,^3A''$	16.79	16.69	16.59	16.55	16.51	16.43	16.36
Acetamide	$1\,^1A''$	21.69		21.52	21.48	21.44	21.36	21.28
Acetamide	$2\,^1A'$	23.64		24.10	23.98	23.87	23.67	23.50
Acetamide	$3\,^1A'$	24.93		24.37	24.23	24.11	23.90	23.71
Propanamide	$1\,^1A''$	26.72	26.63	26.54	26.50	26.46	26.38	26.31
Propanamide	$2\,^1A'$	28.74	28.56	28.40	28.32	28.26	28.12	28.00
Propanamide	$3\,^1A'$	29.18	28.99	28.84	28.76	28.70	28.57	28.45
Cytosine	$2\,^1A'$	41.63		41.34	41.25	41.16	41.01	40.86
Cytosine	$1\,^1A''$	41.33		40.90	40.73	40.73	40.57	40.42
Cytosine	$3\,^1A'$	42.41		41.84	41.72	41.60	41.39	41.18
Cytosine	$4\,^1A'$	43.22		42.82	42.63	42.47	42.22	41.94
Cytosine	$5\,^1A'$	42.87		42.19	42.03	41.90	41.64	41.40
Thymine	$1\,^1A''$	46.49	46.31	46.17	46.11	46.05	45.93	45.82
Thymine	$2\,^1A'$	48.67	48.22	47.97	47.83	47.86	47.65	47.47
Thymine	$3\,^1A'$	48.42	48.28	47.70	47.56	47.57	47.32	47.11
Thymine	$4\,^1A'$	49.13	48.57	48.22				
Thymine	$5\,^1A'$	49.24		48.38	48.25	48.22	48.03	47.64
Uracil	$1\,^1A''$	41.44	41.24	41.10	41.03	40.97	40.85	40.74
Uracil	$2\,^1A'$	43.23	42.94	42.73	42.63	42.54	42.36	42.19
Uracil	$3\,^1A'$	43.14	42.82	42.60	42.49	42.40	42.21	42.04
Uracil	$4\,^1A'$	43.74	43.36	43.09	42.96	42.84	42.62	42.42
Uracil	$5\,^1A'$	43.90	43.45	43.14	43.00	42.87	42.63	42.41
Adenine	$2\,^1A'$			50.14	50.02	49.91	49.70	
Adenine	$3\,^1A'$			48.88	48.75	48.64	48.41	

S3.7 Comparison to other Computational Results

In Table S24 we present the excitation energies for the molecules of the Thiel benchmark set obtained at various levels of theory using the TZVP basis set: NEVPT2 (partially and strongly contracted [PC, SC]),[56] coupled cluster (CCSD and CC3),[51] TD-DFT (BP86, B3LYP,

Table S23: Mean signed errors (MSEE) in eV of CASPT2 vertical excitation energies compared to experimental reference data for different ANO-RCC basis sets and IPEA shift values ε. MSEE computed as $\text{MSEE} = \sum_{i=1}^{\text{N}_{\text{States}}} \left(V_i^{\text{calc}} - V_i^{\text{exp}}\right) / \text{N}_{\text{States}}$

**Basis Set**	**IPEA Shift ε [a.u.]**						
**ANO-RCC**	**0**	**0.10**	**0.20**	**0.25**	**0.30**	**0.40**	**0.50**
MB	0.85	0.95	1.02	1.05	1.08	1.13	1.18
VDZ	0.18	0.34	0.46	0.52	0.56	0.66	0.74
VDZP	−0.13	0.09	0.25	0.32	0.38	0.51	0.63
VTZP	−0.29	−0.10	0.08	0.18	0.27	0.41	0.54
VQZP	−0.34	−0.08	0.07	0.15	0.20	0.34	0.46

and BHLYP),[57] DFT/MRCI (BHLYP),[57] and ADC [ADC(2)-s, ADC(3)].[58] In Table S25 we show the mean signed and unsigned errors of the excitation energies compared to experimental reference data (see also Figure 12 in the main paper).

Table S24: Vertical excitation energies in eV at various levels of theory computed using the TVZP basis set for the 28 organic molecules of the Thiel benchmark set.

TZVP Basis Set		NEVPT2		CC			TDDFT		DFT/	ADC	
Molecule	State	PC	SC	CCSD	CC3	BP86	B3LYP	BHLYP	MRCI	ADC(2)-s	ADC(3)
Ethene	$1\ ^1B_{1u}$	8.64	8.69	8.51	8.37	7.73	7.70	7.69	7.96	8.36	8.14
Ethene	$1\ ^3B_{1u}$	4.60	4.60	4.42	4.48	4.15	4.03	3.47	4.31	4.52	4.23
Butadiene	$1\ ^1B_u$	6.80	6.82	6.72	6.58	5.60	5.74	5.94	6.02	6.43	6.36
Butadiene	$2\ ^1A_g$	5.56	5.59	7.42	6.77	6.30	6.82	7.61	6.18	7.68	5.77
Butadiene	$1\ ^3B_u$	3.38	3.39	3.25	3.32	2.87	2.76	2.15	3.09	3.40	3.03
Butadiene	$1\ ^3A_g$	5.27	5.28	5.15	5.17	4.95	4.86	4.44	4.85	5.22	4.89
Hexatriene	$1\ ^1B_u$	4.84	4.96	5.72	5.58	4.50	4.69	4.93	4.95	5.35	5.35
Hexatriene	$2\ ^1A_g$	5.56	5.59	6.61	5.72	5.07	5.69	6.66	4.92	6.72	4.52
Hexatriene	$1\ ^3B_u$	2.73	2.74	2.62	2.69	2.21	2.09	1.35	2.45	2.79	2.38
Hexatriene	$1\ ^3A_g$	4.39	4.40	4.28	4.32	3.98	3.92	3.50	3.96	4.38	4.00
Octatetraene	$2\ ^1A_g$	4.72	4.74	5.99	4.97	4.19	4.84	5.83	4.01	5.93	3.73
Octatetraene	$1\ ^1B_u$	4.04	4.17	5.07	4.94	3.82	4.02	4.29	4.25	4.66	4.70
Octatetraene	$2\ ^1B_u$	5.86	5.89								
Octatetraene	$3\ ^1A_g$	5.97	6.24								
Octatetraene	$4\ ^1A_g$	6.67	6.71								
Octatetraene	$3\ ^1B_u$	8.35	8.40								
Octatetraene	$1\ ^3B_u$	2.32	2.33	2.23	2.30	1.81	1.68	0.72	2.04	2.41	1.97
Octatetraene	$1\ ^3A_g$	3.72	3.73	3.62	3.67	3.30	3.24	2.80	3.32	3.74	3.33
Cyclopropene	$1\ ^1B_1$	6.85	6.85	6.96	6.90	6.30	6.46	6.77	6.73	6.97	6.75
Cyclopropene	$1\ ^1B_2$	7.07	7.18	7.24	7.10	6.13	6.31	6.50	6.74	7.14	6.91
Cyclopropene	$1\ ^3B_2$	4.54	4.56	4.30	4.34	3.74	3.70	3.25	4.03	4.43	4.08
Cyclopropene	$1\ ^3B_1$	6.58	6.58	6.66	6.62	5.81	6.01	6.28	6.31	6.66	6.45
Cyclopentadiene	$1\ ^1B_2$	5.21	5.30	5.87	5.73	4.93	5.02	5.15	5.42	5.66	5.52

Table S24: ... continued

TZVP Basis Set		NEVPT2		CC			TDDFT		DFT/	ADC	
**Molecule**	**State**	**PC**	**SC**	**CCSD**	**CC3**	**BP86**	**B3LYP**	**BHLYP**	**MRCI**	**ADC(2)-s**	**ADC(3)**
Cyclopentadiene	$2\,^1A_1$	6.72	6.74	7.05	6.61	6.09	6.52	7.23	6.15	7.08	5.81
Cyclopentadiene	$3\,^1A_1$	8.22	8.51	8.95	8.69	8.04	8.15	8.29	8.16	8.85	7.79
Cyclopentadiene	$1\,^3B_2$	3.32	3.33	3.18	3.25	2.82	2.71	2.14	3.07	3.35	2.97
Cyclopentadiene	$1\,^3A_1$			5.07	5.09	4.82	4.75	4.38	4.78	5.19	4.83
Norbornadiene	$1\,^1A_2$	5.04	5.07	5.80	5.64	4.48	4.79	5.15	5.30	5.57	5.48
Norbornadiene	$1\,^1B_2$	5.79	5.84	6.69	6.49	5.02	5.52	6.22	6.12	5.70	6.45
Norbornadiene	$2\,^1B_2$	6.97	7.10	7.87	7.64	6.61	6.87	7.21	7.21	7.63	7.54
Norbornadiene	$2\,^1A_2$	7.03	7.07	7.87	7.71	6.56	6.86	7.40	7.33	7.67	7.63
Norbornadiene	$1\,^3A_2$	3.79	3.81	3.67	3.72	3.11	3.08	2.63	3.42	3.74	3.46
Norbornadiene	$1\,^3B_2$	4.30	4.31	4.09	4.16	3.71	3.62	3.07	3.85	4.24	3.91
Benzene	$2\,^1A'$	5.21	5.24	5.19	5.07	5.24	5.40	5.64	5.04	5.27	4.99
Benzene	$3\,^1A'$	6.40	6.47	6.74	6.68	6.00	6.10	6.15	6.31	6.64	6.47
Benzene	$4\,^1A'$	7.11	7.28	7.65	7.45	6.96	7.07	7.27	7.19	7.43	7.35
Benzene	$5\,^1A'$	7.11	7.28	7.65	7.45	6.96	7.07	7.27	7.19	7.43	7.35
Benzene	$6\,^1A'$	8.42	8.45	9.21	8.43	8.28	8.91	9.70	7.51	9.06	8.58
Benzene	$7\,^1A'$	8.42	8.45	9.21	8.43	8.28	8.91	9.70	7.51	9.06	8.58
Benzene	$1\,^3A'$	4.32	4.33	3.94	4.12	3.93	3.77	3.08	4.13	4.30	3.88
Benzene	$2\,^3A'$	4.98	5.00	4.97	4.90	4.60	4.70	4.79	4.69	5.13	4.62
Benzene	$3\,^3A'$	4.98	5.00	4.97	4.90	4.60	4.70	4.79	4.69	5.13	4.62
Benzene	$4\,^3A'$	5.47	5.54	6.00	6.04	4.94	5.09	5.26	5.57	6.05	5.73
Naphthalene	$1\,^1B_{3u}$	4.37	4.39	4.41	4.27	4.23	4.44	4.71	4.10	4.45	4.14
Naphthalene	$1\,^1B_{2u}$	4.37	4.47	5.21	5.03	4.08	4.35	4.65	4.60	4.93	4.90
Naphthalene	$2\,^1A_g$	6.23	6.27	6.23	5.98	5.85	6.18	6.63	5.65	6.22	5.54
Naphthalene	$1\,^1B_{1g}$	6.15	6.20	6.53	6.07	5.04	5.58	6.28	5.53	6.23	6.06

Table S24: ... continued

TZVP Basis Set		NEVPT2		CC		TDDFT			DFT/	ADC	
**Molecule**	**State**	**PC**	**SC**	**CCSD**	**CC3**	**BP86**	**B3LYP**	**BHLYP**	**MRCI**	**ADC(2)-s**	**ADC(3)**
Naphthalene	$2\,^1B_{3u}$	5.61	5.85	6.55	6.33	5.73	5.93	6.21	5.89	6.23	6.26
Naphthalene	$2\,^1B_{1g}$	6.22	6.41	6.97	6.79	6.17	6.32	6.65	6.26	6.22	6.63
Naphthalene	$2\,^1B_{2u}$	6.01	6.17	6.77	6.57	5.88	6.12	6.41	6.21	6.55	6.47
Naphthalene	$3\,^1A_g$	6.85	6.90	7.77	6.90	6.20	6.85	7.70	6.05	7.38	6.41
Naphthalene	$3\,^1B_{2u}$	7.81	8.09	8.77	8.44	7.53	7.87	8.45	7.84		
Naphthalene	$3\,^1B_{3u}$	7.92	7.98	9.03	8.12	8.00	8.65	9.84	7.38		
Naphthalene	$1\,^3B_{2u}$			2.99	3.11	2.76	2.69	2.06	2.97	3.26	2.85
Naphthalene	$1\,^3B_{3u}$			4.27	4.18	3.81	3.95	4.06	3.93	4.36	3.90
Naphthalene	$1\,^3B_{1g}$			4.44	4.47	4.19	4.17	3.90	4.25	4.63	4.21
Naphthalene	$2\,^3B_{2u}$			4.67	4.64	4.31	4.40	4.45	4.49	4.86	4.37
Naphthalene	$2\,^3B_{3u}$			5.10	5.11	4.03	4.22	4.42	4.65	5.08	4.82
Naphthalene	$1\,^3A_g$	5.80	5.95	5.57	5.52	5.25	5.33	5.31	5.18	5.74	5.24
Naphthalene	$2\,^3B_{1g}$	6.11	6.25	6.79	6.48	5.00	5.55	6.39	6.02	6.45	6.27
Naphthalene	$2\,^3A_g$	6.52	6.56	6.81	6.47	5.67	5.95	6.33	6.00	6.82	6.16
Naphthalene	$3\,^3A_g$			6.96	6.79	5.59	6.07	6.58	6.31	6.94	6.59
Naphthalene	$3\,^3B_{1g}$			7.04	6.76	6.30	6.56	6.85	6.41	7.24	6.49
Furan	$1\,^1B_2$	6.42	6.59	6.80	6.60	6.11	6.16	6.23	6.33	6.76	6.39
Furan	$2\,^1A_1$	6.75	6.79	6.89	6.62	6.38	6.70	7.22	6.32	6.85	6.48
Furan	$3\,^1A_1$	8.35	8.62	8.83	8.53	8.16	8.25	8.43	8.21	8.73	8.23
Furan	$1\,^3B_2$	4.33	4.36	4.10	4.17	3.85	3.71	2.70	3.91	4.35	3.84
Furan	$1\,^3A_1$	5.62	5.67	5.48	5.48	5.24	5.21	4.67	5.15	5.59	5.22
Pyrrole	$2\,^1A_1$	6.56	6.60	6.61	6.40	6.26	6.53	6.94	6.13	6.60	6.38
Pyrrole	$1\,^1B_2$	6.78	6.90	6.87	6.71	6.34	6.40	6.48	6.46	6.89	6.53
Pyrrole	$3\,^1A_1$	8.19	8.44	8.44	8.17	7.85	7.96	8.15	7.88	8.43	7.92

Table S24: ... continued

TZVP Basis Set		NEVPT2		CC			TDDFT		DFT/	ADC	
**Molecule**	**State**	**PC**	**SC**	**CCSD**	**CC3**	**BP86**	**B3LYP**	**BHLYP**	**MRCI**	**ADC(2)-s**	**ADC(3)**
Pyrrole	$1\,^3B_2$	4.73	4.74	4.41	4.48	4.18	4.07	3.63	4.23	4.66	4.21
Pyrrole	$1\,^3A_1$	5.68	5.70	5.54	5.51	5.24	5.25	5.19	5.19	5.67	5.26
Imidazole	$1\,^1A''$	6.97	7.00	7.01	6.82	5.91	6.46	7.09	6.35	6.74	6.46
Imidazole	$2\,^1A'$	6.80	6.85	6.80	6.58	6.29	6.45	6.65	6.29	6.73	6.49
Imidazole	$3\,^1A'$	6.85	6.99	7.27	7.10	6.86	7.04	7.35	6.82	7.26	6.98
Imidazole	$2\,^1A''$	8.01	8.06	8.15	7.93	7.18	7.45	8.16	7.63	7.80	7.72
Imidazole	$4\,^1A'$	8.39	8.68	8.70	8.45	8.12	8.27	8.45	8.22	8.60	8.12
Imidazole	$1\,^3A'$			4.62	4.69	4.33	4.24	3.82	4.41	4.86	4.40
Imidazole	$2\,^3A'$			5.83	5.79	5.39	5.44	5.33	5.43	5.98	5.52
Imidazole	$1\,^3A''$			6.43	6.37	5.53	5.83	6.12	5.92	6.38	6.26
Imidazole	$3\,^3A'$			6.56	6.55	5.92	5.95	5.99	6.22	6.71	6.29
Imidazole	$4\,^3A'$			7.54	7.42	6.76	6.93	7.21	7.14	7.60	7.20
Imidazole	$2\,^3A''$			7.76	7.51	6.35	6.86	7.81	7.32	7.61	7.50
Pyridine	$1\,^1B_2$	5.33	5.36	5.27	5.15	5.35	5.49	5.71	5.09	5.32	5.06
Pyridine	$2\,^1A_1$	7.09	7.17	6.94	6.85	6.21	6.31	6.37	6.47	6.83	6.58
Pyridine	$3\,^1A_1$	7.23	7.50	7.94	7.70	7.27	7.32	7.55	7.43	7.70	7.59
Pyridine	$2\,^1B_2$	7.10	7.38	7.81	7.59	7.13	7.30	7.56	7.27	7.59	7.46
Pyridine	$4\,^1A_1$	8.03	8.11	9.45	6.68					7.99	8.73
Pyridine	$3\,^1B_2$	8.53	8.58	9.64	8.77					8.84	8.99
Pyridine	$1\,^1B_1$	5.26	5.28	5.25	5.05	4.38	4.80	5.30	4.75	5.10	5.05
Pyridine	$1\,^1A_2$	5.46	5.50	5.73	5.50	4.48	5.11	6.03	5.41	5.37	5.80
Pyridine	$1\,^3A_1$	4.47	4.48	4.07	4.25	4.05	3.89	3.14	4.25	4.45	3.98
Pyridine	$1\,^3B_2$	4.94	4.97	4.91	4.86	4.42	4.51	4.57	4.60	5.06	4.55
Pyridine	$2\,^3A_1$			5.13	5.05	4.78	4.84	4.89	4.84	5.30	4.74

Table S24: . . . continued

TZVP Basis Set		NEVPT2		CC		TDDFT			DFT/	ADC	
**Molecule**	**State**	**PC**	**SC**	**CCSD**	**CC3**	**BP86**	**B3LYP**	**BHLYP**	**MRCI**	**ADC(2)-s**	**ADC(3)**
Pyridine	2 $^3B_2$			6.41	6.40	5.46	5.64	5.84	5.97	6.47	6.08
Pyridine	3 $^3A_1$			7.90	7.66	7.29	7.44	7.51	7.21	7.86	7.23
Pyridine	3 $^3B_2$			8.12	7.83	7.54	7.75	7.93	7.52	8.14	7.40
Pyridine	1 $^3B_1$			4.61	4.50	3.71	4.04	4.39	4.31	4.52	4.43
Pyridine	1 $^3A_2$			5.67	5.46	4.34	4.98	5.85	5.33	5.34	5.72
Pyrazine	1 $^1B_{2u}$	5.31	5.34	5.14	5.02	5.25	5.37	5.52	4.94	5.16	4.88
Pyrazine	1 $^1B_{1u}$	6.76	6.85	7.18	7.07	6.41	6.50	6.55	6.71	7.06	6.85
Pyrazine	2 $^1B_{1u}$	7.72	8.01	8.34	8.06	7.53	7.68	7.89	7.82	8.11	7.95
Pyrazine	2 $^1B_{2u}$	7.43	7.69	8.29	8.05	7.75	7.78	8.13	7.75	8.06	8.02
Pyrazine	1 $^1B_{3g}$	8.73	8.76	9.75	8.77					9.39	8.75
Pyrazine	2 ^1A_g	8.87	8.92	9.55	8.69					8.11	7.52
Pyrazine	1 $^1B_{3u}$	4.20	4.25	4.42	4.24	3.59	3.96	4.40	4.00	4.29	4.21
Pyrazine	1 ^1A_u	4.93	4.99	5.29	5.05	4.06	4.69	5.59	5.02	4.97	5.28
Pyrazine	1 $^1B_{2g}$	5.86	5.91	6.02	5.74	5.11	5.55	6.02	5.26	5.93	5.65
Pyrazine	1 $^1B_{1g}$	6.77	6.83	7.13	6.75	5.57	6.38	7.66	6.46	6.70	7.18
Pyrimidine	1 $^1B_2$	5.61	5.63	5.49	5.36	5.59	5.74	5.98	5.35	5.49	5.30
Pyrimidine	2 $^1A_1$	7.42	7.51	7.17	7.06	6.46	6.58	6.67	6.69	7.03	6.66
Pyrimidine	3 $^1A_1$	7.73	8.00	7.97	7.74	7.32	7.48	7.73	7.46	7.70	7.53
Pyrimidine	2 $^1B_2$	7.51	7.80	8.24	8.01	7.57	7.76	7.96	7.74	7.89	7.90
Pyrimidine	1 $^1B_1$	4.52	4.57	4.70	4.50	3.80	4.27	4.87	4.36	4.45	4.57
Pyrimidine	1 $^1A_2$	4.81	4.87	5.12	4.93	4.02	4.60	5.39	4.82	4.80	5.10
Pyridazine	2 $^1A_1$	5.46	5.48	5.35	5.22	5.46	5.61	5.83	5.16	5.37	5.10
Pyridazine	1 $^1B_2$	7.34	7.47	7.09	6.93	6.32	6.43	6.48	6.51	6.97	6.73
Pyridazine	2 $^1B_2$	7.25	7.50	7.79	7.55	7.10	7.24	7.45	7.25	7.58	7.43

Table S24: . . . continued

TZVP Basis Set		NEVPT2		CC			TDDFT		DFT/	ADC	
**Molecule**	**State**	**PC**	**SC**	**CCSD**	**CC3**	**BP86**	**B3LYP**	**BHLYP**	**MRCI**	**ADC(2)-s**	**ADC(3)**
Pyridazine	$3\,^1A_1$	7.38	7.70	8.11	7.82	7.39	7.50	7.76	7.53	7.89	7.64
Pyridazine	$1\,^1B_1$	3.92	3.96	4.11	3.92	3.15	3.58	4.10	5.29	3.91	3.92
Pyridazine	$1\,^1A_2$	4.57	4.61	4.76	4.49	3.54	4.18	5.03	4.25	4.41	4.68
Pyridazine	$2\,^1A_2$	5.89	5.95	6.00	5.74	5.01	5.44	6.05	5.29	5.83	5.70
Pyridazine	$2\,^1B_1$	6.68	6.74	6.70	6.41	5.45	6.09	6.99	6.15	6.40	6.64
Triazine	$2\,^1A'$	5.92	5.94	5.84	5.71	5.95	6.14	6.45	5.70	5.76	5.71
Triazine	$3\,^1A'$	7.21	7.36	7.51	7.41	6.87	7.01	7.12	7.02	7.34	6.86
Triazine	$4\,^1A'$	7.94	8.25	8.28	8.04	7.63	7.79	8.06	7.81	7.92	8.05
Triazine	$5\,^1A'$	9.01	9.08	10.24	9.44					8.64	9.31
Triazine	$1\,^1A''$	4.65	4.77	4.96	4.78	3.84	4.45	5.31	4.69	4.62	5.03
Triazine	$2\,^1A''$	4.88	4.94	4.98	4.76	4.08	4.45	5.16	4.56	4.73	4.79
Triazine	$3\,^1A''$	4.87	4.94	5.01	4.81	3.99	4.54	5.27	4.77	4.70	4.95
Triazine	$4\,^1A''$	4.87	4.94	5.01	4.81	3.99	4.54	5.27	4.77	4.70	4.95
Tetrazine	$1\,^1B_{2u}$	5.47	5.50	5.27	5.12	5.46	5.58	5.74	5.07	5.20	4.98
Tetrazine	$1\,^1B_{1u}$	6.67	6.93	7.66	7.45	6.82	6.90	6.88	7.08	7.54	7.18
Tetrazine	$2\,^1B_{1u}$	6.87	7.24	8.06	7.79	7.36	7.48	7.70	7.53	7.76	7.66
Tetrazine	$2\,^1B_{2u}$	8.33	8.40	8.88	8.51	8.09	8.26	8.57	8.26	8.59	7.77
Tetrazine	$2\,^1B_{3g}$	8.10	8.24	9.44	8.47	8.72	9.30	9.96	7.44	8.85	8.25
Tetrazine	$1\,^1B_{3u}$	2.41	2.47	2.71	2.53	1.85	2.24	2.72	2.35	2.52	2.48
Tetrazine	$1\,^1A_u$	3.76	3.82	4.07	3.79	2.86	3.51	4.40	3.70	3.72	3.96
Tetrazine	$1\,^1B_{1g}$	5.17	5.22	5.32	4.97	4.13	4.73	5.33	4.45	5.13	5.00
Tetrazine	$1\,^1B_{2g}$	5.53	5.57	5.70	5.34	4.79	5.29	5.80	4.75	5.57	4.95
Tetrazine	$1\,^1B_{3g}$	6.30	6.35							7.84	6.48
Tetrazine	$2\,^1A_u$	5.70	5.77	5.70	5.46	4.60	5.04	5.60	5.05	5.54	5.39

Table S24: ... continued

TZVP Basis Set		NEVPT2		CC			TDDFT		DFT/	ADC	
Molecule	State	PC	SC	CCSD	CC3	BP86	B3LYP	BHLYP	MRCI	ADC(2)-s	ADC(3)
Tetrazine	$2\ ^1B_{2g}$	6.28	6.35	6.76	6.23	5.24	5.99	7.27	5.68	6.34	6.56
Tetrazine	$2\ ^1B_{1g}$	6.83	6.89	7.25	6.87	5.87	6.64	7.73	6.00	6.93	6.57
Tetrazine	$2\ ^1B_{3u}$	7.11	7.18	6.99	6.67	5.64	6.29	7.22	6.41	6.73	6.82
Tetrazine	$3\ ^1B_{1g}$	7.02	7.09	8.36	7.08	6.53	7.40	9.20	6.49	7.71	7.13
Tetrazine	$1\ ^3B_{3u}$	1.64	1.69	1.99	1.89	1.11	1.42	1.69	1.88	1.89	1.75
Tetrazine	$1\ ^3A_u$	3.42	3.46	3.74	3.52	2.52	3.10	3.76	3.50	3.47	3.58
Tetrazine	$1\ ^3B_{1g}$	4.33	4.37	4.31	4.21	3.32	3.63	3.90	3.92	4.29	4.03
Tetrazine	$1\ ^3B_{1u}$	4.55	4.56	4.05	4.33	4.24	3.83	2.51	4.27	4.60	3.93
Tetrazine	$1\ ^3B_{2u}$	4.72	4.76	4.57	4.54	4.11	4.06	3.91	4.21	4.79	4.05
Tetrazine	$1\ ^3B_{2g}$	5.19	5.22	5.09	4.93	4.17	4.48	4.76	4.64	5.04	4.70
Tetrazine	$2\ ^3A_u$	5.03	5.11	5.20	5.03	4.00	4.43	4.99	4.84	5.08	4.98
Tetrazine	$1\ ^3B_{3g}$	7.81	5.64								
Tetrazine	$2\ ^3B_{1u}$	5.51	5.54	5.48	5.38	5.12	5.24	5.36	5.26	5.64	5.00
Tetrazine	$2\ ^3B_{2g}$	6.11	6.18	6.51	6.04	4.77	5.62	6.91	5.78	6.09	6.12
Tetrazine	$2\ ^3B_{1g}$	6.55	6.62	7.11	6.60	5.61	6.33	7.53	6.27	6.75	6.43
Tetrazine	$2\ ^3B_{3u}$	6.72	6.78	6.80	6.53	5.36	5.97	6.83	6.36	6.54	6.64
Tetrazine	$2\ ^3B_{2u}$	6.40	6.54	7.46	7.36	6.42	6.63	6.94	6.94	7.59	6.07
Formaldehyde	$1\ ^1A_2$	4.22	4.22	3.97	3.95	3.80	3.89	4.00	3.71	3.91	3.82
Formaldehyde	$1\ ^1B_1$	9.40	9.40	9.26	9.18	8.80	8.89	9.09	8.76	7.67	8.96
Formaldehyde	$2\ ^1A_1$	8.79	9.08	10.54	10.45	9.95	9.17	9.32	9.19	9.37	9.09
Formaldehyde	$1\ ^3A_2$	3.75	3.75	3.52	3.55	3.05	3.13	3.21	3.32	3.41	3.43
Formaldehyde	$1\ ^3A_1$	6.06	6.05	5.78	5.89	5.48	5.18	4.38	5.46	5.96	5.46
Acetone	$1\ ^1A_2$	4.47	4.49	4.43	4.40	4.21	4.34	4.55	4.23	4.30	4.38
Acetone	$1\ ^1B_1$	9.50	9.59	9.26	9.17	8.15	8.60	9.06	8.56	9.12	9.14

Table S24: ... continued

TZVP Basis Set		NEVPT2		CC		TDDFT			DFT/	ADC	
**Molecule**	**State**	**PC**	**SC**	**CCSD**	**CC3**	**BP86**	**B3LYP**	**BHLYP**	**MRCI**	**ADC(2)-s**	**ADC(3)**
Acetone	2 $^1A_1$	9.28	9.58	9.87	9.65	8.76	9.04	8.97	8.53	9.44	9.96
Acetone	1 $^3A_2$	4.10	4.13	4.03	4.05	3.56	3.69	3.84	3.85	3.88	4.00
Acetone	1 $^3A_1$	6.06	6.04	5.94	6.03	5.57	5.39	4.81	5.64	6.12	5.71
Benzoquinone	1 $^1B_{1g}$	3.00	3.06	3.19	2.85	1.89	2.43	3.04	2.22	2.67	2.83
Benzoquinone	1 ^1A_u	2.99	3.04	3.07	2.75	2.02	2.58	2.55	2.29	2.76	2.98
Benzoquinone	1 $^1B_{3g}$	4.35	4.43	4.93	4.59	3.36	3.73	4.25	3.99	4.80	4.29
Benzoquinone	1 $^1B_{1u}$	4.85	5.02	5.89	5.62	4.49	4.83	5.31	5.07	5.42	5.43
Benzoquinone	1 $^1B_{3u}$	5.88	5.96	6.55	5.82	4.39	5.43	6.80	5.81	5.62	5.33
Benzoquinone	2 $^1B_{3g}$	6.70	6.82	7.62	7.27	6.12	6.59	7.32	6.71	7.25	6.94
Benzoquinone	2 $^1B_{1u}$	7.72	7.78	8.47	7.82	6.82	7.25	7.73	7.60		
Benzoquinone	1 $^3B_{1g}$	2.82	2.88	2.71	2.51	1.44	1.92	2.42	2.21	2.35	2.57
Benzoquinone	1 ^3A_u	2.82	2.89	2.83	2.62	1.56	2.05	3.20	2.31	2.44	2.72
Benzoquinone	1 $^3B_{1u}$			2.89	2.96	2.42	2.19	1.21	2.62	3.07	2.63
Benzoquinone	1 $^3B_{3g}$			3.42	3.41	2.59	2.68	2.44	3.09	3.52	3.06
Formamide	1 $^1A''$	5.93	5.93	5.66	5.65	5.46	5.55	5.77	5.47	5.46	5.69
Formamide	2 $^1A'$	7.58	7.81	8.52	8.27	7.90	8.13	8.84	8.14	7.82	7.48
Formamide	3 $^1A'$	10.75	10.97	11.34	10.93	10.98	10.92	11.38	10.57	7.98	8.83
Formamide	1 $^3A''$	5.64	5.65	5.32	5.36	4.87	4.97	5.14	5.12	5.13	5.36
Formamide	1 $^3A'$			5.67	5.74	5.20	5.13	4.93	5.42	5.81	5.51
Acetamide	1 $^1A''$	5.97	5.96	5.71	5.69	5.41	5.56	5.86	5.48	5.48	5.78
Acetamide	2 $^1A'$	7.48	7.69	7.85	7.67	7.50	7.46	8.14	7.51	7.47	7.59
Acetamide	3 $^1A'$	10.28	10.50	10.77	10.50	9.42	10.01	10.59	9.98	8.33	6.09
Acetamide	1 $^3A''$			5.39	5.42	4.85	5.01	5.25	5.13	5.16	5.46
Acetamide	1 $^3A'$			5.83	5.88	5.26	5.26	5.14	5.52	5.92	5.70

Table S24: ... continued

**TZVP Basis Set**		**NEVPT2**		**CC**			**TDDFT**		**DFT/**	**ADC**	
**Molecule**	**State**	**PC**	**SC**	**CCSD**	**CC3**	**BP86**	**B3LYP**	**BHLYP**	**MRCI**	**ADC(2)-s**	**ADC(3)**
Propanamide	$1\,^1A''$	5.99	5.99	5.74	5.72	5.43	5.59	5.89	5.47	5.49	5.81
Propanamide	$2\,^1A'$	7.40	7.61	7.80	7.62	7.28	7.76	8.09	7.46	7.39	7.58
Propanamide	$3\,^1A'$	10.16	10.37	10.34	10.06	8.17	9.00	10.07	9.51	7.94	8.08
Propanamide	$1\,^3A''$			5.41	5.45	4.89	5.04	5.29	5.13	5.18	5.49
Propanamide	$1\,^3A'$			5.84	5.90	5.27	5.28	5.18	5.51	5.92	5.73
Cytosine	$2\,^1A'$	4.70	4.78	4.98	4.72	4.20	4.64	5.19	4.62	4.60	5.83
Cytosine	$1\,^1A''$	5.50	5.56	5.45	5.16	3.79	4.76	6.21	4.86	4.81	5.42
Cytosine	$3\,^1A'$	5.65	5.74	5.95	5.61	4.92	5.42	6.16	5.43	5.56	5.76
Cytosine	$4\,^1A'$	6.47	6.70	6.81	6.61	6.49	6.72	6.99	6.38	6.43	6.64
Cytosine	$2\,^1A''$	5.73	5.80	5.99	5.52	4.49	5.11	5.64	5.32	5.24	6.16
Cytosine	$5\,^1A'$	6.83	6.98	7.23		6.37	6.46	7.44	6.74		
Cytosine	$6\,^1A'$	8.06	8.21	8.69							
Thymine	$1\,^1A''$	4.96	5.04	5.14	4.94	4.09	4.70	5.30	4.48	4.67	5.22
Thymine	$2\,^1A'$	5.05	5.18	5.60	5.34	4.60	5.00	5.48	5.18	5.30	5.35
Thymine	$3\,^1A'$	6.32	6.43	6.78	6.34	5.33	5.97	6.94	5.98	6.29	6.57
Thymine	$2\,^1A''$	6.49	6.57	6.57	6.59	4.79	5.80	6.77	5.93	6.09	6.52
Thymine	$4\,^1A'$	6.44	6.64	7.05	6.71	5.85	6.31	7.03	6.42	6.72	6.80
Thymine	$3\,^1A''$	6.69	6.89	7.67		5.33	6.21	7.70	6.43	6.58	6.76
Thymine	$5\,^1A'$	7.41	7.56	7.90		6.93	7.47	8.17	7.36		
Uracil	$1\,^1A''$	4.92	4.99	5.11	4.90	3.97	4.63	5.27	4.41	4.64	5.19
Uracil	$2\,^1A'$	5.27	5.39	5.70	5.44	4.77	5.19	5.67	5.33	5.41	5.44
Uracil	$3\,^1A'$	6.22	6.33	6.76	6.29	5.21	5.87	6.90	5.92	6.26	6.53
Uracil	$2\,^1A''$	6.42	6.49	7.68	6.32	4.76	5.74	6.61	5.84	6.01	6.68
Uracil	$3\,^1A''$	6.70	6.89	6.50	6.84	5.23	6.14	7.72	6.43	6.59	6.71

Table S24: ... continued

TZVP Basis Set		NEVPT2		CC			TDDFT		DFT/	ADC	
**Molecule**	**State**	**PC**	**SC**	**CCSD**	**CC3**	**BP86**	**B3LYP**	**BHLYP**	**MRCI**	**ADC(2)-s**	**ADC(3)**
Uracil	$4\,^1A'$	6.68	6.86	7.19	6.84	6.01	6.50	7.21	6.56	6.89	6.94
Uracil	$4\,^1A''$	7.27	7.42	7.74	7.12	6.11	6.64	7.91	6.79	6.95	7.85
Uracil	$5\,^1A'$	7.38	7.64	7.81		7.06	7.45	8.09	7.31	7.40	7.76
Adenine	$2\,^1A'$	5.07	5.22	5.37	5.18	4.99	5.27	5.67	4.99	5.20	5.19
Adenine	$3\,^1A'$	5.43	5.46	5.61	5.39	4.57	5.00	5.48	5.15	5.33	5.30
Adenine	$1\,^1A''$	5.36	5.43	5.58	5.34	4.30	4.97	5.81	5.11	5.19	5.56
Adenine	$4\,^1A'$	6.45	6.68	6.83	6.53	5.84	6.32	6.87	6.29	6.49	6.56
Adenine	$5\,^1A'$	6.82	6.95	7.17		6.27	6.69	7.30	6.19	6.81	6.60
Adenine	$2\,^1A''$	6.07	6.16	6.19	5.96	5.05	5.61	6.34	5.72	5.84	6.13
Adenine	$6\,^1A'$	6.95	7.01	7.72		6.65	7.08	7.71	7.10		
Adenine	$7\,^1A'$	7.72	7.83	8.47		6.91	7.52	8.22	6.62		

Table S25: Mean signed error (MSEE) and mean unsigned error (MUEE) in eV for vertical excitation energies V_i^{calc} computed at different levels of theory using the TZVP basis set compared to experimental excitation energies V_i^{exp} of the organic molecules from Thiel's benchmark set.

**Method**	**MSEE**[a]	**MUEE**[b]	**Ref.**
CASPT2 ($\varepsilon = 0$)	−0.13	0.33	this work
CASPT2 ($\varepsilon = 0.25$)	0.29	0.33	this work
PC-NEVPT2[c]	0.37	0.43	56
SC-NEVPT2[c]	0.46	0.49	56
CCSD	0.64	0.65	51
CC3	0.44	0.44	51
ADC(2)-s	0.36	0.45	58
ADC(3)	0.25	0.39	58
TDDFT (BP86)	−0.17	0.41	57
TDDFT (B3LYP)	0.10	0.36	57
TDDFT (BHLYP)	0.42	0.69	57
DFT/MRCI (BHLYP)	0.13	0.28	57

[a]mean signed error of excitation energies computed as MSEE $= \sum_{i=1}^{\text{N}_{\text{States}}} \left(V_i^{\text{calc}} - V_i^{\text{exp}}\right) / \text{N}_{\text{States}}$

[b]mean unsigned error of excitation energies computed as MUEE $= \sum_{i=1}^{\text{N}_{\text{States}}} \left(|V_i^{\text{calc}} - V_i^{\text{exp}}|\right) / \text{N}_{\text{States}}$

[c]obtained using the state-specific Fock operator

References

(1) Andersson, K.; Malmqvist, P.-Å.; Roos, B. O. J. Chem. Phys. **1992**, 96, 1218–1226.

(2) Fülscher, M. P.; Andersson, K.; Roos, B. O. J. Phys. Chem. **1992**, 96, 9204–9212.

(3) Serrano-Andrés, L.; Merchán, M.; Nebot-Gil, I.; Roos, B. O.; Fülscher, M. P. J. Am. Chem. Soc. **1993**, 115, 6184–6197.

(4) Serrano-Andrés, L.; Lindh, R.; Roos, B. O.; Merchán, M. J. Phys. Chem. **1993**, 97, 9360–9368.

(5) Rubio, M.; Merchán, M.; Ortí, E.; Roos, B. O. Chem. Phys **1994**, 179, 395–409.

(6) Fülscher, M. P.; Roos, B. O. Theor. Chim. Acta **1994**, 87, 403–413.

(7) Roos, B. O.; Merchán, M.; McDiarmid, R.; Xing, X. J. Am. Chem. Soc. **1994**, 116, 5927–5936.

(8) Lorentzon, J.; Malmqvist, P.-Å.; Fülscher, M. P.; Roos, B. O. Theor. Chim. Acta **1995**, 91, 91–108.

(9) Borowski, P.; Fülscher, M. P.; Malmqvist, P.-Å.; Roos, B. O. Chem. Phys. Lett. **1995**, 237, 195–203.

(10) Merchán, M.; Roos, B. O. Theor. Chim. Acta **1995**, 92, 227–239.

(11) Andersson, K. Theor. Chim. Acta **1995**, 91, 31–46.

(12) Roos, B. O.; Andersson, K.; Fülscher, M. P.; Serrano-Andrés, L.; Pierloot, K.; Merchán, M.; Molina, V. J. Mol. Struct. Theochem **1996**, 388, 257–276.

(13) Serrano-Andrés, L.; Fülscher, M. P. J. Am. Chem. Soc. **1996**, 118, 12200–12206.

(14) Serrano-Andrés, L.; Fülscher, M. P.; Roos, B. O.; Merchán, M. J. Phys. Chem. **1996**, 100, 6484–6491.

(15) Serrano-Andrés, L.; Roos, B. O. J. Am. Chem. Soc. **1996**, 118, 185–195.

(16) Beck, M. E.; Rebentisch, R.; Hohlneicher, G.; Fülscher, M. P.; Serrano-Andrés, L.; Roos, B. O. J. Chem. Phys. **1997**, 107, 9464–9474.

(17) Molina, V.; Merchán, M.; Roos, B. O. J. Phys. Chem. A **1997**, 101, 3478–3487.

(18) Serrano-Andrés, L.; Fülscher, M. P.; Karlström, G. Int. J. Quant. Chem. **1997**, 65, 167–181.

(19) Fülscher, M. P.; Serrano-Andrés, L.; Roos, B. O. J. Am. Chem. Soc. **1997**, 119, 6168–6176.

(20) Malsch, K.; Rebentisch, R.; Swiderek, P.; Hohlneicher, G. Theor. Chem. Acc. **1998**, 100, 171–182.

(21) Serrano-Andrés, L.; Merchán, M.; Rubio, M.; Roos, B. O. Chem. Phys. Lett. **1995**, 295, 195–203.

(22) Finley, J. P.; Malmqvist, P.-Å.; Roos, B. O.; Serrano-Andrés, L. Chem. Phys. Lett. **1998**, 288, 299–306.

(23) Molina, V.; Merchán, M.; Roos, B. O. Spectrochim. Acta, Part A **1999**, 55, 433–446.

(24) Rubio, M.; Roos, B. O. Mol. Phys. **1999**, 96, 603–615.

(25) Molina, V.; Smith, B. R.; Merchán, M. Chem. Phys. Lett. **1999**, 309, 486–494.

(26) González-Luque, R.; Merchán, M.; Roos, B. O. Z. Phys. D **1996**, 36, 311–316.

(27) Borin, A. C.; Serrano-Andrés, L. J. Mol. Struct. (THEOCHEM) **1999**, 464, 121–128.

(28) Merchán, M.; Serrano-Andrés, L.; Slater, L. S.; Roos, B. O.; McDiarmid, R.; Xing, X. J. Phys. Chem. A **1999**, 103, 5468–5476.

(29) Cai, Z.-L.; Reimers, J. R. J. Phys. Chem. A **2000**, 104, 8389–8408.

(30) Kröhl, O.; Malsch, K.; Swiderek, P. Phys. Chem. Chem. Phys. **2000**, 2, 947–953.

(31) Borin, A. C.; Serrano-Andrés, L. Chem. Phys. **2000**, 262, 253–265.

(32) Molina, V.; Merchán, M. J. Phys. Chem. A **2001**, 105, 3745–3751.

(33) Roos, B. O.; Malmqvist, P.-Å.; Molina, V.; Serrano-Andrés, L.; Merchán, M. J. Chem. Phys. **2002**, 116, 7526–7535.

(34) Serrano-Andrés, L.; Pou-Amérigo, R.; Fülscher, M. P.; Borin, A. C. J. Chem. Phys. **2002**, 117, 1649–1659.

(35) Weber, J.; Malsch, K.; Hohlneicher, G. Chem. Phys. **2001**, 264, 275–318.

(36) Climent, T.; González-Luque, R.; Merchán, M. J. Phys. Chem. A **2003**, 107, 6995–7003.

(37) Borin, A. C.; Serrano-Andrés, L.; Ludwig, V.; Canuto, S. Phys. Chem. Chem. Phys. **2003**, 5, 5001–5009.

(38) Aquilante, F.; Barone, V.; Roos, B. O. J. Chem. Phys. **2003**, 119, 12323–12334.

(39) Murakami, A.; Kobayashi, T.; Goldberg, A.; Nakamura, S. J. Chem. Phys. **2004**, 120, 1245–1252.

(40) Rubio, M.; Merchán, M.; Pou-Amérigo, R.; Ortí, E. ChemPhysChem **2003**, 4, 1308–1315.

(41) Aquilante, F.; Cossi, M.; Crescenzi, O.; Scalmani, G.; Barone, V. Mol. Phys. **2003**, 101, 19465–1953.

(42) Balková, A.; Kucharski, S. A.; Bartlett, R. J. Chem. Phys. Lett. **1991**, 182, 511–518.

(43) Koch, H.; Christiansen, O.; Jørgensen, P.; Olsen, J. Chem. Phys. Lett. **1995**, 244, 75–82.

(44) Christiansen, O.; Koch, H.; Jørgensen, P.; Olsen, J. Chem. Phys. Lett. **1996**, 256, 185–194.

(45) Larsen, H.; Hald, K.; Olsen, J.; Jørgensen, P. J. Chem. Phys. **2001**, 115, 3015–3020.

(46) Hald, K.; Jørgensen, P.; Olsen, J.; Jaszuński, M. J. Chem. Phys. **2001**, 115, 671–679.

(47) Li, X.; Paldus, J. J. Chem. Phys. **2003**, 119, 5346.

(48) Abrams, M. L.; Sherrill, C. D. J. Chem. Phys. **2004**, 121, 9211–9219.

(49) Pitarch-Ruiz, J.; Sánchez-Marín, J.; Velasco, A. M. J. Comput. Chem. **2008**, 29, 523–532.

(50) Huber, K. P.; Herzberg, G. In NIST Chemistry WebBook, NIST Standard Reference Database Number 69; Linstrom, P., Mallard, W., Eds.; National Institute of Standards and Technology, Gaithersburg MD, 20899, http://webbook.nist.gov, (retrieved November 19, 2015), 2015; Chapter Constants of Diatomic Molecules.

(51) Schreiber, M.; Silva-Junior, M. R.; Sauer, S. P. A.; Thiel, W. J. Chem. Phys. **2008**, 128, 134110.

(52) Schäfer, A.; Huber, C.; Ahlrichs, R. J. Chem. Phys. **1994**, 100, 5829–5853.

(53) Roos, B. O.; Lindh, R.; Malmqvist, P.-Å.; Veryazov, V.; Widmark, P.-O. J. Phys. Chem. A **2004**, 108, 2851–2858.

(54) Ghigo, G.; Roos, B. O.; Malmqvist, P.-Å. Chem. Phys. Lett. **2004**, 396, 142–149.

(55) Andersson, K.; Roos, B. O. Int. J. Quant. Chem. **1993**, 45, 591–607.

(56) Schapiro, I.; Sivalingam, K.; Neese, F. J. Chem. Theory. Comput. **2013**, 9, 3567–3580.

(57) Silva-Junior, M. R.; Schreiber, M.; Sauer, S. P. A.; Thiel, W. J. Chem. Phys. **2008**, 129, 104103.

(58) Harbach, P. H. P.; Wormit, M.; Dreuw, A. J. Chem. Phys. **2014**, 141, 064113.
